# Supplementary material for: Ketone α-alkylation at the more-hindered site
Source: Nat Commun. 2023 Jun 7;14:3326. doi: 10.1038/s41467-023-38741-w (PMC10247815; doi:10.1038/s41467-023-38741-w)
Supplement: Supplementary file 1 — Supplementary Information [file 41467_2023_38741_MOESM1_ESM.pdf]

Supplementary Information for:

# Ketone α-alkylation at the more-hindered site

Ming-Ming Li[1], Tianze Zhang[1], Lei Cheng[1], Wei-Guo Xiao[1], Jin-Tao Ma[1], Li-Jun Xiao[1,]* & Qi-Lin Zhou[1,]*

[1]State Key Laboratory and Institute of Elemento-Organic Chemistry, College of Chemistry, Frontiers Science Center for New Organic Matter, Nankai University, Tianjin 300071, China

*Correspondence to: ljxiao@nankai.edu.cn; qlzhou@nankai.edu.cn


## 1 Supplementary Methods

1.1 General Information…........………………………………………..........2

1.2 Preparation of Starting Materials and Ligands.............................................3

1.3. Optimization of Reaction Conditions.…………………..…………………11

## 2 Supplementary Discussion

2.1 Mechanistic Study………………………..............................................12

2.2 Typical Procedure of Nickel-Catalyzed Allylic Alkylation and Characterization Products.........................................................................17

2.3 Computational Details.....................................................................30

## 3 Supplementary Figures

3.1 NMR Spectra of New Compounds………...................................................39

## 4 Supplementary References.........................................................................101

# 1 Supplementary Methods

## 1.1 General Information

Unless mentioned otherwise, all manipulations were performed in an argon-filled glove box MBRAUN LABstar or using standard Schlenk techniques. NMR spectra were recorded on a Bruker AV 400 spectrometer at 400 MHz ($^{1}$H NMR), 101 MHz ($^{13}$C NMR), and Bruker AV 600 spectrometer at 600 MHz ($^{1}$H NMR), 151 MHz ($^{13}$C NMR). Chemical shifts were reported in ppm relative to internal TMS for $^{1}$H NMR data and deuterated solvent for $^{13}$C NMR data. Data are presented in the following space: chemical shift, multiplicity, coupling constant in hertz (Hz), and signal area integration in natural numbers. High-resolution mass spectra were recorded with ESI resource on an IonSpec FT-ICR mass spectrometer. All the solvents used for reactions were distilled under argon after drying over an appropriate drying agent. [Ni(COD)$_2$] was purchased from Strem Chemicals. Other commercially available reagents were purchased from Acros, Sigma-Aldrich, and Alfa Aesar chemical company. All of the liquid ketones for allylic alkylation were distilled under reduced pressure before use.

## 1.2 Preparation of Starting Materials and Ligands

### (a) Starting materials 5a-5f.

Allylic alcohols **5a**–**5f** were prepared according to the literature procedure.

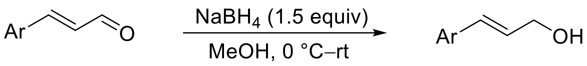


Cinnamic aldehyde (10.0 mmol) was dissolved in methanol (20 mL). $NaBH_4$ (0.57 g, 15.0 mmol) was added in portion-wise at 0 °C. The mixture was stirred at room temperature for about one hour until the starting material was consumed as monitored by TLC. Then the reaction was quenched with water and the mixture was diluted with dichloromethane. The layers were separated and the aqueous layer was extracted with dichloromethane, dried over $Na_2SO_4$, and concentrated. The crude product was purified by silica gel chromatography (petroleum ether/ethyl acetate = 4/1 to 1/1) to afford the pure allylic alcohol.

**(*E*)-3-phenylprop-2-en-1-ol (5a)**[1]

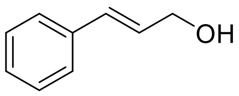


Colorless oil. 1.22 g; 91% yield. $^1$H NMR (400 MHz, $CDCl_3$) δ 7.34–7.19 (m, 5H), 6.55 (d, *J* = 15.9 Hz, 1H), 6.30 (dt, *J* = 15.9, 5.7 Hz, 1H), 4.24 (dd, *J* = 5.7, 1.6 Hz, 2H), 2.83 (s, 1H). $^{13}$C NMR (101 MHz, $CDCl_3$) δ 136.7, 130.9, 128.6, 128.6, 127.6, 126.5, 63.4.

**(*Z*)-3-phenylprop-2-en-1-ol (5b)**[2]

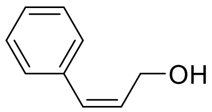


We used **5a** and **5b** to form 1:1 mixture of *Z*- and *E*-3-phenylprop-2-en-1-ol. Colorless oil. 1.22 g; 84% yield. $^1$H NMR (600 MHz, $CDCl_3$) δ 7.34 (t, *J* = 7.6 Hz, 2H), 7.26 (t, *J* = 7.1 Hz, 1H), 7.20 (d, *J* = 7.6 Hz, 2H), 6.56 (d, *J* = 11.7 Hz, 1H), 5.86 (dt, *J* = 12.3, 6.4 Hz, 1H), 4.43 (d, *J* = 6.5 Hz, 2H), 1.70 (s, 1H). $^{13}$C NMR (151 MHz, $CDCl_3$) δ 136.6, 131.3, 131.1, 128.9, 128.4, 127.4, 59.8.

**(*E*)-3-(2-methoxyphenyl)prop-2-en-1-ol (5c)**[3]

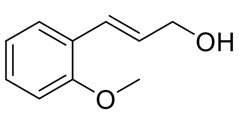


Colorless oil. 1.45 g; 88% yield. $^1$H NMR (400 MHz, $CDCl_3$) δ 7.41 (dd, *J* = 7.6, 1.7 Hz, 1H), 7.25–7.16 (m, 1H), 6.96–6.80 (m, 3H), 6.35 (dt, *J* = 16.0, 5.9 Hz, 1H), 4.28 (d, *J* = 5.8 Hz, 2H), 3.81 (s, 3H), 2.14 (s, 1H). $^{13}$C NMR (101 MHz, $CDCl_3$) δ 156.7, 129.4, 128.8, 127.0, 126.0, 125.8, 120.7, 110.9, 64.1, 55.4.

**(*E*)-3-(3-methoxyphenyl)prop-2-en-1-ol (5d)**[3]

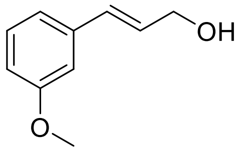


Colorless oil. 1.40 g; 85% yield. $^{1}$H NMR (400 MHz, $CDCl_3$) δ 7.26–7.19 (m, 1H), 6.98–6.94 (m, 1H), 6.92–6.89 (m, 1H), 6.81–6.77 (m, 1H), 6.56 (d, *J* = 15.9 Hz, 1H), 6.33 (dt, *J* = 15.9, 5.6 Hz, 1H), 4.29 (d, *J* = 5.6 Hz, 2H), 3.79 (s, 3H), 2.12 (s, 1H). $^{13}$C NMR (101 MHz, $CDCl_3$) δ 159.8, 138.2, 130.9, 129.6, 129.0, 119.2, 113.3, 111.9, 63.6, 55.3.

**(*E*)-3-(p-tolyl)prop-2-en-1-ol (5e)**[1]

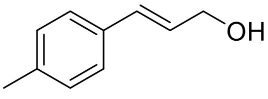


White solid. 1.32 g; 90% yield. mp: 51–52 ℃. $^{1}$H NMR (400 MHz, $CDCl_3$) δ 7.24 (d, *J* = 6.6 Hz, 2H), 7.09 (d, *J* = 7.8 Hz, 2H), 6.53 (d, *J* = 16.6 Hz, 1H), 6.27 (dt, *J* = 16.0, 5.8 Hz, 1H), 4.25 (d, *J* = 6.1 Hz, 2H), 2.38–2.24 (m, 4H). $^{13}$C NMR (101 MHz, $CDCl_3$) δ 137.5, 133.9, 131.1, 129.3, 127.5, 126.4, 63.7, 21.3.

**(*E*)-3-(4-fluorophenyl)prop-2-en-1-ol (5f)**[1]

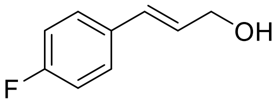


White solid. 1.25 g; 82% yield. mp: 56–57 ℃. $^{1}$H NMR (400 MHz, $CDCl_3$) δ 7.38–7.28 (m, 2H), 7.04–6.94 (m, 2H), 6.56 (d, *J* = 15.9 Hz, 1H), 6.26 (dt, *J* = 16.0, 5.7 Hz, 1H), 4.30 (d, *J* = 5.8 Hz, 2H), 2.02 (s, 1H). $^{19}$F NMR (376 MHz, $CDCl_3$) δ -114.34. $^{13}$C NMR (101 MHz, $CDCl_3$) δ 163.7, 161.2, 132.9 (two signals), 130.0, 128.3 (two signals), 128.1, 128.0, 115.7, 115.5, 63.6.

**(b) Starting materials 1ai, 1aj.**[4]

Preparation of substrate **1ai**.

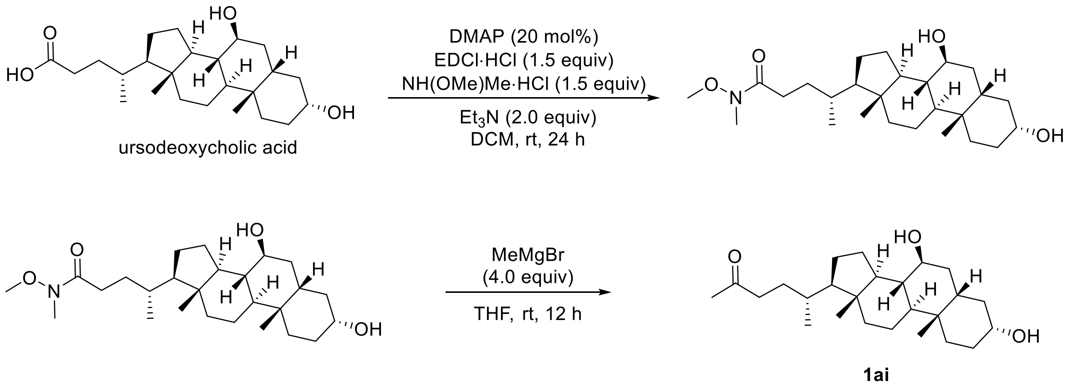


To a 250 mL round-bottomed flask, Ursodeoxycholic acid (1.96 g 5.0 mmol), DMAP (125 mg, 1.0 mmol), NH(OMe)Me·HCl (743 mg, 7.5 mmol), EDCI·HCl (1.5 g, 7.5 mmol), DCM (50 mL) and $Et_3N$ (1.5 mL, 10.0 mmol) were added sequentially.

The mixture was stirred at room temperature for 24 h. Upon completion, the reaction mixture was diluted with $H_2O$ (50 mL) and extracted with DCM (50 mL×3). The combined organic layers were dried over $MgSO_4$ and the solvent was evaporated. affording the crude amide product. A solution of the crude amide product in 20 mL THF was cooled to 0 °C, and a solution of MeMgBr (20.0 mL, 20.0 mmol, 1.0 M in THF) was added dropwise at 0 °C under argon atmosphere. The mixture was warmed to room temperature and stirred for 12 h. Upon completion, the reaction mixture was quenched by adding $NH_4Cl$ solution and extracted with EtOAc (30 mL×3). The combined organic layers were dried over $MgSO_4$ and the solvent was evaporated. The residue was purified by silica gel column chromatography (petroleum ether/ethyl acetate = 1/1 to 1/2), affording 1.4 g **1ai** with 72% yield. mp: 188–189 °C. $^1$H NMR (600 MHz, $CDCl_3$) δ 3.61–3.56 (m, 2H), 2.49–2.44 (m, 1H), 2.37–2.32 (m, 1H), 2.14 (s, 3H), 2.00–1.99 (m, 1H), 1.91–1.86 (m, 1H), 1.80–1.73 (m, 4H), 1.66–1.56 (m, 6H), 1.49–1.41 (m, 6H), 1.32–1.25 (m, 5H), 1.16–1.13 (m, 1H), 1.08–1.00 (m, 2H), 0.95–0.91 (m, 6H), 0.67 (s, 3H). $^{13}$C NMR (151 MHz, $CDCl_3$) δ 209.9, 71.5 (two signals), 55.8, 55.1, 43.9, 42.6, 40.8, 40.3, 39.3, 37.4, 37.0, 35.3, 35.1, 34.2, 30.5, 30.0 (two signals), 28.8, 27.0, 23.5, 21.3, 18.7, 12.3. HRMS (ESI) m/z: $[M+Na]^+$ calcd. for $C_{25}H_{42}NaO_3^+$: 413.3026. Found: 413.3031.

Preparation of substrate **1aj**.

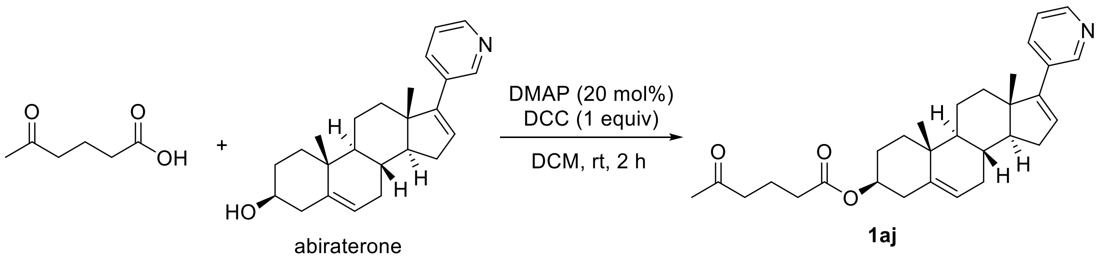


To a 50 mL round-bottomed flask, 5-oxohexanoic acid (260 mg, 2.0 mmol), Abiraterone (1.05 g, 3.0 mmol), DMAP (49 mg, 0.4 mmol), DCC (412 mg, 2.0 mmol), were added sequentially. The mixture was stirred at room temprature for 2 h. Upon completion, the reaction mixture was diluted with Sodium bicarbonate saturated

solution (20 mL) and extracted with DCM (20 mL×3). The combined organic layers were dried over $MgSO_4$ and the solvent was evaporated. The residue was purified by silica gel column chromatography (petroleum ether/ethyl acetate = 2/1 to 1/1), affording 780 mg **1aj** with 85% yield. mp: 110–111 °C. $^1$H NMR (400 MHz, $CDCl_3$) δ 8.62 (s, 1H), 8.46 (d, $J$ = 4.8 Hz, 1H), 7.65 (d, $J$ = 7.9 Hz, 1H), 7.24–7.21 (m, 1H), 6.00–5.99 (m, 1H), 5.43–5.41 (m, 1H), 4.67–4.59 (m, 1H), 2.51 (t, $J$ = 7.2 Hz, 2H), 2.35–2.30 (m, 4H), 2.15 (s, 3H), 2.15–2.03 (m, 3H), 1.91–1.86 (m, 4H), 1.79–1.47 (m, 9H), 1.19–1.16 (m, 1H), 1.08 (s, 3H), 1.05 (s, 3H). $^{13}$C NMR (101 MHz, $CDCl_3$) δ 208.1, 172.6, 151.7, 148.0 (two signals), 140.1, 133.7, 133.0, 129.3, 123.1, 122.4, 73.9, 57.5, 50.3, 47.4, 42.6, 38.2, 37.0, 36.9, 35.3, 33.7, 31.9, 31.6, 30.5, 30.1, 27.8, 20.9, 19.3, 19.1, 16.7. HRMS (ESI) m/z: $[M+Na]^+$ calcd. for $C_{30}H_{39}NNaO_3^+$: 484.2822. Found: 484.2825.

**(c) Synthesis of ligands L2-L6.**

Preparation of substrate [1,1'-biphenyl]-2,2'-diyl bis(trifluoromethanesulfonate) (**S1**)[5].

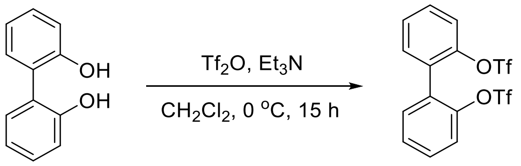


**S1**

In a flame-dried 100 mL round-bottom flask, 2,2´-biphenol (4.8 g, 25.8 mmol, 1.0 equiv) was dissolved in DCM (100 mL). The solution was cooled down to 0 °C and $Et_3N$ (11.00 mL, 78.6 mmol, 3.0 equiv) was added drop-wise. The reaction was stirred for 30 minutes at 0 °C and $Tf_2O$ (9.5 mL, 56.6 mmol, 2.2 equiv) was added drop-wise. Then, the reaction was refluxed for 15 hours. When completed, the mixture was cooled to room temperature and diluted with DCM. 1 N HCl solution was carefully added drop-wise and the layers were separated. The organic layer was washed with saturated $NaHCO_3$ and brine and dried over $MgSO_4$. The solvent was removed by evaporation, then the title compound was purified by column chromatography on silica gel (petroleum ether/ethyl acetate = 10/1) resulting in a white powder (11.30 g, 97% yield).

$^1$H NMR (400 MHz, $CDCl_3$) δ 7.49–7.40 (m, 8H). $^{13}$C NMR (101 MHz, $CDCl_3$) δ 147.0, 132.7, 130.9, 129.6, 128.7, 123.3, 121.8, 120.1, 116.9, 113.7.

Synthesis of ligand 2,2'-bis(bis(3,5-dimethylphenyl)phosphanyl)-1,1'-biphenyl (**L4**)

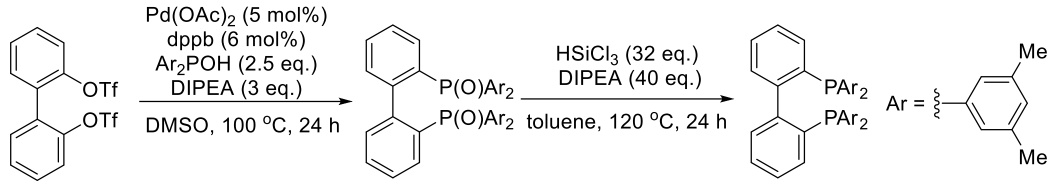


A 35 mL sealed tube with branch port was charged with $Pd(OAc)_2$ (6.5 mg, 0.03 mmol), dppb (17 mg, 0.04 mmol) and [1,1'-biphenyl]-2,2'-diyl bis(trifluoromethanesulfonate) (**S1**) (300 mg, 0.65 mmol), degassed DMSO (7 mL), $Ar_2POH$ (419 mg, 1.6 mmol) and DIPEA (348 µL, 2.0 mmol) sequentially. The mixture was stirred at 100 °C for 24 h after freeze degassing. Upon completion, the reaction mixture was cooled to room temperature and was diluted with EtOAc (10 mL) and $H_2O$ (15 mL), and extracted with EtOAc (30 mL×3). The combined organic layers were dried over $MgSO_4$ and the solvent was evaporated. The residue was purified by silica gel column chromatography, affording 320 mg phosphine oxide with 74% yield. A 50 mL sealed tube was charged with phosphine oxide (320 mg, 0.48 mmol), DIPEA (3.3 mL, 19.2 mmol, 40.00 equiv) degassed toluene (10 mL) and $HSiCl_3$ (11.00 mL, 15.4 mmol, 32.00 equiv) was added drop-wise, The mixture was stirred at 120 °C for 24 h. Upon completion, the reaction mixture was cooled to room temperature and was diluted with EtOAc (10 mL). The reaction was quenched with saturated NaOH solution and extracted with EtOAc (30 mL×3). The combined organic layers were dried over $MgSO_4$ and the solvent was evaporated. The residue was purified by silica gel column chromatography (petroleum ether/ethyl acetate = 50/1), affording 277 mg **L4** with 91% yield. mp: 90–91 °C. $^1$H NMR (400 MHz, $CDCl_3$) δ 7.24–7.15 (m, 4H), 7.09 (d, $J$ = 7.1 Hz, 2H), 7.00–6.99 (m, 2H), 6.93 (s, 2H), 6.85–6.84 (m, 6H), 6.67 (d, $J$ = 5.2 Hz, 4H), 2.24 (s, 12H), 2.15 (s, 12H). $^{31}$P NMR (162 MHz, $CDCl_3$) δ –14.74. $^{13}$C NMR (101

MHz, $CDCl_3$) δ 147.9, 147.7, 147.5, 137.9 (two signals), 137.8 (two signals), 137.6, 137.4 (three signal), 137.0, 136.9 (two signals), 134.4, 132.2, 132.1, 132.0, 131.9, 131.8, 131.5, 131.4, 131.3 (two signals), 131.2 (two signals), 130.3, 129.9, 127.7, 127.6, 21.5. HRMS (ESI) m/z: $[M+H]^+$ calcd. for $C_{44}H_{45}P_2^+$: 635.2991. Found: 635.2994.

Synthesis of ligand 2,2'-bis(bis(4-fluorophenyl)phosphanyl)-1,1'-biphenyl (**L2**)

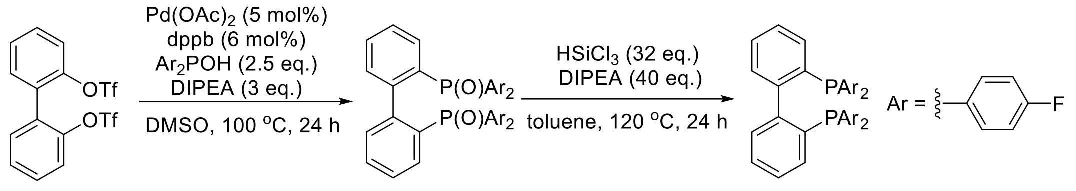


**S1** **L2**

The operations are the same as synthesis of the ligand **L4**, silica gel column chromatography (petroleum ether/ethyl acetate = 50/1), affording 380 mg of **L2**, 76% total yield. mp: 194–195 °C. $^1$H NMR (600 MHz, $CDCl_3$) δ 7.28–7.23 (m, 2H), 7.18–7.08 (m, 10H), 7.03–6.94 (m, 10H), 6.84–6.80 (m, 2H). $^{31}$P NMR (162 MHz, $CDCl_3$) δ –16.15. $^{19}$F NMR (376 MHz, CDCl3) δ –112.36, –113.08. $^{13}$C NMR (151 MHz, $CDCl_3$) δ 164.3, 164.1, 162.6, 162.5, 147.0, 146.9, 146.8, 136.7 (three signal), 136.2, 136.1, 136.0 (two signals), 135.4 (two signals), 135.3, 135.2, 133.4, 133.0 (two signals), 132.7 (two signals), 131.1, 131.0 (two signals), 128.4, 128.1. HRMS (ESI) m/z: $[M+H]^+$ calcd. for $C_{36}H_{25}F_4P_2^+$: 595.1362. Found: 595.1365.

Synthesis of ligand 2,2'-bis(bis(4-methoxyphenyl)phosphanyl)-1,1'-biphenyl (**L3**)

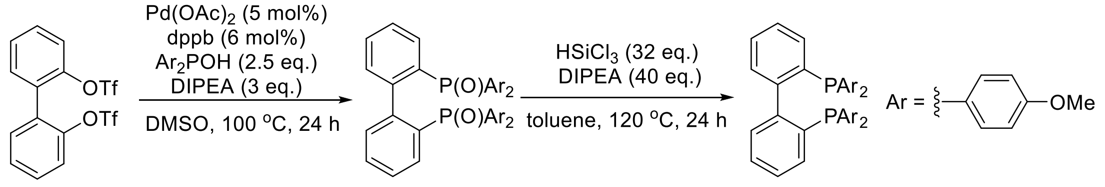


**S1** **L3**

The operations are the same as synthesis of the ligand **L4**, silica gel column chromatography (petroleum ether/ethyl acetate = 50/1), affording 420 mg of **L3** with 80% total yield. mp: 81–82 °C. $^1$H NMR (600 MHz, $CDCl_3$) δ 7.22 (t, $J$ = 7.4 Hz, 2H), 7.15–7.10 (m, 6H), 7.07–7.03 (m, 6H), 6.89–6.86 (m, 2H), 6.85–6.77 (m, 8H), 3.79 (s,

6H), 3.76 (s, 6H). $^{31}$P NMR (243 MHz, $CDCl_3$) δ –17.34. $^{13}$C NMR (151 MHz, $CDCl_3$) δ 160.1, 159.8, 147.1, 147.0, 146.9, 137.8, 137.7 (two signals), 135.8, 135.7 (two signals), 135.6, 135.1, 135.0, 134.9, 133.5, 131.1, 131.0 (two signals), 129.2 (two signals), 129.1, 128.9 (two signals), 128.8, 127.7, 127.6, 114.1, 114.0 (two signals), 55.3, 55.2. HRMS (ESI) m/z: $[M+H]^+$ calcd. for $C_{40}H_{37}O_4P_2^+$: 643.2162. Found: 643.2166.

Synthesis of ligand 2,2'-bis(bis(3,5-di-tert-butylphenyl)phosphanyl)-1,1'-biphenyl (**L5**)

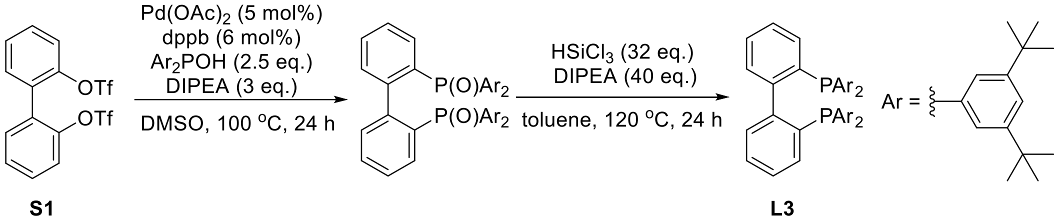


The operations are the same as synthesis of the ligand **L4**, silica gel column chromatography (petroleum ether/ethyl acetate = 50/1), affording 400 mg **L5** with 77% total yield. mp: 82–83 °C. $^{1}$H NMR (400 MHz, $CDCl_3$) δ 7.37–7.35 (m, 4H), 7.31–7.27 (m, 4H), 7.18–7.10 (m, 4H), 7.06–6.99 (m, 6H), 6.69 (d, $J$ = 6.3 Hz, 2H), 1.22–1.21 (m, 72H). $^{31}$P NMR (162 MHz, $CDCl_3$) δ –10.52. $^{13}$C NMR (101 MHz, $CDCl_3$) δ 150.1, 150.0 (two signals), 149.9 (three signal), 147.7, 147.5, 147.3, 139.0, 138.9 (two signals), 137.9, 137.8, 137.7, 137.1 (two signals), 137.0, 133.4, 130.6, 130.5 (two signals), 128.8, 128.7, 128.6, 128.4, 128.3, 128.2, 127.6, 127.2, 122.1, 121.7, 35.1, 34.9, 31.6 (two signals). HRMS (ESI) m/z: $[M+H]^+$ calcd. for $C_{68}H_{93}P_2^+$: 971.6747. Found: 971.6739.

Synthesis of ligand 2,2'-bis(bis(3,5-di-tert-butyl-4-methoxyphenyl)phosphanyl)-1,1'-biphenyl (**L6**)

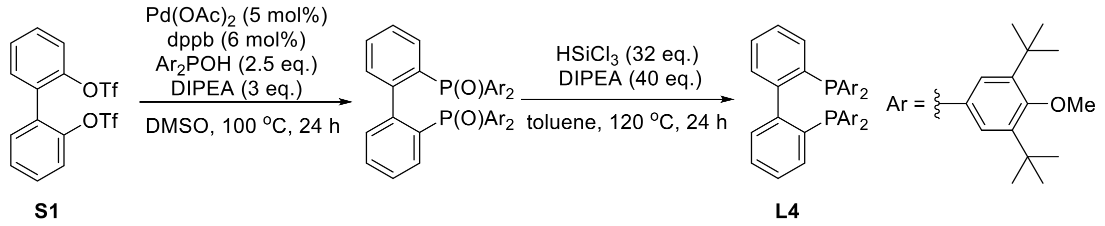


The operations are the same as synthesis of the ligand **L4**, silica gel column chromatography (petroleum ether/ethyl acetate = 50/1), affording 1.5g **L6** with 76% total yield, mp: 86–87 °C. $^{1}$H NMR (400 MHz, $CDCl_3$) δ 7.32–7.31 (m, 4H), 7.21–7.18 (m, 2H), 7.06–7.03 (m, 4H), 7.01–6.99 (m, 4H), 6.66 (d, $J$ = 6.4 Hz, 2H), 3.68 (s, 6H), 3.63 (s, 6H), 1.32 (s, 36H), 1.29 (s, 36H). $^{31}$P NMR (162 MHz, $CDCl_3$) δ –13.01. $^{13}$C NMR (101 MHz, $CDCl_3$) δ 160.0, 159.6, 147.4, 147.2, 147.0, 143.0 (two signals), 142.9, 142.8 (two signals), 142.7, 139.2 (two signals), 139.1, 133.1 (two signals), 132.9, 132.8, 132.5, 132.4, 132.3, 132.2, 132.1 (two signals), 131.6 (two signals), 131.5, 130.5 (two signals), 130.4, 127.6, 127.2, 64.4, 64.3, 36.0, 35.8, 32.3, 32.2. HRMS (ESI) m/z: $[M+H]^+$ calcd. for $C_{72}H_{101}P_2^+$: 1091.7170. Found: 1091.7174.

## 1.3 Optimization of Reaction Conditions of 1m[a]

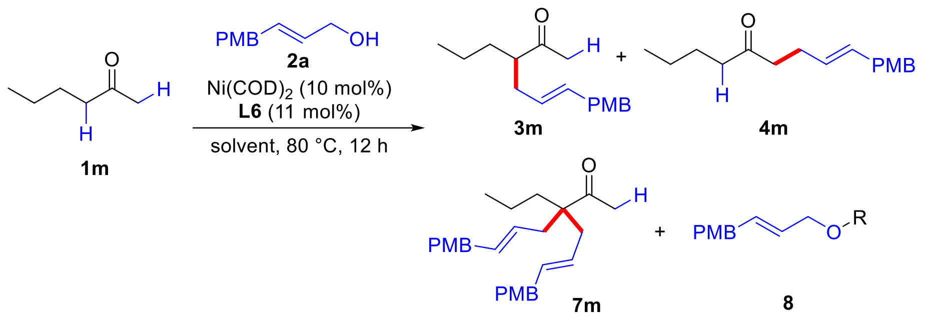


| entry | solvent | conv. (%) | **3m** yield (%) | **3m/4m** | **3m/7m** | **8** yield (%) |
|---|---|---|---|---|---|---|
| 1[b] | EtOH | 100 | 33 | 99:1 | 88:12 | 55 |
| 2 | EtOH | 100 | 65 | 99:1 | 88:12 | 24 |
| 3 | MeOH | 85 | 45 | 95:5 | 91:9 | 20 |
| 4 | $^{n}$PrOH | 100 | 57 | 99:1 | 88:12 | 28 |
| 5 | **$^{i}$PrOH** | 100 | **72** | **>99:1** | **99:1** | **16** |
| 6 | $^{n}$BuOH | 100 | 34 | 99:1 | 91:9 | 57 |
| 7 | Dioxane | 0 | 0 | - | - | - |
| 8 | Toluene | 0 | 0 | - | - | - |

**Supplementary Fig. 1.** Optimization of Reaction Conditions of **1m**. [a] **1m** (0.3 mmol), **2a** (0.1 mmol), solvent (0.5 mL). Ni(COD)$_2$ (10 mol %), ligand (11 mol %). Conversion rate is about **2a**. The regioisomeric ratio of **3m**/**4m** and chemoselectivity of **3m**/**7m** is determined by GC analysis. [b] **3m** (0.1 mmol), **2a** (0.1 mmol), EtOH was used as a solvent.

# 2 Supplementary Discussion

## 2.1 Mechanistic study

### (a) Deuterium labeling experiment

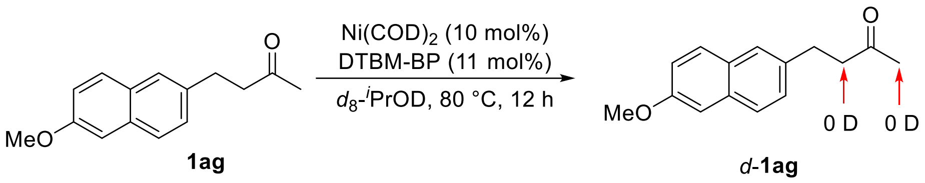


In an argon-filled glove box, an oven-dried tube was charged with a stir bar, catalyst precursor Ni(COD)$_2$ (2.75 mg, 0.010 mmol), ligand DTBM-BP (12.0 mg, 0.011 mmol), ketones **1ag**(0.30 mmol). The tube was sealed and removed from the glove box, and $d_8$-$^i$PrOD (0.5 mL) was injected into the tube under argon. The mixture was stirred at room temperature for 5 min and heated at 80 °C for 12 h. After cooling to room temperature, the solvent was removed under a vacuum. The pure product *d*-**1ag** was obtained by preparative TLC.

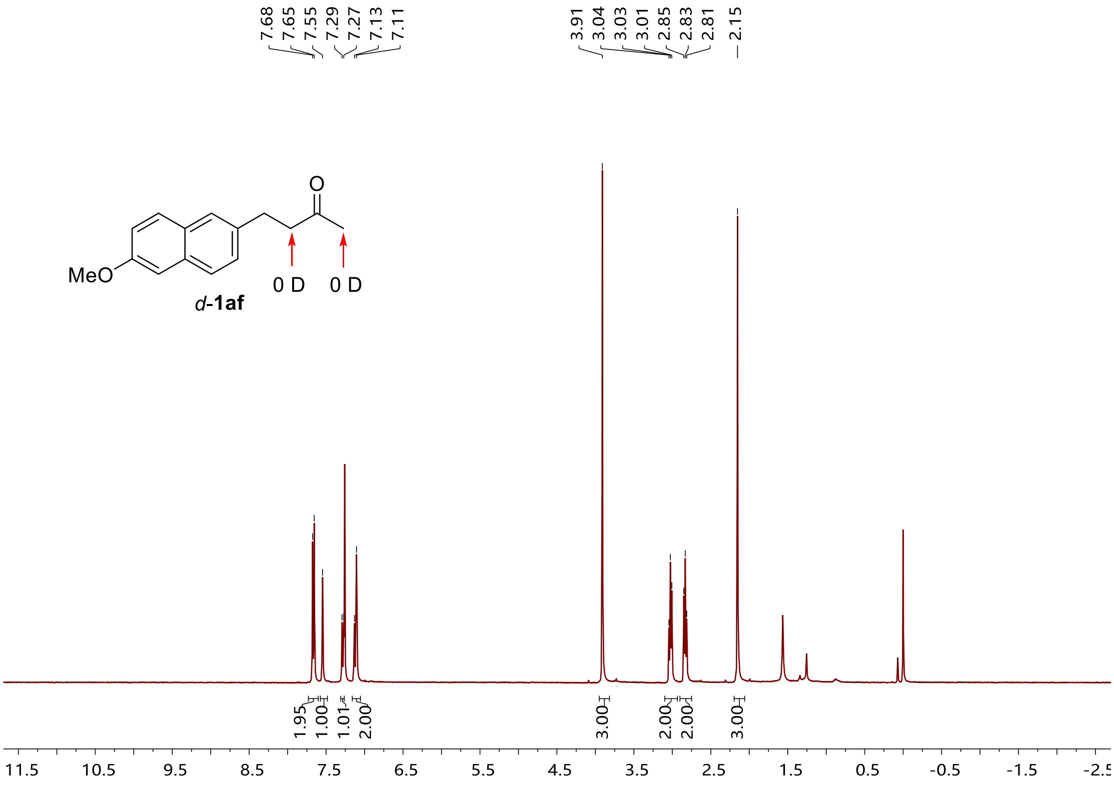


**Supplementary Fig. 2.** $^1$H NMR spectrum of *d*-**1ag**.

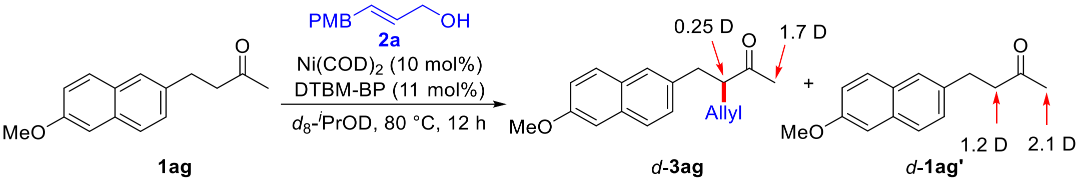


In an argon-filled glove box, an oven-dried tube was charged with a stir bar, catalyst precursor Ni(COD)$_2$ (2.75 mg, 0.010 mmol), ligand DTBM-BP (12.0 mg, 0.011 mmol), ketones **1ag** (0.30 mmol) and allyl alcohols **2a** (0.10 mmol). The tube was sealed and removed from the glove box, and $d_8$-$^i$PrOD (0.5 mL) was injected into the tube under argon. The mixture was stirred at room temperature for 5 min and heated at 80 °C for 12 h. After cooling to room temperature, the solvent was removed under a vacuum. The pure product ***d*-1ag′** and ***d*-3ag** was obtained by preparative TLC.

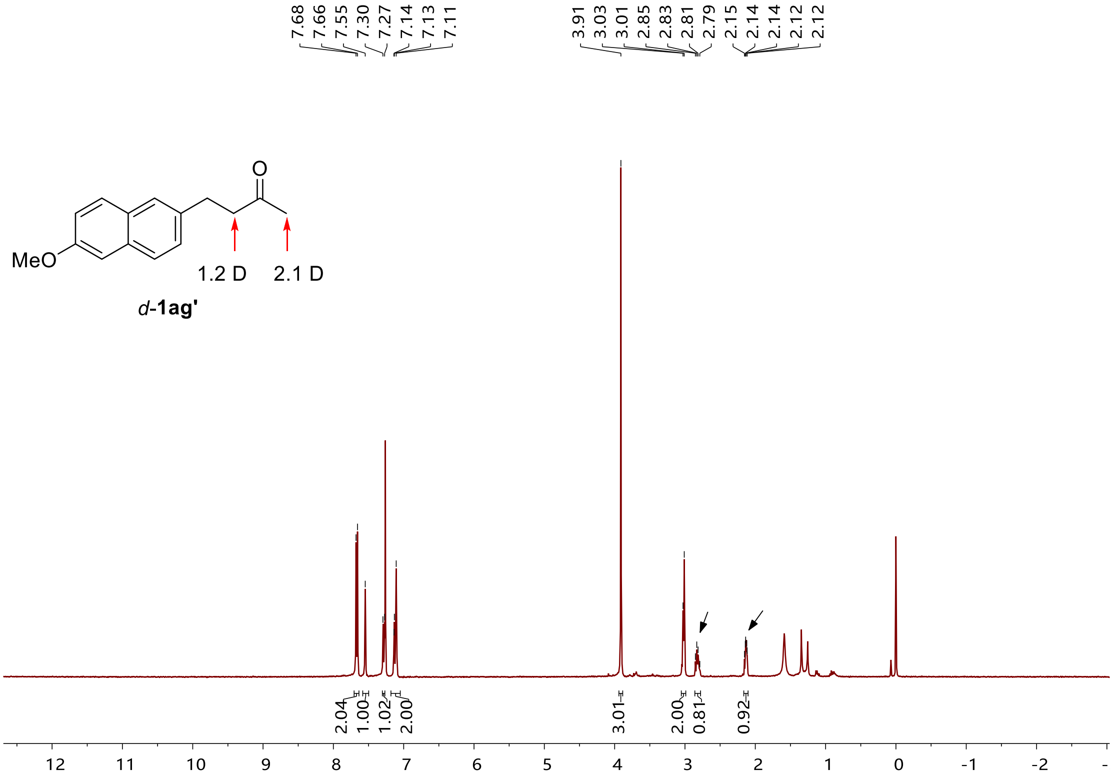


**Supplementary Fig. 3.** $^1$H NMR spectrum of ***d*-1ag′**.

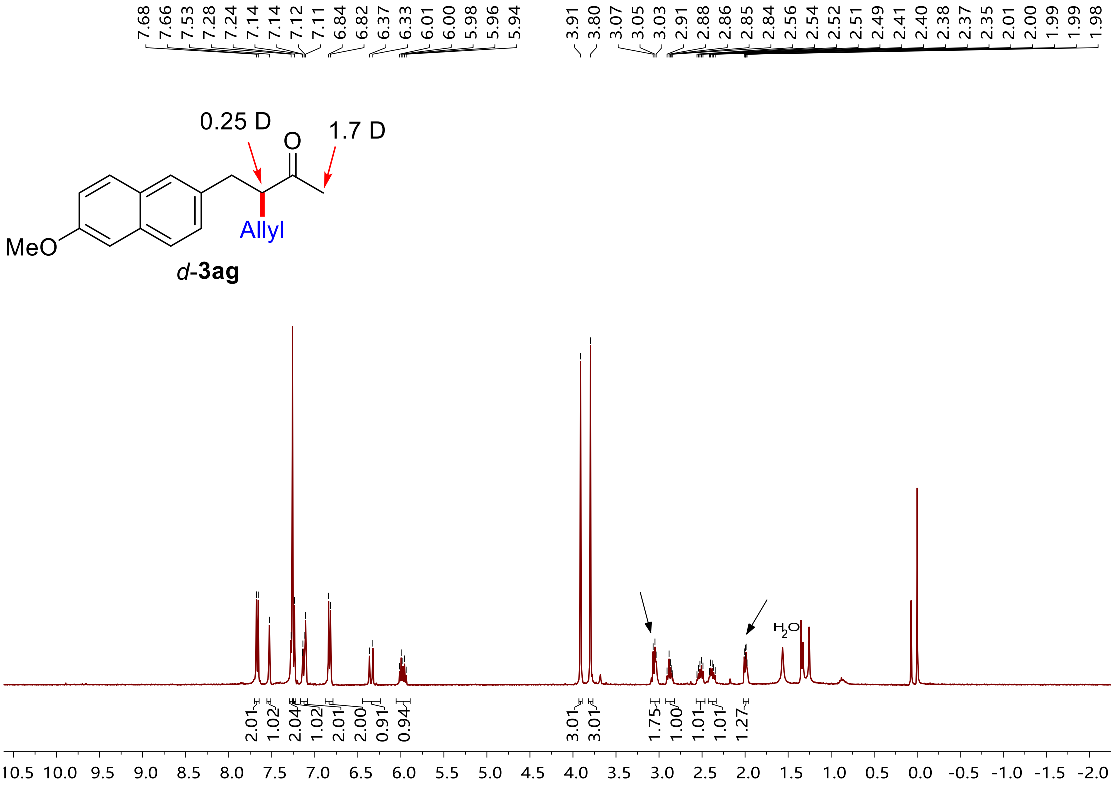


**Supplementary Fig. 4.** $^{1}$H NMR spectrum of *d*-**3ag**.

## (b) Reaction using alcohol

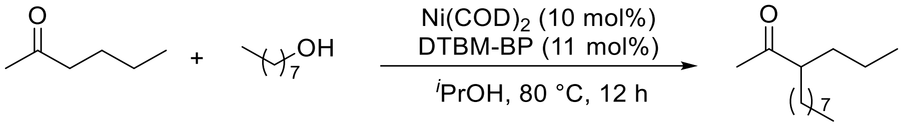


**1m** not obtained

In an argon-filled glove box, an oven-dried tube was charged with a stir bar, catalyst precursor Ni(COD)$_2$ (2.75 mg, 10 mol %), ligand DTBM-BP (**L4**) (12 mg, 11 mol %), ketones **1m** (0.3 mmol) and 1-octanol (0.1 mmol). $^{i}$PrOH (0.5 mL) was injected into the tube under argon and the mixture was stirred at room temperature for 5 min and then heated at 80 °C for 12 h. No reaction occurred.

### (c) Reaction using a 1:1 mixture of *E* and *Z*-allylic alcohol

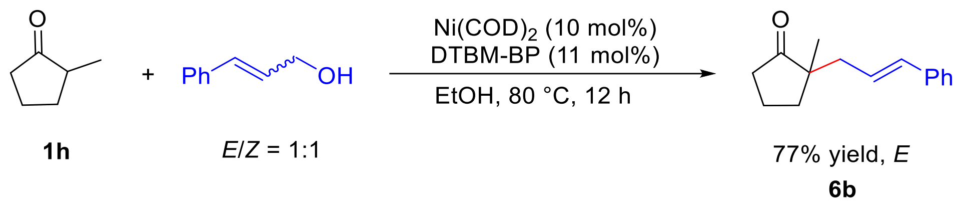


In an argon-filled glove box, an oven-dried tube was charged with a stir bar, catalyst precursor Ni(COD)$_2$ (2.75 mg, 10 mol %), ligand DTBM-BP (**L4**) (12 mg, 11 mol %), ketones **1h** (0.1 mmol) and a mixture of *E* and *Z* 3-phenylprop-2-en-1-ol (*E*/*Z* = 1:1) (0.1 mmol). EtOH (0.5 mL) was injected into the tube under argon and the mixture was stirred at room temperature for 5 min and then heated at 80 °C for 12 h. The *E*-product **6a** was obtained in 77% yield.

### (d) Reaction using 1-phenylpentane-1,4-dione

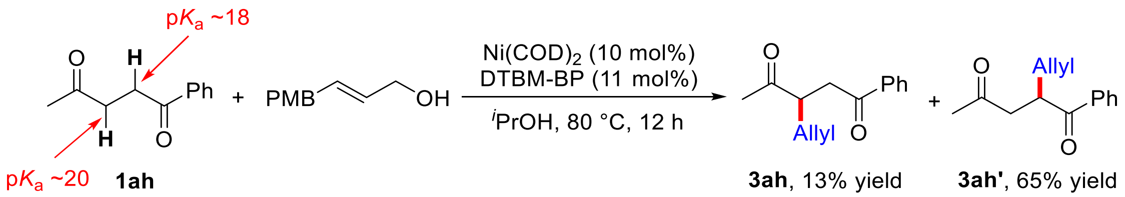


In an argon-filled glove box, an oven-dried tube was charged with a stir bar, catalyst precursor Ni(COD)$_2$ (2.75 mg, 10 mol %), ligand DTBM-BP (**L4**) (12 mg, 11 mol %), ketones **1ah** (0.3 mmol) and allyl alcohol **2a** (0.1 mmol). $^i$PrOH (0.5 mL) was injected into the tube under argon and the mixture was stirred at room temperature for 5 min and then heated at 80 °C for 12 h. After cooling to room temperature, the solvent was removed under a vacuum. The products were obtained by preparative TLC. The regioisomeric ratio of products was determined by NMR.

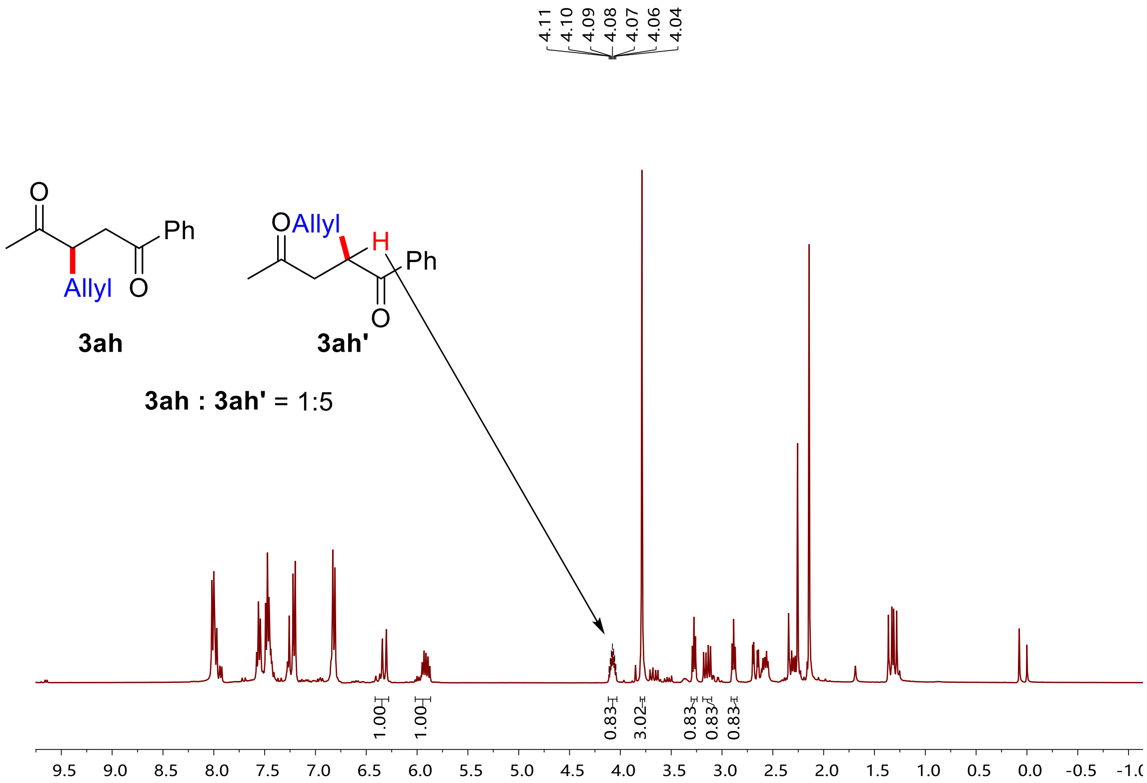


**Supplementary Fig. 5.** $^{1}$H NMR spectrum of **3ah**, **3ah′** (mixture).

## (e) Allylic alkylation using palladium catalysts

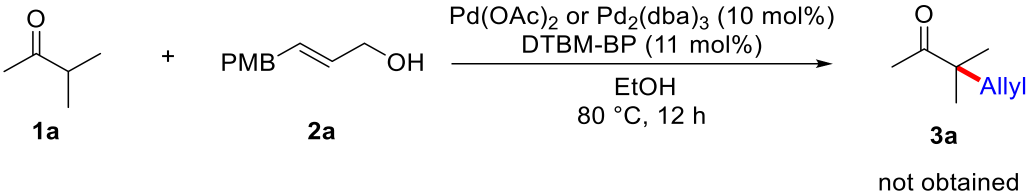


In an argon-filled glove box, an oven-dried tube was charged with a stir bar, catalyst precursor $Pd(OAc)_2$ (2.24 mg, 10 mol %) or $Pd_2(dba)_3$ (9.15 mg, 10 mol %), ligand DTBM-BP (**L4**) (12 mg, 11 mol %), ketones **1** (0.1 mmol) and allyl alcohol **2a** (0.1 mmol). EtOH (0.5 mL) was injected into the tube under argon and the mixture was stirred at room temperature for 5 min and then heated at 80 °C for 12 h. No desired product was observed.

## 2.2 Typical Procedure of Nickel-Catalyzed Allylic Alkylation and Characterization of Products

### (a) General procedure for allylic alkylation

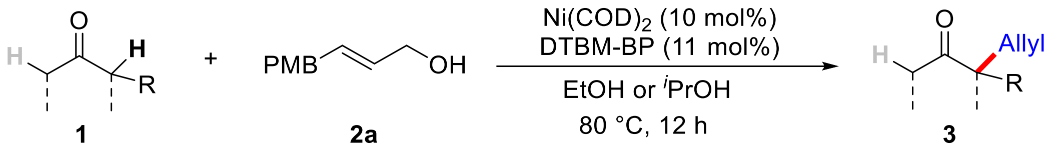


In an argon-filled glove box, an oven-dried tube was charged with a stir bar, catalyst precursor Ni(COD)$_2$ (2.75 mg, 10 mol %), ligand DTBM-BP (**L4**) (12 mg, 11 mol %), ketones **1** (0.1 mmol) and allyl alcohols **2a** (0.1 mmol). EtOH (0.5 mL) was injected into the tube under argon and then the tube was sealed and removed from the glove box. The mixture was stirred at room temperature for 5 min and heated at 80 °C for 12 h. After cooling to room temperature, the solvent was removed under a vacuum. The pure product **3** was obtained by preparative TLC.

### (b) Gram-scale reaction procedure

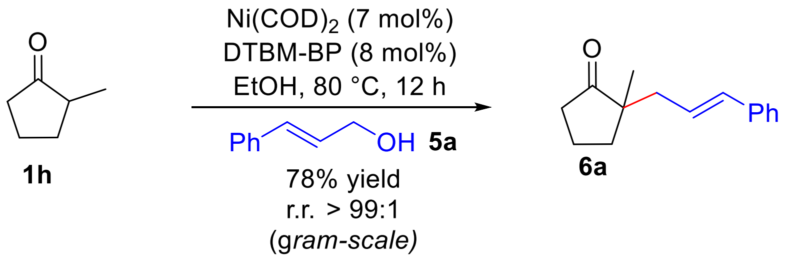


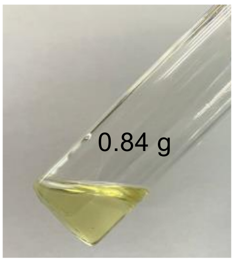


In an argon-filled glove box, an oven-dried tube was charged with a stir bar, catalyst precursor Ni(COD)$_2$ (96 mg, 7 mol %), ligand DTBM-BP (420 mg, 8 mol %), ketones **1h** (5 mmol, 490 mg) and allyl alcohols **5a** (5 mmol, 670 mg). The tube was sealed and removed from the glove box, and EtOH (20 mL) was injected into the tube under argon. The mixture was stirred at room temperature for 5 min and heated at 80 °C for 12 h. After cooling to room temperature, the solvent was removed under a vacuum. then the title compound was purified by column chromatography on silica gel (petroleum ether/ethyl acetate = 20/1) affording 0.84 g **6a** with 78% yield.

**(c) Characterization of products**

**(*E*)-6-(4-methoxyphenyl)-3,3-dimethylhex-5-en-2-one (3a)**

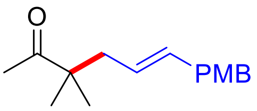


Colorless oil. 18.8 mg; 81% yield. $^{1}$H NMR (400 MHz, $CDCl_3$) δ 7.26 (d, *J* = 8.7 Hz, 2H), 6.83 (d, *J* = 8.7 Hz, 2H), 6.34 (d, *J* = 15.9 Hz, 1H), 5.94 (dt, *J* = 15.4, 7.5 Hz, 1H), 3.79 (s, 3H), 2.38 (dd, J = 7.5, 1.4 Hz, 2H), 2.15 (s, 3H), 1.17 (s, 6H). $^{13}$C NMR (101 MHz, $CDCl_3$) δ 213.6, 159.1, 132.5, 130.3, 127.3, 123.6, 114.0, 55.4, 48.3, 43.2, 32.1, 25.5, 24.4. HRMS (ESI) m/z: $[M+Na]^+$ calcd. for $C_{15}H_{20}NaO_2^+$: 255.1356. Found: 255.1358.

**(*E*)-3-ethyl-6-(4-methoxyphenyl)-3-methylhex-5-en-2-one (3b)**

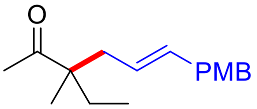


Colorless oil. 14.8 mg; 60% yield. $^{1}$H NMR (400 MHz, $CDCl_3$) δ 7.25 (d, *J* = 8.7 Hz, 2H), 6.83 (d, *J* = 8.7 Hz, 2H), 6.34 (d, *J* = 15.7 Hz, 1H), 5.91 (dt, *J* = 15.4, 7.5 Hz, 1H), 3.79 (s, 3H), 2.50–2.44 (m, 1H), 2.34–2.29 (m, 1H), 2.13 (s, 3H), 1.75–1.65 (m, 1H), 1.59–1.50 (m, 1H), 1.12 (s, 2H), 0.82 (t, *J* = 7.5 Hz, 3H). $^{13}$C NMR (101 MHz, $CDCl_3$) δ 213.5, 159.0, 132.4, 130.3, 127.3, 123.6, 114.0, 55.4, 52.2, 41.2, 31.1, 25.9, 20.7, 8.9. HRMS (ESI) m/z: $[M+Na]^+$ calcd. for $C_{16}H_{22}NaO_2^+$: 269.1512. Found: 269.1517.

**(*E*)-1-(1-(3-(4-methoxyphenyl)allyl)cyclobutyl)ethan-1-one (3c)**

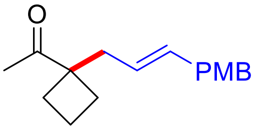


Colorless oil. 12.5 mg; 51% yield. $^{1}$H NMR (400 MHz, $CDCl_3$) δ 7.25 (d, *J* = 10.0 Hz, 2H), 6.82 (d, *J* = 8.6 Hz, 2H), 6.37 (d, *J* = 15.7 Hz, 1H), 5.95–5.83 (m, 1H), 3.79 (s, 3H), 2.65 (d, *J* = 7.2 Hz, 2H), 2.43–2.35 (m, 2H), 2.10 (s, 3H), 1.96–1.89 (m, 3H), 1.83–1.76 (m, 1H). $^{13}$C NMR (101 MHz, $CDCl_3$) δ 211.8, 159.1, 132.2, 130.1, 127.4, 122.8, 114.0, 55.4, 54.1, 40.8, 28.4, 24.7, 14.7. HRMS (ESI) m/z: $[M+Na]^+$ calcd. for $C_{16}H_{20}NaO_2^+$: 267.1356. Found: 267.1360.

**(*E*)-1-(1-(3-(4-methoxyphenyl)allyl)cyclopentyl)ethan-1-one (3d)**

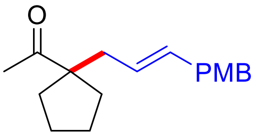


Colorless oil. 17.3 mg; 67% yield. $^{1}$H NMR (400 MHz, $CDCl_3$) δ 7.24 (d, *J* = 8.7 Hz, 2H), 6.82 (d, *J* = 8.6 Hz, 2H), 6.33 (d, *J* = 15.7 Hz, 1H), 5.88 (dt, *J* = 15.3, 7.3 Hz, 1H), 3.79 (s, 3H), 2.49 (d, *J* = 7.3 Hz, 2H), 2.17 (s, 3H), 2.07–2.01 (m, 2H), 1.67–1.52 (m, 6H). $^{13}$C NMR (101 MHz, $CDCl_3$) δ 212.3, 159.1, 132.1, 130.2, 127.4, 124.1, 114.0, 60.3, 55.4, 41.8, 34.4, 26.0, 25.4. HRMS (ESI) m/z: $[M+Na]^+$ calcd. for $C_{17}H_{22}NaO_2^+$: 281.1512. Found: 281.1518.

**(*E*)-1-(1-(3-(4-methoxyphenyl)allyl)cyclohexyl)ethan-1-one (3e)**

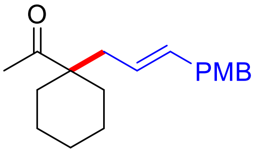


Colorless oil. 16.9 mg; 62% yield. $^{1}H$ NMR (400 MHz, $CDCl_3$) δ 7.24 (d, *J* = 8.7 Hz, 2H), 6.82 (d, *J* = 8.7 Hz, 2H), 6.30 (d, *J* = 15.6 Hz, 1H), 5.86 (dt, *J* = 15.4, 7.5 Hz, 1H), 3.79 (s, 3H), 2.36 (d, *J* = 7.5 Hz, 2H), 2.14 (s, 3H), 2.04–1.98 (m, 2H), 1.61–1.49 (m, 3H), 1.39–1.25 (m, 5H). $^{13}C$ NMR (101 MHz, $CDCl_3$) δ 213.3, 159.1, 132.3, 130.2, 127.4, 122.8, 114.0, 55.4, 52.9, 42.4, 33.3, 26.1, 25.8, 23.1. HRMS (ESI) m/z: $[M+Na]^+$ calcd. for $C_{18}H_{24}NaO_2^+$: 295.1669. Found: 295.1670.

**(*E*)-1-(4-(3-(4-methoxyphenyl)allyl)tetrahydro-2H-pyran-4-yl)ethan-1-one (3f)**

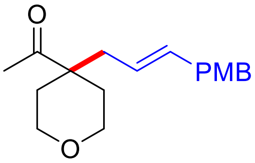


Colorless oil. 19.2 mg; 70% yield. $^{1}H$ NMR (400 MHz, $CDCl_3$) δ 7.24 (d, *J* = 8.7 Hz, 2H), 6.82 (d, *J* = 8.7 Hz, 2H), 6.33 (d, *J* = 15.6 Hz, 1H), 5.82 (dt, *J* = 15.4, 7.5 Hz, 1H), 3.83–3.79 (m, 5H), 3.49–3.43 (m, 2H), 2.44 (d, *J* = 7.4 Hz, 2H), 2.17 (s, 3H), 2.09–2.06 (m, 2H), 1.67–1.60 (m, 2H). $^{13}C$ NMR (101 MHz, $CDCl_3$) δ 211.8, 159.2, 133.1, 129.8, 127.4, 121.5, 114.1, 65.1, 55.4, 50.5, 42.3, 33.1, 25.9. HRMS (ESI) m/z: $[M+Na]^+$ calcd. for $C_{17}H_{22}NaO_3^+$: 297.1461. Found: 297.1463.

**(*E*)-1-(1-(3-(4-methoxyphenyl)allyl)cycloheptyl)ethan-1-one (3g)**

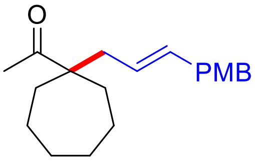


Colorless oil. 16.3 mg; 57% yield. $^{1}H$ NMR (400 MHz, $CDCl_3$) δ 7.23 (d, *J* = 8.6 Hz, 2H), 6.82 (d, *J* = 8.7 Hz, 2H), 6.32 (d, *J* = 15.7 Hz, 1H), 5.84 (dt, *J* = 15.3, 7.4 Hz, 1H), 3.79 (s, 3H), 2.39 (d, *J* = 7.6 Hz, 2H), 2.09–2.03 (m, 2H), 1.60–1.41 (m, 10H). $^{13}C$ NMR (101 MHz, $CDCl_3$) δ 213.3, 159.1, 132.3, 130.2, 127.4, 123.4, 114.0, 55.8, 55.4, 43.7, 35.2, 30.4, 25.9, 23.7. HRMS (ESI) m/z: $[M+Na]^+$ calcd. for $C_{19}H_{26}NaO_2^+$: 309.1825. Found: 309.1830.

**(*E*)-2-(3-(4-methoxyphenyl)allyl)-2-methylcyclopentan-1-one (3h)**

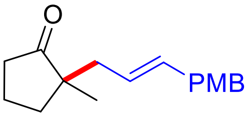


Colorless oil. 19.5 mg; 80% yield. $^{1}H$ NMR (400 MHz, $CDCl_3$) δ 7.27 (d, *J* = 8.6 Hz, 2H), 6.84 (d, *J* = 8.5 Hz, 2H), 6.35 (d, *J* = 15.7 Hz, 1H), 5.96 (dt, *J* = 15.5, 7.6 Hz, 1H), 3.80 (s, 3H), 2.36–2.18 (m, 4H), 2.04–1.98 (m, 1H), 1.92–1.83 (m, 2H), 1.75–1.69 (m, 1H), 1.05 (s, 3H). $^{13}C$ NMR (101 MHz, $CDCl_3$) δ 223.3, 159.1, 132.8, 130.3, 127.3, 123.4, 114.1, 55.4, 48.9, 40.3, 37.9, 35.3, 22.2, 18.9. HRMS (ESI) m/z: $[M+Na]^+$ calcd. for $C_{16}H_{20}NaO_2^+$: 267.1356 Found: 267.1359.

**(*E*)-2-(3-(4-methoxyphenyl)allyl)-2-methylcyclohexan-1-one (3i)**

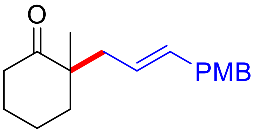


Colorless oil. 19.6 mg; 76% yield. $^{1}H$ NMR (400 MHz, $CDCl_3$) δ 7.26 (d, *J* = 8.7 Hz, 2H), 6.83 (d, *J* = 8.7 Hz, 2H), 6.33 (d, *J* = 15.7 Hz, 1H), 5.97 (dt, *J* = 15.4, 7.5 Hz, 1H), 3.79 (s, 3H), 2.46–2.38 (m, 4H), 1.88–1.73 (m, 5H), 1.65–1.58 (m, 1H), 1.12 (s, 3H).

$^{13}$C NMR (101 MHz, $CDCl_3$) δ 215.5, 159.0, 132.5, 130.4, 127.3, 123.5, 114.0, 55.4, 49.1, 41.3, 39.0, 38.7, 27.5, 23.0, 21.3. HRMS (ESI) m/z: $[M+Na]^+$ calcd. for $C_{17}H_{22}NaO_2^+$: 281.1512. Found: 281.1516.

**(*E*)-2-(3-(4-methoxyphenyl)allyl)-2-propylcyclohexan-1-one (3j)**

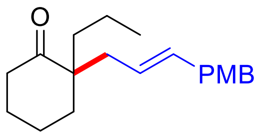


Colorless oil. 19.5 mg; 68% yield. $^1$H NMR (400 MHz, $CDCl_3$) δ 7.26 (d, *J* = 8.7 Hz, 2H), 6.83 (d, *J* = 8.7 Hz, 2H), 6.31 (d, *J* = 15.7 Hz, 1H), 5.95 (dt, *J* = 15.4, 7.5 Hz, 1H), 3.79 (s, 3H), 2.46–2.32 (m, 4H), 1.93–1.87 (m, 1H), 1.81–1.66 (m, 6H), 1.49–1.41 (m, 1H), 1.38–1.29 (m, 1H), 1.11–1.01 (m, 1H), 0.89 (t, *J* = 7.2 Hz, 3H). $^{13}$C NMR (101 MHz, $CDCl_3$) δ 215.3, 158.9, 132.2, 130.6, 127.3, 124.1, 114.0, 55.4, 52.3, 39.4, 38.5, 37.6, 36.7, 27.3, 21.0, 16.9, 14.9. HRMS (ESI) m/z: $[M+Na]^+$ calcd. for $C_{19}H_{26}NaO_2^+$: 309.1825 Found: 309.1831.

**(*E*)-3-(3-(4-methoxyphenyl)allyl)-1,3-dimethylpiperidin-4-one (3k)**

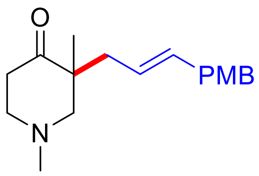


Colorless oil. 20.2 mg; 74% yield. $^1$H NMR (400 MHz, $CDCl_3$) δ 7.26 (d, *J* = 8.7 Hz, 2H), 6.83 (d, *J* = 8.7 Hz, 2H), 6.36 (d, *J* = 15.7 Hz, 1H), 5.94 (dt, *J* = 15.4, 7.6 Hz, 1H), 3.79 (s, 3H), 2.80–2.75 (m, 1H), 2.65–2.54 (m, 4H), 2.49–2.43 (m, 2H), 2.33 (s, 3H), 2.29–2.26 (m, 1H), 1.11 (s, 3H). $^{13}$C NMR (101 MHz, $CDCl_3$) δ 212.8, 159.0, 132.7, 130.3, 127.4, 122.9, 114.0, 66.5, 56.3, 55.4, 49.4, 46.0, 40.4, 38.8, 20.9. HRMS (ESI) m/z: $[M+H]^+$ calcd. for $C_{17}H_{24}NO_2^+$: 274.1802 Found: 274.1805.

**(*E*)-7-(4-methoxyphenyl)-4,4-dimethylhept-6-en-3-one (3l)**

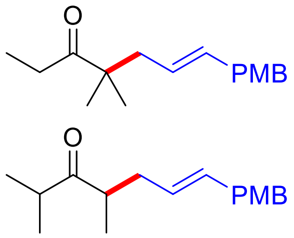


r.r = 2.3:1

Colorless oil. Isomeric mixture; r.r = 2.3:1. 16.2 mg; 66% yield. $^1$H NMR (400 MHz, $CDCl_3$) δ 7.25 (d, *J* = 8.7 Hz, 2H), 6.83 (d, *J* = 8.7 Hz, 2H), 6.37–6.28 (m, 1H), 6.00–5.89 (m, 1H), 3.79 (s, 3H), 2.87–2.79 (m, 0.3H), 2.77–2.70 (m, 0.3H), 2.54–2.45 (m, 1.7H), 2.38 (d, *J* = 8.8 Hz, 1.4H), 2.23–2.16 (m, 0.3H), 1.17 (s, 4H), 1.11–1.01 (m, 5H). $^{13}$C NMR (101 MHz, $CDCl_3$) δ 217.9, 216.0, 159.0, 132.4, 131.4, 130.3, 127.3, 127.2, 125.5, 123.9, 114.0, 55.4, 48.1, 44.8, 43.4, 40.1, 36.8, 30.5, 24.5, 18.4, 18.3, 16.8, 8.2. HRMS (ESI) m/z: $[M+Na]^+$ calcd. for $C_{16}H_{22}NaO_2^+$: 269.1512 Found: 269.1516.

**(*E*)-6-(4-methoxyphenyl)-3-propylhex-5-en-2-one (3m)**

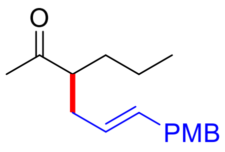


Colorless oil. 17.7 mg; 72% yield. $^1$H NMR (400 MHz, $CDCl_3$) δ 7.25 (d, *J* = 7.6 Hz, 2H), 6.83 (d, *J* = 7.7 Hz, 2H), 6.34 (d, *J* = 15.8 Hz, 1H), 3.79 (s, 3H), 2.66–2.59 (m, 1H), 2.49–2.42 (m, 1H), 2.34–2.27 (m, 1H), 2.14 (s, 3H), 1.65–1.58 (m, 1H), 1.50–1.41 (m, 1H), 1.34–1.25 (m, 2H), 0.91 (t, *J* = 7.1 Hz, 3H). $^{13}$C NMR (101 MHz, $CDCl_3$) δ 212.4,

159.0, 131.4, 130.3, 127.3, 125.2, 114.1, 55.4, 53.1, 35.1, 33.6, 29.5, 20.7, 14.3. HRMS (ESI) m/z: $[M+Na]^+$ calcd. for $C_{16}H_{22}NaO_2^+$: 269.1512. Found: 269.1514.

**(*E*)-6-(4-methoxyphenyl)-3-methylhex-5-en-2-one (3n)**

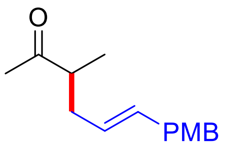


Colorless oil. 14.1 mg; 65% yield. $^1H$ NMR (400 MHz, $CDCl_3$) δ 7.26 (d, $J$ = 8.5 Hz, 2H), 6.83 (d, $J$ = 8.3 Hz, 2H), 6.35 (d, $J$ = 15.7 Hz, 1H), 5.98 (dt, $J$ = 15.2, 7.2 Hz, 1H), 3.80 (s, 3H), 2.68–2.63 (m, 1H), 2.56–2.50 (m, 1H), 2.27–2.20 (m, 1H), 2.16 (s, 3H), 1.14 (d, $J$ = 7.0 Hz, 3H). $^{13}C$ NMR (101 MHz, $CDCl_3$) δ 212.2, 159.0, 131.6, 130.3, 127.3, 125.2, 114.1, 55.4, 47.4, 36.3, 28.6, 16.2. HRMS (ESI) m/z: $[M+Na]^+$ calcd. for $C_{14}H_{18}NaO_2^+$: 241.1199. Found: 241.1203.

**(*E*)-3-ethyl-6-(4-methoxyphenyl)hex-5-en-2-one (3o)**

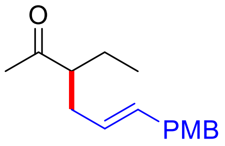


Colorless oil. 14.2 mg; 61% yield. $^1H$ NMR (400 MHz, $CDCl_3$) δ 7.25 (d, $J$ = 8.5 Hz, 2H), 6.83 (d, $J$ = 8.7 Hz, 2H), 6.34 (d, $J$ = 15.7 Hz, 1H), 5.96 (dt, $J$ = 15.6, 7.2 Hz, 1H), 3.79 (s, 3H), 2.59–2.52 (m, 1H), 2.50– 2.42 (m, 1H), 2.35–2.28 (m, 1H), 2.14 (s, 3H), 1.71–1.64 (m, 1H), 1.60– 1.52 (m, 1H), 0.90 (t, $J$ = 7.4 Hz, 3H). $^{13}C$ NMR (101 MHz, $CDCl_3$) δ 212.3, 159.0, 131.4, 130.3, 127.3, 125.2, 114.1, 55.4, 54.7, 34.6, 29.6, 24.3, 11.8. HRMS (ESI) m/z: $[M+Na]^+$ calcd. for $C_{15}H_{20}NaO_2^+$: 255.1356. Found: 255.1360.

**(*E*)-3-(3-(4-methoxyphenyl)allyl)octan-2-one (3p)**

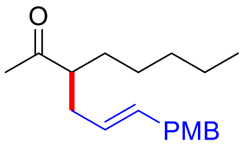


Colorless oil. 19.2 mg; 70% yield. $^1H$ NMR (400 MHz, $CDCl_3$) δ 7.25 (d, $J$ = 7.9 Hz, 2H), 6.83 (d, $J$ = 8.3 Hz, 2H), 6.34 (d, $J$ = 15.7 Hz, 1H), 5.95 (dt, $J$ = 15.2, 7.2 Hz, 1H), 3.80 (s, 3H), 2.64–2.57 (m, 1H), 2.49–2.41 (m, 1H), 2.34–2.27 (m, 1H), 2.14 (s, 3H), 1.66–1.61 (m, 1H), 1.49–1.44 (m, 1H), 1.30–1.24 (m, 6H), 0.87 (t, $J$ = 6.6 Hz, 3H). $^{13}C$ NMR (101 MHz, $CDCl_3$) δ 212.5, 159.0, 131.4, 130.2, 127.3, 125.2, 114.0, 55.4, 53.3, 35.1, 32.0, 31.4, 29.5, 27.1, 22.6, 14.2. HRMS (ESI) m/z: $[M+Na]^+$ calcd. for $C_{18}H_{26}NaO_2^+$: 297.1825. Found: 297.1830.

**(*E*)-3-isopropyl-6-(4-methoxyphenyl)hex-5-en-2-one (3q)**

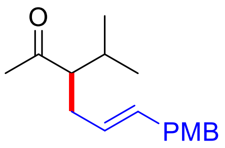


Colorless oil. 12.8 mg; 52% yield. $^1H$ NMR (400 MHz, $CDCl_3$) δ 7.24 (d, $J$ = 8.7 Hz, 2H), 6.82 (d, $J$ = 8.7 Hz, 2H), 6.33 (d, $J$ = 15.7 Hz, 1H), 5.98–5.90 (m, 1H), 3.79 (s, 3H), 2.45–2.35 (m, 3H), 2.12 (s, 3H), 1.99–1.90 (m, 1H), 0.95 (dd, $J$ = 6.8, 4.9 Hz, 6H). $^{13}C$ NMR (101 MHz, $CDCl_3$) δ 212.5, 159.0, 131.1, 130.3, 127.3, 125.6, 114.0, 60.1, 55.4, 32.4, 31.1, 30.0, 21.2, 20.0. HRMS (ESI) m/z: $[M+Na]^+$ calcd. for $C_{16}H_{22}NaO_2^+$: 269.1512. Found: 269.1516.

**(*E*)-3-isobutyl-6-(4-methoxyphenyl)hex-5-en-2-one (3r)**

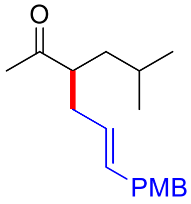


Colorless oil. 17.7 mg; 68% yield. $^{1}H$ NMR (400 MHz, $CDCl_3$) δ 7.25 (d, $J$ = 7.0 Hz, 2H), 6.83 (d, $J$ = 8.7 Hz, 2H), 6.33 (d, $J$ = 15.7 Hz, 1H), 5.95 (dt, $J$ = 15.7, 7.2 Hz, 1H), 3.79 (s, 3H), 2.73–2.66 (m, 1H), 2.46–2.39 (m, 1H), 2.33–2.26 (m, 1H), 2.14 (s, 3H), 1.61–1.52 (m, 2H), 1.32–1.24 (m, 1H), 0.90 (dd, $J$ = 6.3, 4.1 Hz, 6H). $^{13}C$ NMR (101 MHz, $CDCl_3$) δ 212.5, 159.1, 131.4, 130.2, 127.3, 125.1, 114.1, 55.4, 51.3, 40.7, 35.7, 29.4, 26.2, 23.1, 22.5. HRMS (ESI) m/z: $[M+Na]^+$ calcd. for $C_{17}H_{24}NaO_2^+$: 283.1669. Found: 283.1673.

**(*E*)-3-(2-(diethylamino)ethyl)-6-(4-methoxyphenyl)hex-5-en-2-one (3s)**

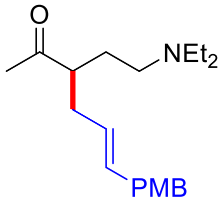


Colorless oil. 19.1 mg; 63% yield. $^{1}H$ NMR (400 MHz, $CDCl_3$) δ 7.25 (d, $J$ = 9.2 Hz, 2H), 6.83 (d, $J$ = 8.8 Hz, 2H), 6.34 (d, $J$ = 15.7 Hz, 1H), 6.00–5.92 (m, 1H), 3.80 (s, 3H), 2.70–2.63 (m, 1H), 2.51–2.44 (m, 5H), 2.39–2.27 (m, 3H), 2.17 (s, 3H), 1.91–1.81 (m, 1H), 1.62–1.54 (m, 1H), 0.98 (t, $J$ = 7.1 Hz, 6H). $^{13}C$ NMR (101 MHz, $CDCl_3$) δ 211.8, 159.0, 131.6, 130.2, 127.3, 125.00, 114.0, 55.4, 51.4, 50.9, 46.7, 35.5, 30.0, 28.9, 11.7. HRMS (ESI) m/z: $[M+H]^+$ calcd. for $C_{19}H_{30}NO_2^+$: 304.2271. Found: 304.2274.

**(*E*)-3-(3-chloropropyl)-6-(4-methoxyphenyl)hex-5-en-2-one (3t)**

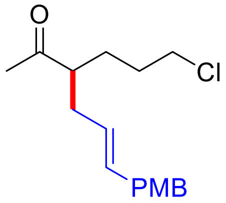


Colorless oil. 17.9 mg; 64% yield. $^{1}H$ NMR (400 MHz, $CDCl_3$) δ 7.26 (d, $J$ = 8.8 Hz, 2H), 6.83 (d, $J$ = 8.7 Hz, 2H), 6.35 (d, $J$ = 15.7 Hz, 1H), 5.94 (dt, $J$ = 15.2, 7.2 Hz, 1H), 3.80 (s, 3H), 3.52 (t, $J$ = 5.8 Hz, 2H), 2.68–2.61 (m, 1H), 2.52–2.45 (m, 1H), 2.37–2.30 (m, 1H), 2.17 (s, 3H), 1.82–1.64 (m, 4H). $^{13}C$ NMR (101 MHz, $CDCl_3$) δ 211.5, 159.1, 131.9, 130.0, 127.4, 124.5, 114.1, 55.4, 52.5, 44.9, 35.1, 30.3, 29.5, 28.2. HRMS (ESI) m/z: $[M+Na]^+$ calcd. for $C_{16}H_{21}ClNaO_2^+$: 303.1122. Found: 303.1128.

**(*E*)-3-(2-(benzyloxy)ethyl)-6-(4-methoxyphenyl)hex-5-en-2-one (3u)**

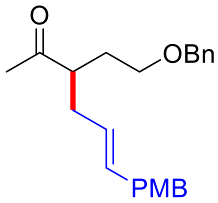


Colorless oil. 28.1 mg; 83% yield. $^{1}H$ NMR (400 MHz, $CDCl_3$) δ 7.36–7.25 (m, 5H), 7.24 (d, $J$ = 9.6 Hz, 2H), 6.82 (d, $J$ = 8.7 Hz, 2H), 6.33 (d, $J$ = 15.7 Hz, 1H), 5.98–5.91 (m, 1H), 4.45 (s, 2H), 3.79 (s, 3H), 3.50– 3.43(m, 2H), 2.86–2.79 (m, 1H), 2.50–2.43 (m, 1H), 2.34–2.28 (m, 1H), 2.13 (s, 3H), 2.03–1.97 (m, 1H), 1.80–1.74 (m, 1H). $^{13}C$ NMR (101 MHz, $CDCl_3$) δ 212.0, 159.1, 138.4, 131.7, 130.1, 128.5, 127.8, 127.7, 127.3, 124.8, 114.1, 73.1, 68.2, 55.4, 50.0, 35.4, 31.3, 30.0. HRMS (ESI) m/z: $[M+Na]^+$ calcd. for $C_{22}H_{26}NaO_3^+$: 361.1774. Found: 361.1780.

**(*E*)-3-allyl-6-(4-methoxyphenyl)hex-5-en-2-one (3v)**

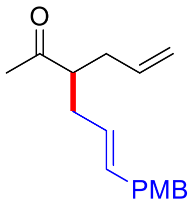


Colorless oil. 19.5 mg; 80% yield. $^1$H NMR (400 MHz, $CDCl_3$) δ 7.26 (d, *J* = 8.7 Hz, 2H), 6.83 (d, *J* = 8.7 Hz, 2H), 6.35 (d, *J* = 15.7 Hz, 1H), 5.95 (dt, *J* = 15.3, 7.3 Hz, 1H), 5.79–5.68 (m, 1H), 5.08–5.03 (m, 2H), 3.80 (s, 3H), 2.74–2.67 (m, 1H), 2.52–2.44 (m, 1H), 2.40–2.32 (m, 2H), 2.29–2.22 (m, 1H), 2.15 (s, 3H). $^{13}$C NMR (101 MHz, $CDCl_3$) δ 211.4, 159.1, 135.5, 131.8, 130.2, 127.3, 124.8, 117.2, 114.1, 55.4, 52.7, 35.4, 34.6, 29.9. HRMS (ESI) m/z: $[M+Na]^+$ calcd. for $C_{16}H_{20}NaO_2^+$: 267.1356. Found: 267.1358.

**(*E*)-4-acetyl-7-(4-methoxyphenyl)-N,N-dimethylhept-6-enamide (3w)**

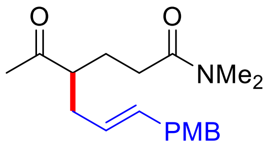


Colorless oil. 20.9 mg; 69% yield. $^1$H NMR (400 MHz, $CDCl_3$) δ 7.24 (d, *J* = 8.6 Hz, 2H), 6.82 (d, *J* = 8.6 Hz, 2H), 6.35 (d, *J* = 15.7 Hz, 1H), 5.96 (dt, *J* = 15.2, 7.2 Hz, 1H), 3.79 (s, 3H), 2.97 (s, 3H), 2.92 (s, 3H), 2.78–2.71 (m, 1H), 2.52–2.47 (m, 1H), 2.38–2.31 (m, 2H), 2.27–2.21 (m, 1H), 2.17 (s, 3H), 2.01–1.94 (m, 1H), 1.89–1.82 (m, 1H). $^{13}$C NMR (101 MHz, $CDCl_3$) δ 211.9, 172.3, 159.1, 131.7, 130.1, 127.3, 124.6, 114.0, 55.4, 52.3, 37.3, 35.5, 35.2, 30.8, 29.4, 26.2. HRMS (ESI) m/z: $[M+Na]^+$ calcd. for $C_{18}H_{25}NNaO_3^+$: 326.1727. Found: 326.1730.

**(*E*)-6-(4-methoxyphenyl)-3-(pyridin-3-ylmethyl)hex-5-en-2-one (3x)**

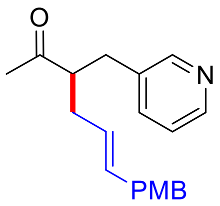


Colorless oil. 20.1 mg; 68% yield. $^1$H NMR (400 MHz, $CDCl_3$) δ 8.46–8.45 (m, 2H), 7.48 (d, *J* = 7.7 Hz, 1H), 7.26 (d, *J* = 8.1 Hz, 2H), 7.20 (dd, *J* = 7.6, 4.9 Hz, 1H), 6.84 (d, *J* = 8.4 Hz, 2H), 6.36 (d, *J* = 15.7 Hz, 1H), 5.95 (dt, *J* = 15.2, 7.3 Hz, 1H), 3.80 (s, 3H), 2.97–2.93 (m, 2H), 2.77–2.70 (m, 1H), 2.54–2.47 (m, 1H), 2.41–2.34 (m, 1H), 2.06 (s, 3H). $^{13}$C NMR (101 MHz, $CDCl_3$) δ 210.7, 159.2, 150.4, 148.0, 136.6, 135.1, 132.4, 129.9, 127.4, 123.8, 123.5, 114.1, 55.4, 54.3, 35.1, 34.1, 30.7. HRMS (ESI) m/z: $[M+H]^+$ calcd. for $C_{19}H_{22}NO_2^+$: 296.1645. Found: 296.1646.

**(*E*)-6-(4-methoxyphenyl)-3-(2-(thiophen-2-yl)ethyl)hex-5-en-2-one (3y)**

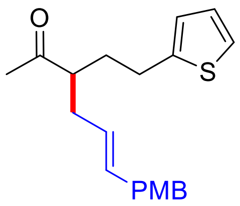


Colorless oil. 20.7 mg; 66% yield. $^1$H NMR (400 MHz, $CDCl_3$) δ 7.25 (d, *J* = 8.7 Hz, 2H), 7.12 (dd, *J* = 5.1, 1.2 Hz, 1H), 6.92–6.90 (m, 1H), 6.83 (d, *J* = 8.7 Hz, 2H), 6.78–6.77 (m, 1H), 6.34 (d, *J* = 15.8 Hz, 1H), 5.97–5.90 (m, 1H), 3.79 (s, 3H), 2.87–2.77 (m, 2H), 2.72–2.66 (m, 1H), 2.52–2.45 (m, 1H), 2.39–2.32 (m, 1H), 2.15 (s, 3H), 2.09–2.04 (m, 1H), 1.87–1.80 (m, 1H). $^{13}$C NMR (101 MHz, $CDCl_3$) δ 211.6, 159.1, 144.4, 131.8, 130.1, 127.3, 126.9, 124.6, 124.5, 123.4, 114.1, 55.4, 52.0, 35.1, 32.9, 29.8, 27.6. HRMS (ESI) m/z: $[M+Na]^+$ calcd. for $C_{19}H_{22}NaO_2S^+$: 337.1233. Found: 337.1238.

**(*E*)-3-(furan-2-ylmethyl)-6-(4-methoxyphenyl)hex-5-en-2-one (3z)**

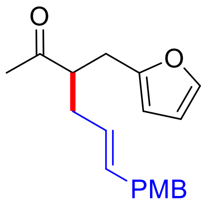


Colorless oil. 15.3 mg; 54% yield. $^{1}$H NMR (400 MHz, $CDCl_3$) δ 7.31–7.30 (m, 1H), 7.25 (d, *J* = 8.7 Hz, 2H), 6.83 (d, *J* = 8.7 Hz, 2H), 6.35 (d, *J* = 15.7 Hz, 1H), 6.27 (d, *J* = 1.9 Hz, 1H), 6.01 (d, *J* = 2.9 Hz, 1H), 5.94 (dd, *J* = 15.7, 7.3 Hz, 1H), 3.80 (s, 3H), 3.05–2.93 (m, 2H), 2.83–2.78 (m, 1H), 2.53–2.46 (m, 1H), 2.39–2.32 (m, 1H), 2.11 (s, 3H). $^{13}$C NMR (101 MHz, $CDCl_3$) δ 211.0, 159.0, 153.1, 141.4, 132.0, 129.9, 127.2, 124.1, 113.9, 110.3, 106.5, 55.3, 51.5, 34.7, 30.0, 29.4. HRMS (ESI) m/z: $[M+Na]^+$ calcd. for $C_{18}H_{20}NaO_3^+$: 307.1305. Found: 307.1310.

**2-cinnamyl-2-methylcyclopentan-1-one (6a)**[6]

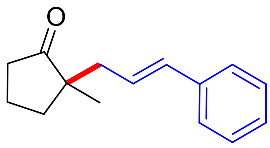


Colorless oil. 16.3 mg; 76% yield. $^{1}$H NMR (400 MHz, $CDCl_3$) δ 7.35–7.28 (m, 4H), 7.23–7.19 (m, 1H), 6.41 (d, *J* = 15.7 Hz, 1H), 6.15–6.07 (m, 1H), 2.36–2.19 (m, 4H), 2.04–1.98 (m, 1H), 1.93–1.85 (m, 2H), 1.76–1.70 (m, 1H), 1.06 (s, 3H). $^{13}$C NMR (101 MHz, $CDCl_3$) δ 223.3, 137.5, 133.4, 128.6, 127.3, 126.2, 125.7, 48.9, 40.4, 37.9, 35.3, 22.2, 18.9.

**(*E*)-2-(3-(2-methoxyphenyl)allyl)-2-methylcyclopentan-1-one (6c)**

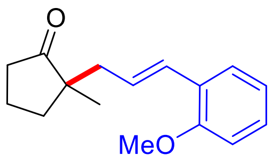


Colorless oil. 19.1 mg; 78% yield. $^{1}$H NMR (400 MHz, $CDCl_3$) δ 7.40 (d, *J* = 7.5 Hz, 1H), 7.19 (t, *J* = 7.7 Hz, 1H), 6.92–6.84 (m, 2H), 6.73 (d, *J* = 15.9 Hz, 1H), 6.10 (dt, *J* = 15.5, 7.6 Hz, 1H), 3.83 (s, 3H), 2.37–2.17 (m, 4H), 2.06–2.00 (m, 1H), 1.93–1.83 (m, 2H), 1.75–1.69 (m, 1H), 1.06 (s, 3H). $^{13}$C NMR (101 MHz, $CDCl_3$) δ 223.4, 156.5, 128.3, 128.0, 126.7, 126.5, 126.3, 120.7, 111.0, 55.6, 48.9, 40.8, 37.9, 35.3, 22.2, 18.9. HRMS (ESI) m/z: $[M+Na]^+$ calcd. for $C_{16}H_{20}NaO_2^+$: 267.1356. Found: 267.1360.

**(*E*)-2-(3-(3-methoxyphenyl)allyl)-2-methylcyclopentan-1-one (6d)**

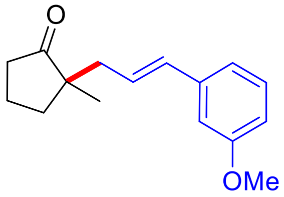


Colorless oil. 19.5 mg; 80% yield. $^{1}$H NMR (400 MHz, $CDCl_3$) δ 7.21 (t, *J* = 7.9 Hz, 1H), 6.94 (d, *J* = 7.7 Hz, 1H), 6.88–6.87 (m, 1H), 6.77 (dd, *J* = 8.1, 2.4 Hz, 1H), 6.38 (d, *J* = 15.7 Hz, 1H), 6.10 (dt, *J* = 15.5, 7.6 Hz, 1H), 3.81 (s, 3H), 2.36–2.16 (m, 4H), 2.04–1.97 (m, 1H), 1.93–1.85 (m, 2H), 1.76–1.70 (m, 1H), 1.06 (s, 3H). $^{13}$C NMR (101 MHz, $CDCl_3$) δ 223.3, 159.9, 138.9, 133.3, 129.6, 126.1, 118.9, 112.9, 111.7, 55.4, 48.9, 40.3, 37.9, 35.3, 22.3, 18.9. HRMS (ESI) m/z: $[M+Na]^+$ calcd. for $C_{16}H_{20}NaO_2^+$: 267.1356. Found: 267.1360.

**(*E*)-2-methyl-2-(3-(p-tolyl)allyl)cyclopentan-1-one (6e)**

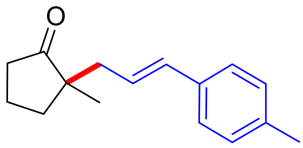

Colorless oil. 16.9 mg; 74% yield. $^{1}$H NMR (400 MHz, $CDCl_3$) δ 7.23 (d, *J* = 8.1 Hz, 2H), 7.10 (d, *J* = 7.9 Hz, 2H), 6.37 (d, *J* = 15.7 Hz, 1H), 6.05 (dt, *J* = 15.4, 7.6 Hz, 1H), 2.34–2.16 (m, 7H), 2.04–1.97 (m, 1H), 1.92–1.84 (m, 2H), 1.75–1.69 (m, 1H), 1.05 (s, 3H). $^{13}$C NMR (101 MHz, $CDCl_3$) δ 223.3, 137.1, 134.7, 133.3, 129.3, 126.1, 124.7, 48.9, 40.4, 37.9, 35.3, 22.3, 21.3, 18.9. HRMS (ESI) m/z: $[M+Na]^+$ calcd. for $C_{16}H_{20}NaO^+$: 251.1406. Found: 251.1410.

**(*E*)-2-(3-(4-fluorophenyl)allyl)-2-methylcyclopentan-1-one (6f)**[6]

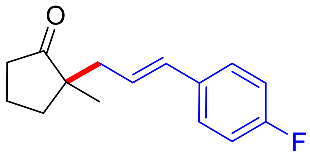

Colorless oil. 15.3 mg; 66% yield. $^{1}$H NMR (400 MHz, $CDCl_3$) δ 7.31–7.28 (m, 2H), 7.00–6.96 (m, 2H), 6.37 (d, *J* = 15.7 Hz, 1H), 6.02 (dt, *J* = 15.4, 7.6 Hz, 1H), 2.37–2.16 (m, 4H), 2.03–1.96 (m, 1H), 1.93–1.85 (m, 2H), 1.76–1.70 (m, 1H), 1.05 (s, 3H). $^{13}$C NMR (101 MHz, $CDCl_3$) δ 223.3, 137.1, 134.7, 133.3, 129.3, 126.1, 124.7, 48.9, 40.4, 37.9, 35.3, 22.3, 21.3, 18.9. $^{19}$F NMR (376 MHz, $CDCl_3$) δ –115.1. $^{13}$C NMR (101 MHz, $CDCl_3$) δ 223.1, 162.2 (d, $J_{CF}$ = 242.4 Hz), 133.6, 132.2, 127.7 (d, $J_{CF}$ = 10.1 Hz), 125.5, 115.5 (d, $J_{CF}$ = 20.2 Hz), 48.9, 40.3, 37.8, 35.4, 22.2, 18.9.

**(*E*)-2-methyl-2-(oct-2-en-1-yl)cyclopentan-1-one (6h)**

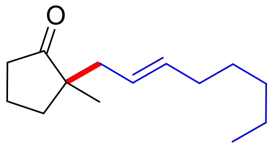

Colorless oil. 11.1 mg; 53% yield. $^{1}$H NMR (400 MHz, $CDCl_3$) δ 5.43 (dt, *J* = 14.1, 6.6 Hz, 1H), 5.26 (dt, *J* = 15.1, 7.3 Hz, 1H), 2.28–1.92 (m, 7H), 1.88–1.80 (m, 2H), 1.66–1.60 (m, 1H), 1.34–1.22 (m, 6H), 0.96 (s, 3H), 0.86 (t, *J* = 6.8 Hz, 3H). $^{13}$C NMR (101 MHz, $CDCl_3$) δ 223.7, 134.8, 125.0, 48.7, 39.9, 38.0, 35.2, 32.7, 31.5, 29.3, 22.6, 22.1, 18.8, 14.2. HRMS (ESI) m/z: $[M+Na]^+$ calcd. for $C_{14}H_{24}NaO^+$: 231.1719. Found: 231.1725.

**(*E*)-2-(hept-2-en-1-yl)-2-methylcyclopentan-1-one (6i)**

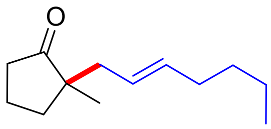

Colorless oil. 9.9 mg; 51% yield. $^{1}$H NMR (400 MHz, $CDCl_3$) δ 5.45 (dt, *J* = 13.7, 6.7 Hz, 1H), 5.29 (dt, *J* = 15.0, 7.3 Hz, 1H), 2.31–1.93 (m, 7H), 1.90–1.82 (m, 2H), 1.69–1.63 (m, 1H), 1.34–1.27 (m, 4H), 0.99 (s, 3H), 0.89 (t, *J* = 7.0 Hz, 3H). $^{13}$C NMR (101 MHz, $CDCl_3$) δ 223.7, 134.7, 125.0, 48.7, 39.9, 37.9, 35.2, 32.4, 31.8, 22.3, 22.1, 18.8, 14.1. HRMS (ESI) m/z: $[M+Na]^+$ calcd. for $C_{13}H_{22}NaO^+$: 217.1563. Found: 217.1568.

**2-((2*E*,4*E*)-5,9-dimethyldeca-2,4,8-trien-1-yl)-2-methylcyclopentan-1-one (6j)**

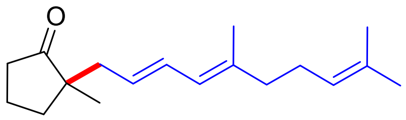

Colorless oil. 16.9 mg; 65% yield. $^1$H NMR (600 MHz, $CDCl_3$) δ 6.26 (dd, *J* = 15.0, 10.8 Hz, 1H), 5.80 (d, *J* = 10.8 Hz, 1H), 5.45 (dt, *J* = 15.2, 7.6 Hz, 1H), 5.10 (t, *J* = 7.0 Hz, 1H), 2.32–2.26 (m, 1H), 2.22–2.02 (m, 7H), 1.99–1.93 (m, 1H), 1.91–1.84 (m, 2H), 1.74 (s, 3H), 1.68 (s, 4H), 1.60 (s, 3H), 1.01 (s, 3H). $^{13}$C NMR (151 MHz, $CDCl_3$) δ 223.4, 137.6, 131.7, 130.2, 126.6, 124.3, 124.0, 48.7, 40.1, 39.9, 37.8, 35.2, 26.6, 25.7, 22.0, 18.7, 17.7, 16.7. HRMS (ESI) m/z: $[M+Na]^+$ calcd. for $C_{18}H_{28}NaO^+$: 283.2032. Found: 283.2038.

## 2-allyl-2-methylcyclopentan-1-one (6k)[6]

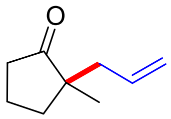

Colorless oil. 66% yield, 97:3 r.r.; the yield and r.r. determined by GC analysis using 2-nonanone as internal standard. MS m/z: calcd. For $C_9H_{14}O^+$: 138.1. Found: 138.1.

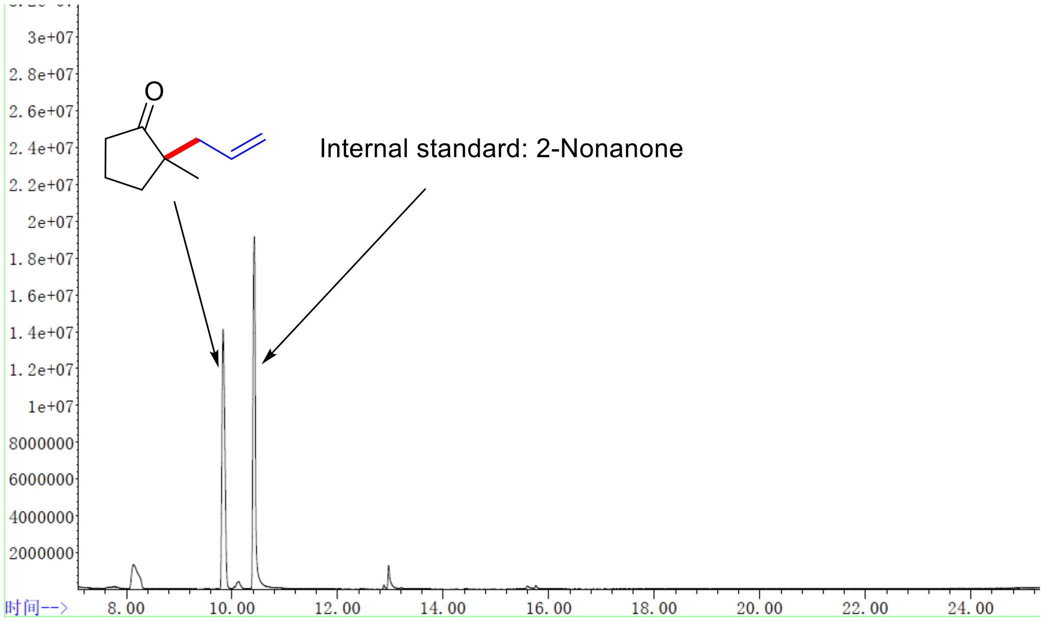


**Supplementary Fig. 6.** GC spectrum of **6j**.

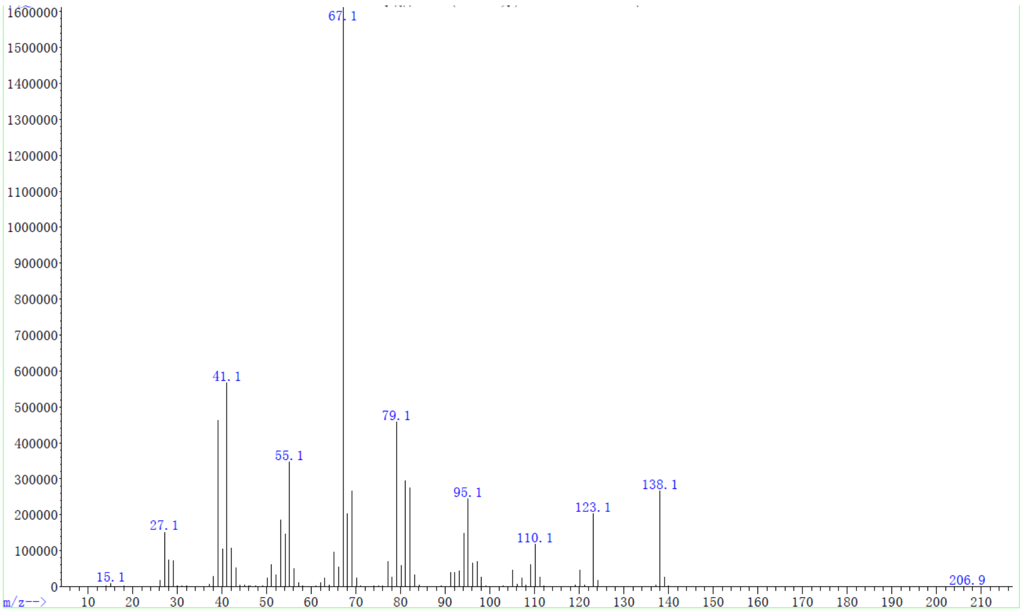


**Supplementary Fig. 7.** Mass spectrum of **6j**.

**ethyl (*E*)-4-acetyl-7-(4-methoxyphenyl)hept-6-enoate (3aa)**

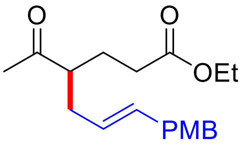


Colorless oil. 24.9 mg; 82% yield. $^1$H NMR (400 MHz, $CDCl_3$) δ 7.25 (d, *J* = 8.6 Hz, 2H), 6.83 (d, *J* = 8.8 Hz, 2H), 6.35 (d, *J* = 15.8 Hz, 1H), 5.94 (dt, *J* = 15.7, 7.2 Hz, 1H), 4.12 (q, *J* = 7.1 Hz, 2H), 3.80 (s, 3H), 2.72–2.66 (m, 1H), 2.52–2.45 (m, 1H), 2.37–2.24 (m, 3H), 2.17 (s, 3H), 2.03–1.94 (m, 1H), 1.85–1.76 (m, 1H), 1.25 (t, *J* = 7.1 Hz, 3H). $^{13}$C NMR (101 MHz, $CDCl_3$) δ 211.3, 173.2, 159.1, 131.9, 130.0, 127.4, 124.3, 114.1 60.6, 55.4, 52.0, 35.1, 31.9, 29.8, 25.9, 14.4. HRMS (ESI) m/z: $[M+Na]^+$ calcd. for $C_{18}H_{24}NaO_4^+$: 327.1567. Found: 327.1570.

**(*E*)-6-(4-methoxyphenyl)-3-(2-(pyridin-2-yl)ethyl)hex-5-en-2-one (3ab)**

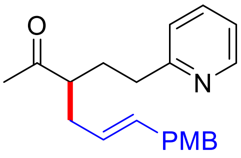


Colorless oil. 21.5 mg; 70% yield. $^1$H NMR (400 MHz, $CDCl_3$) δ 8.53 (d, *J* = 3.8 Hz, 1H), 7.64 1–7.57 (m, 1H), 7.25 (d, *J* = 8.6 Hz, 2H), 7.14–7.10 (m, 2H), 6.83 (d, *J* = 8.7 Hz, 2H), 6.35 (d, *J* = 15.7 Hz, 1H), 5.96 (dt, *J* = 15.2, 7.2 Hz, 1H), 3.80 (s, 3H), 2.84–2.75 (m, 2H), 2.73–2.66 (m, 1H), 2.55–2.48 (m, 1H), 2.42–2.36 (m, 1H), 2.17–2.07 (m, 4H), 1.97–1.91 (m, 1H). $^{13}$C NMR (101 MHz, $CDCl_3$) δ 211.8, 161.3, 159.1, 149.4, 136.5, 131.6, 130.1, 127.3, 124.7, 123.0, 121.3, 114.0, 55.4, 52.6, 36.0, 35.2, 31.0, 29.6. HRMS (ESI) m/z: $[M+Na]^+$ calcd. for $C_{20}H_{23}NNaO_2^+$: 332.1621. Found: 332.1625.

**(5*R*)-2-((*E*)-3-(4-methoxyphenyl)allyl)-2-methyl-5-(prop-1-en-2-yl)cyclohexan-1-one (3ac)**

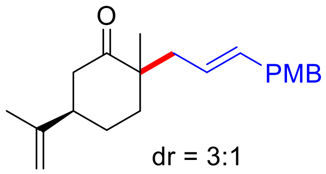


Colorless oil. 22.3 mg; 75% yield. dr = 3:1. $^{1}$H NMR (400 MHz, $CDCl_3$) δ 7.29–7.24 (m, 2H), 6.84– 6.81 (m, 2H), 6.37–6.30 (d, *J* = 15.7 Hz, 1H), 6.10–6.02 (m, 0.28H), 5.92–5.84 (m, 0.70H), 4.79–4.72 (m, 2H), 3.79 (s, 3H), 2.56–2.50 (m, 1.71H), 2.41–2.29 (m, 3.29H), 1.96–1.92 (m, 0.70H), 1.81–1.74 (m, 5.60H), 1.56–1.49 (m, 0.70H), 1.17 (s, 0.75H), 1.08 (s, 2.25H). $^{13}$C NMR (101 MHz, $CDCl_3$) δ 215.0, 214.8, 159.1, 158.9, 147.7, 132.8, 132.4, 130.6, 130.2, 127.4, 127.3, 124.4, 122.4, 114.0, 110.0, 110.0, 55.4, 48.6, 48.0, 46.2, 46.0, 43.7, 43.6, 41.7, 40.8, 37.4, 36.6, 26.3, 26.2, 23.5, 22.5, 20.9, 20.8. HRMS (ESI) m/z: $[M+Na]^+$ calcd. for $C_{20}H_{26}NaO_2^+$: 321.1825. Found: 321.1831.

**(*E*)-1-(4-acetyl-7-(4-methoxyphenyl)hept-6-en-1-yl)-3,7-dimethyl-3,7-dihydro-1H-purine-2,6-dione (3ad)**

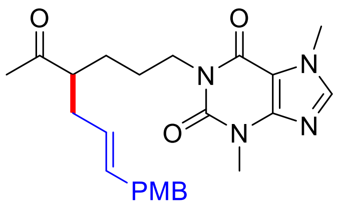


Colorless oil. 17.60 mg; 40% yield. $^{1}$H NMR (600 MHz, $CDCl_3$) δ 7.49 (s, 1H), 7.23 (d, *J* = 8.0 Hz, 2H), 6.82 (d, *J* = 8.4 Hz, 2H), 6.33 (d, *J* = 15.2 Hz, 1H), 5.98–5.91 (m, 1H), 4.00–3.93 (m, 5H), 3.80 (s, 3H), 3.56 (s, 3H), 2.71–2.68 (m, 1H), 2.50–2.43 (m, 1H), 2.35–2.31 (m, 1H), 2.16 (s, 3H), 1.72–1.56 (m, 4H). $^{13}$C NMR (151 MHz, $CDCl_3$) δ 212.0, 159.0, 155.3, 151.6, 148.9, 141.5, 131.6, 130.2, 127.3, 127.2, 124.9, 114.0, 55.4, 52.6, 41.2, 35.0, 33.7, 29.9, 29.8, 28.4, 25.9. HRMS (ESI) m/z: $[M+Na]^+$ calcd. for $C_{23}H_{28}N_4NaO_4^+$: 447.2003. Found: 447.2008.

**(*E*)-3-((*R*)-2-((3*R*, 5*S*, 7*S*, 8*R*, 9*S*, 10*S*, 13*R*, 14*S*, 17*R*)-3,7-dihydroxy-10,13-dimethylhexadecahydro-1H-cyclopenta[a]phenanthren-17-yl)propyl)-7-(4-methoxyphenyl)hept-5-en-2-one (3ae)**

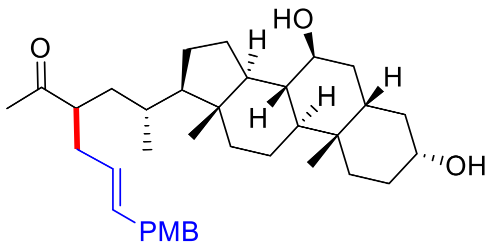


Colorless oil.. 25.0 mg; 47% yield. $^{1}$H NMR (600 MHz, $CDCl_3$) δ 7.26–7.23 (m, 2H), 6.84–6.82 (m, 1H), 6.34 (d, *J* = 15.6 Hz, 1H), 5.95 (dt, *J* = 15.2, 7.2 Hz, 1H), 3.80 (s, 3H), 3.61–3.56 (m, 2H), 2.79–2.74 (m, 0.56H), 2.67–2.63 (m, 0.44H), 2.45–2.40 (m, 1H), 2.36–2.34 (m, 0.44H), 2.26–2.22 (m, 0.56H), 2.17–2.13 (m, 3H), 2.03–1.79 (m, 6H), 1.66–1.43 (m, 12H), 1.30–1.22 (m, 5H), 1.12–1.01 (m, 3H), 0.96–0.93 (m, 6H), 0.69– 0.64 (m, 3H). $^{13}$C NMR (151 MHz, $CDCl_3$) δ 212.8, 212.3, 159.1, 159.0, 131.5, 131.4, 130.3, 130.2, 127.4, 127.3, 125.3, 125.0, 114.1, 114.1, 71.6, 71.5 (two signals), 56.0, 55.9 (three signal), 55.4, 50.9, 50.7, 44.0, 43.9 (two signals), 42.6, 40.3, 39.3, 38.4, 37.5, 37.4, 37.0 (two signals), 35.1, 34.6, 34.3, 34.2, 30.5, 29.4,

29.3, 29.1, 29.0, 27.0, 23.5, 21.3, 18.9, 18.8, 12.3 (two signals). HRMS (ESI) m/z: $[M+Na]^+$ calcd. for $C_{35}H_{52}NaO_4^+$: 559.3758. Found: 559.3763.

**(3*S*, 8*R*, 9*S*, 10*R*, 13*S*, 14*S*)-10,13-dimethyl-17-(pyridin-3-yl)-2, 3, 4, 7, 8, 9, 10, 11, 12, 13, 14, 15-dodecahydro-1H-cyclopenta[a]phenanthren-3-yl (E)-4-acetyl-7-(4-methoxyphenyl)hept-6-enoate (3af)**

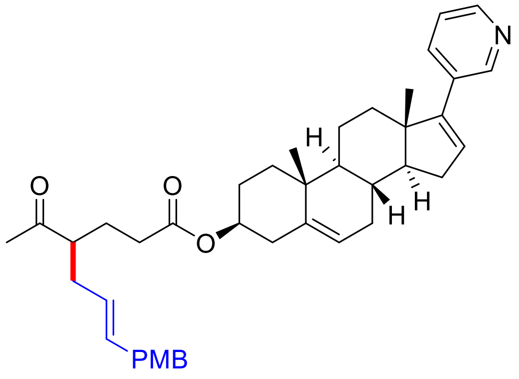


Colorless oil.. 25.1 mg; 41% yield. $^1$H NMR (400 MHz, $CDCl_3$) δ 8.63 (s, 1H), 8.46 (d, *J* = 4.8 Hz, 1H), 7.66–7.63 (m, 1H), 7.26–7.20 (m, 3H), 6.85–6.82 (m, 2H), 6.42–6.34 (m, 1H), 6.00–5.89 (m, 2H), 5.42–5.41 (m, 1H), 4.66–4.58 (m, 1H), 3.80–3.79 (m, 3H), 2.72–2.66 (m, 0.75H), 2.52–2.47 (m, 1.25H), 2.34–2.25 (m, 5H), 2.20–2.18 (m, 3H), 2.10–2.03 (m, 3H), 1.87–1.46 (m, 11H), 1.29–1.27 (m, 2H), 1.18–1.12 (m, 1H), 1.07 (s, 3H), 1.05 (s, 3H). $^{13}$C NMR (101 MHz, $CDCl_3$) δ 211.4, 172.6, 159.1, 151.8, 148.0 (two signals), 140.1, 133.8, 133.2, 133.1, 131.9, 130.0, 129.3, 127.4 (two signals), 124.4, 123.14, 122.5, 122.4, 114.1, 74.1, 57.6, 55.4 (two signals), 52.0, 50.4, 47.4, 38.3, 37.0, 36.9, 35.3, 35.0, 32.3, 31.9, 31.6, 31.5, 30.5, 29.8, 27.9, 26.0, 20.9, 19.4, 16.7. HRMS (ESI) m/z: $[M+Na]^+$ calcd. for $C_{40}H_{49}NNaO_4^+$: 630.3554. Found: 630.3558.

**(*E*)-3-((6-methoxynaphthalen-2-yl)methyl)-6-(4-methoxyphenyl)hex-5-en-2-one (3ag)**

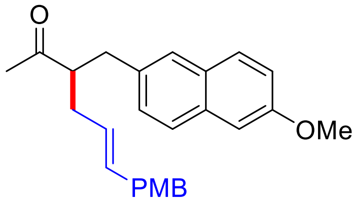


Colorless oil. 23.6 mg; 63% yield. $^1$H NMR (400 MHz, $CDCl_3$) δ 7.67 (d, *J* = 8.5 Hz, 2H), 7.53 (s, 1H), 7.28–7.23 (m, 3H), 7.14–7.10 (m, 2H), 6.83 (d, *J* = 8.4 Hz, 2H), 6.34 (d, *J* = 15.7 Hz, 1H), 5.98 (dt, *J* = 15.3, 7.3 Hz, 1H), 3.91 (s, 3H), 3.79 (s, 3H), 3.09–3.04 (m, 2H), 2.90–2.84 (m, 1H), 2.56–2.49 (m, 1H), 2.41–2.35 (m, 1H), 2.01 (s, 3H). $^{13}$C NMR (101 MHz, $CDCl_3$) δ 211.9, 159.1, 157.5, 134.7, 133.3, 131.9, 130.1, 129.1, 129.1, 127.9, 127.3, 127.1, 124.6, 119.0, 114.1, 105.7, 55.4, 54.8, 37.6, 35.2, 30.9. HRMS (ESI) m/z: $[M+Na]^+$ calcd. for $C_{25}H_{26}NaO_3^+$: 397.1774. Found: 397.1780.

## 2.3 Computational Details

**Computational methods:**

All DFT theoretical calculations have been carried out using the Gaussian 16 program package (Gaussian 16, Revision A.03).[7] The B3LYP[8–10] method with SDD[11] basis set for Ni and 6-31G(d)[12,13] for other atoms has been selected for geometry optimizations and calculation of Gibbs energy corrections with the SMD model[14] [isopropanol solvent (IPA)], including the D3BJ dispersion correction scheme developed by Grimme[15]. Final energies were retrieved from single-point calculations at the PBE0[16] level of theory with def2-TZVPP[17,18] with the SMD model (IPA) on B3LYP-D3(BJ)-optimized geometries, including the D3BJ dispersion correction scheme developed by Grimme. No symmetry constraints were applied. All the transition states were verified by IRC (intrinsic reaction coordinate)[19] calculations. The reported Gibbs free energies in solution include thermal corrections computed at 353.15 K at the standard concentration (1 mol/L) with Shermo version 2.3.4, using Grimme's quasi rigid rotor-harmonic oscillator (qRRHO) approach and adopting the 0.9806 as the scaling factors for the zero-point vibrational energy. Graphical structures are visualized with CYLview.[20]

Methyl ethyl ketone has four possible forms when it undergoes nucleophilic attack on the π-allylnickel intermediate in isopropanol solvent, including enol, the enol anion, and their isopropanol hydrogen-bonding forms. To determine which form of methyl ethyl ketone to attack π-allylnickel intermediate, we compared the free energy of each of these four forms. The calculation results indicated that the enol anion of methyl ethyl ketone is the main form to attack on the allyl nickel.

**Supplementary Table 1.** Free energies of various enolates and enolate complexes at 353.15K. All energies are in kcal/mol and relative to that of ketone **1n**.

| Enol complex | Free energy | Enol complex | Free energy |
|---|---|---|---|
| 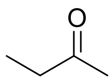 **1n** | 0.0 | 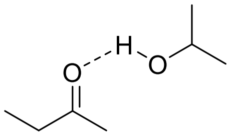 **1n**_IPA | 7.7 |
| 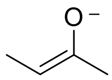 **Ea** | 4.9 | 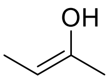 **Ea**_H | 11.4 |
| 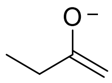 **Eb** | 5.8 | 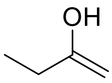 **Eb**_H | 15.0 |
| 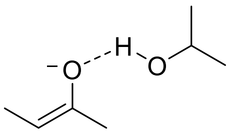 **Ea**_IPA | 7.4 | 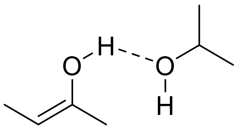 **Ea**_H_IPA | 17.7 |
| 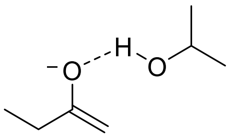 **Eb**_IPA | 8.6 | 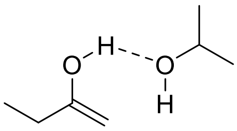 **Eb**_H_IPA | 20.4 |

**Supplementary Table 2.** Free energies of various transition states of C–O bond oxidative addition on Ni(0) at 353.15K. All energies are in kcal/mol and relative to that of **TS1**

| Transition states | Free energy | Transition states | Free energy |
|---|---|---|---|
| 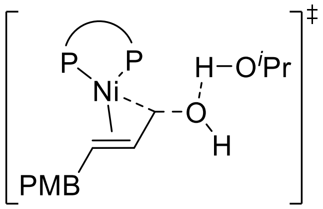 **TS1** | 0 | 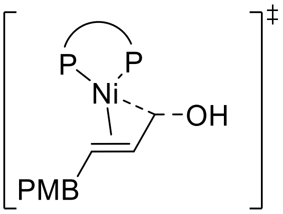 **TS1'** | 2.0 |
| 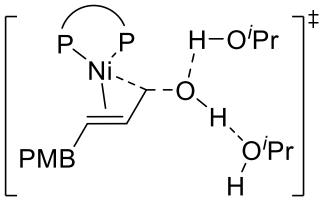 **TS1''** | 8.2 | 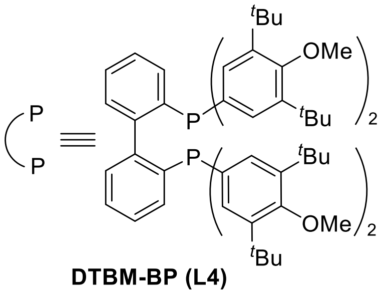 **DTBM-BP (L4)** | |

**Supplementary Table 3.** Free energies of various transition states of C–C bond formation at 353.15K. All energies are in kcal/mol and relative to that of **TS2**

| Transition states | Free energy | Transition states | Free energy |
|---|---|---|---|
| 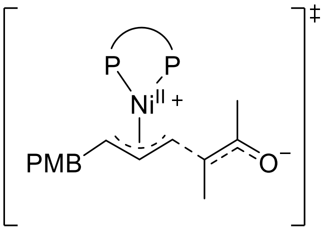 **TS2** | 0 | 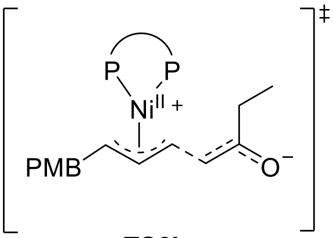 **TS2'** | 3.1 |
| 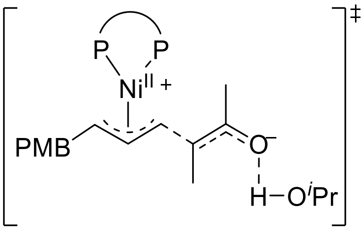 **TS3** | 2.6 | 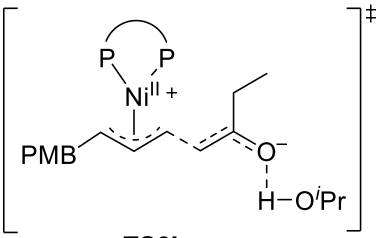 **TS3'** | 5.9 |

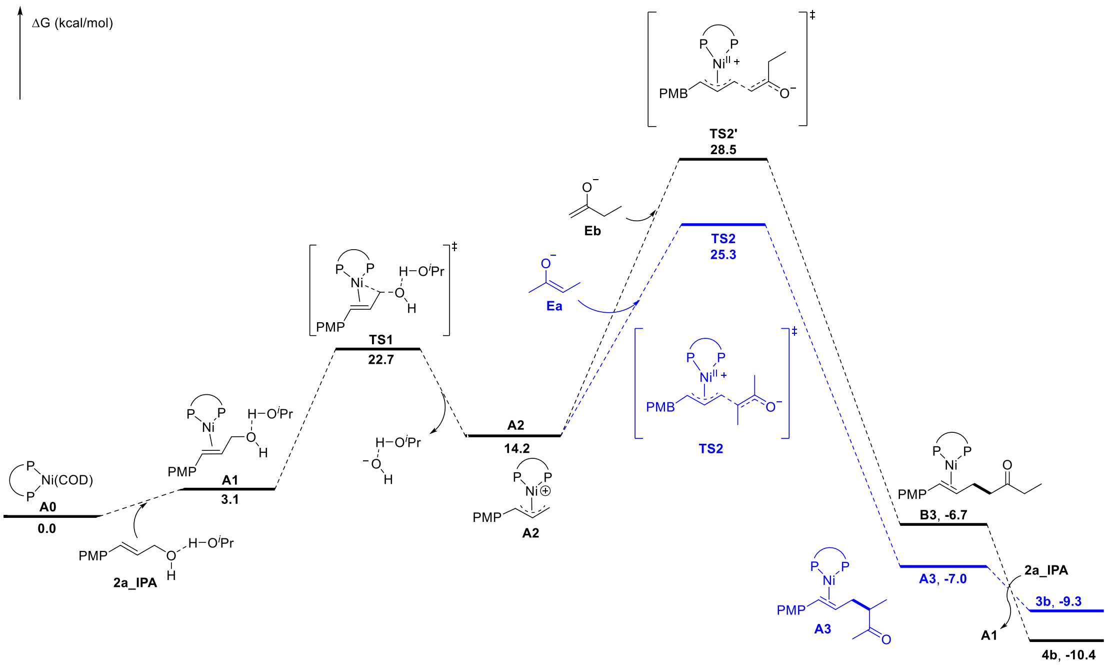


**Supplementary Fig. 8.** The Gibbs free energy profile of the Ni(COD)/L4-catalyzed allylic alkylation of ketone **1n** with allylic alcohol **2a**. Energies are in kcal/mol and calculated using PBE0-D3(BJ)/def2-TZVPP/SMD-($^i$PrOH)//B3LYP-D3(BJ)/SDD(Ni)+6-31G(d)/SMD-($^i$PrOH) method.

As shown in Supplementary Fig. 8, after the first ligand exchange between one COD and DTBM-BP produces (DTBM-BP)Ni(COD), the reaction of (DTBM-BP)Ni(COD) with allylic alcohol **2a_IPA** forms **A1**. Cleavage of the C−O bond of allylic alcohol **2a** proceeds smoothly to afford the cationic nickel-π–allyl complex **D** with the help of $^i$PrOH, via transition state **TS1**. The results suggest that the cleavage of the stable C–O bond can be easily achieved in $^i$PrOH through hydrogen-bond activation, which is consistent with the experimental results of screening solvents (Table S1). It is a reliable pathway based on the previous related mechanistic study of allylic alkylation, in which the cationic complex **A2** reacts with outer-sphere enolate **Ea** to give a product adduct **A3**. Finally, **A3** undergoes deprotonation and releases the allylated product **3a** by binding with allylic alcohol **2a_IPA** to form **A1** complex, which starts the next catalytic cycle. The C−C bond formation processes have the highest energy barriers (**TS2** and **TS2′**) with enolate **Ea** and enolate **Eb**, respectively. Thus, the C−C bond formation step (**A2** → **TS2** → **A3** and **A2** → **TS2′** → **B3**) is suggested to be the rate-determining step; of note, the corresponding **TS2** is more stable than **TS2′** by 3.2 kcal/mol, indicating that the nucleophilic attack to cationic nickel-π-allyl intermediate occurred preferably with the more-substituted enolate **Ea**. In addition, ratios of all the enolates of 2-butanone (**1b**) were also calculated assuming the Boltzmann distribution (Table S5). The calculated result clearly shows that the enolates possessing more substitutes are more stable than less-substituted enolates [(**Ea**+**Ea′**/**Eb**+**Eb′**) =70.1:29.9], which facilitates the controlling of the regioselectivity of this reaction. Therefore, the allylated product at the more-hindered site also should be exclusive, which is consistent with the experimental results.

**Supplementary Table 4.** Thermodynamic stability of possible enolates in $^{i}$PrOH; All energies are in kcal/mol and relative to that of **Ea**; Boltzmann distribution assumption

| Enolate | Free energy (kcal/mol) | Relative abundance (%) |
|---|---|---|
| 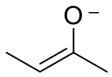 **Ea** | 0.0 | 68.8 |
| 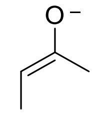 **Ea'** | 2.8 | 1.3 |
| 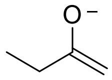 **Eb** | 0.9 | 19.1 |
| 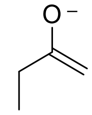 **Eb'** | 1.3 | 10.8 |

**Supplementary Table 5.** Free energies of various transition states of C–C bond formation of ketone **1l** at 353.15K. All energies are in kcal/mol and relative to that of **TS4**

| Transition states | Free energy | Transition states | Free energy |
|---|---|---|---|
| 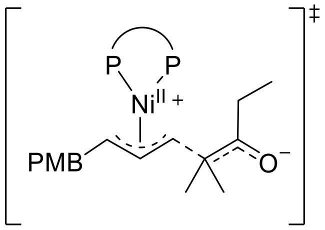 **TS4** | 0 | 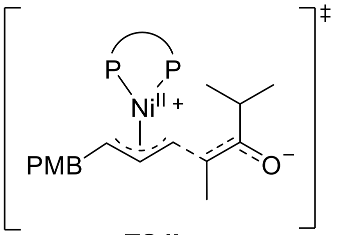 **TS4'** | 1.1 |

**Supplementary Table 6.** The PBE0-D3(BJ)/def2-TZVPP/SMD-($^{i}$PrOH)//B3LYP-D3(BJ)/SDD(Ni)+6-31G(d)/SMD-($^{i}$PrOH) calculated Gibbs energy corrections, single-point energies, Gibbs energies and final corrected Gibbs energies (Hartree)$^{a}$

| Structure | Gibbs energy correction | Single-point energy | Gibbs energy | Corrected Gibbs energy |
|---|---|---|---|---|
| **1n** | 0.075522 | -232.3018411 | -232.2263191 | -232.2275784 |
| **1n_IPA** | 0.17121 | -426.5322931 | -426.3610831 | -426.3615535 |
| **Ea** | 0.062863 | -231.7871286 | -231.7242656 | -231.7259893 |
| **Ea′** | 0.062423 | -231.7827698 | -231.7203468 | -231.7217053 |
| **Eb** | 0.064486 | -231.7873053 | -231.7228193 | -231.7245523 |
| **Eb′** | 0.064348 | -231.7866537 | -231.7223057 | -231.7239593 |
| **Ea_IPA** | 0.159459 | -426.0266392 | -425.8671802 | -425.8682051 |
| **Eb_IPA** | 0.16077 | -426.0259381 | -425.8651681 | -425.866296 |
| **Ea_H** | 0.077031 | -232.2842746 | -232.2072436 | -232.2093413 |
| **Eb_H** | 0.077513 | -232.2800081 | -232.2024951 | -232.2035979 |
| **Ea_H_IPA** | 0.172183 | -426.5169449 | -426.3447619 | -426.3456186 |
| **Eb_H_IPA** | 0.172799 | -426.5133938 | -426.3405948 | -426.3413361 |
| **TS1** | 1.685462 | -6025.650275 | -6023.964813 | -6023.9704191 |
| **TS1′** | 1.587676 | -5831.401397 | -5829.813721 | -5829.8209604 |
| **TS1″** | 1.784766 | -6219.883647 | -6218.098881 | -6218.1035741 |
| **TS2** | 1.672514 | -5987.326673 | -5985.654159 | -5985.6618177 |
| **TS2′** | 1.674221 | -5987.323278 | -5985.649057 | -5985.6568314 |
| **TS3** | 1.772621 | -6181.569799 | -6179.797178 | -6179.8039237 |
| **TS3′** | 1.772663 | -6181.565012 | -6179.792349 | -6179.7987545 |
| **TS4** | 1.727357 | -6065.8936487 | -6064.166292 | -6064.1746336 |
| **TS4′** | 1.729017 | -6065.8932745 | -6064.164257 | -6064.1729354 |
| **A0** | 1.581738 | -5604.910999 | -5603.329261 | -5603.3384209 |
| **A1** | 1.688822 | -6025.685953 | -6023.997131 | -6024.0017076 |

| | | | | |
|---|---|---|---|---|
| **A2** | 1.585818 | -5755.53745 | -5753.951632 | -5753.9610399 |
| **A3** | 1.674543 | -5987.38147 | -5985.706927 | -5985.7133636 |
| **B3** | 1.675541 | -5987.381611 | -5985.70607 | -5985.7129001 |
| **3n** | 0.230339 | -694.2466402 | -694.0163012 | -694.0169304 |
| **4n** | 0.229719 | -694.2482354 | -694.0185164 | -694.018755 |
| **$OH^-$_IPA** | 0.07855 | -270.1153337 | -270.0367837 | -270.0381617 |
| **2a_IPA** | 0.246388 | -732.5480418 | -732.3016538 | -732.3016281 |
| **IPA** | 0.074346 | -194.221541 | -194.147195 | -194.1492888 |
| **$H_2O$_IPA** | 0.090867 | -270.6219548 | -270.5310878 | -270.5319738 |
| **COD** | 0.142945 | -311.7846233 | -311.6416783 | -311.6449081 |

[a] The scaling factors for the zero-point vibrational energy was adopted 0.9806, and the free energies were corrected using Grimme's quasi rigid rotor-harmonic oscillator (qRRHO) approach.

A B

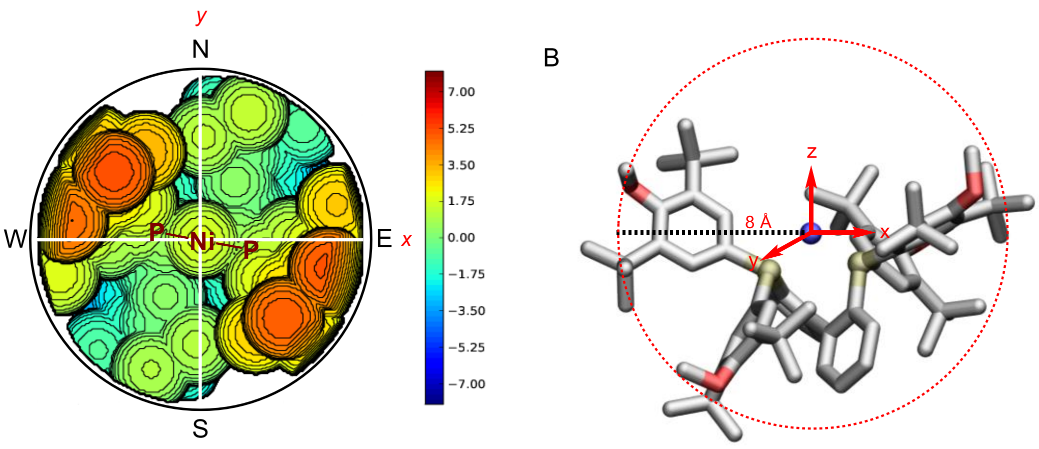


%$V_{Bur}$=48.0

C

| %V Free | **%V Buried** | % V Tot/V Ex |
|---|---|---|
| 52.0 | **48.0** | 100.0 |

| Quadrant | V f | V b | V t | %V f | **%V b** |
|---|---|---|---|---|---|
| SW | 279.4 | 256.5 | 535.9 | 52.1 | **47.9** |
| NW | 280.4 | 255.5 | 535.9 | 52.3 | **47.7** |
| NE | 269.3 | 266.6 | 535.9 | 50.3 | **49.7** |
| SE | 284.5 | 251.4 | 535.9 | 53.1 | **46.9** |

**Supplementary Fig. 9. A**, Steric map of the catalyst Ni(II)/**L4** on the basis of the DFT-optimized structure of TS2. The steric map is viewed down the *z*-axis;. The red and blue zones indicate the more-hindered and less-hindered zones in the catalytic pocket, respectively. %VBur, percentage of buried volume. **B**, the orientation of the catalyst. **C**, The calculated percentage of buried volume and free volume[21].

To understand the recognition of the two isomeric enolates of 2-butanone, the space-constrained and deep pocket of the nickel catalyst were investigated by DFT calculations. Results are shown below:

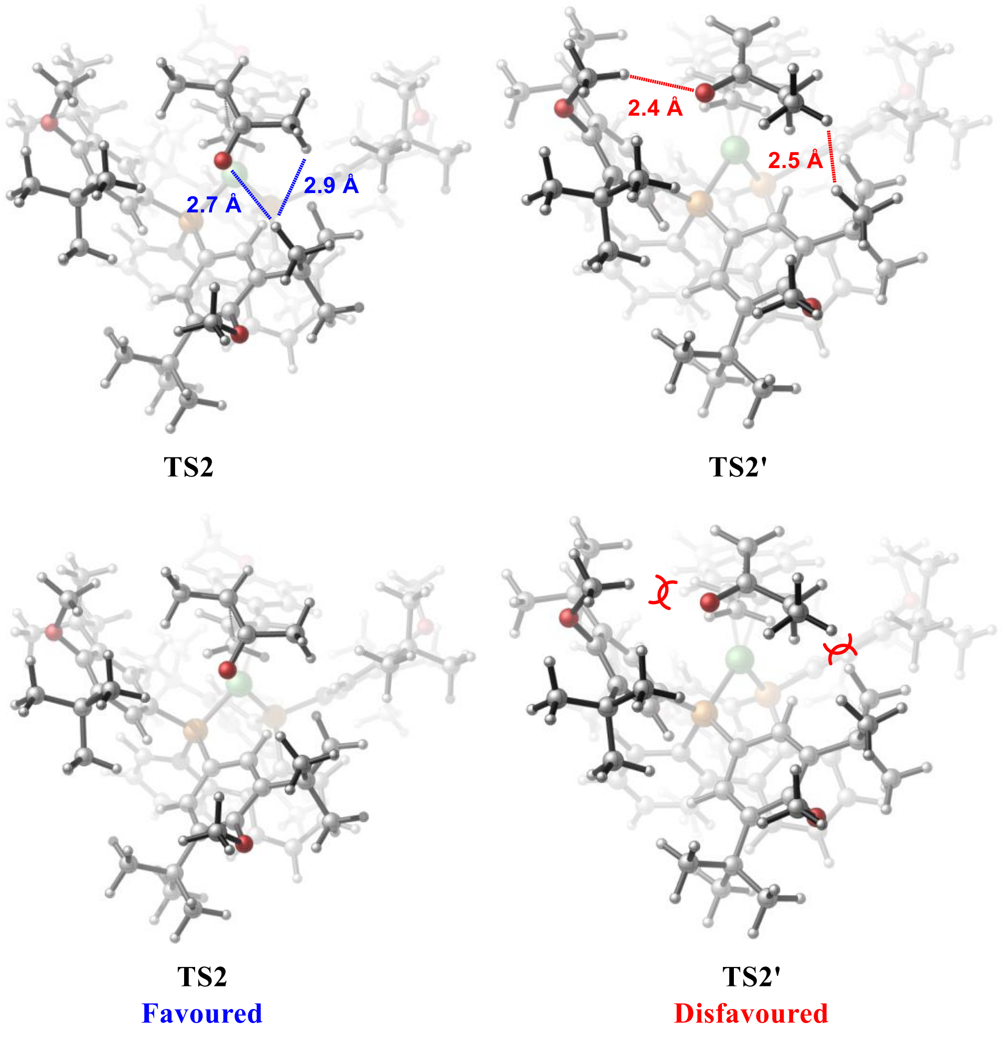


**Supplementary Fig. 10.** Optimized transition state structures **TS2** and **TS2′**; the pocket of the nickel catalyst can differentiate the two isomeric enolates and thus the regioselectivity is high.

# 3 Supplementary Figures

## 3.1 NMR Spectra of New Ligand and Products

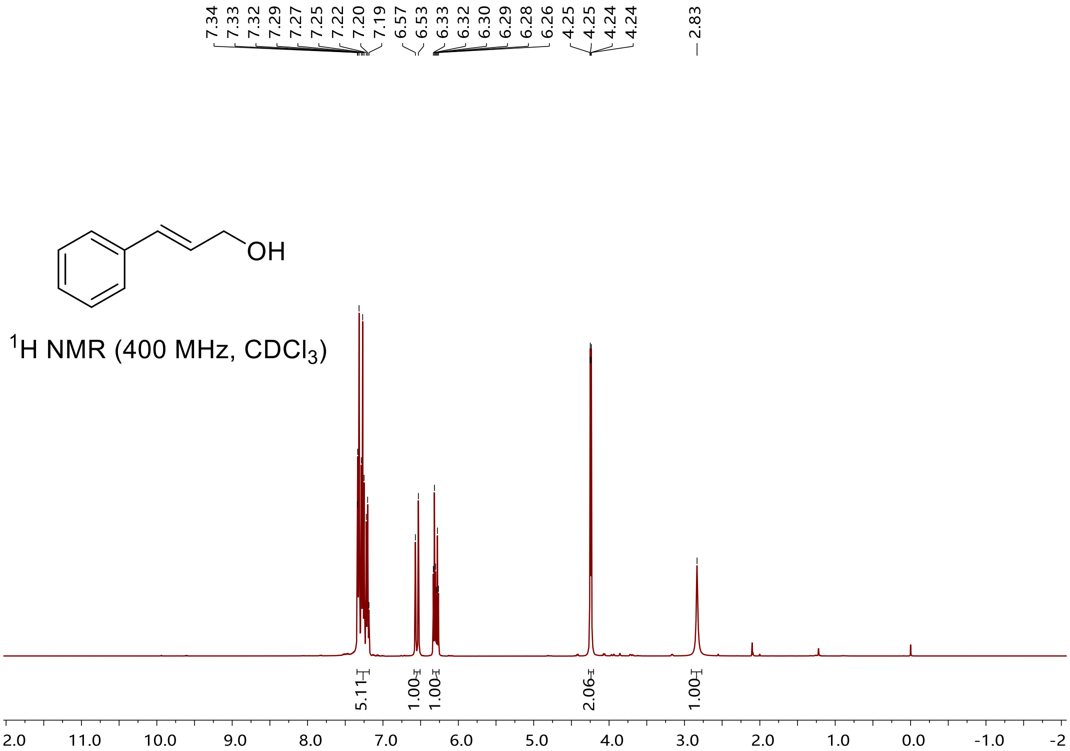


**Supplementary Fig. 11.** $^1$H NMR spectrum of compound **5a**.

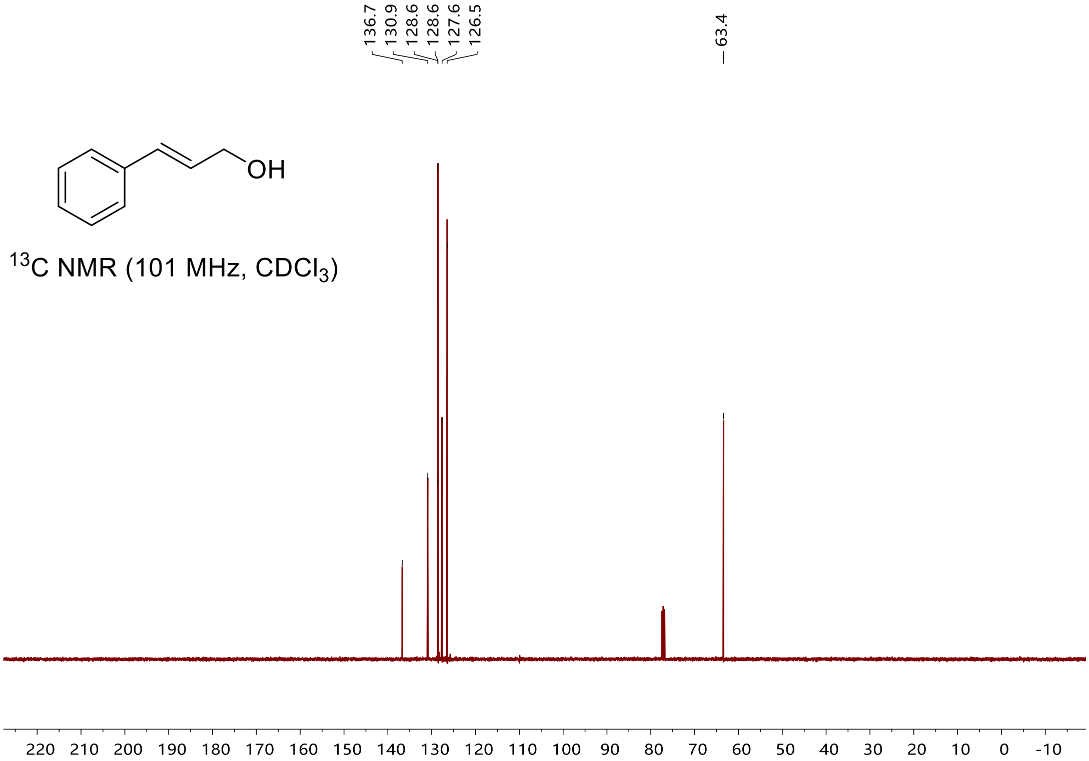


**Supplementary Fig. 12.** $^{13}$C NMR spectrum of compound **5a**.

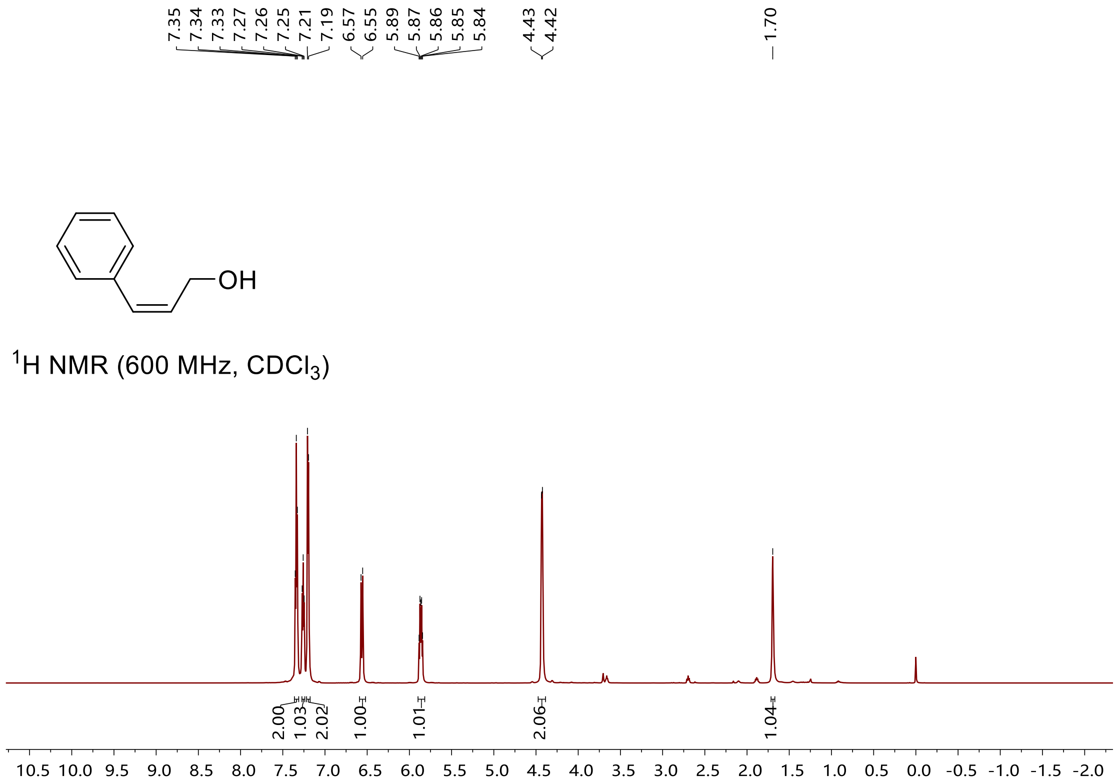


**Supplementary Fig. 13.** $^1$H NMR spectrum of compound **5b**.

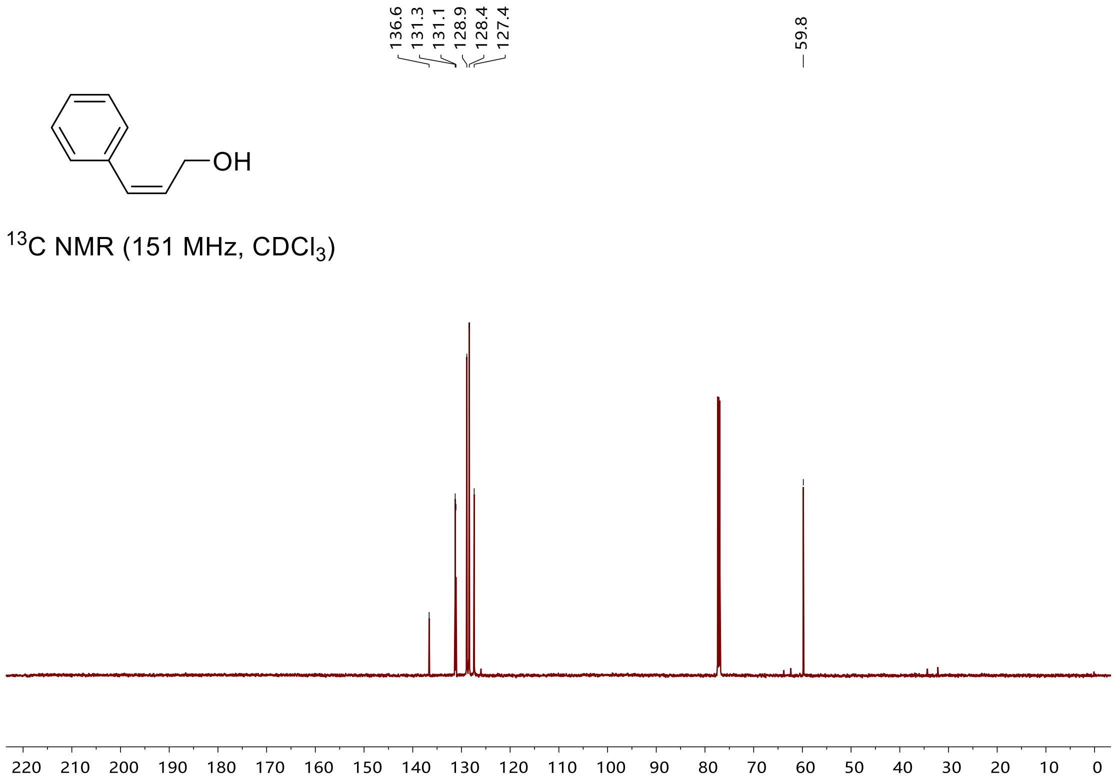


**Supplementary Fig. 14.** $^{13}$C NMR spectrum of compound **5b**.

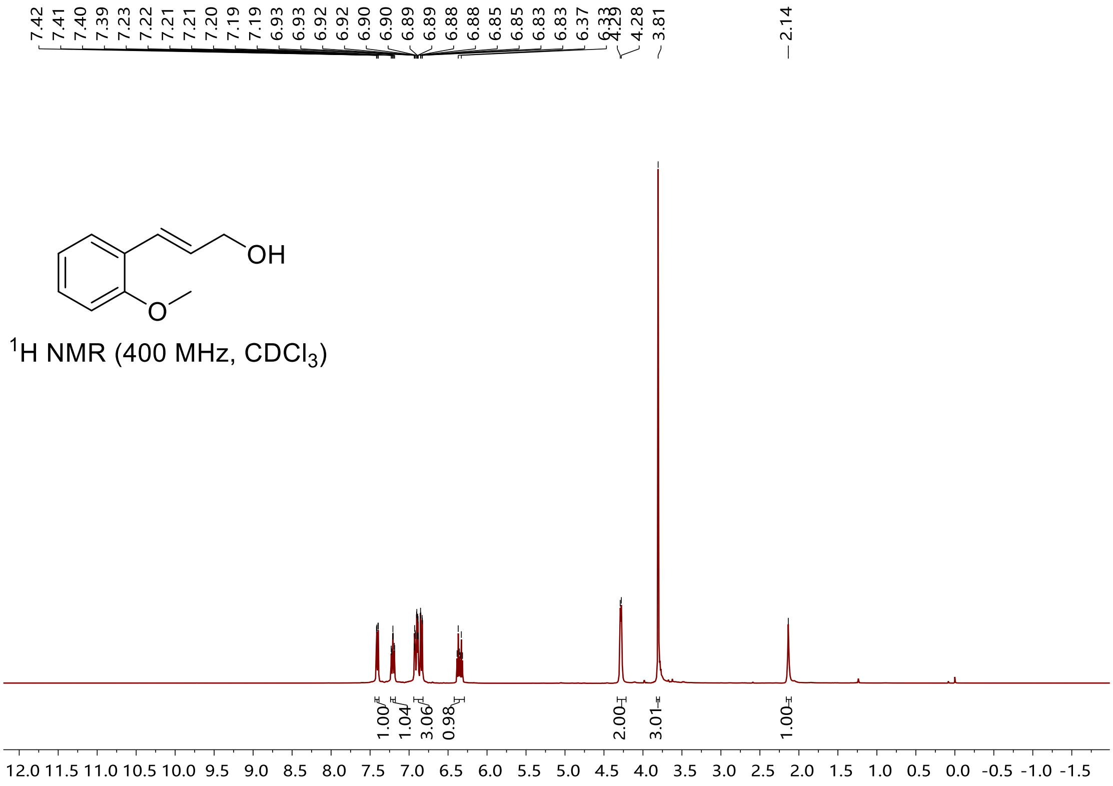


**Supplementary Fig. 15.** $^{1}$H NMR spectrum of compound **5c**.

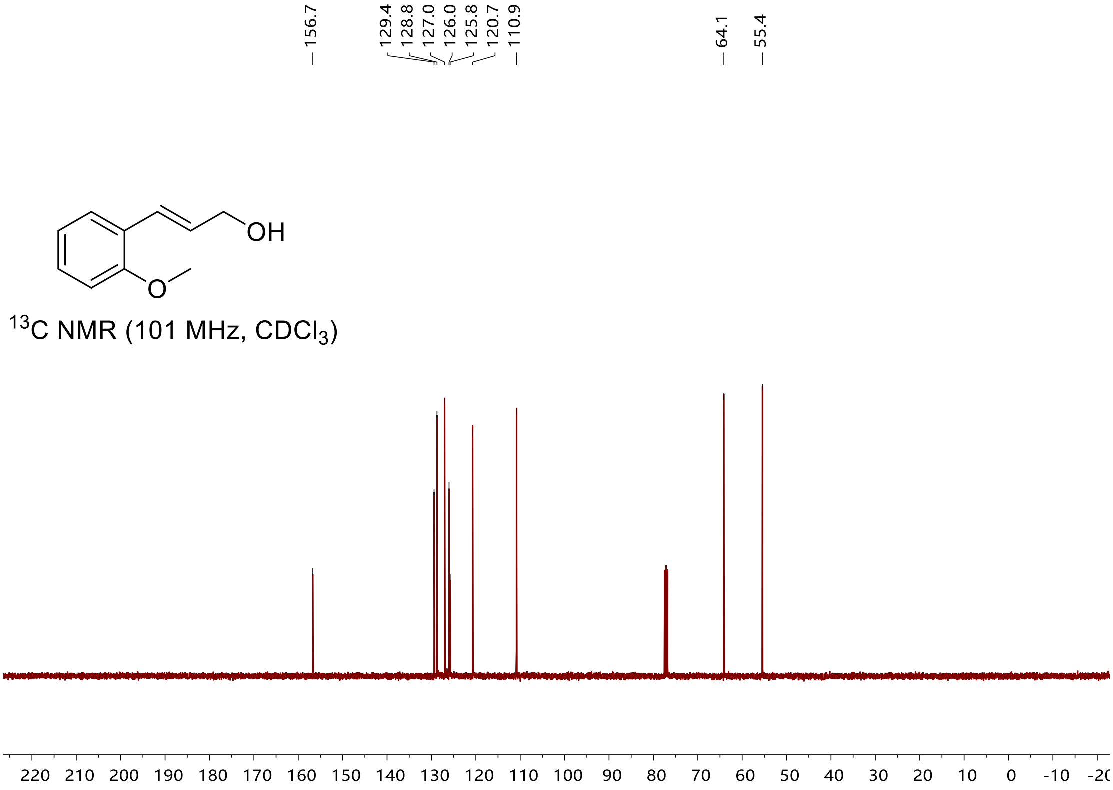


**Supplementary Fig. 16.** $^{13}$C NMR spectrum of compound **5c**.

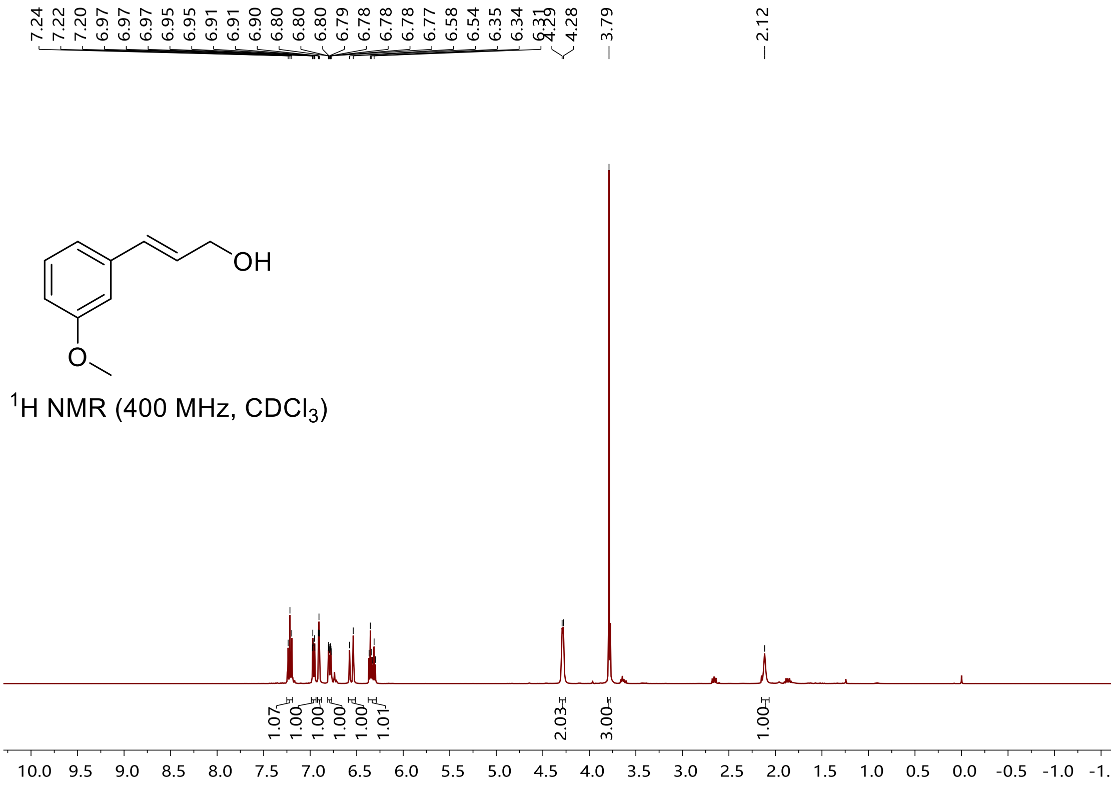


**Supplementary Fig. 17.** $^1$H NMR spectrum of compound **5d**.

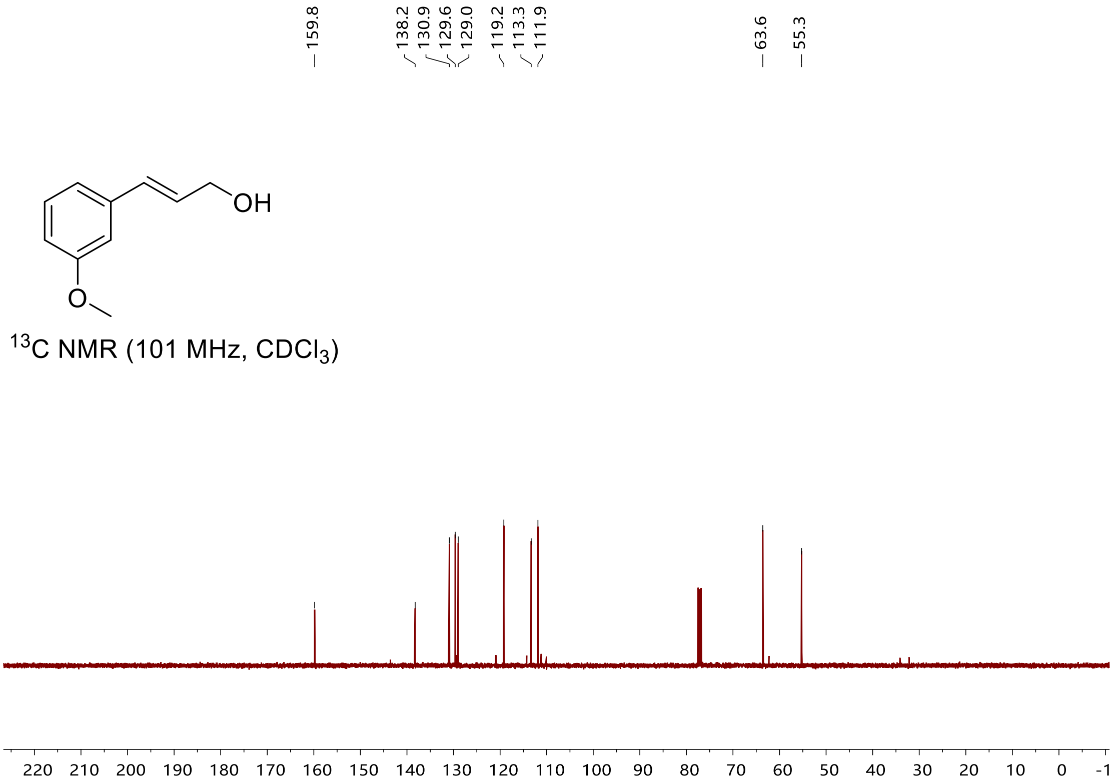


**Supplementary Fig. 18.** $^{13}$C NMR spectrum of compound **5d**.

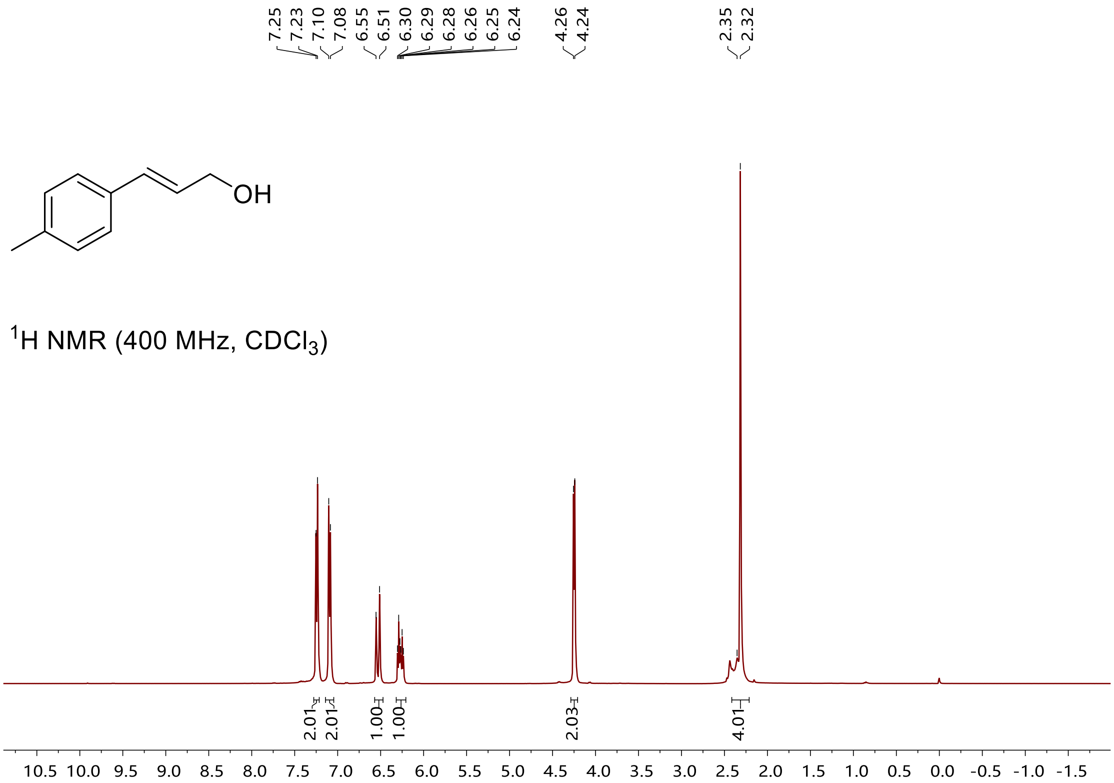


**Supplementary Fig. 19.** $^1$H NMR spectrum of compound **5e**.

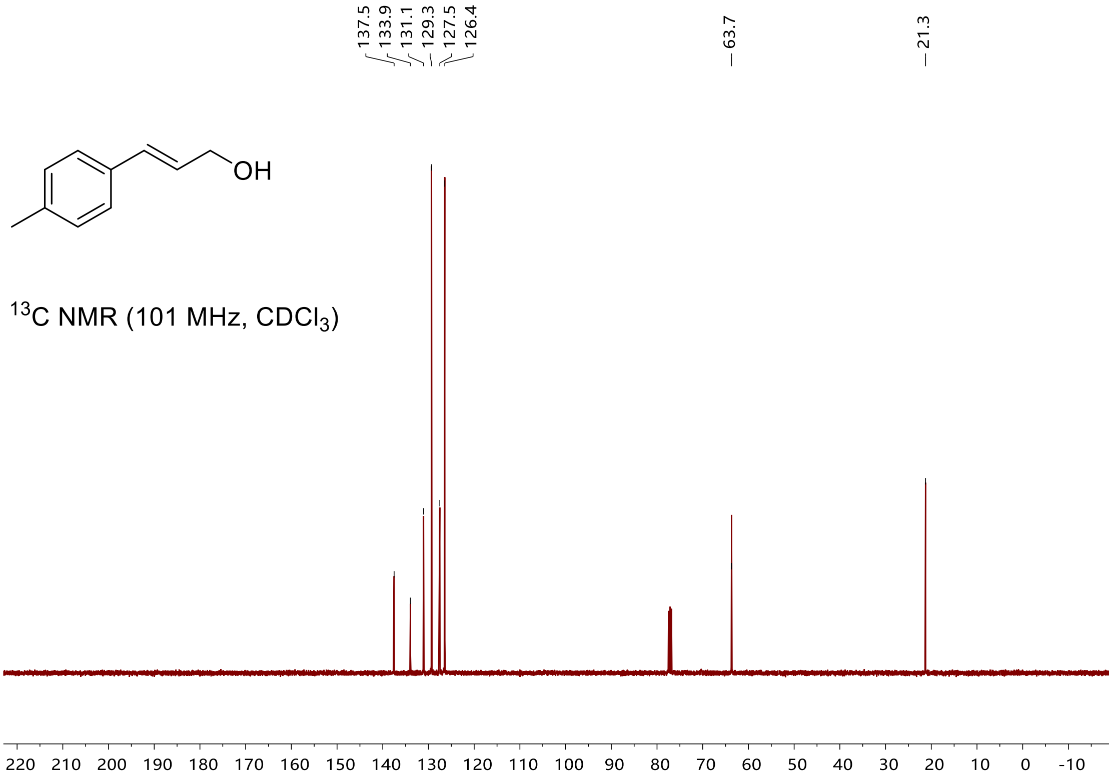


**Supplementary Fig. 20.** $^{13}$C NMR spectrum of compound **5e**.

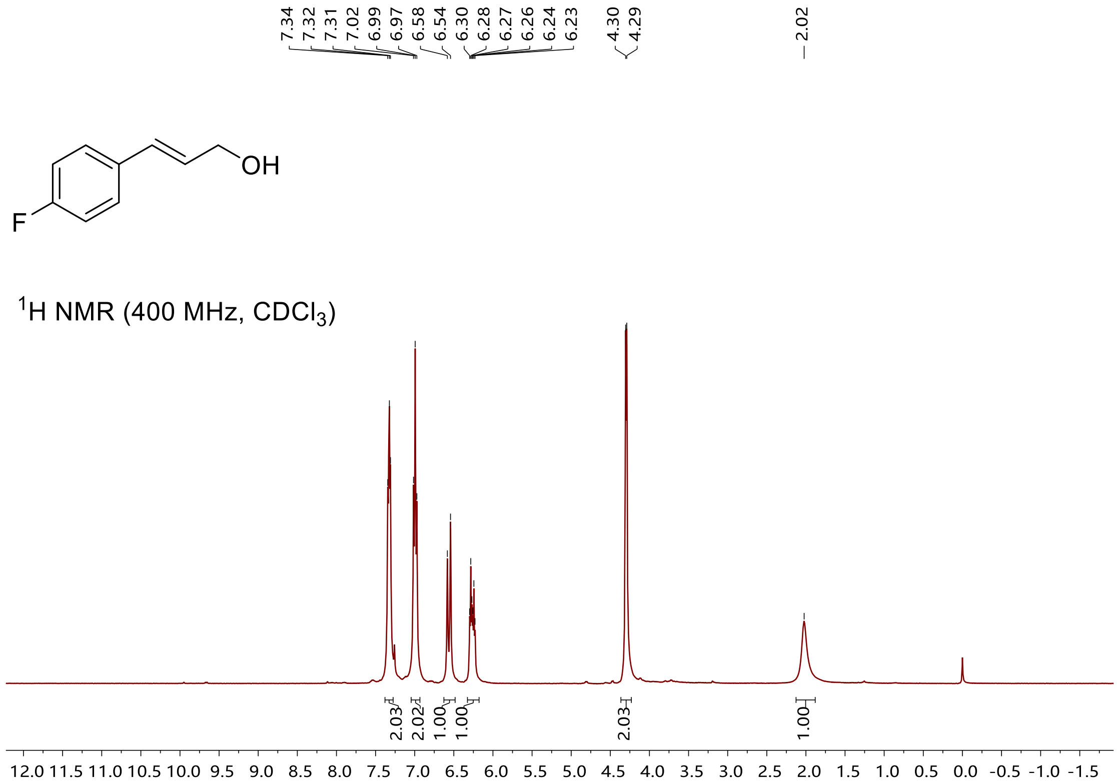


**Supplementary Fig. 21.** $^{1}H$ NMR spectrum of compound **5f**.

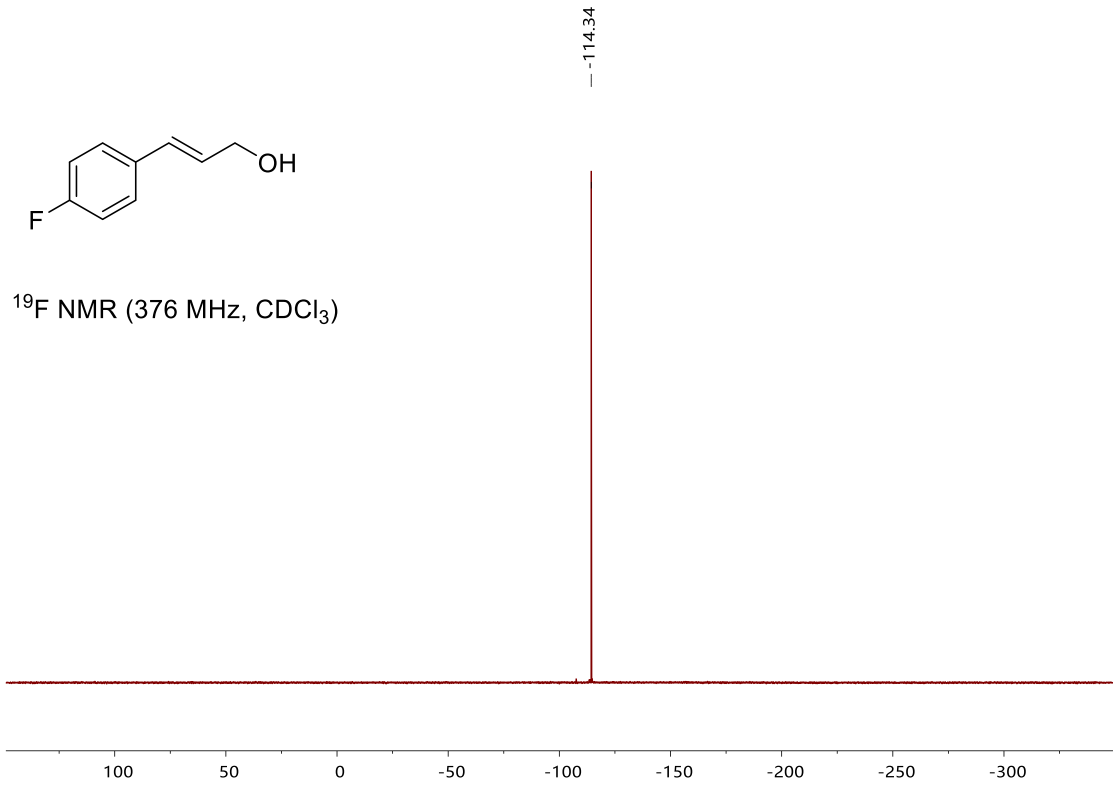


**Supplementary Fig. 22.** $^{19}F$ NMR spectrum of compound **5f**.

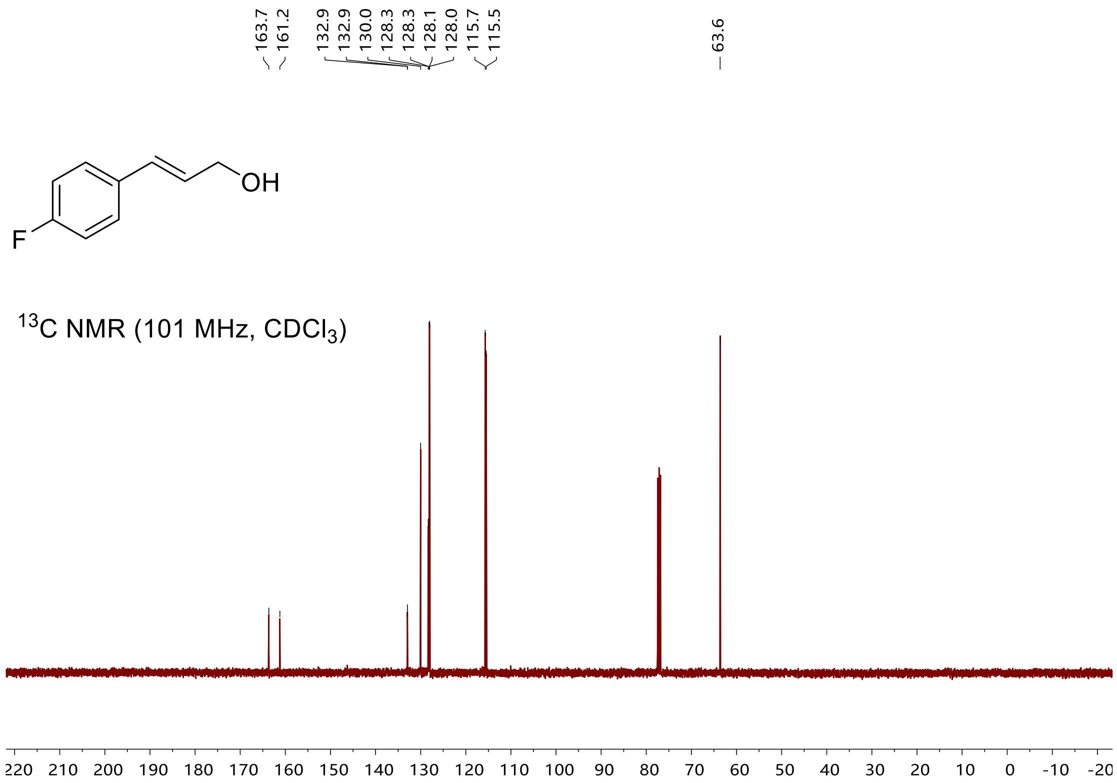


**Supplementary Fig. 23.** $^{13}$C NMR spectrum of compound **5f**.

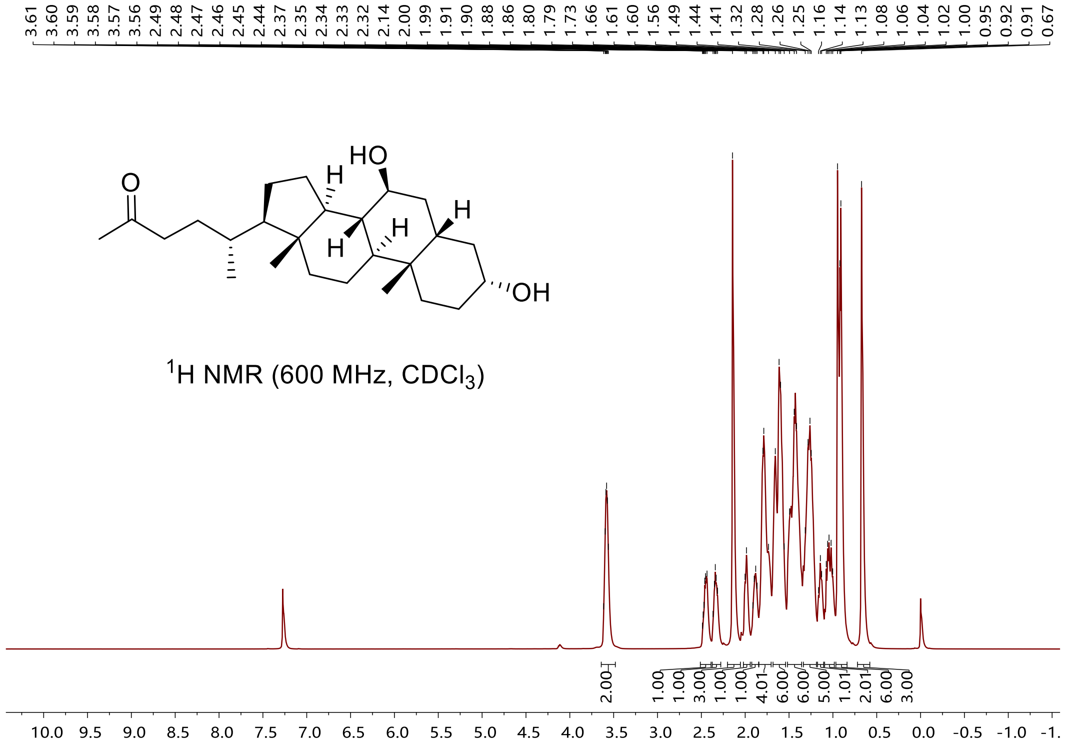


**Supplementary Fig. 24.** $^{1}$H NMR spectrum of compound **1ae**.

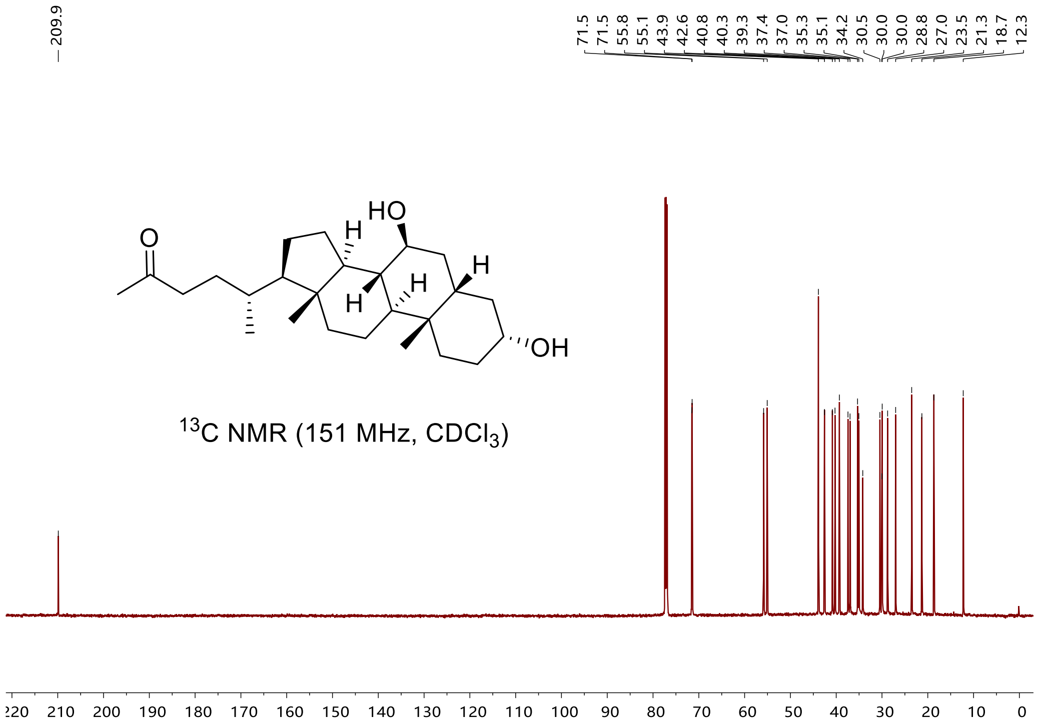


**Supplementary Fig. 25.** $^{13}$C NMR spectrum of compound **1ae**.

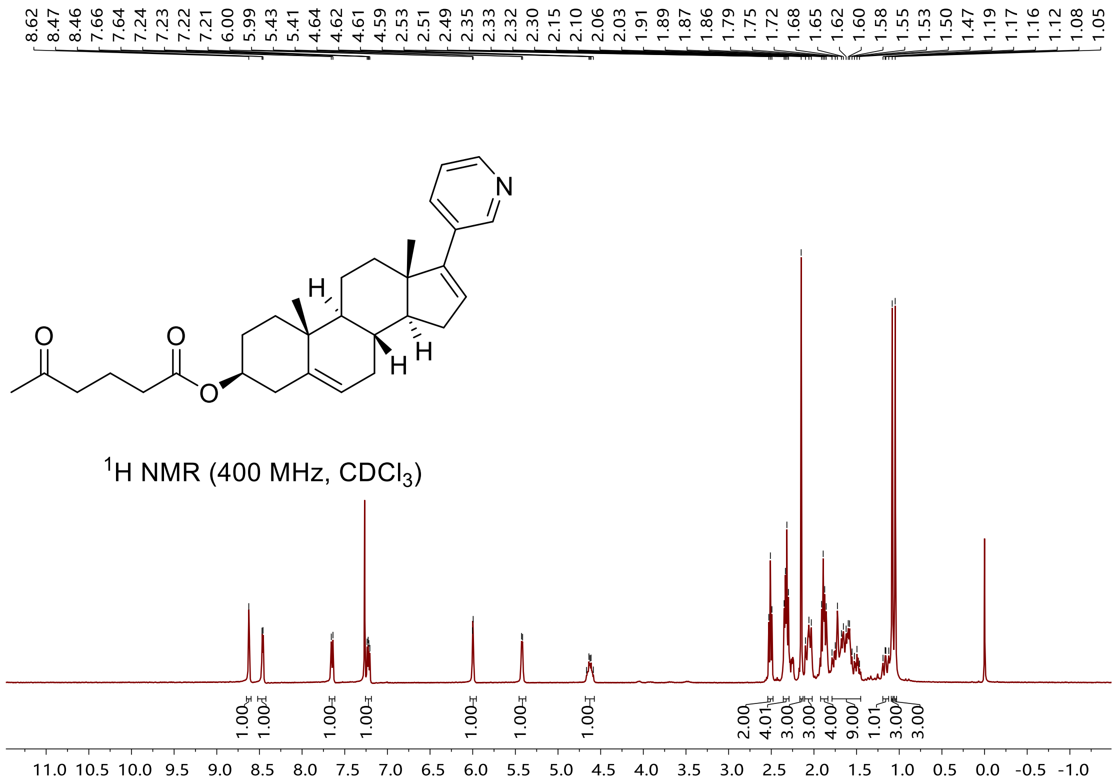


**Supplementary Fig. 26.** $^{1}$H NMR spectrum of compound **1af**.

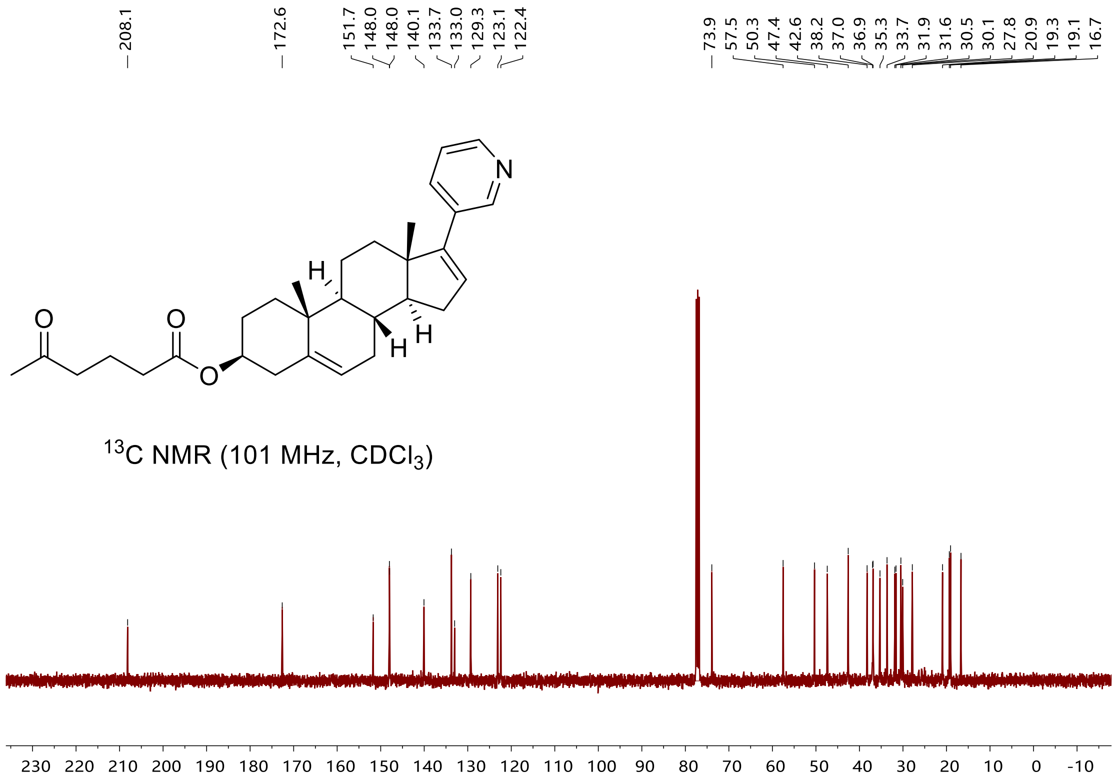


**Supplementary Fig. 27.** $^{13}$C NMR spectrum of compound **1af**.

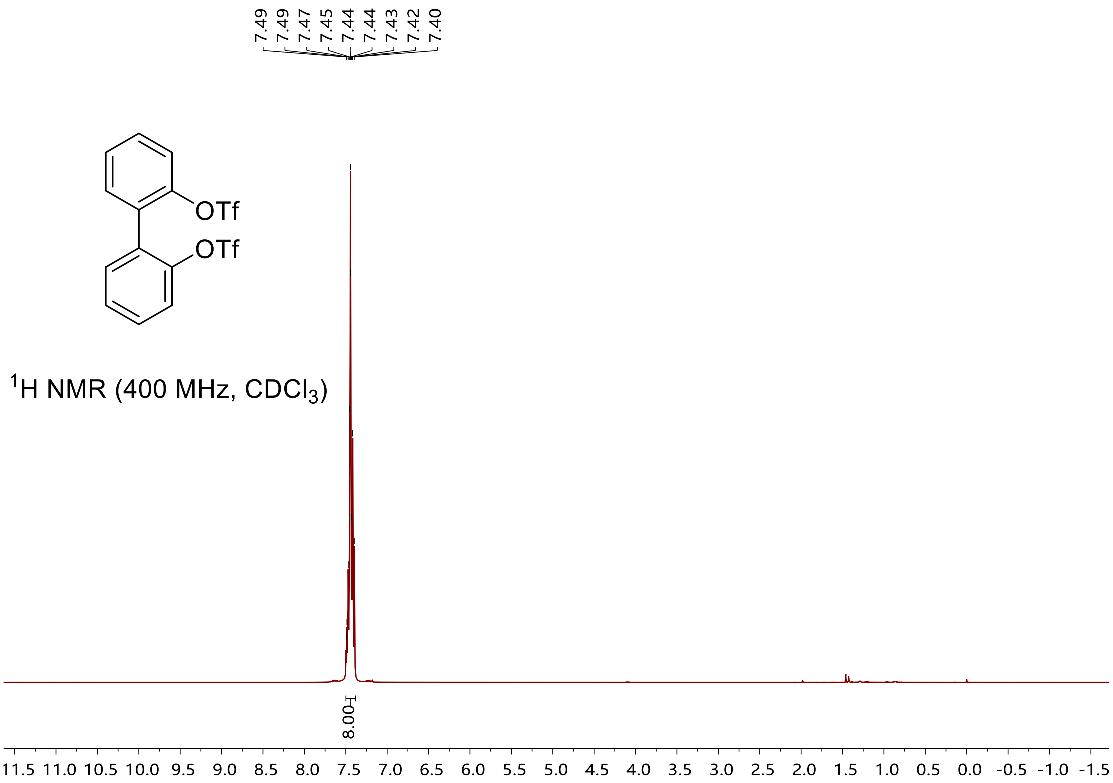


**Supplementary Fig. 28.** $^1$H NMR spectrum of compound **S1**.

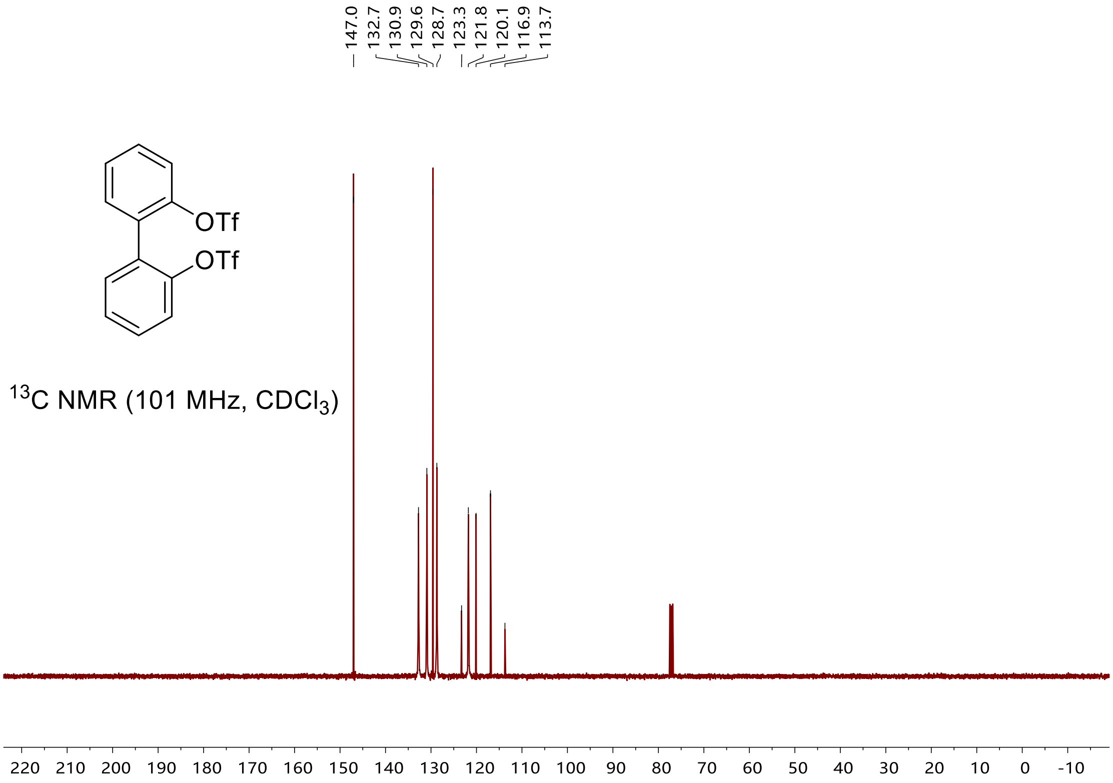


**Supplementary Fig. 29.** $^{13}$C NMR spectrum of compound **S1**.

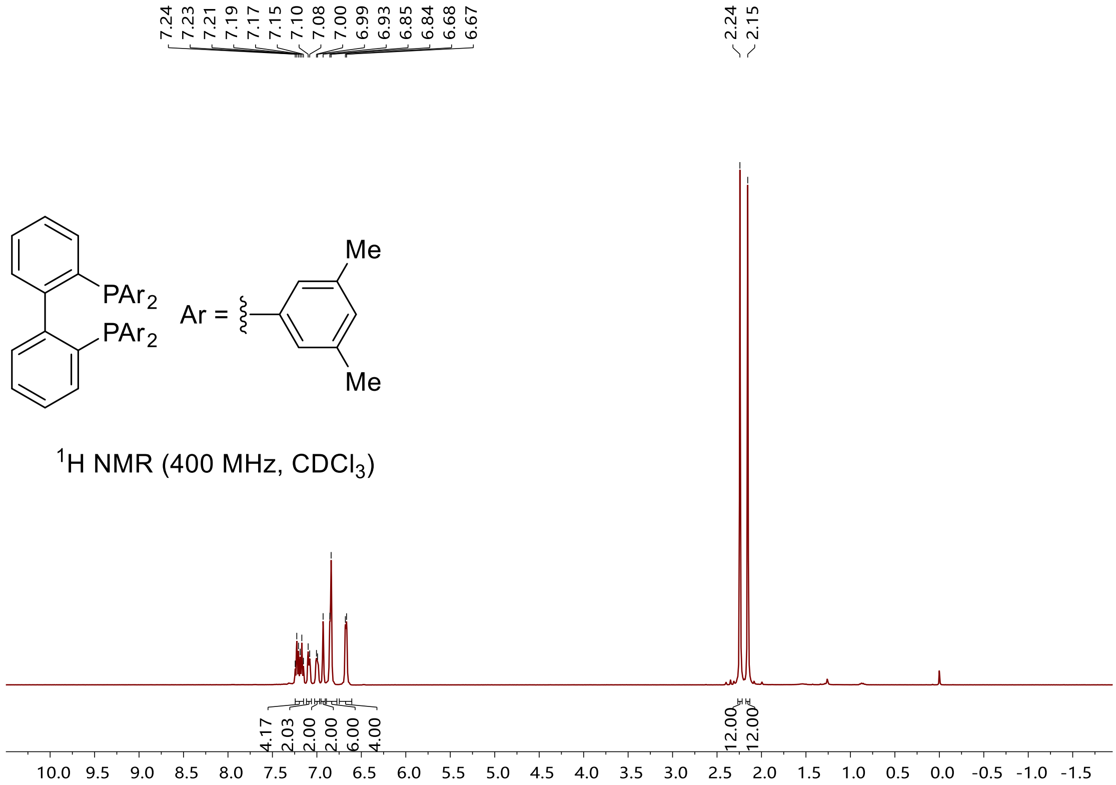


**Supplementary Fig. 30.** $^{1}$H NMR spectrum of compound **L4**.

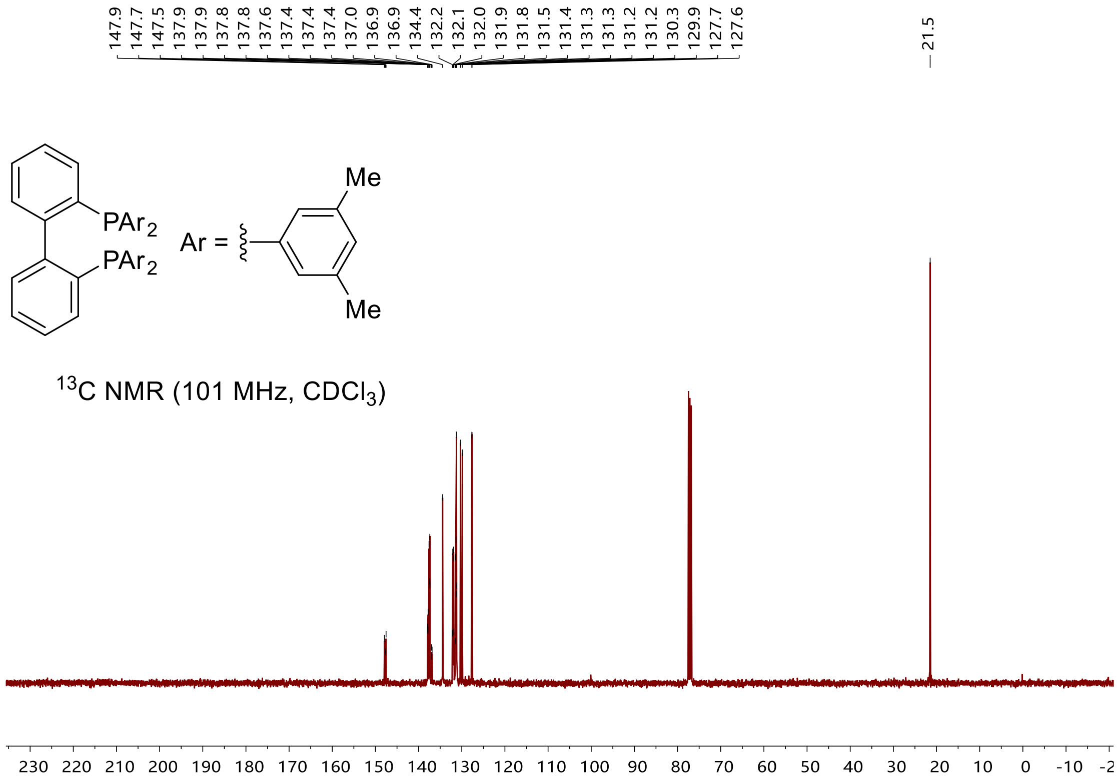


**Supplementary Fig. 31.** $^{13}$C NMR spectrum of compound **L4**.

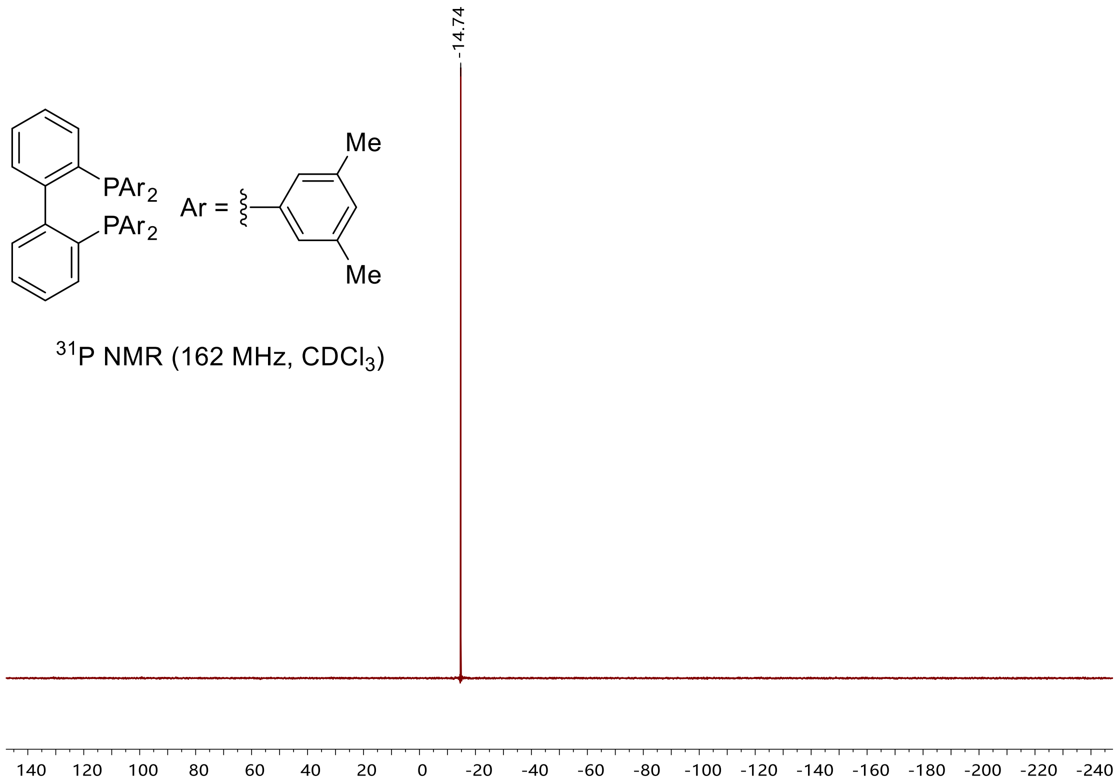


**Supplementary Fig. 32.** $^{31}$P NMR spectrum of compound **L4**.

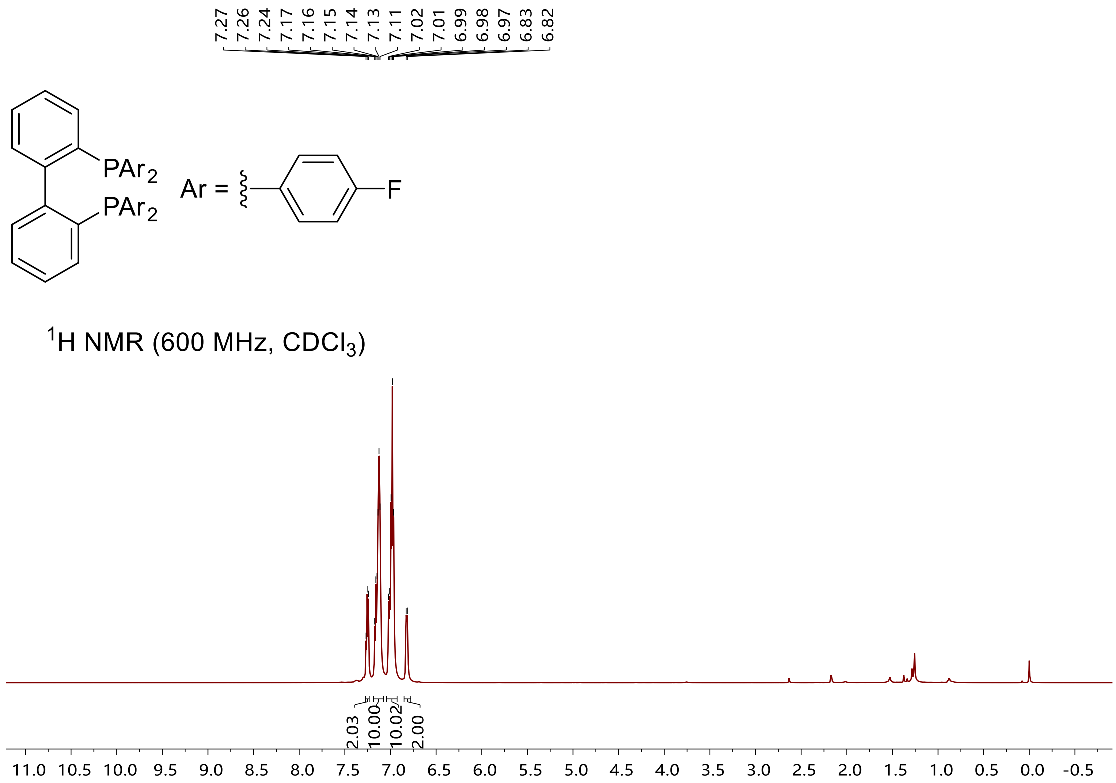


**Supplementary Fig. 33.** $^{1}$H NMR spectrum of compound **L2**.

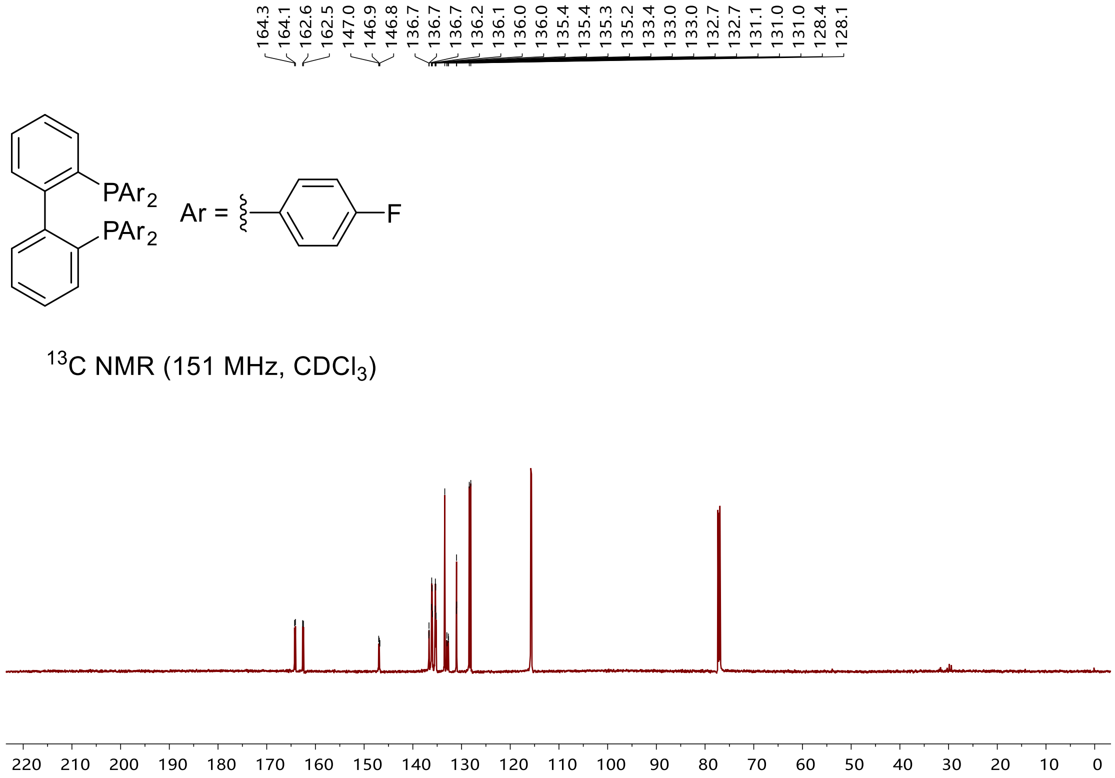


**Supplementary Fig. 34.** $^{13}$C NMR spectrum of compound **L2**.

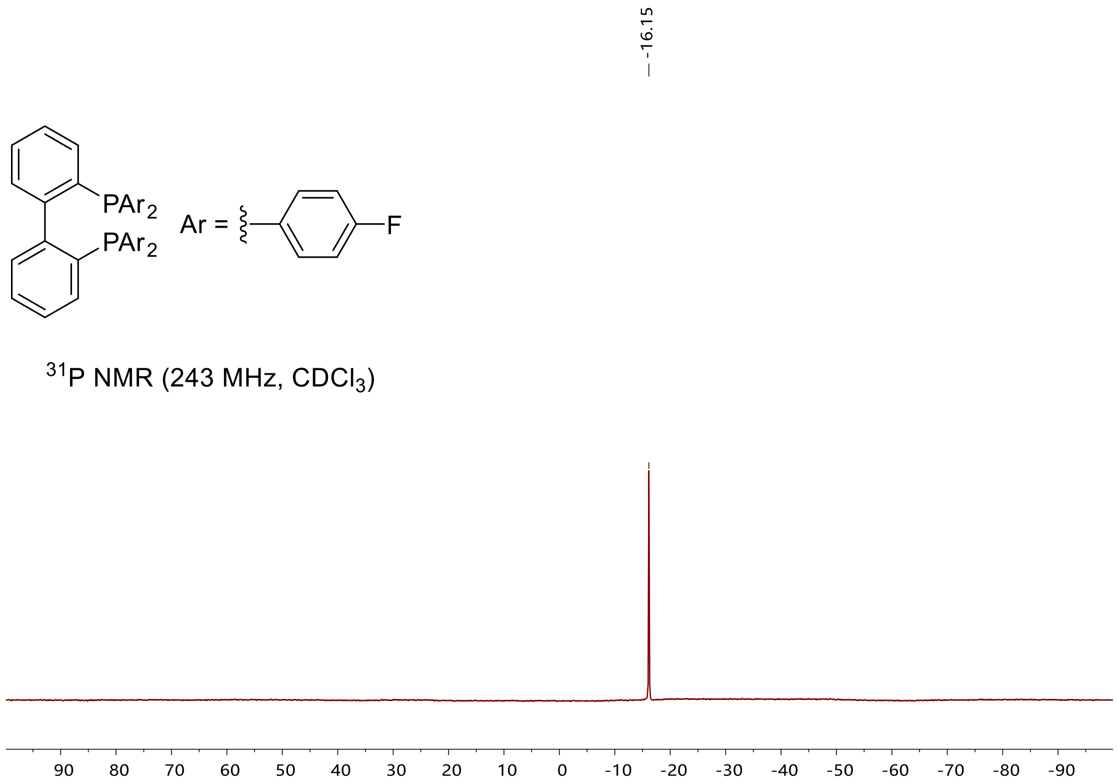


**Supplementary Fig. 35.** $^{31}$P NMR spectrum of compound **L2**.

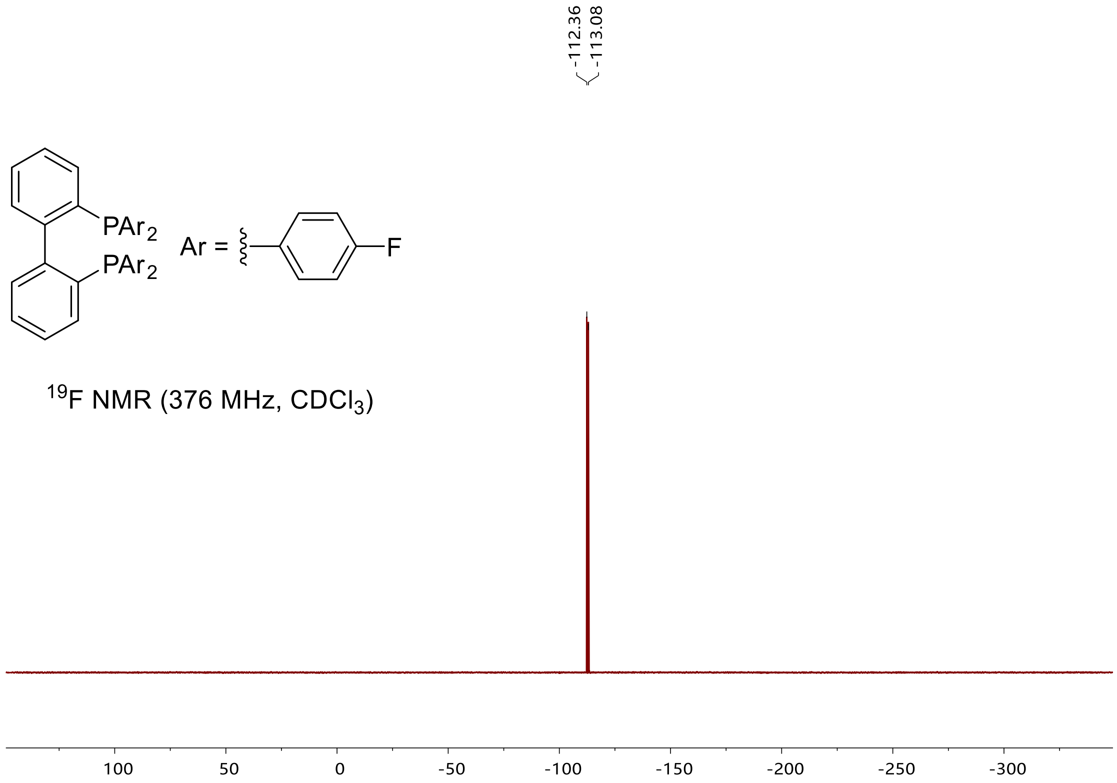


**Supplementary Fig. 36.** $^{19}$F NMR spectrum of compound **L2**.

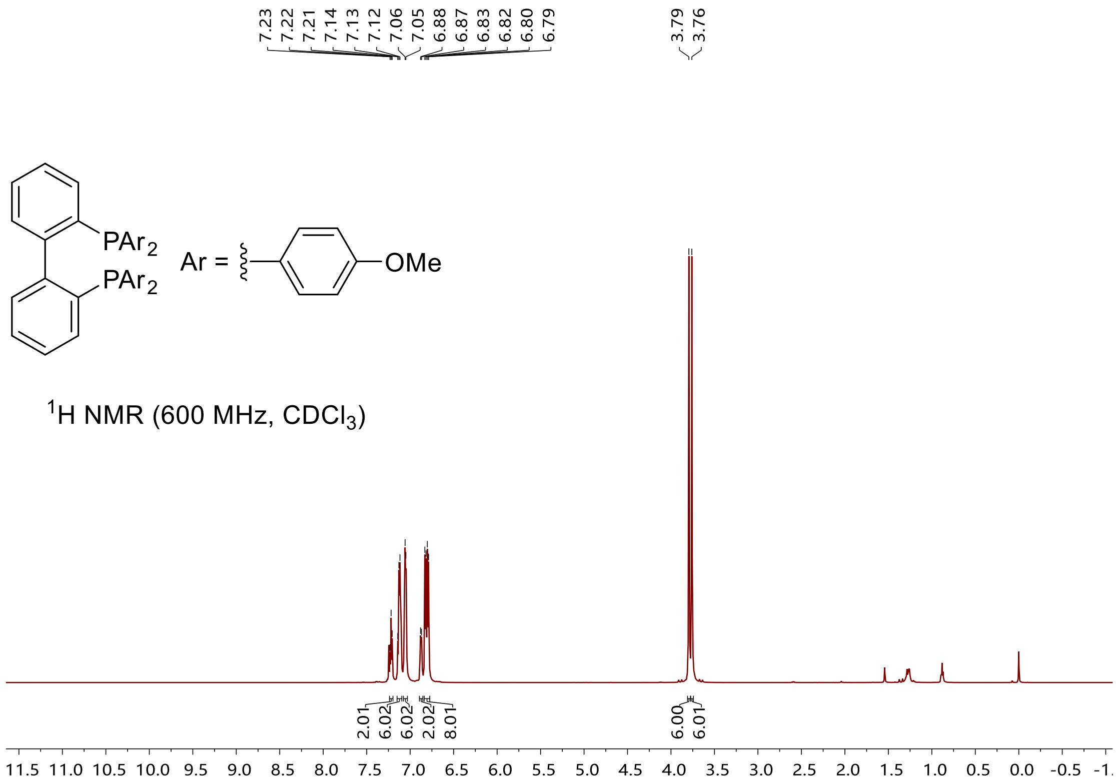


**Supplementary Fig. 37.** $^{1}H$ NMR spectrum of compound **L3**.

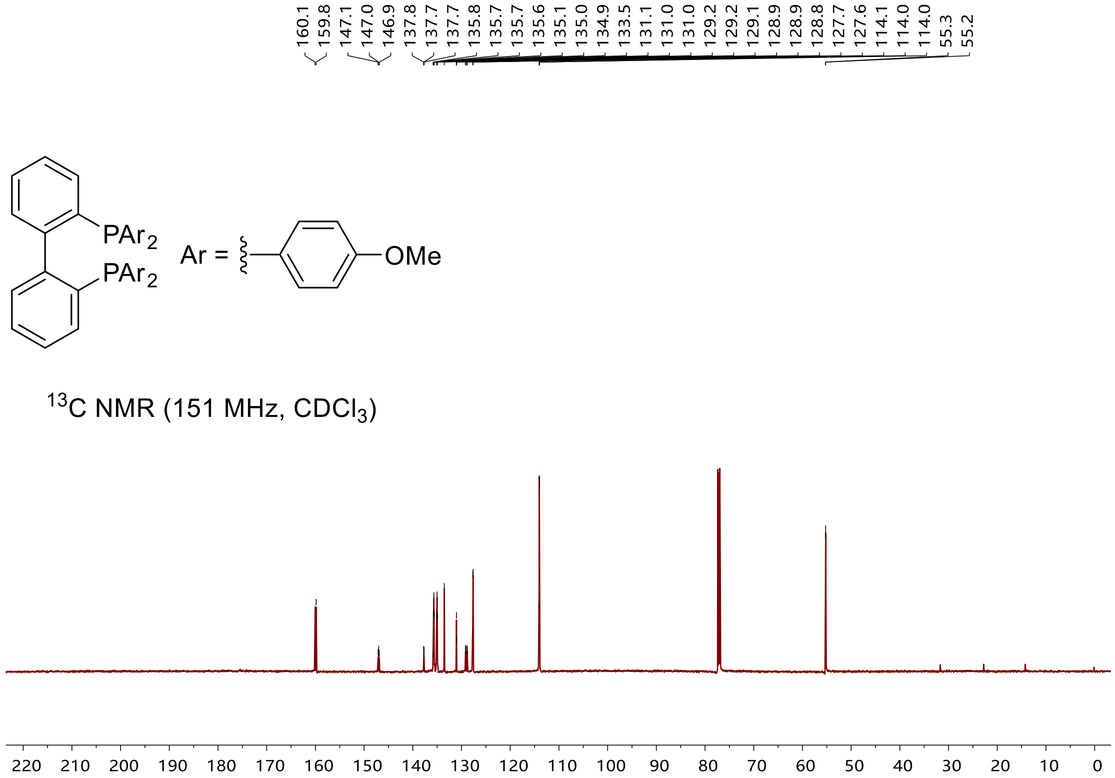


**Supplementary Fig. 38.** $^{13}C$ NMR spectrum of compound **L3**.

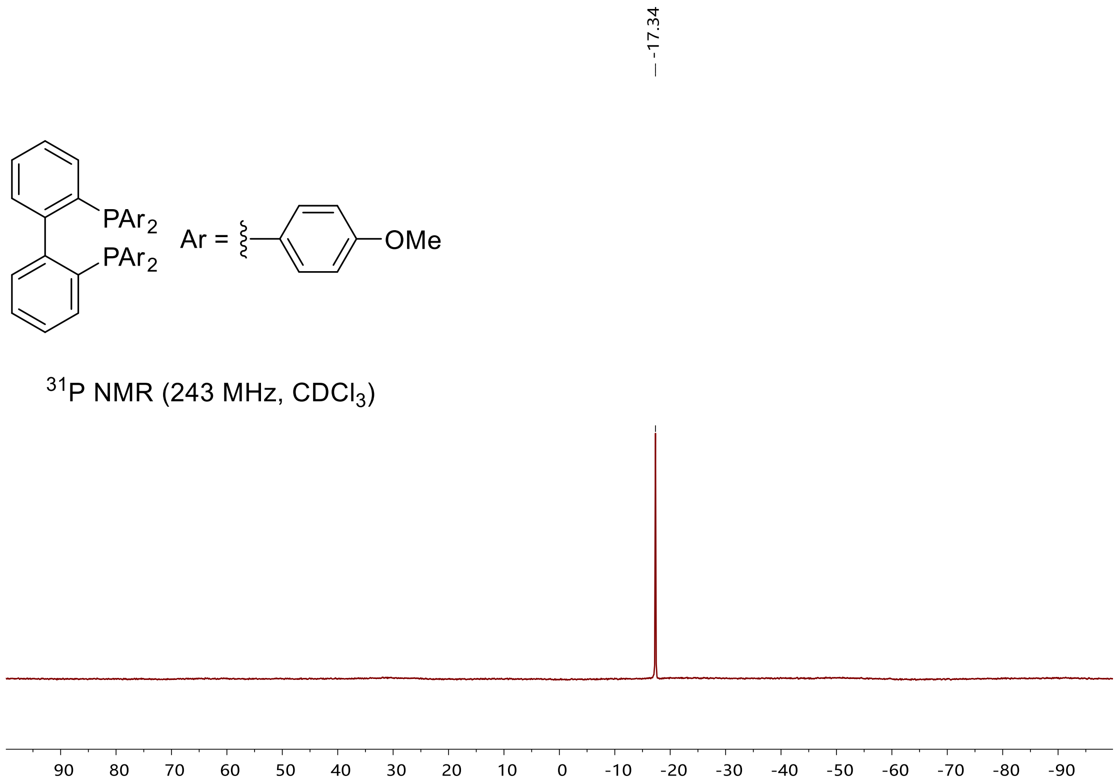


**Supplementary Fig. 39.** $^{31}$P NMR spectrum of compound **L3**.

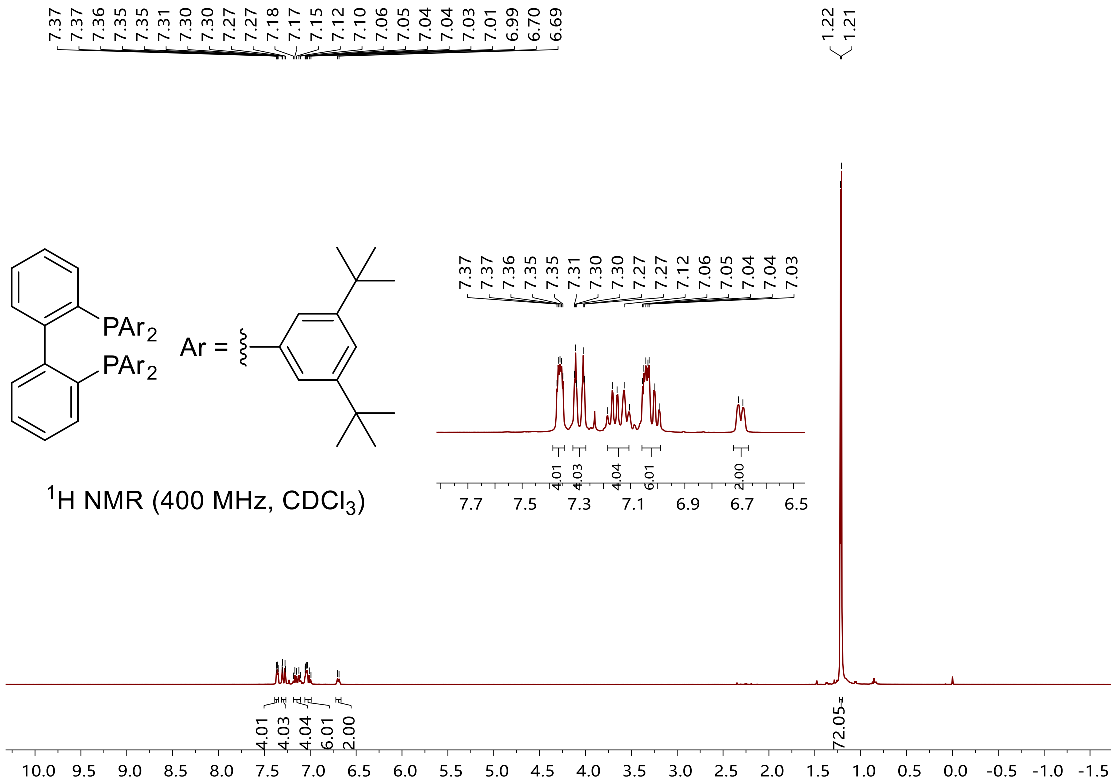


**Supplementary Fig. 40.** $^{1}H$ NMR spectrum of compound **L5**.

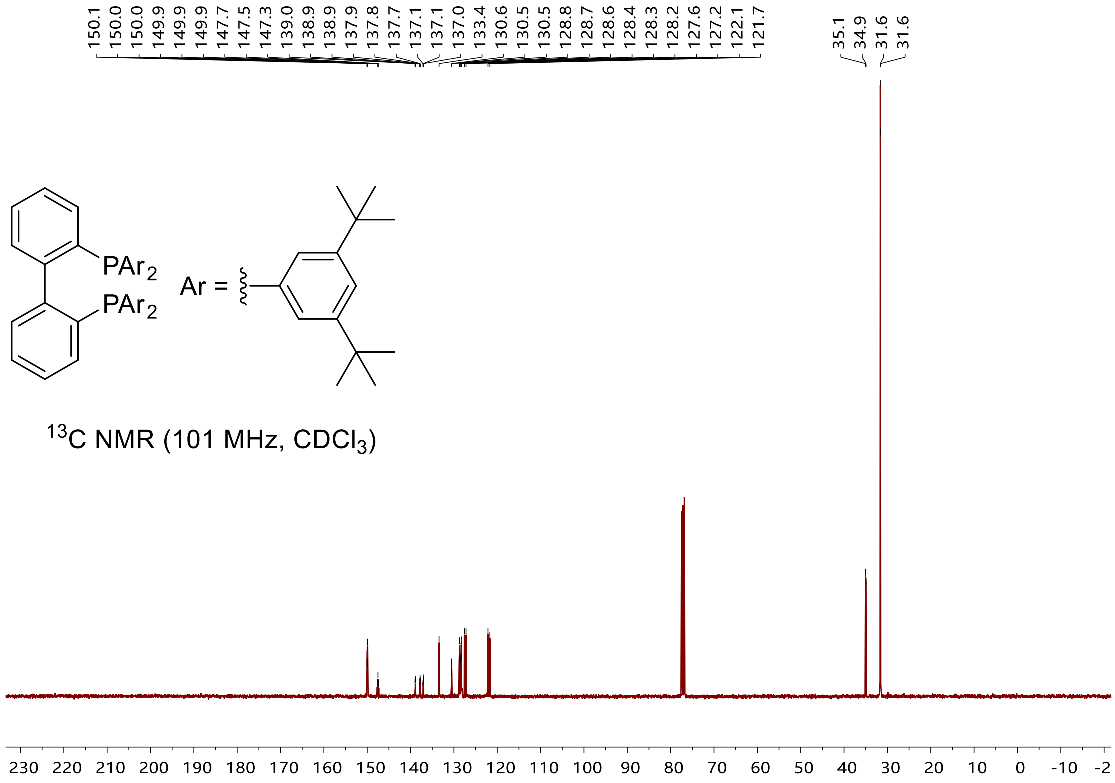


**Supplementary Fig. 41.** $^{13}C$ NMR spectrum of compound **L5**.

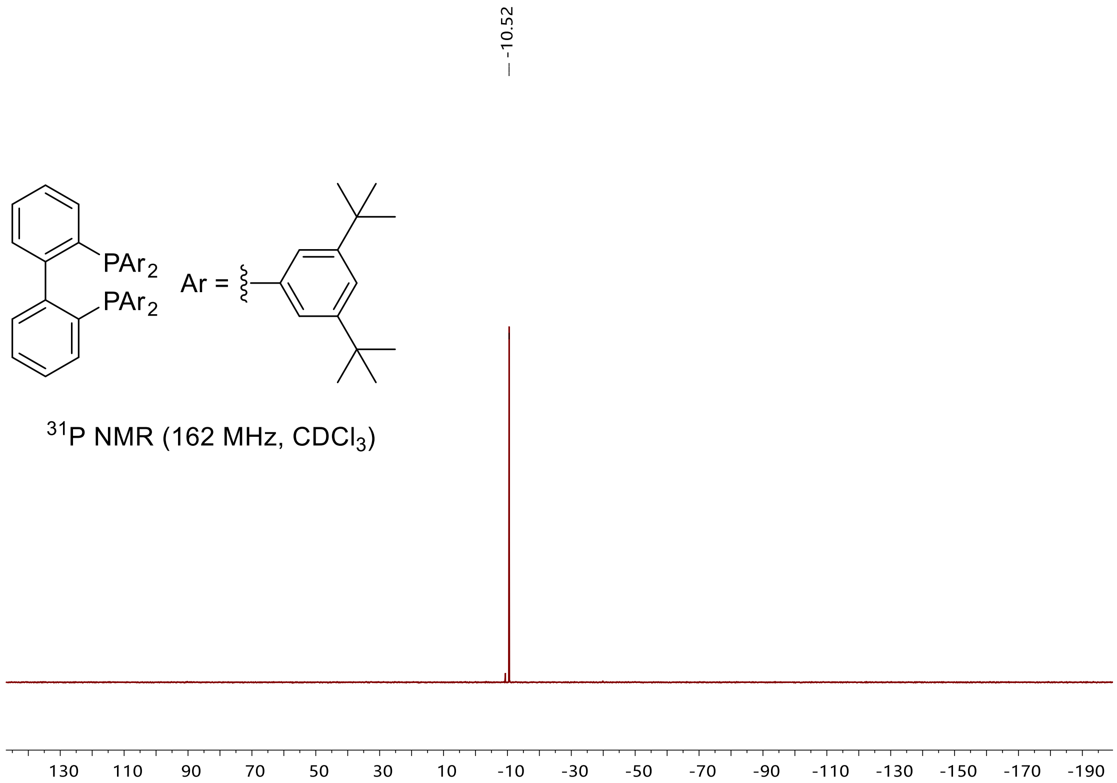


**Supplementary Fig. 42.** $^{31}$P NMR spectrum of compound **L5**.

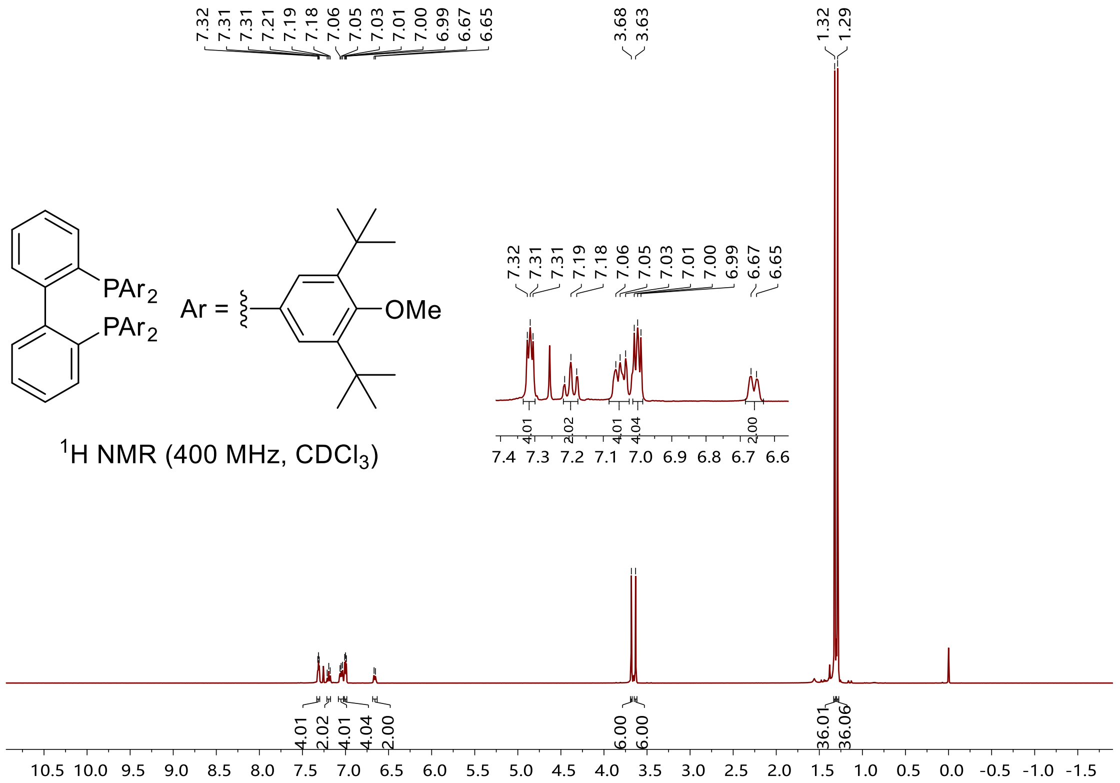


**Supplementary Fig. 43.** $^{1}$H NMR spectrum of compound **L6**.

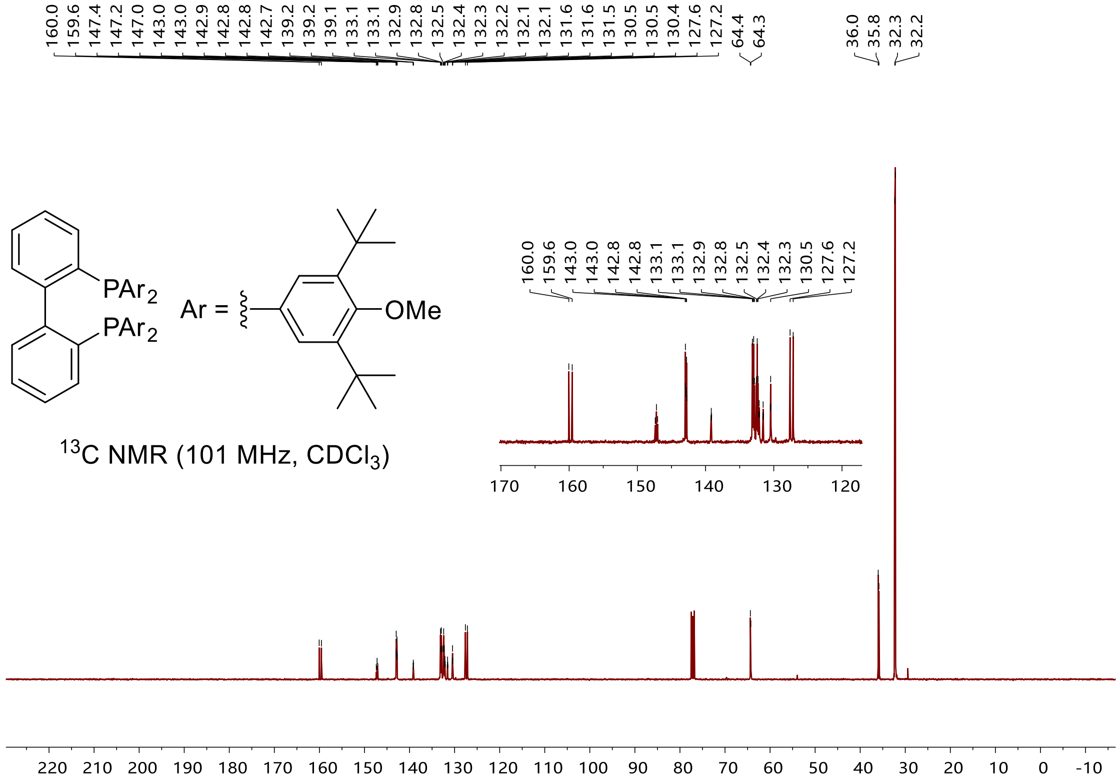


**Supplementary Fig. 44.** $^{13}$C NMR spectrum of compound **L6**.

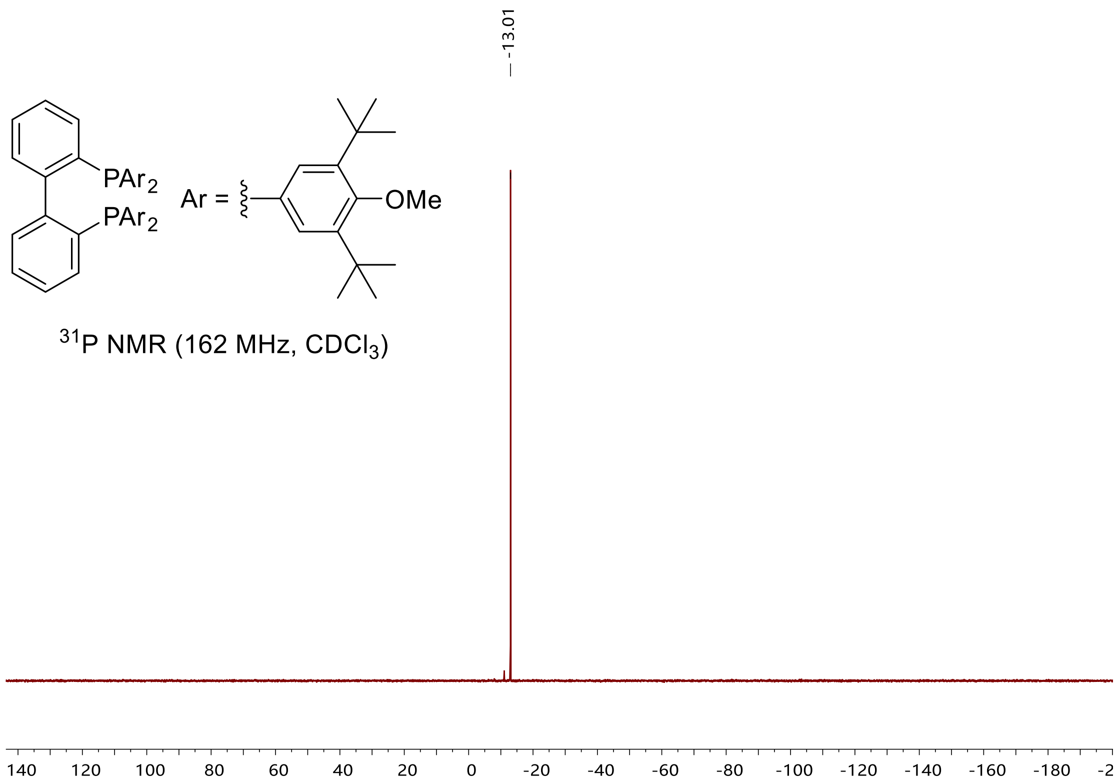


**Supplementary Fig. 45.** $^{31}$P NMR spectrum of compound **L6**.

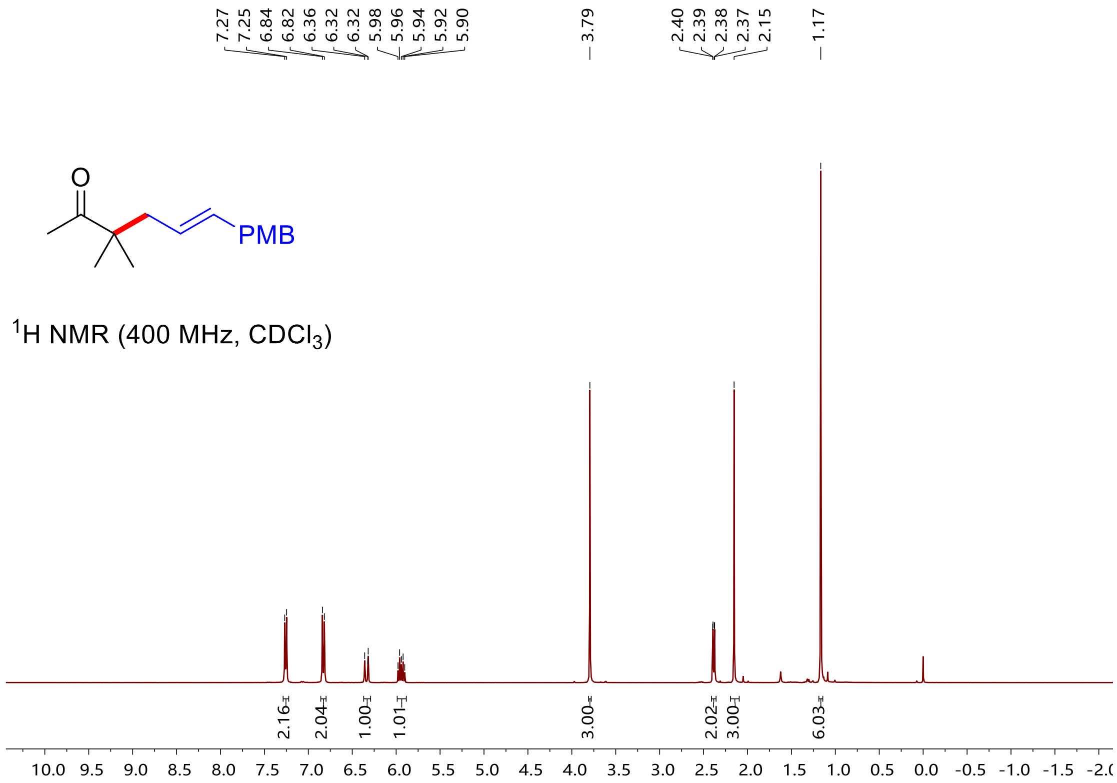


**Supplementary Fig. 46.** $^1$H NMR spectrum of compound **3a**.

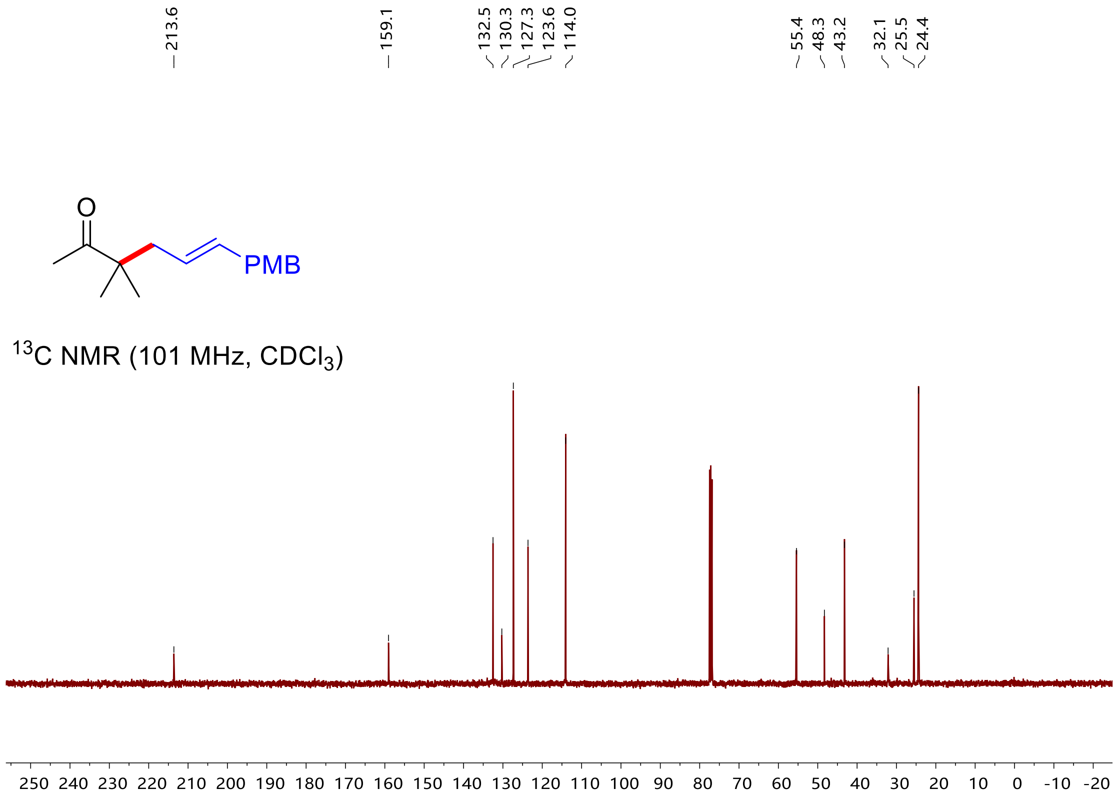


**Supplementary Fig. 47.** $^{13}$C NMR spectrum of compound **3a**.

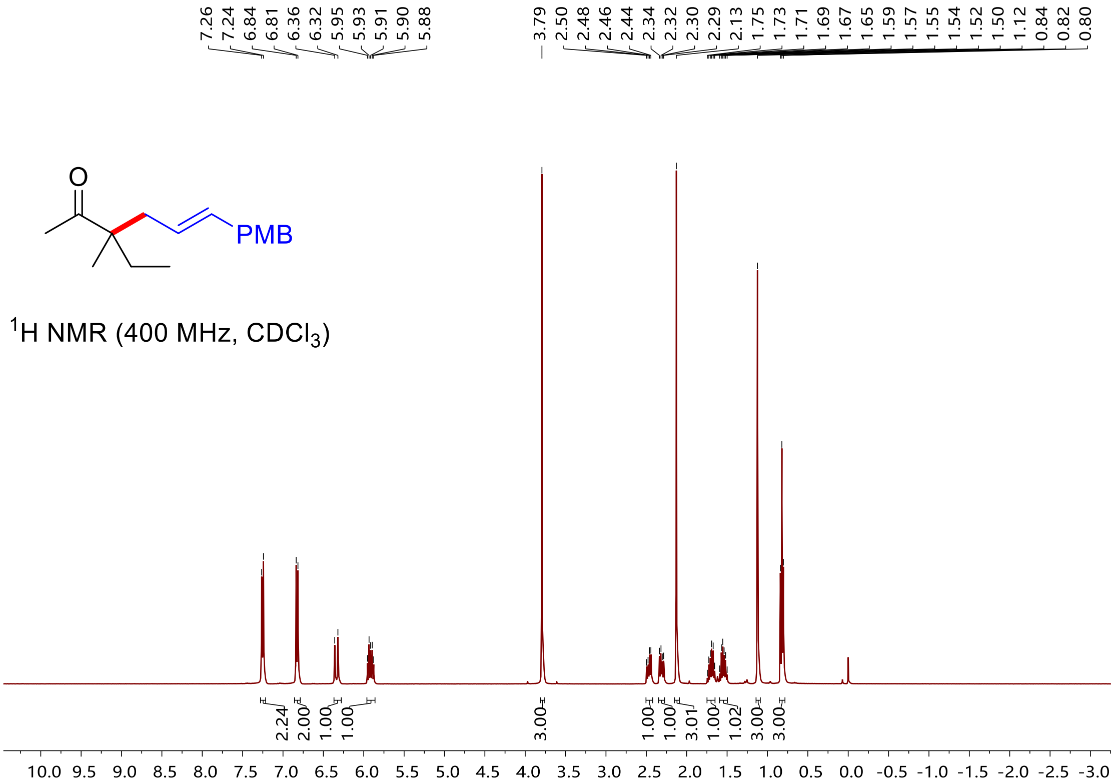


**Supplementary Fig. 48.** $^{1}H$ NMR spectrum of compound **3b**.

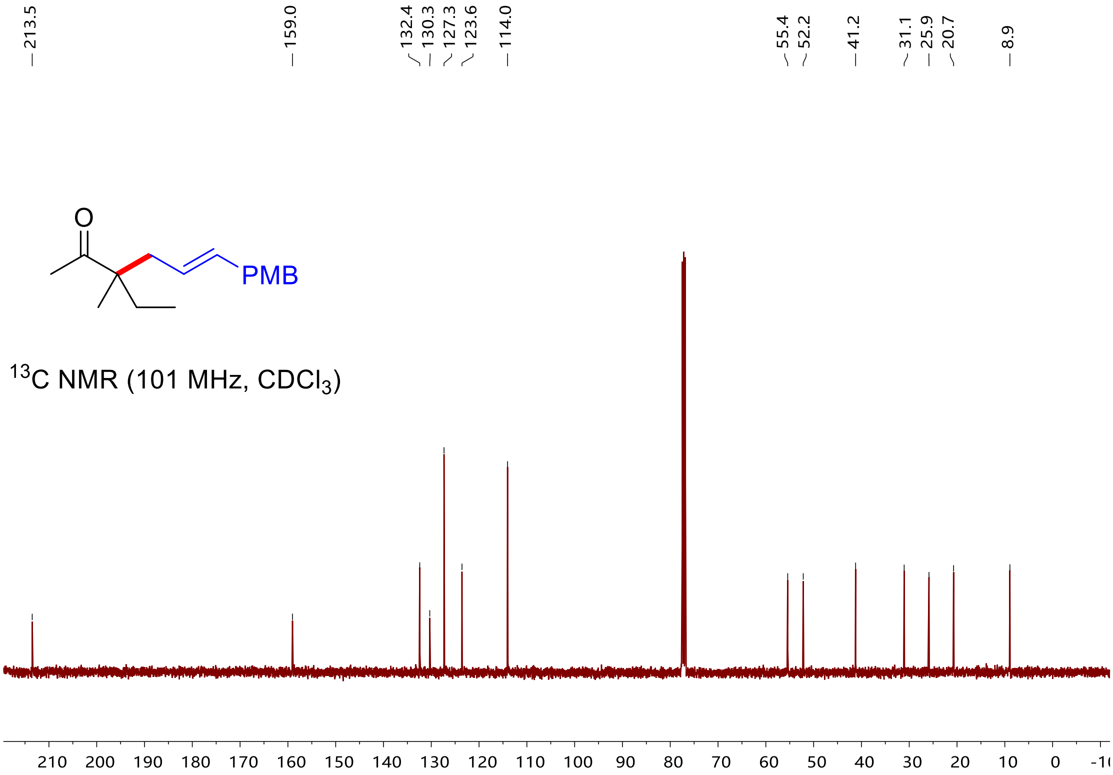


**Supplementary Fig. 49.** $^{13}C$ NMR spectrum of compound **3b**.

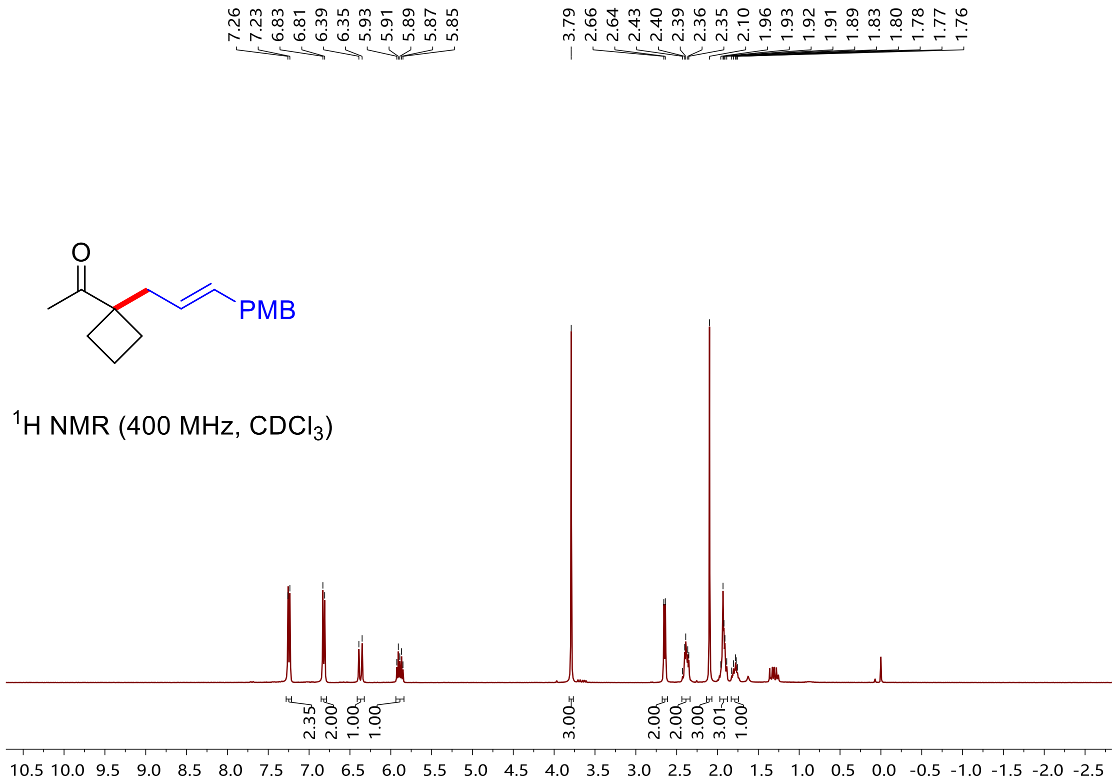


**Supplementary Fig. 50.** $^{1}$H NMR spectrum of compound **3c**.

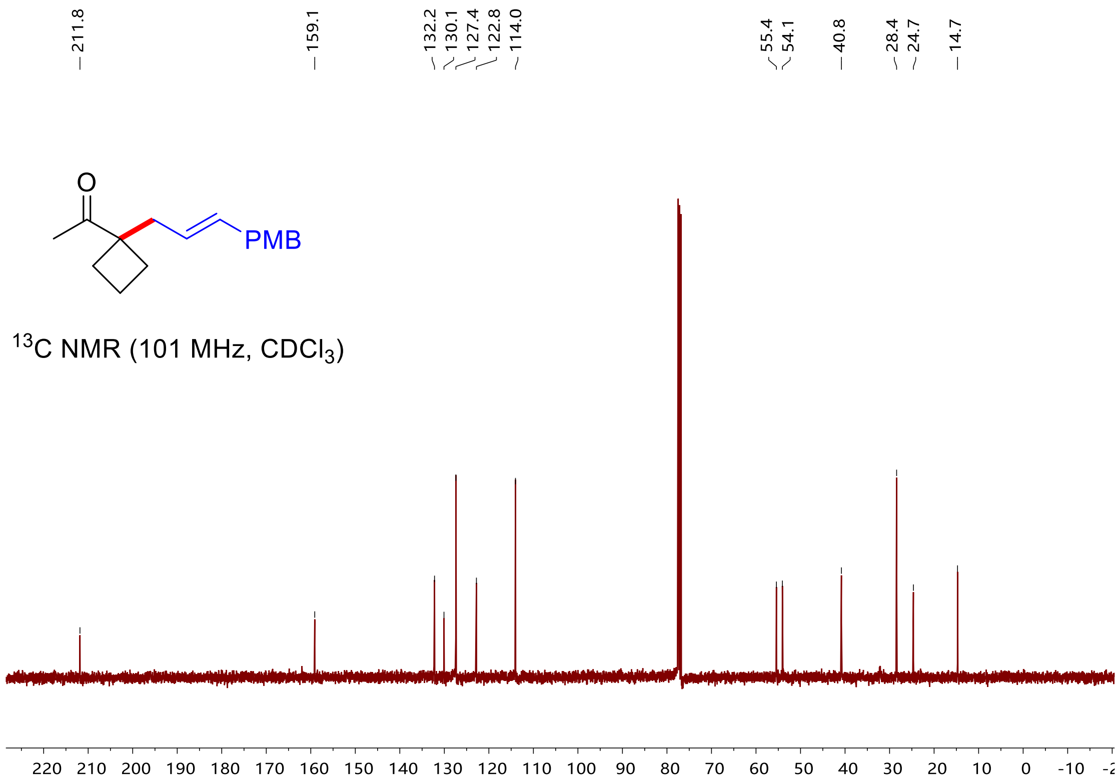


**Supplementary Fig. 51.** $^{13}$C NMR spectrum of compound **3c**.

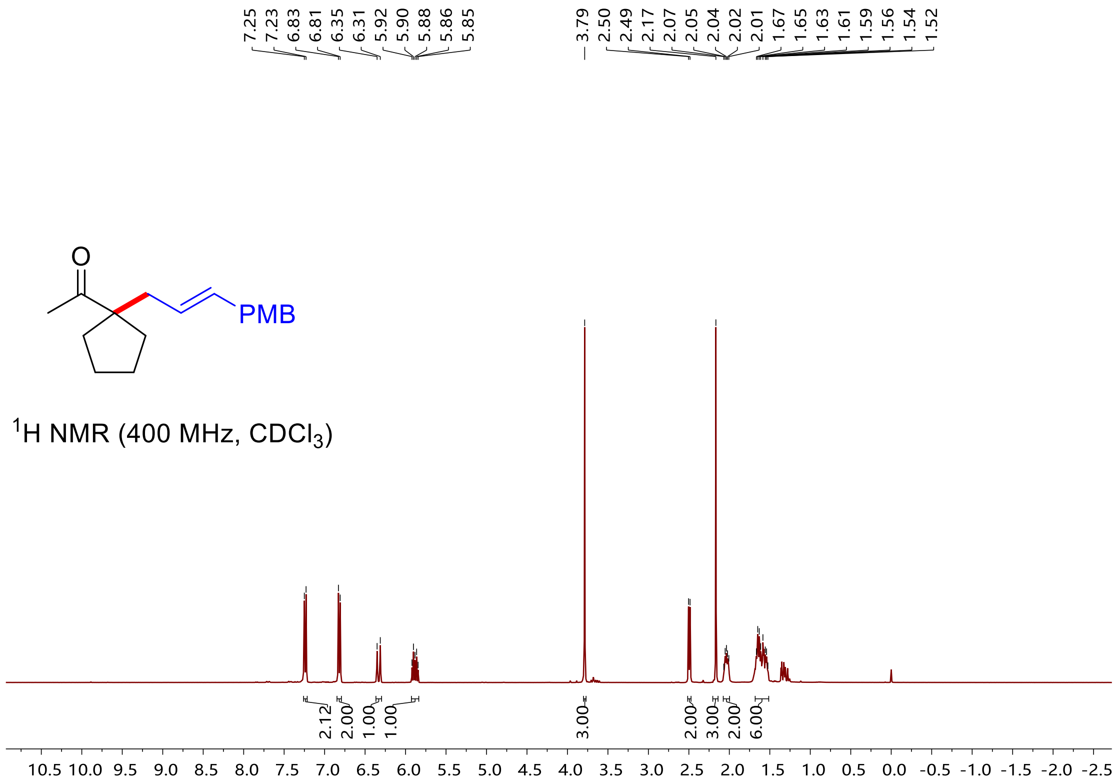


**Supplementary Fig. 52.** $^{1}$H NMR spectrum of compound **3d**.

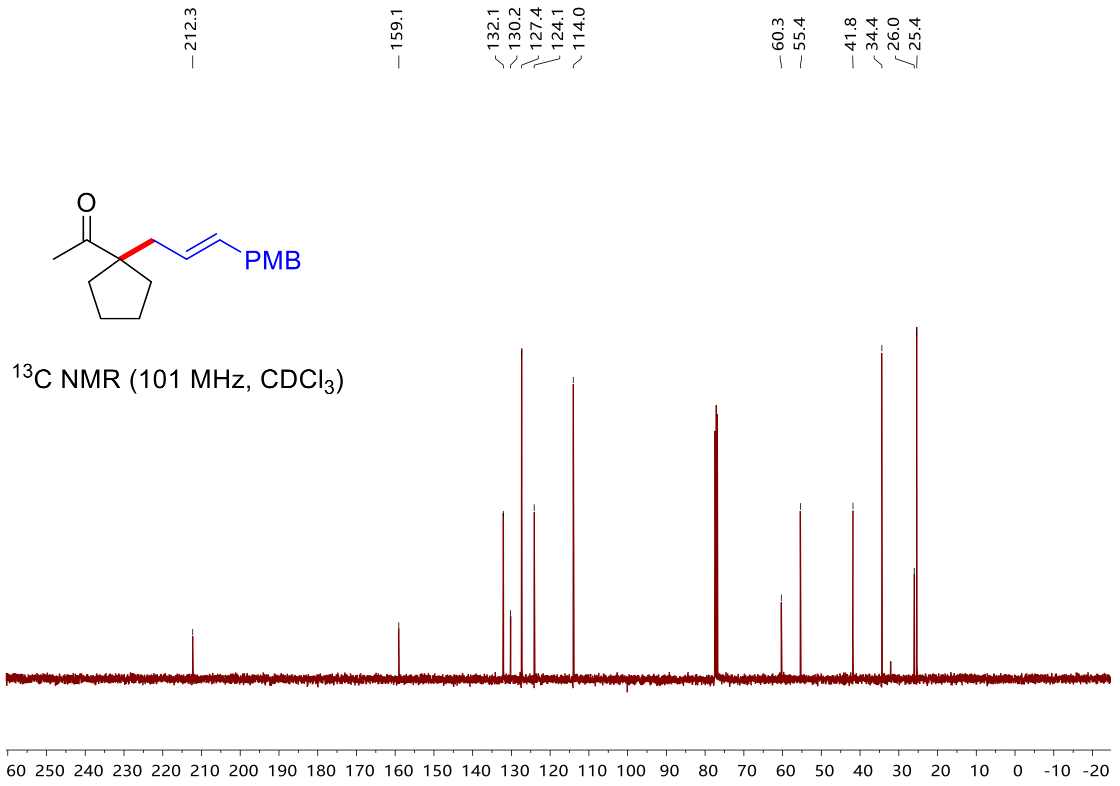


**Supplementary Fig. 53.** $^{13}$C NMR spectrum of compound **3d**.

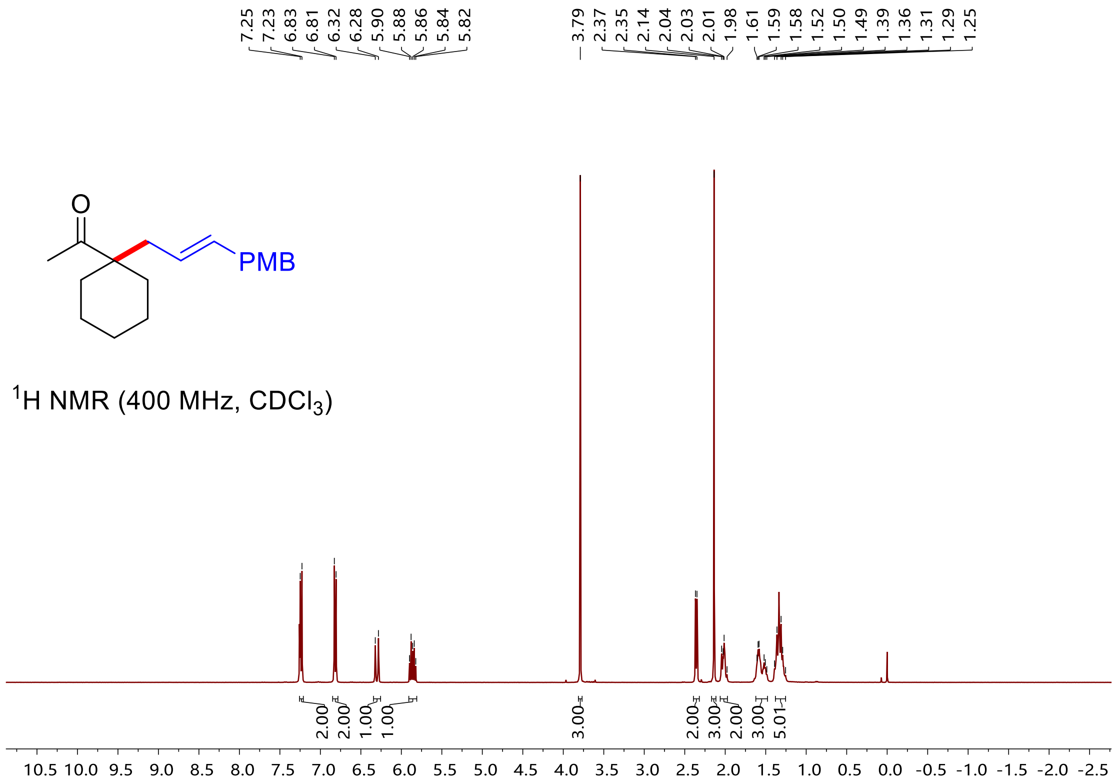


**Supplementary Fig. 54.** $^{1}$H NMR spectrum of compound **3e**.

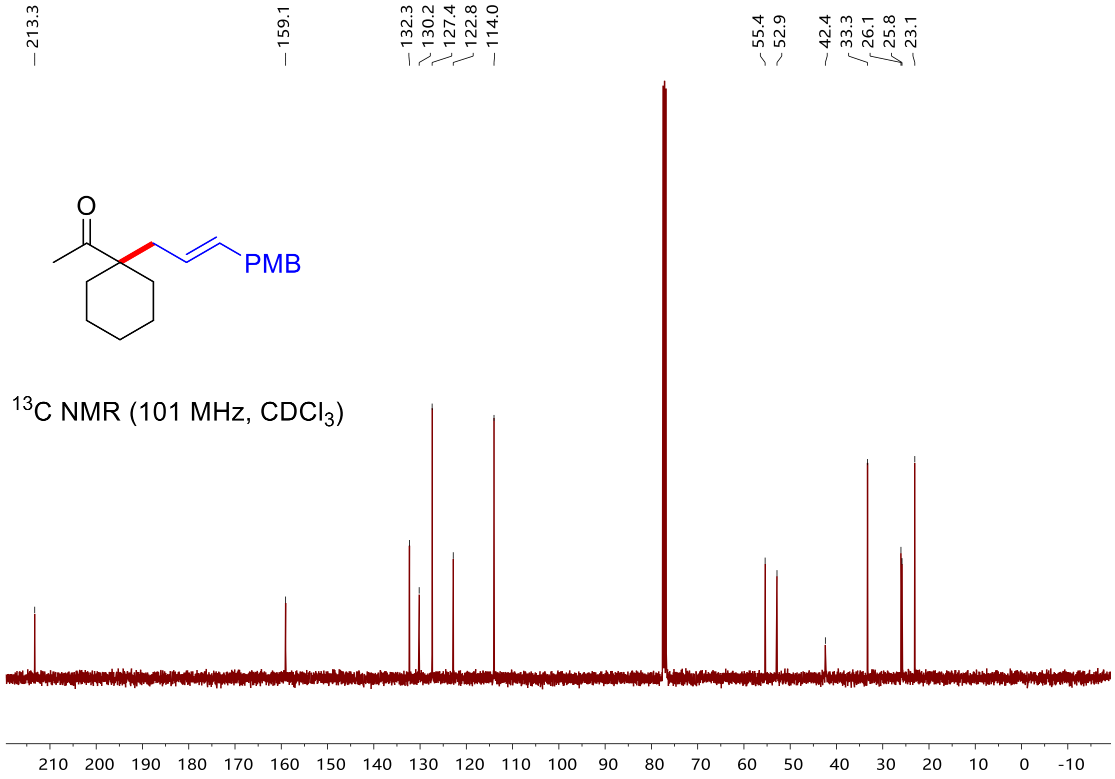


**Supplementary Fig. 55.** $^{13}$C NMR spectrum of compound **3e**.

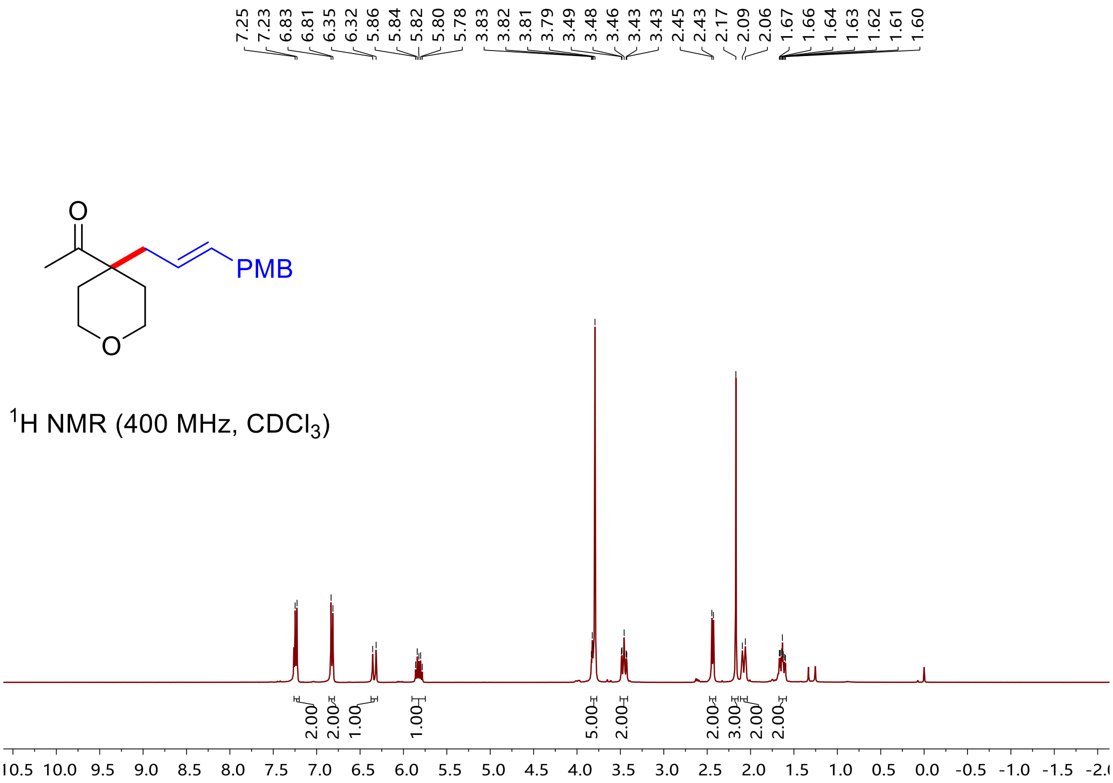


**Supplementary Fig. 56.** $^1$H NMR spectrum of compound **3f**.

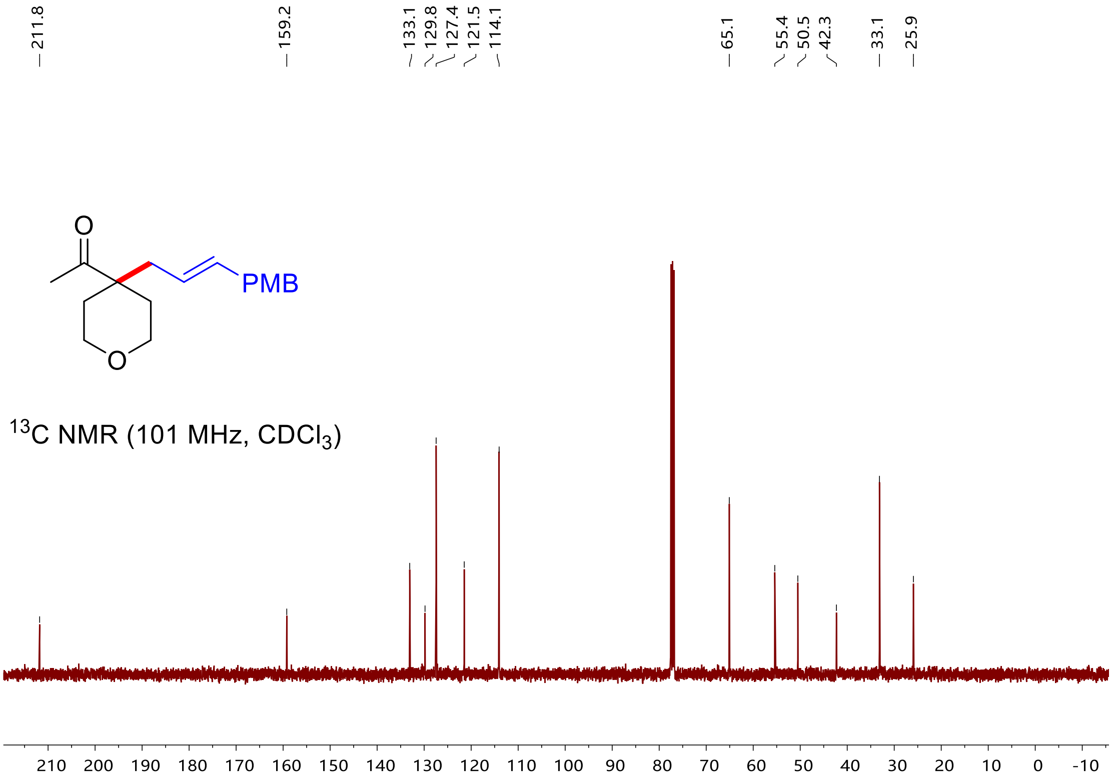


**Supplementary Fig. 57.** $^{13}$C NMR spectrum of compound **3f**.

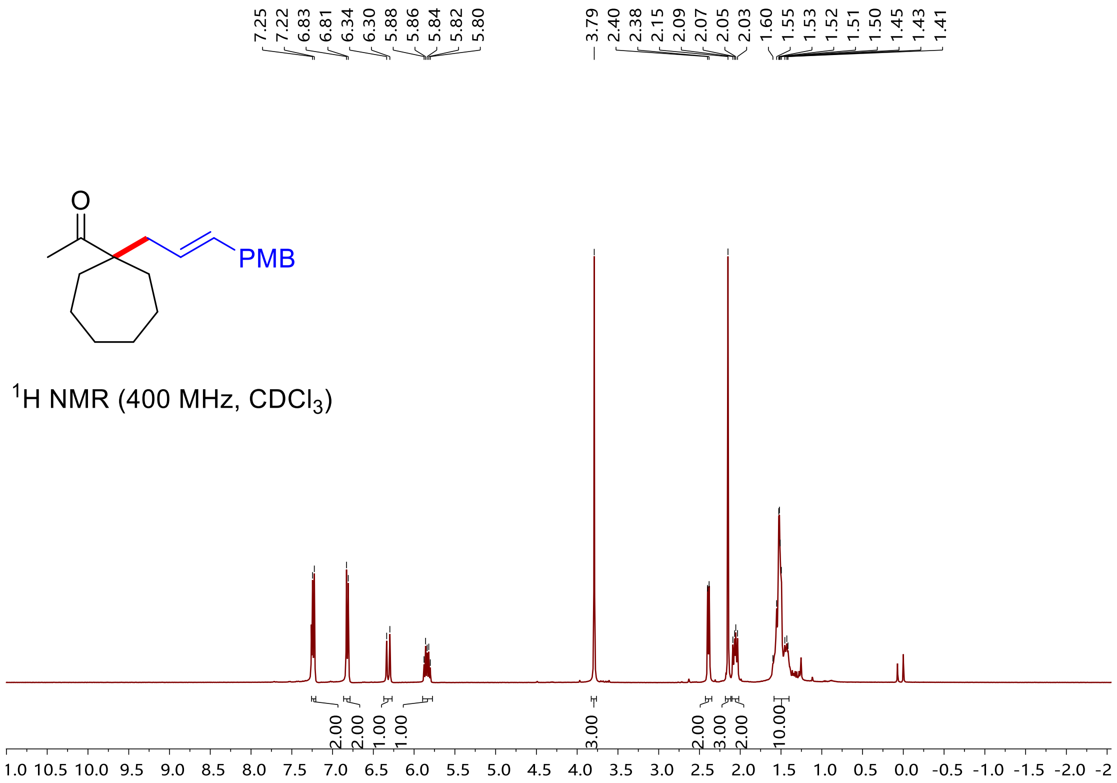


**Supplementary Fig. 58.** $^{1}$H NMR spectrum of compound **3g**.

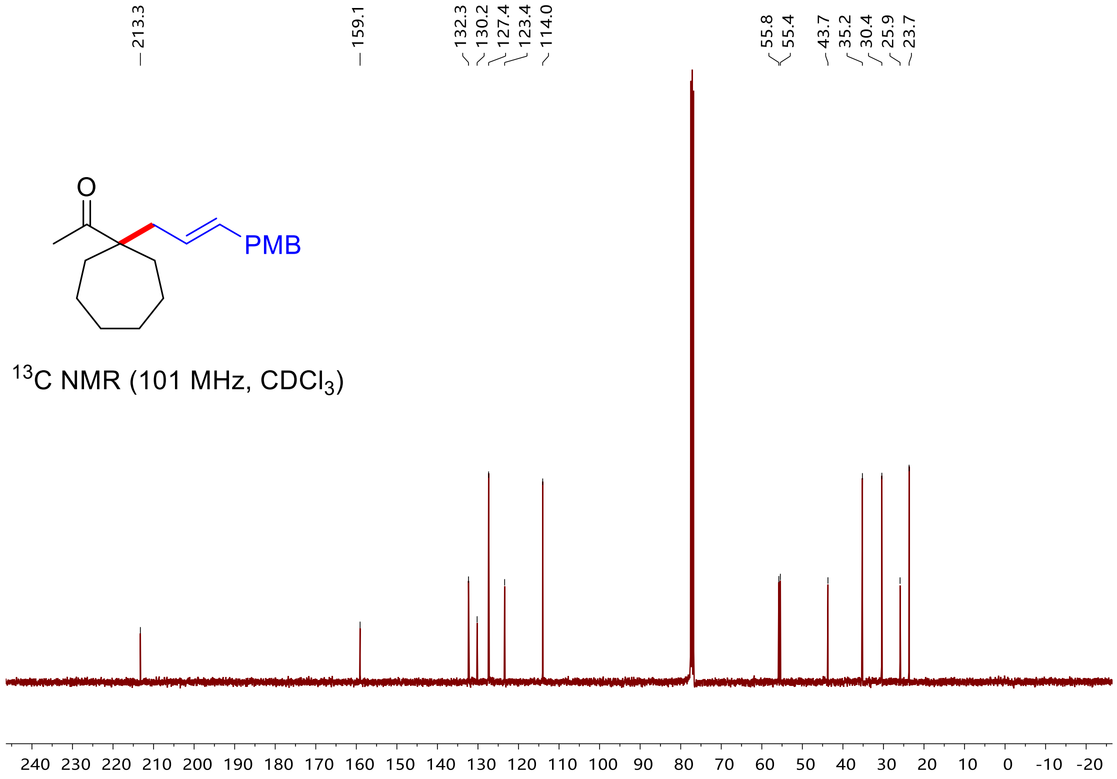


**Supplementary Fig. 59.** $^{13}$C NMR spectrum of compound **3g**.

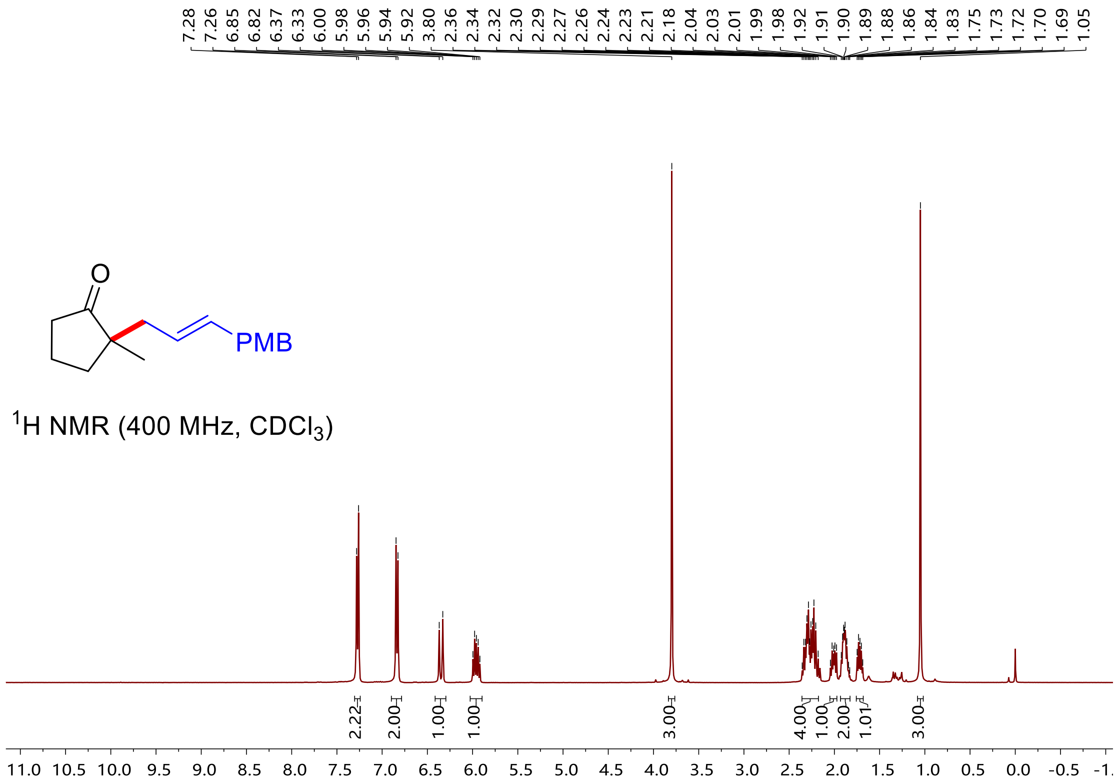


**Supplementary Fig. 60.** $^{1}$H NMR spectrum of compound **3h**.

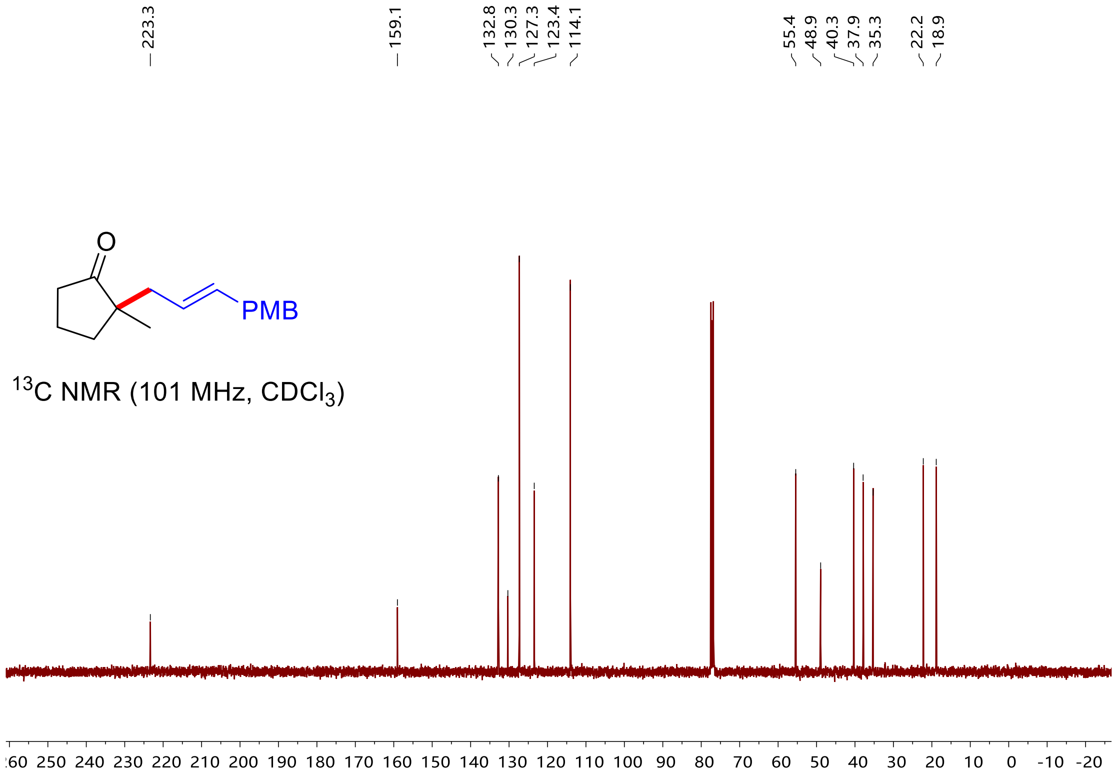


**Supplementary Fig. 61.** $^{13}$C NMR spectrum of compound **3h**.

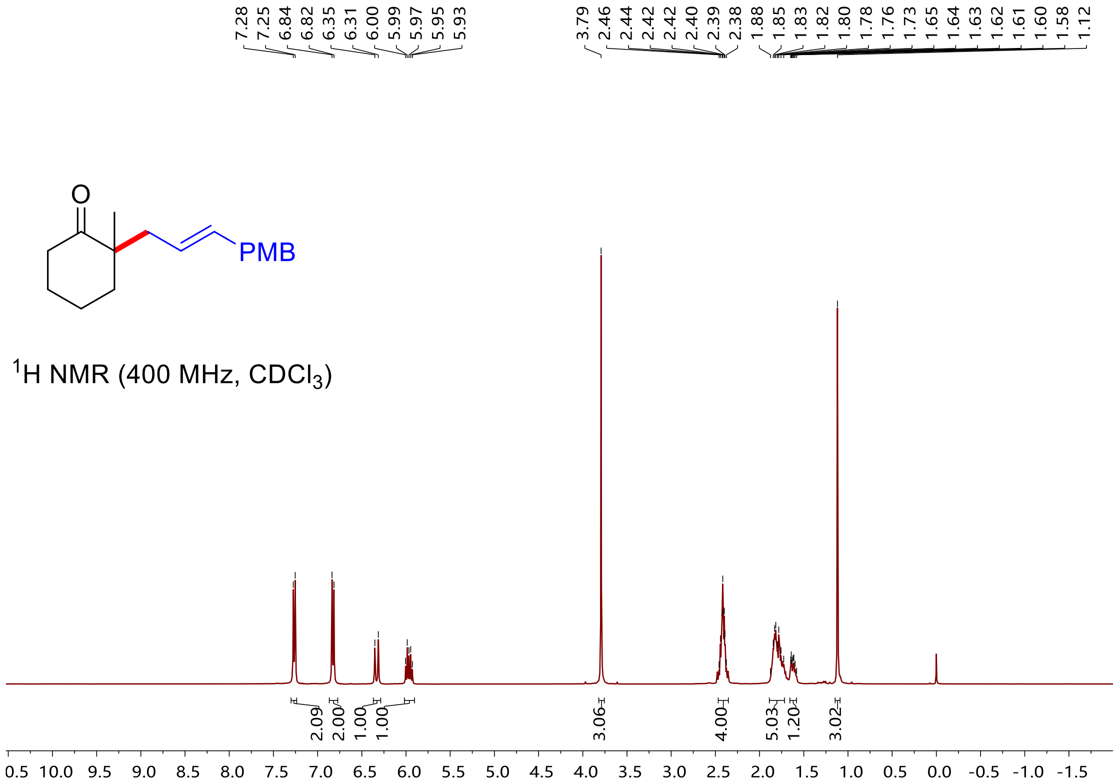


**Supplementary Fig. 62.** $^{1}$H NMR spectrum of compound **3i**.

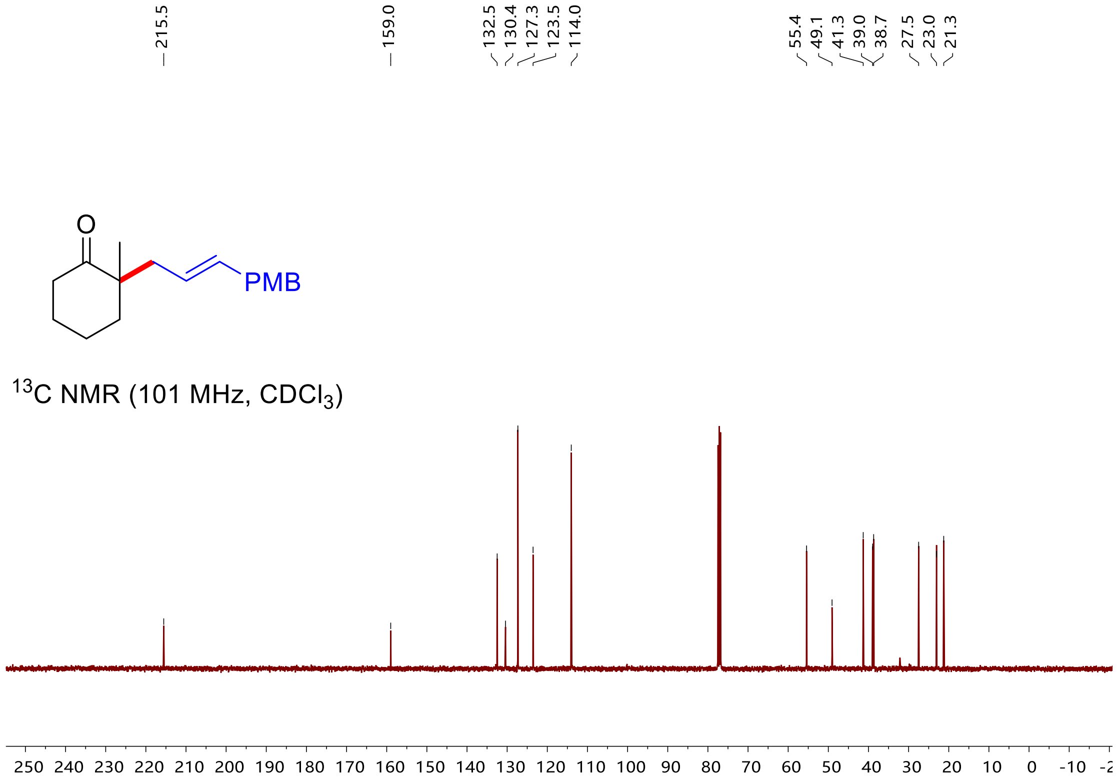


**Supplementary Fig. 63.** $^{13}$C NMR spectrum of compound **3i**.

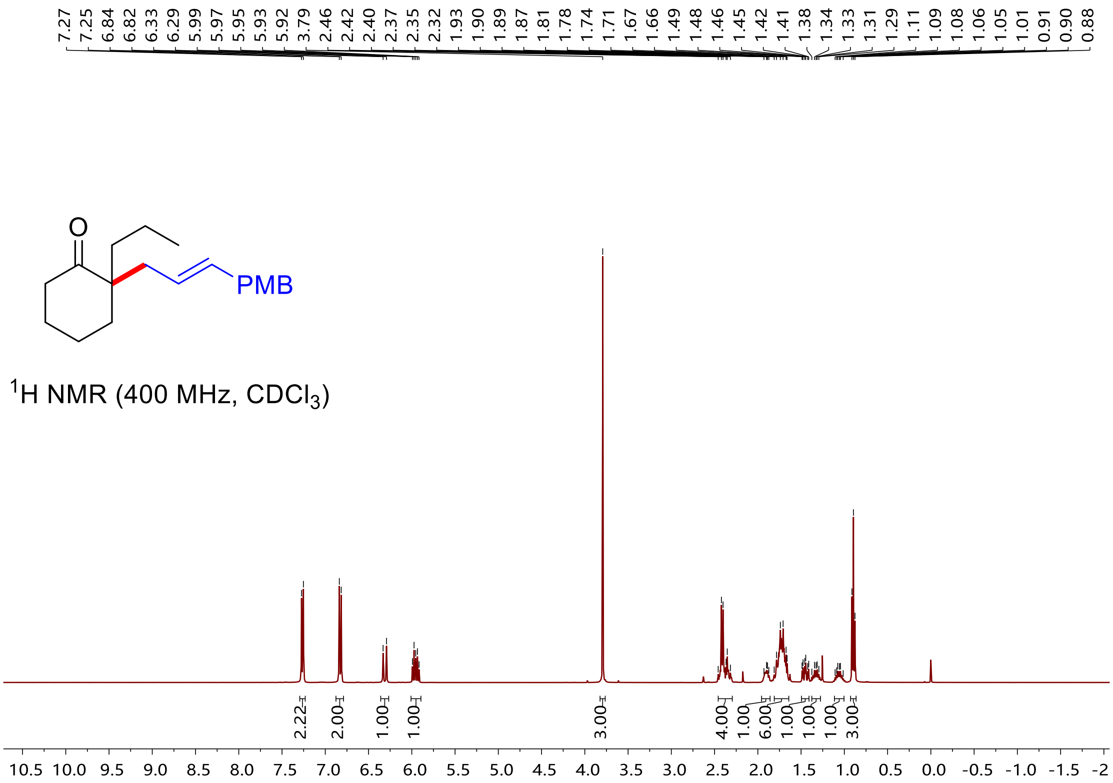


**Supplementary Fig. 64.** $^{1}$H NMR spectrum of compound **3j**.

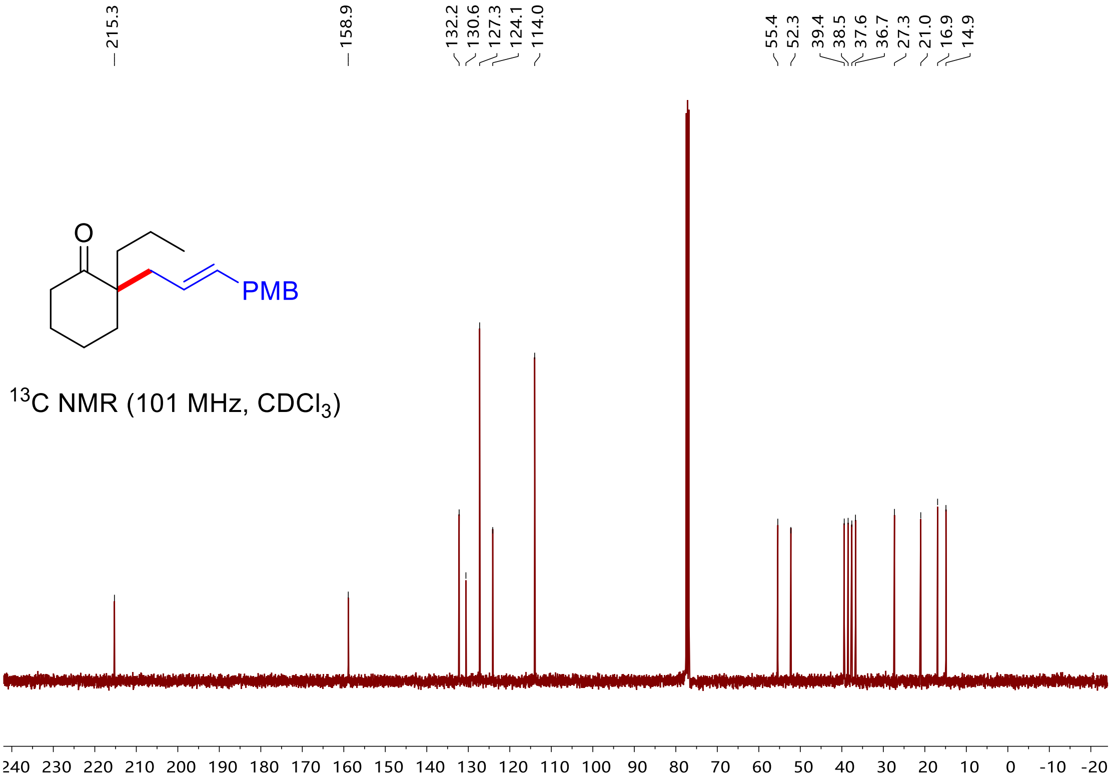


**Supplementary Fig. 65.** $^{13}$C NMR spectrum of compound **3j**.

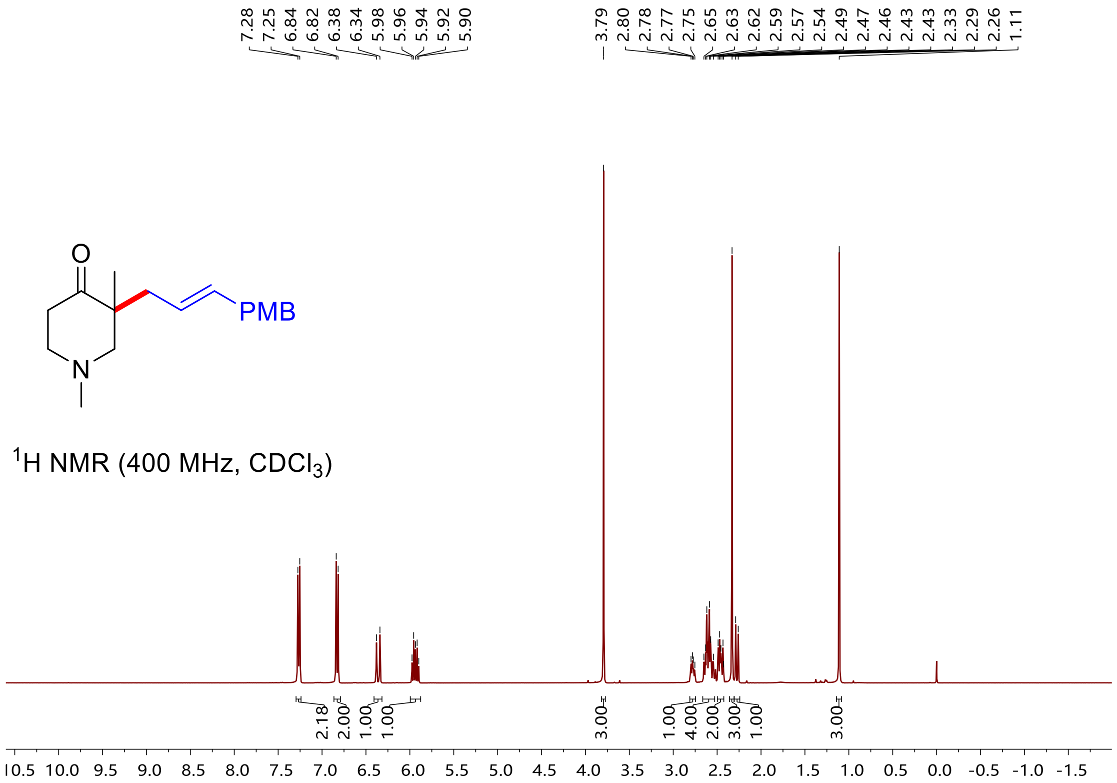


**Supplementary Fig. 66.** $^{1}$H NMR spectrum of compound **3k**.

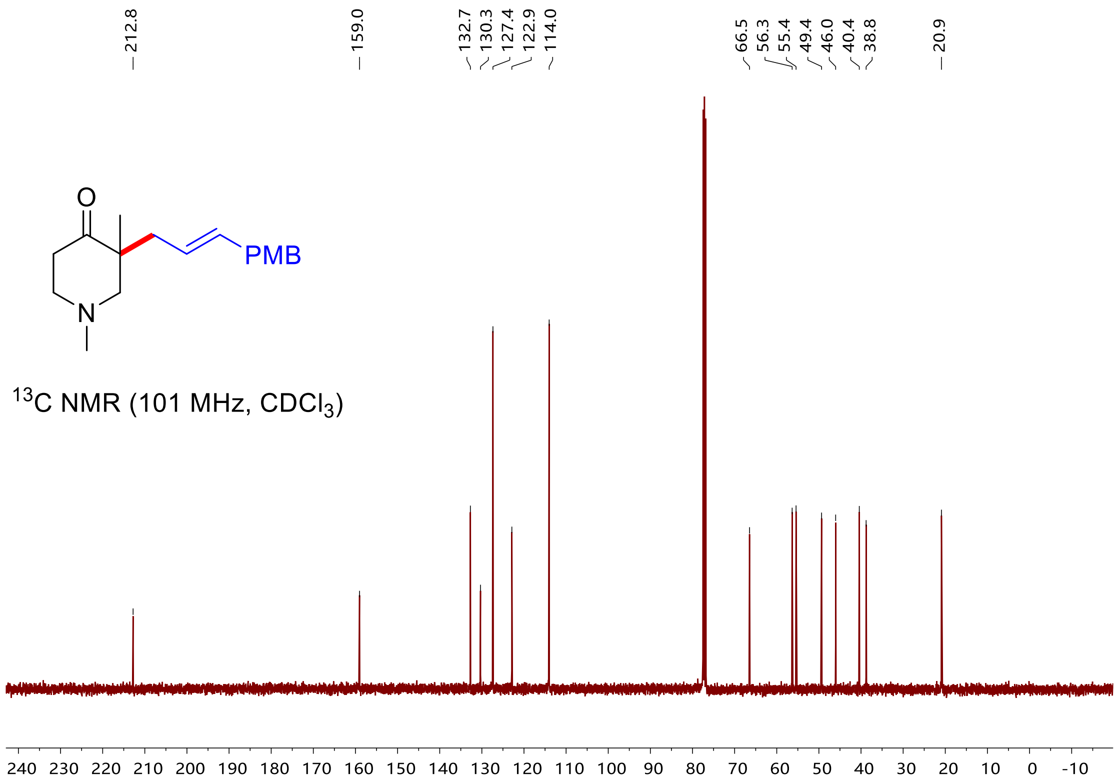


**Supplementary Fig. 67.** $^{13}$C NMR spectrum of compound **3k**.

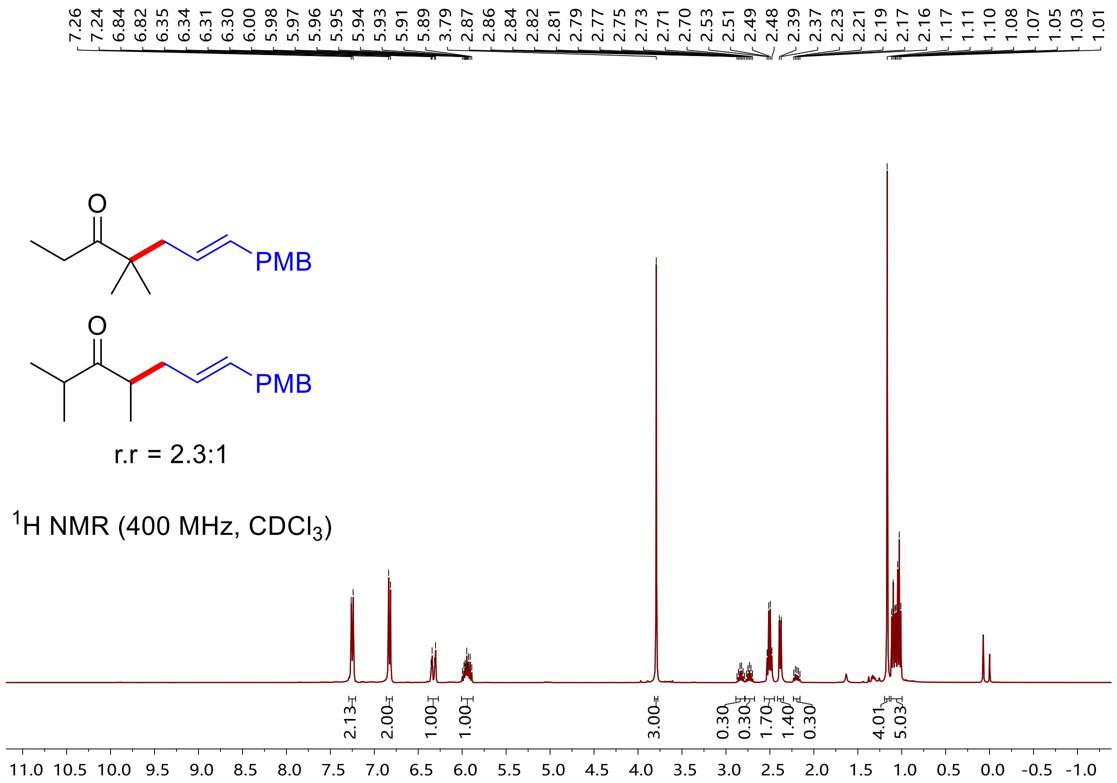


**Supplementary Fig. 68.** $^{1}$H NMR spectrum of compound **3l**. (mixture)

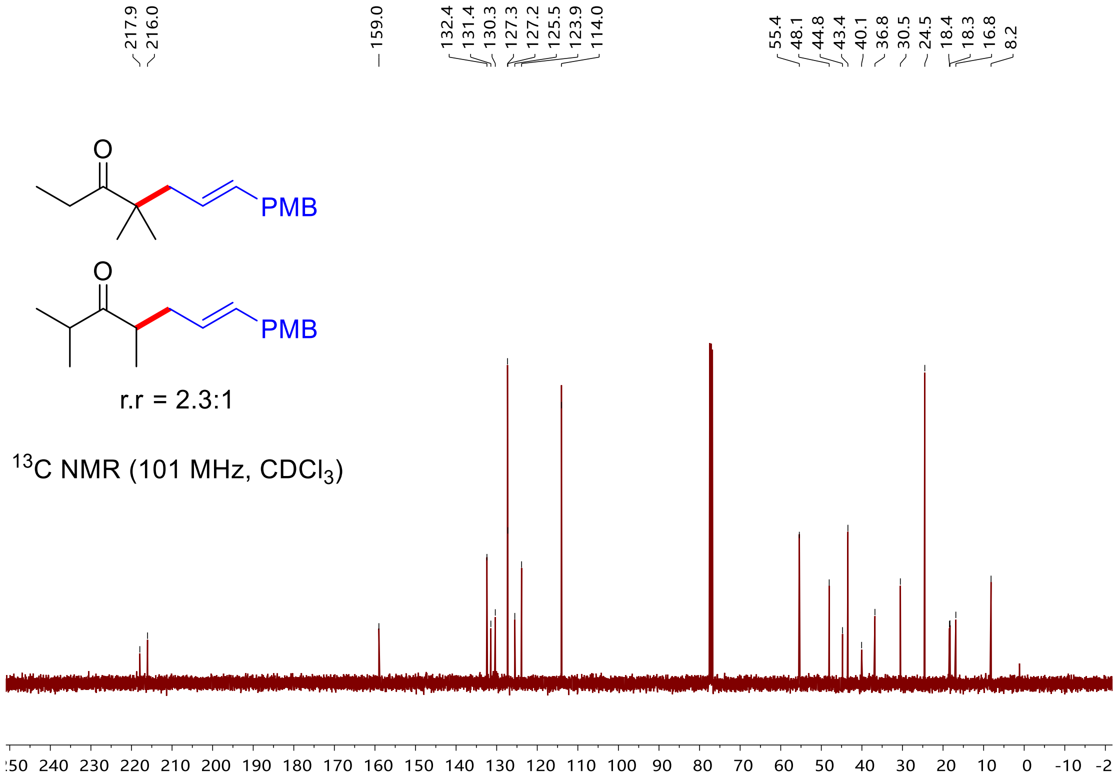


**Supplementary Fig. 69.** $^{13}$C NMR spectrum of compound **3l**. (mixture)

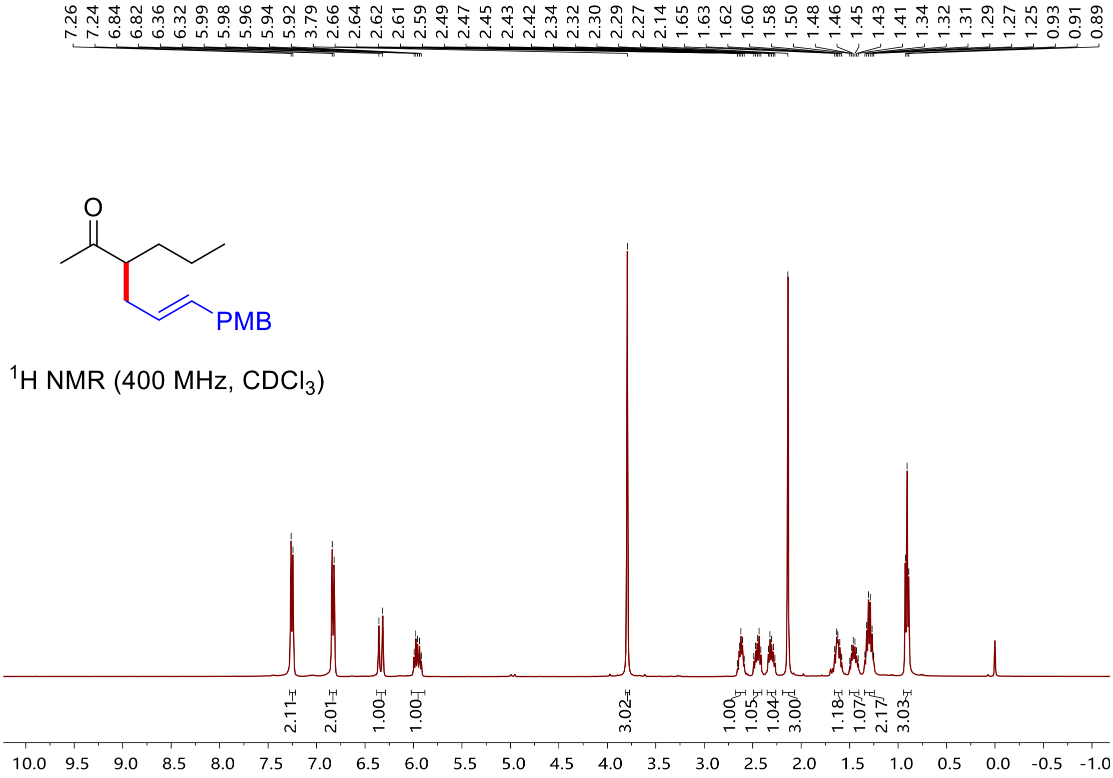


**Supplementary Fig. 70.** $^{1}$H NMR spectrum of compound **3m**.

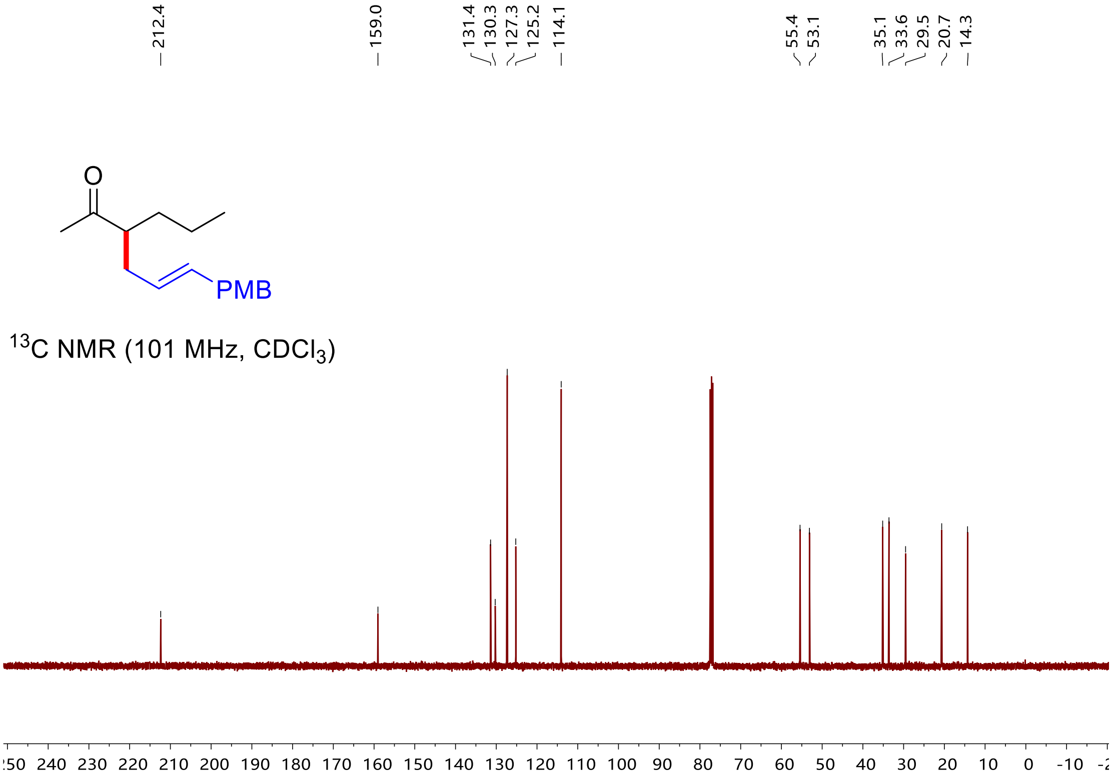


**Supplementary Fig. 71.** $^{13}$C NMR spectrum of compound **3m**.

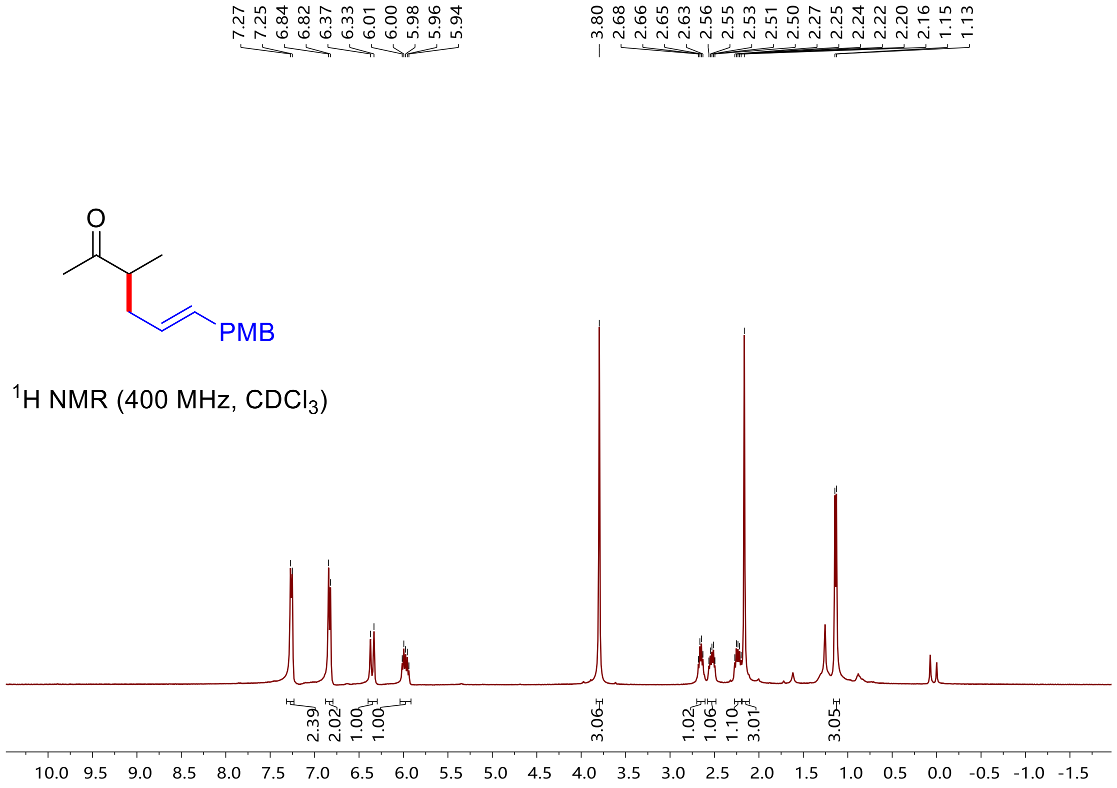


**Supplementary Fig. 72.** $^{1}H$ NMR spectrum of compound **3n**.

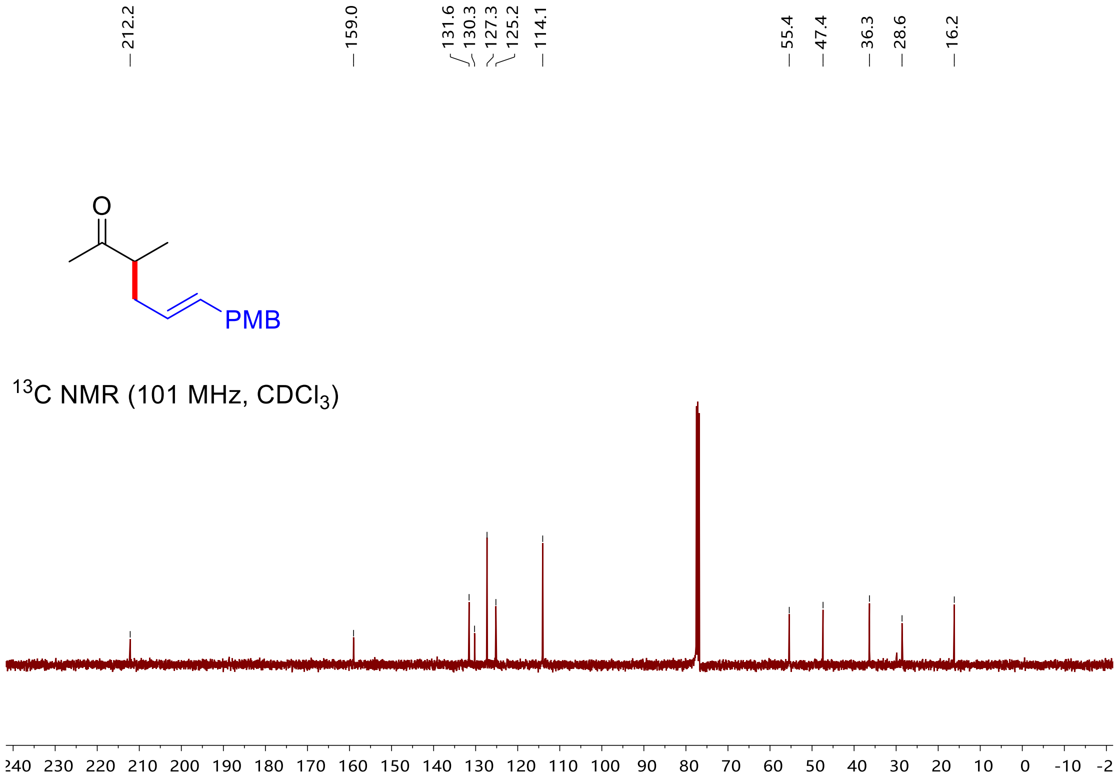


**Supplementary Fig. 73.** $^{13}C$ NMR spectrum of compound **3n**.

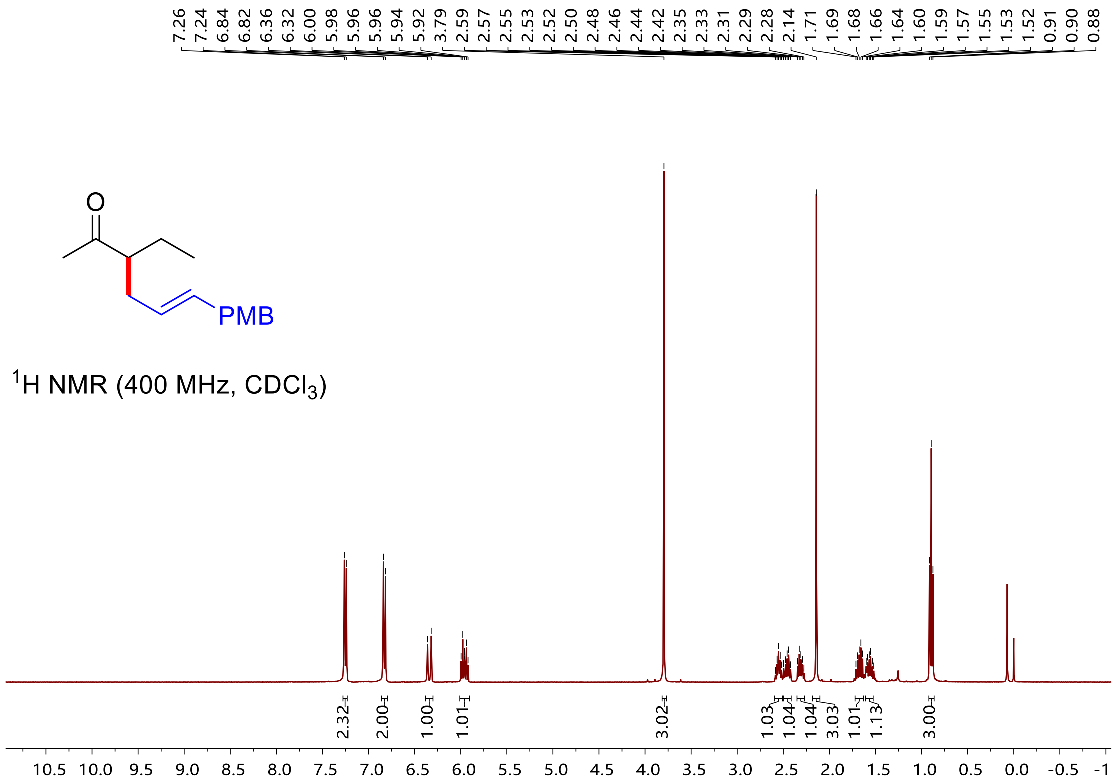


**Supplementary Fig. 74.** $^{1}$H NMR spectrum of compound **3o**.

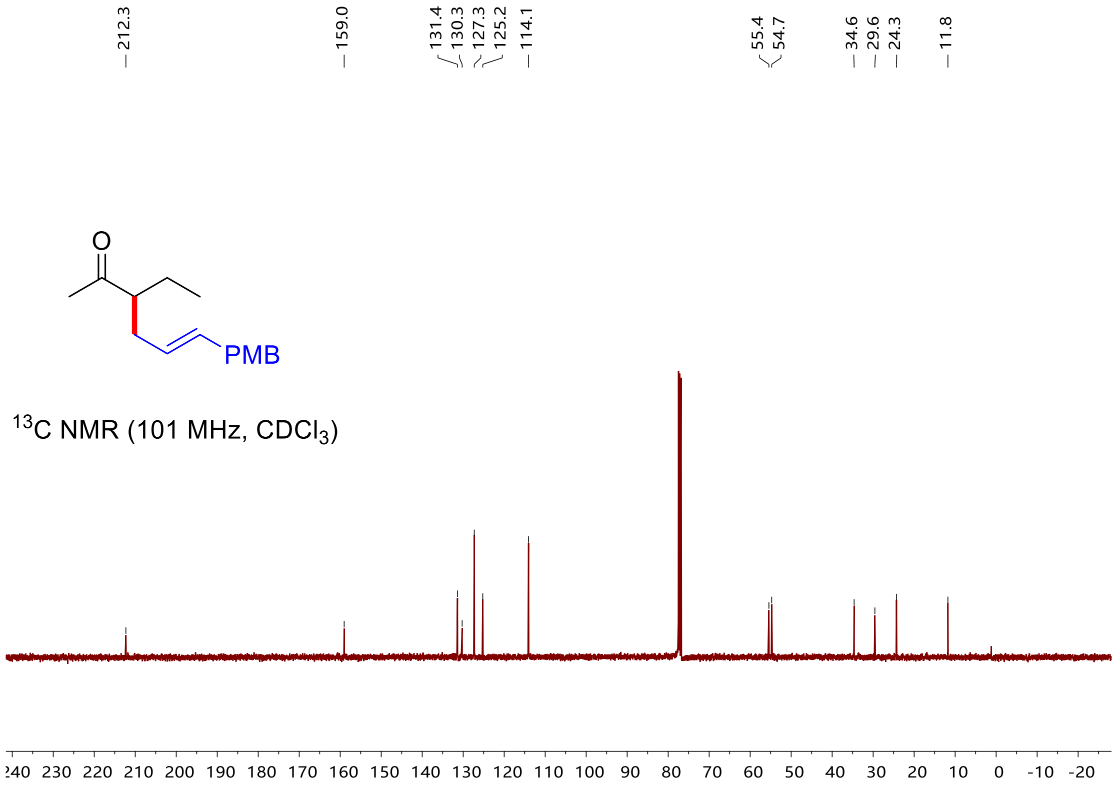


**Supplementary Fig. 75.** $^{13}$C NMR spectrum of compound **3o**.

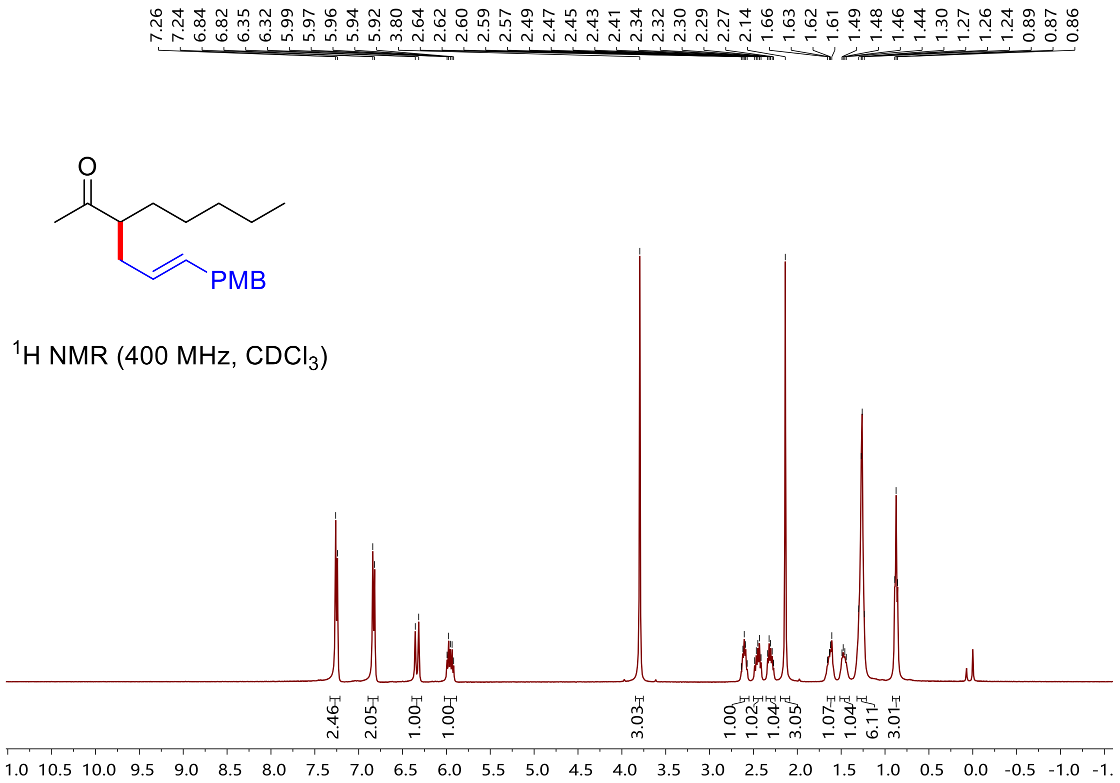


**Supplementary Fig. 76.** $^1$H NMR spectrum of compound **3p**.

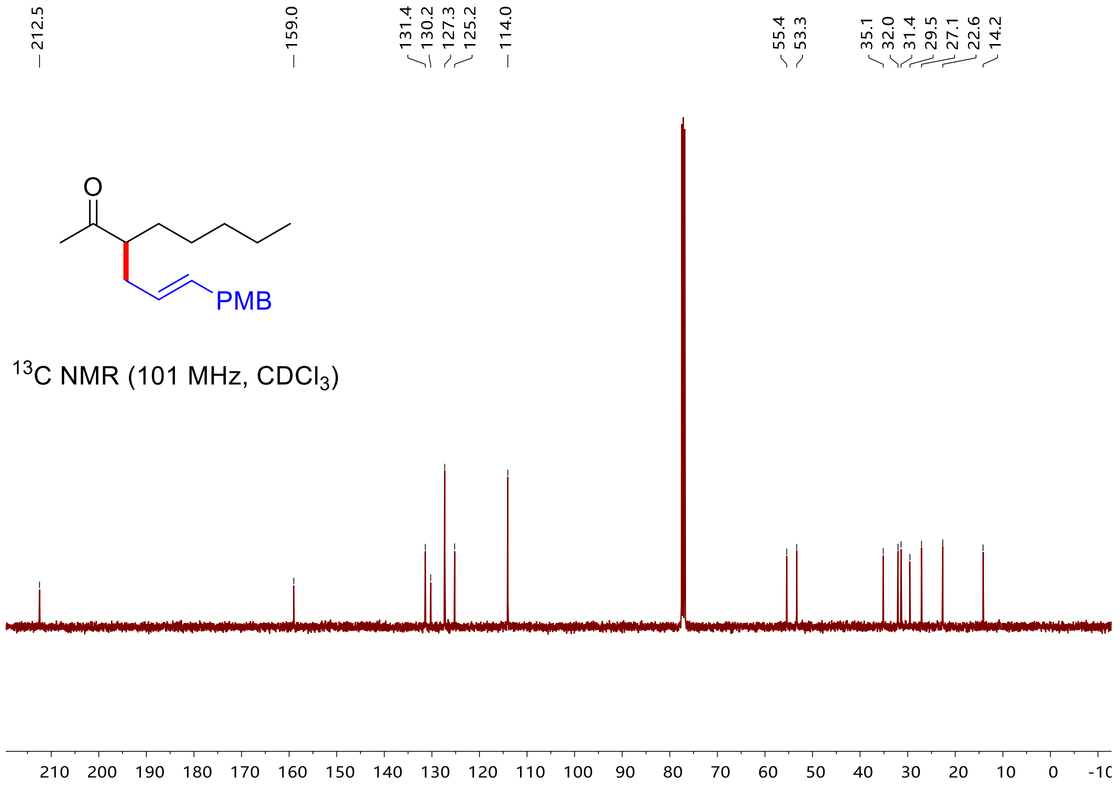


**Supplementary Fig. 77.** $^{13}$C NMR spectrum of compound **3p**.

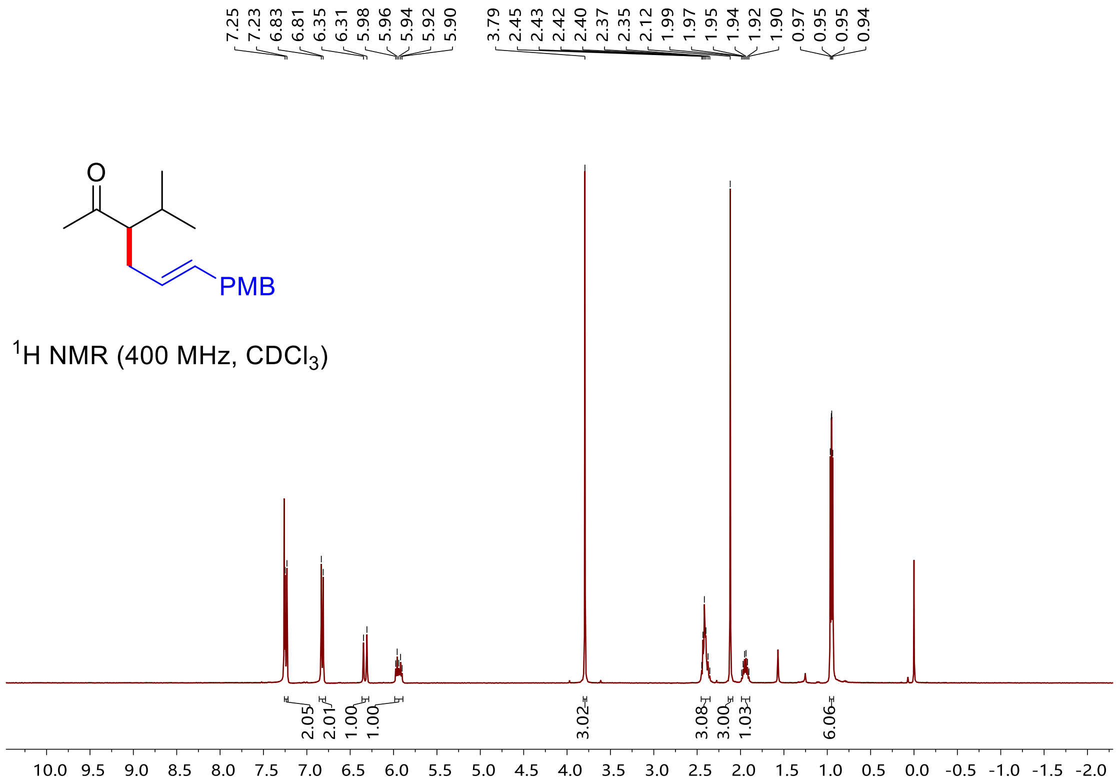


**Supplementary Fig. 78.** $^{1}$H NMR spectrum of compound **3q**.

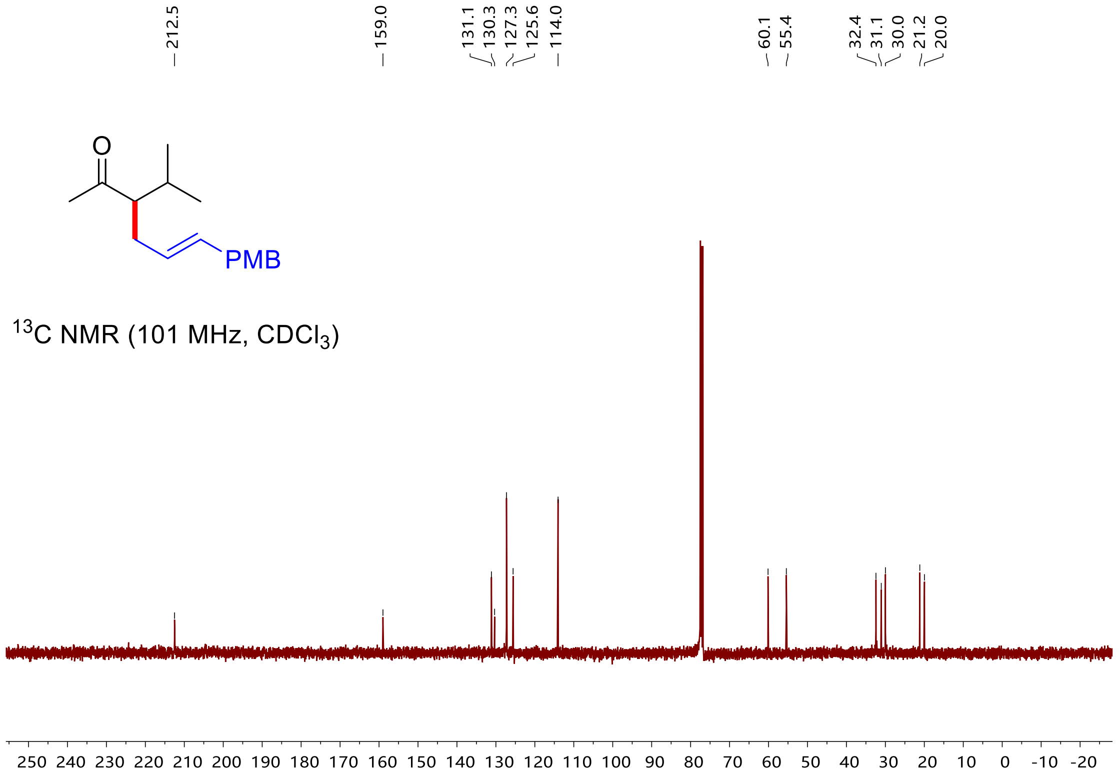


**Supplementary Fig. 79.** $^{13}$C NMR spectrum of compound **3q**.

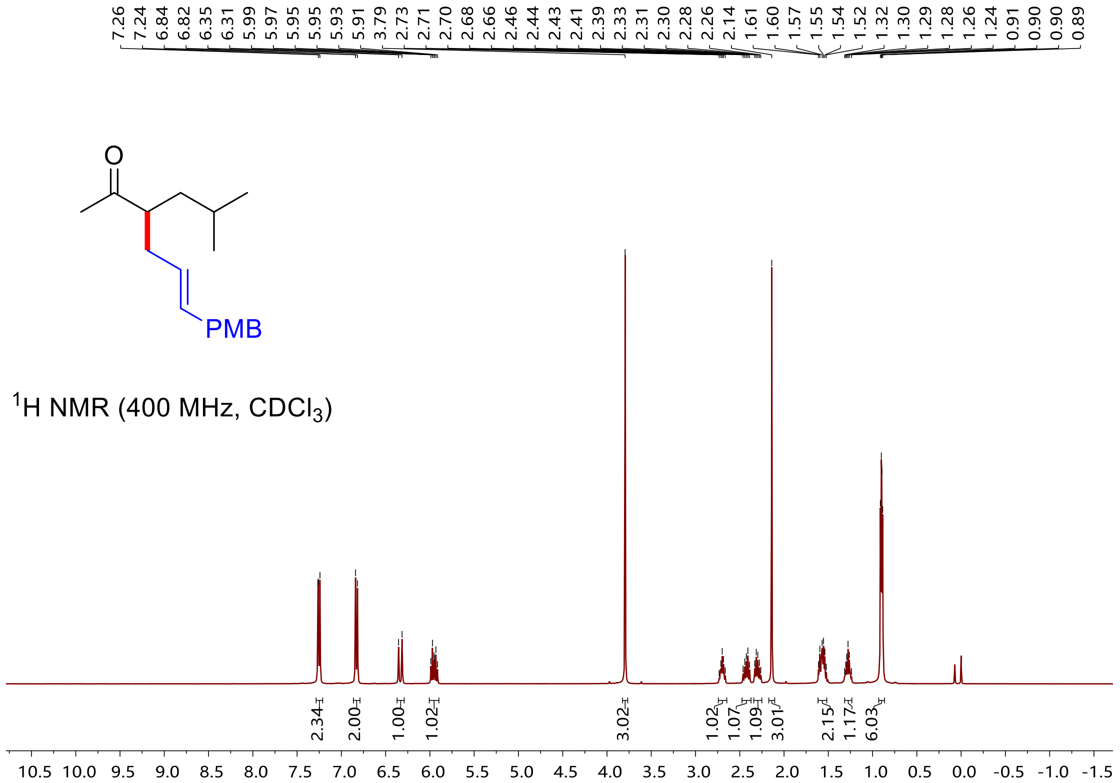


**Supplementary Fig. 80.** $^{1}$H NMR spectrum of compound **3r**.

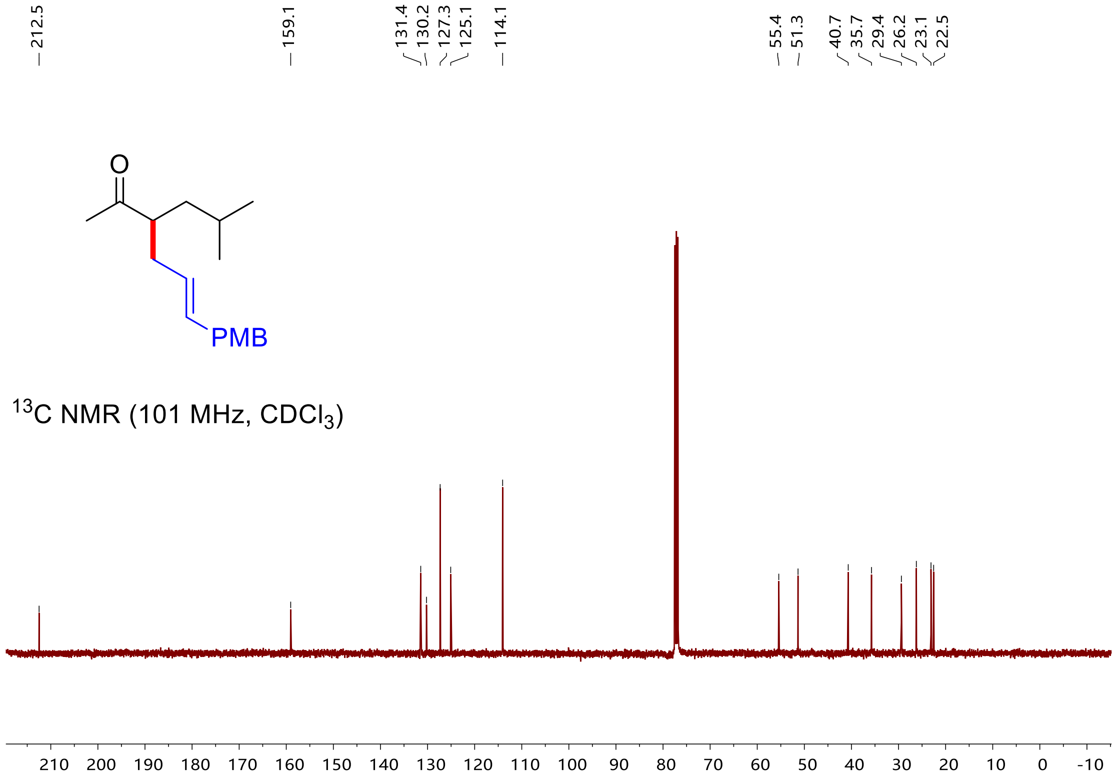


**Supplementary Fig. 81.** $^{13}$C NMR spectrum of compound **3r**.

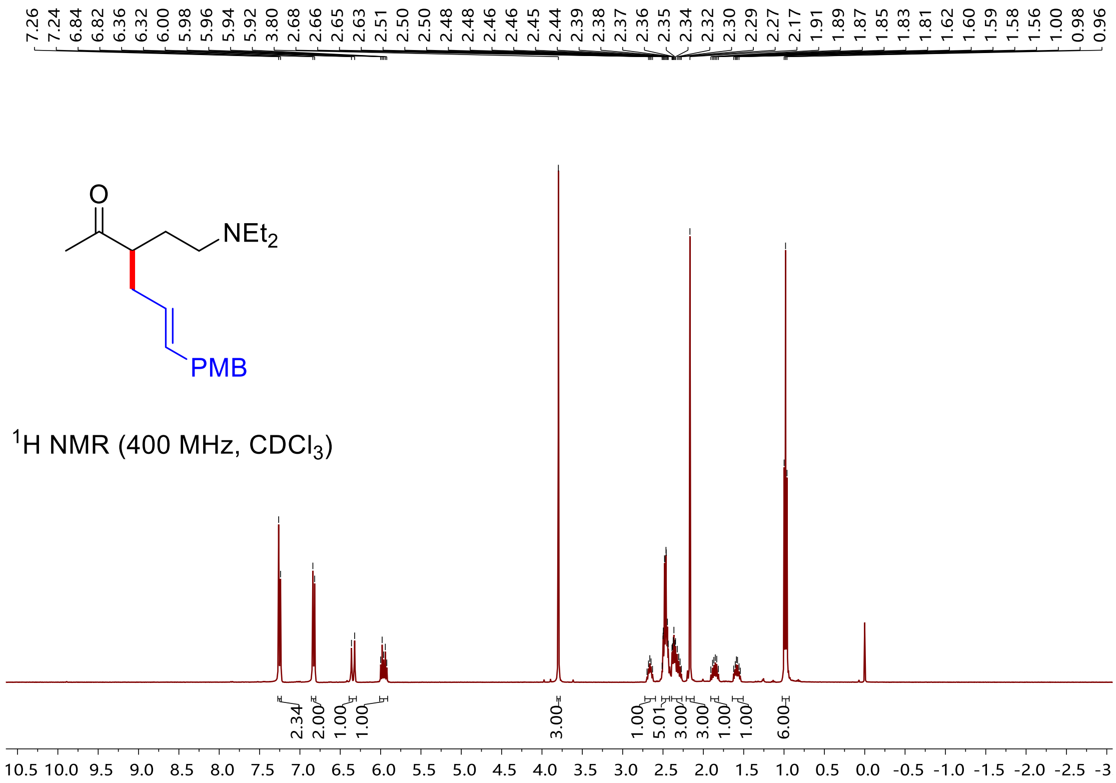


**Supplementary Fig. 82.** $^{1}$H NMR spectrum of compound **3s**.

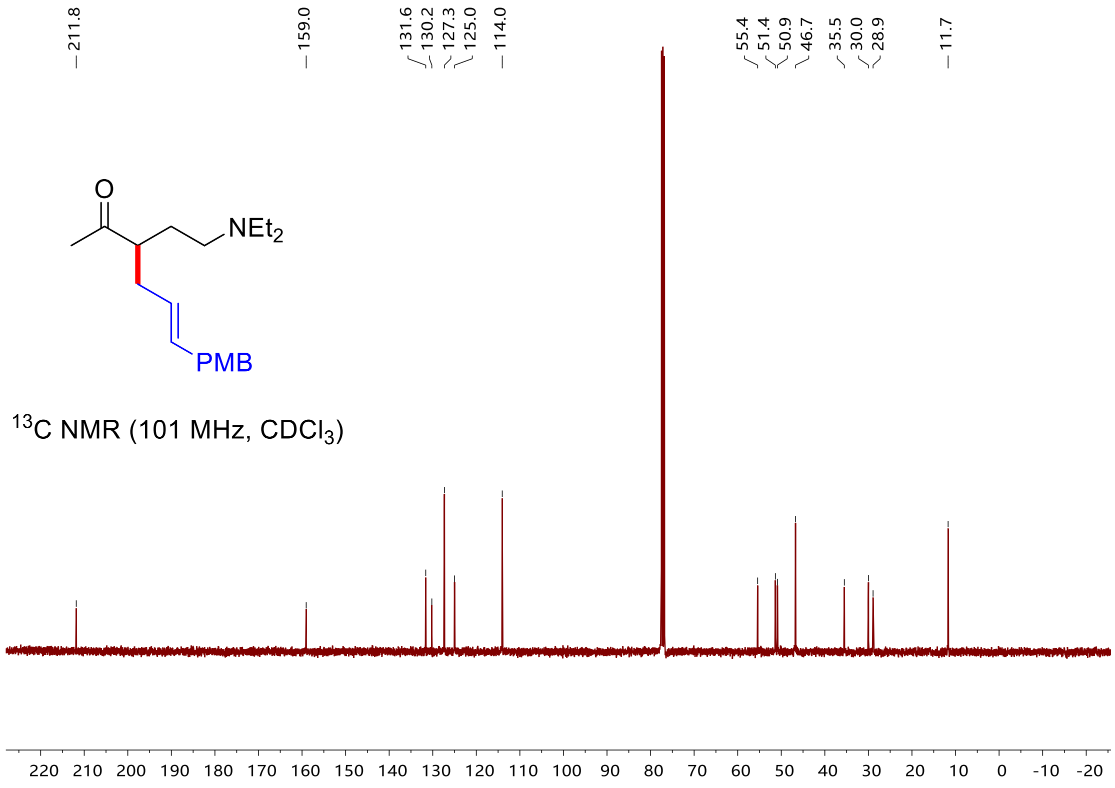


**Supplementary Fig. 83.** $^{13}$C NMR spectrum of compound **3s**.

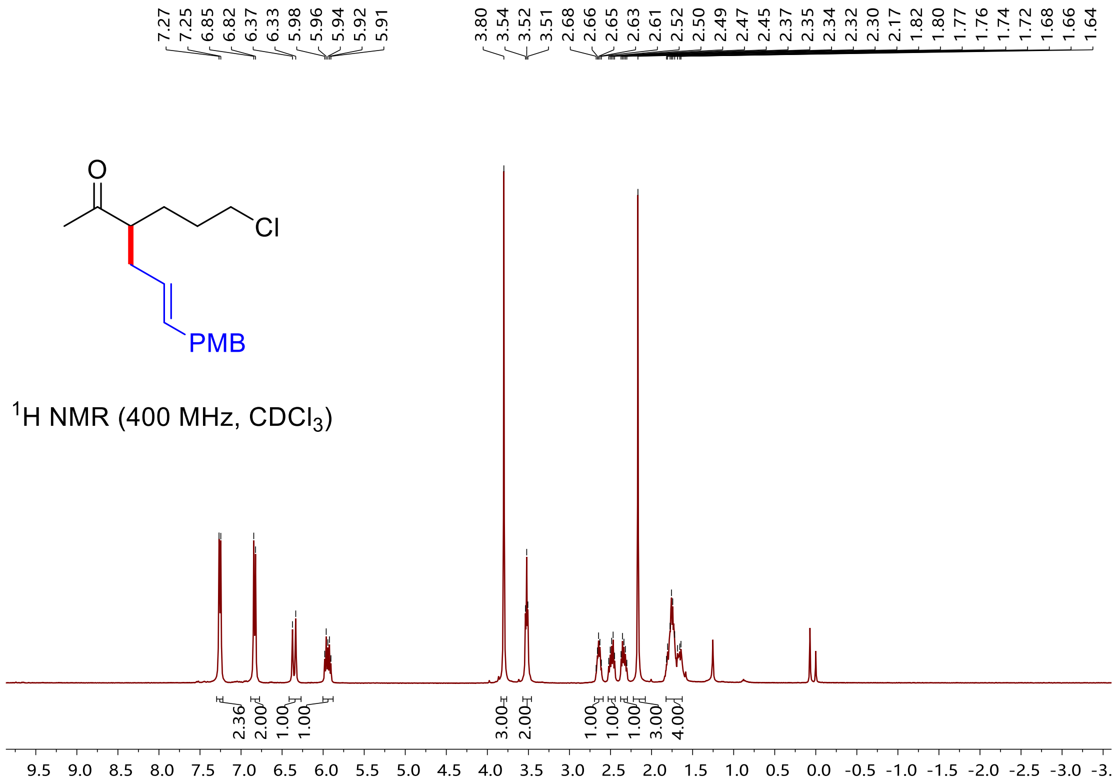


**Supplementary Fig. 84.** $^1$H NMR spectrum of compound **3t**.

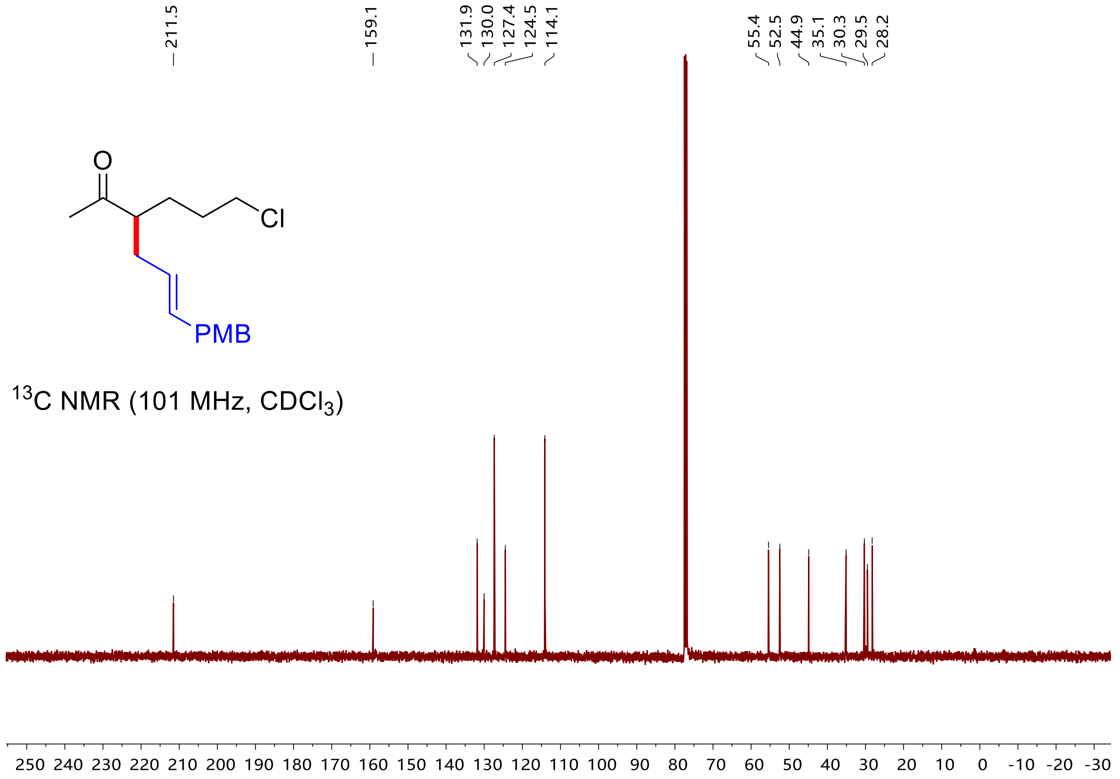


**Supplementary Fig. 85.** $^{13}$C NMR spectrum of compound **3t**.

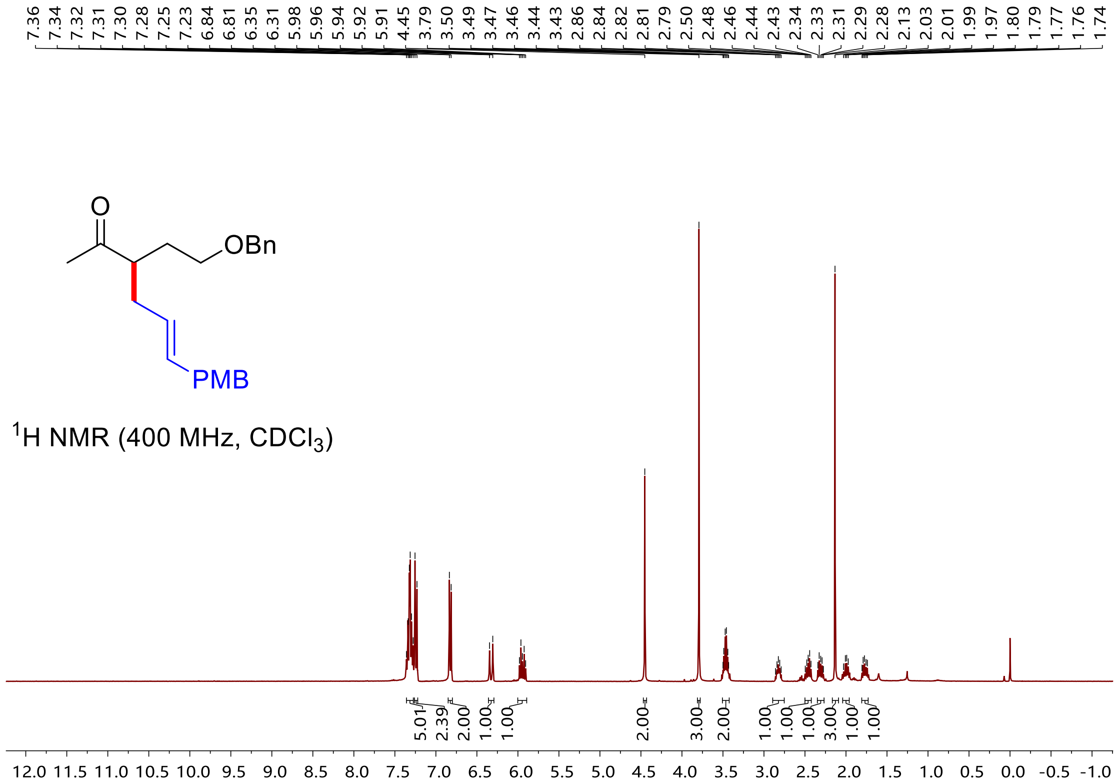


**Supplementary Fig. 86.** $^1$H NMR spectrum of compound **3u**.

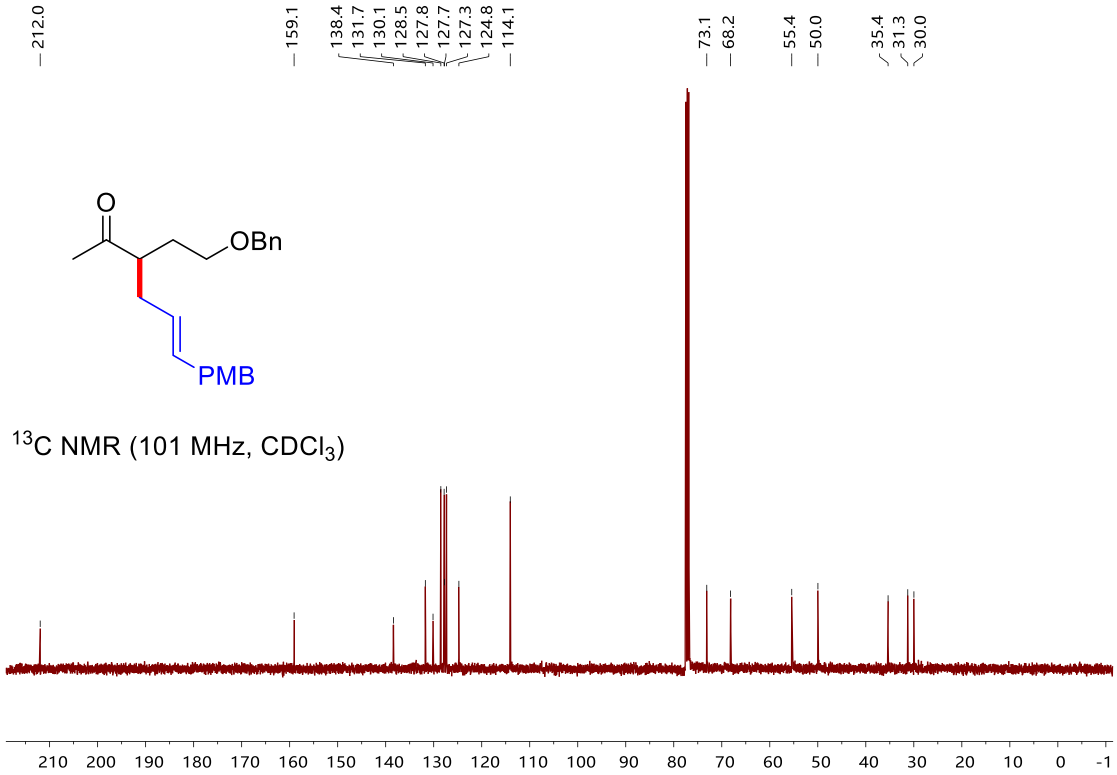


**Supplementary Fig. 87.** $^{13}$C NMR spectrum of compound **3u**.

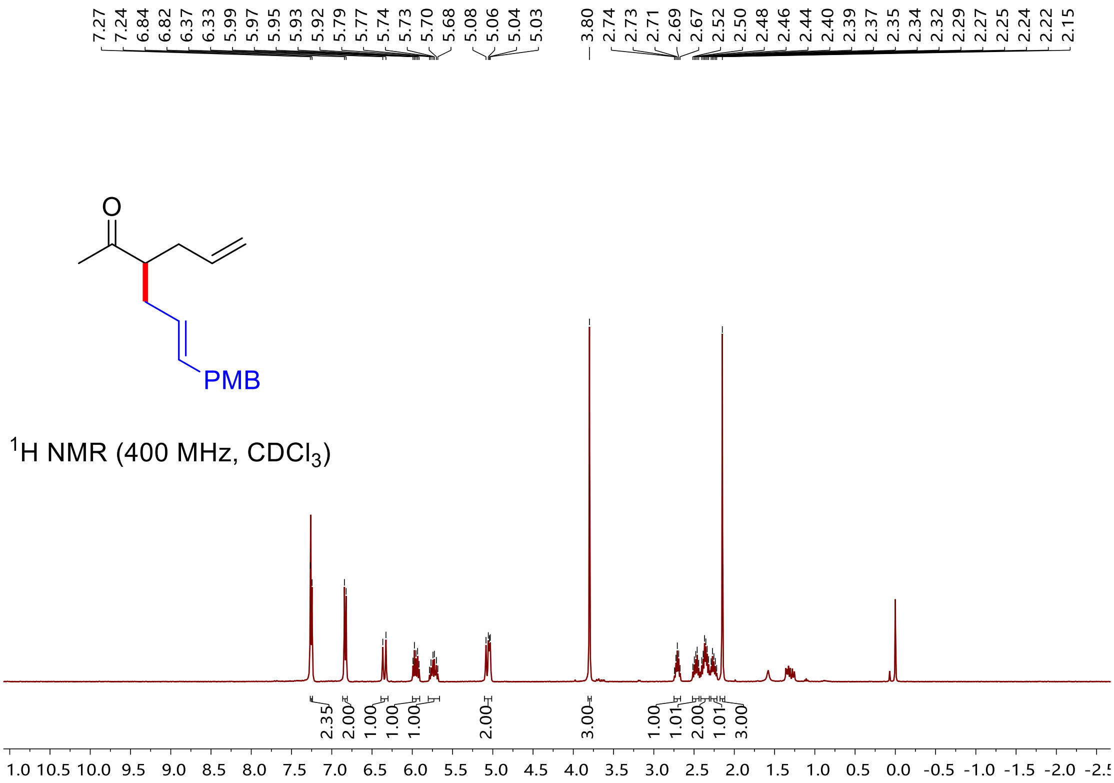


**Supplementary Fig. 88.** $^{1}$H NMR spectrum of compound **3v**.

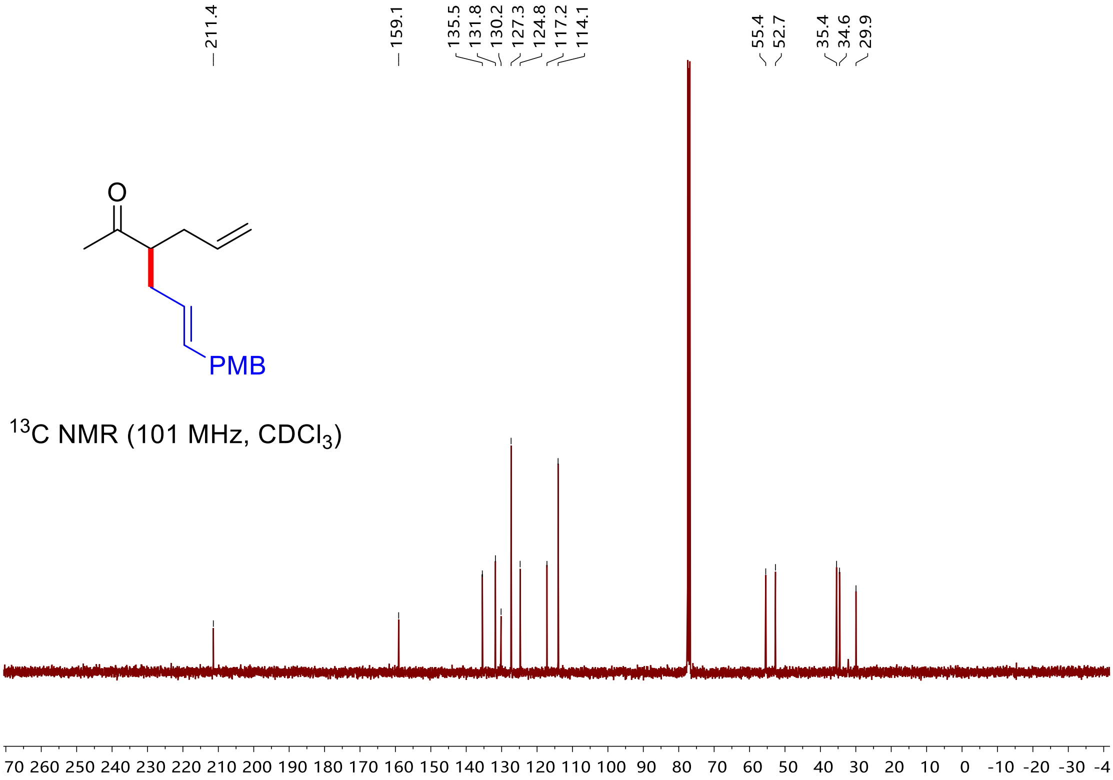


**Supplementary Fig. 89.** $^{13}$C NMR spectrum of compound **3v**.

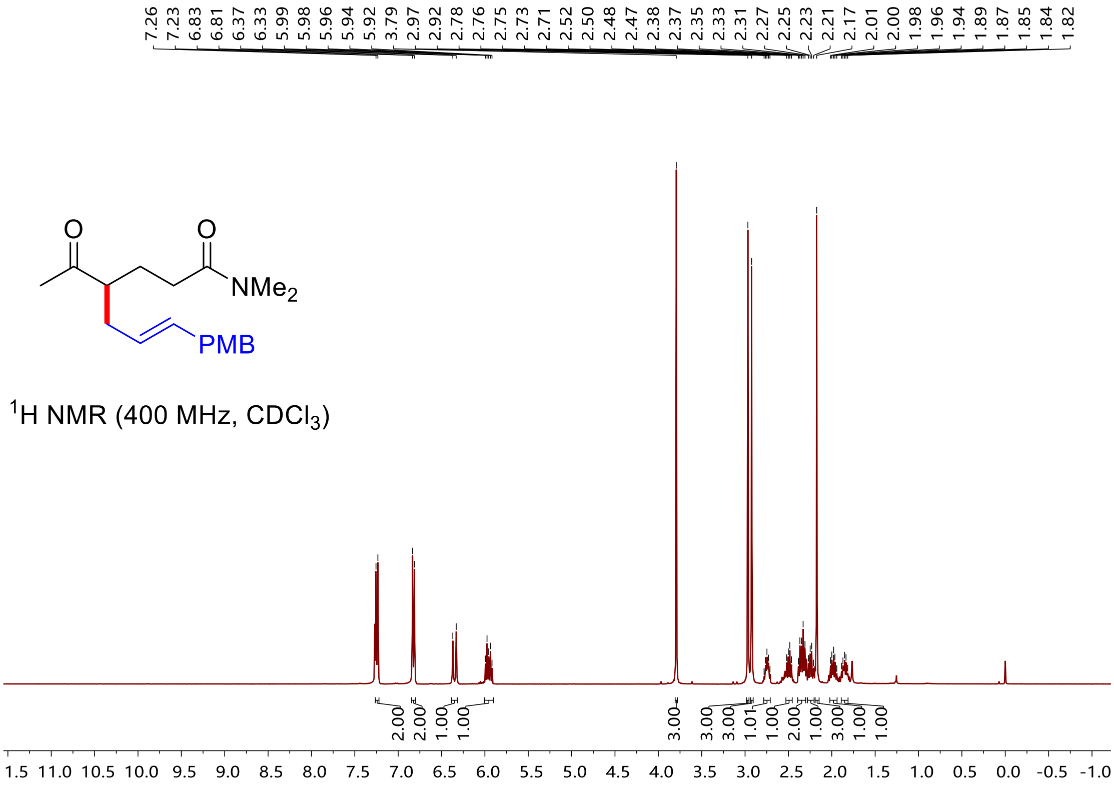


**Supplementary Fig. 90.** $^{1}H$ NMR spectrum of compound **3w**.

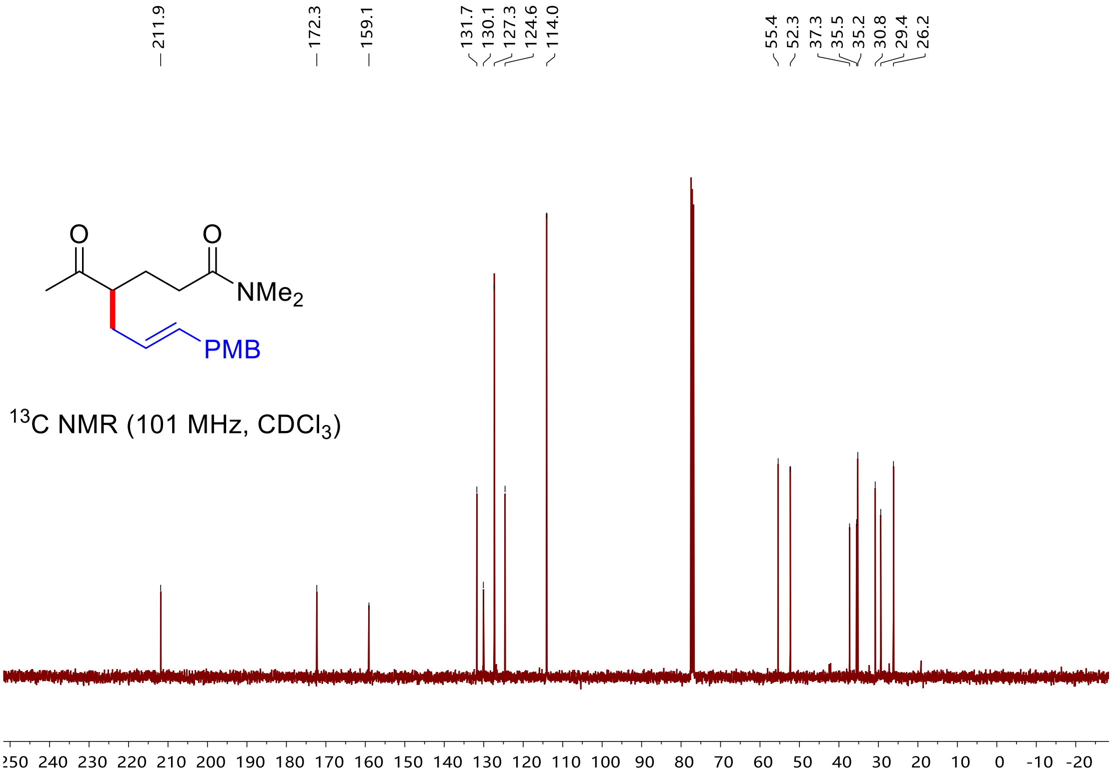


**Supplementary Fig. 91.** $^{13}C$ NMR spectrum of compound **3w**.

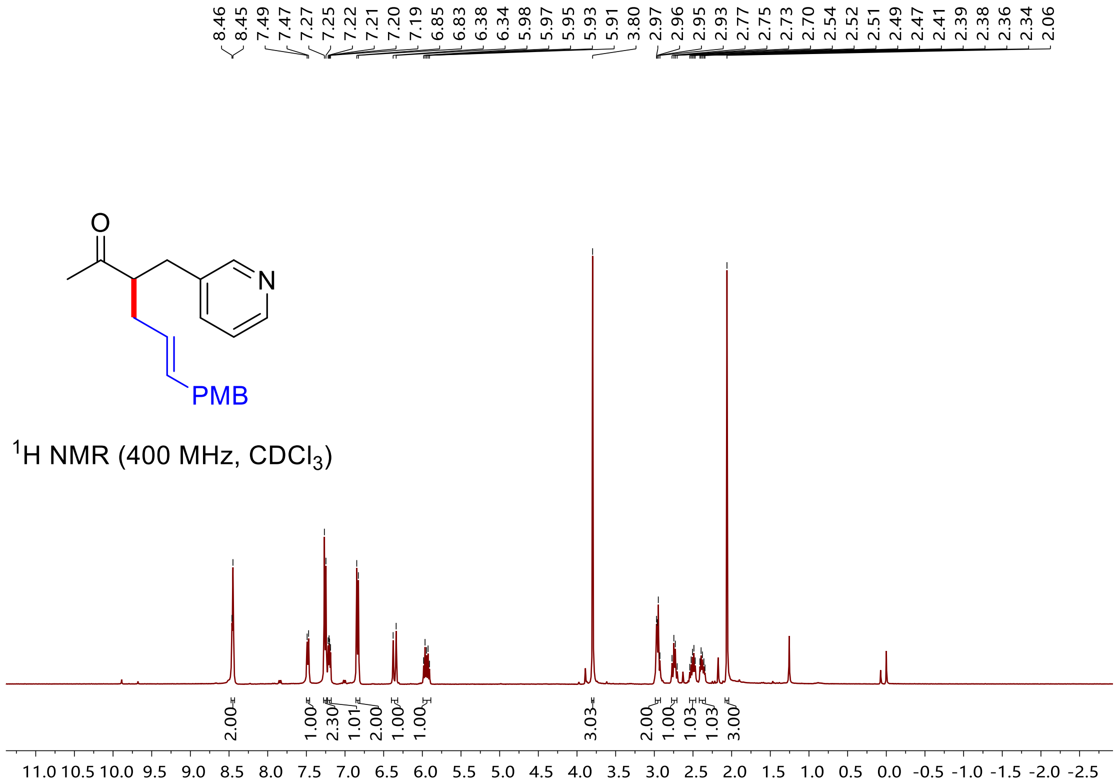


**Supplementary Fig. 92.** $^{1}$H NMR spectrum of compound **3x**.

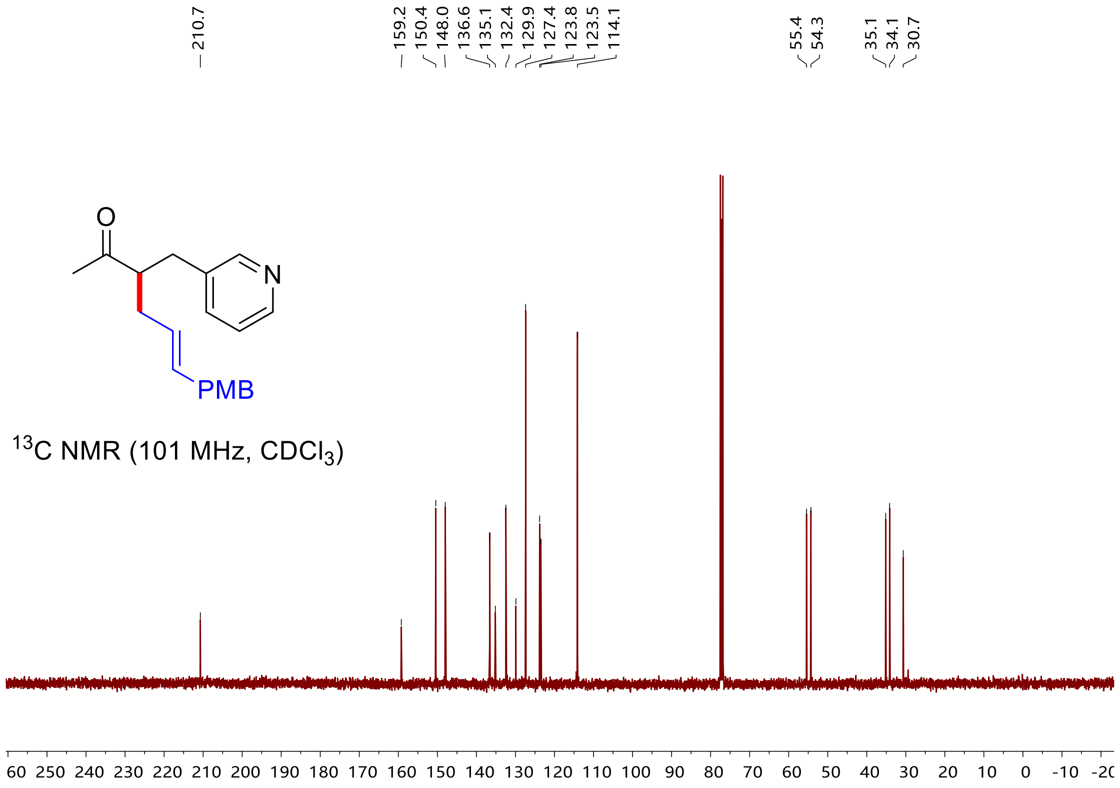


**Supplementary Fig. 93.** $^{13}$C NMR spectrum of compound **3x**.

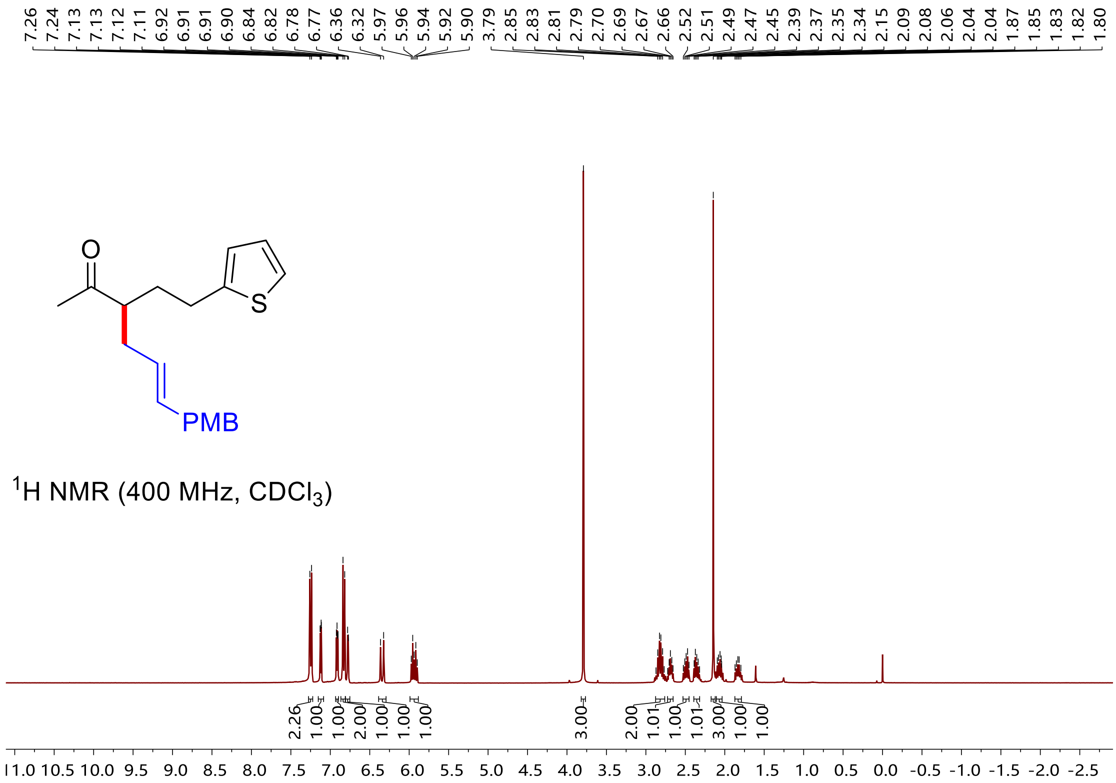


**Supplementary Fig. 94.** $^{1}$H NMR spectrum of compound **3y**.

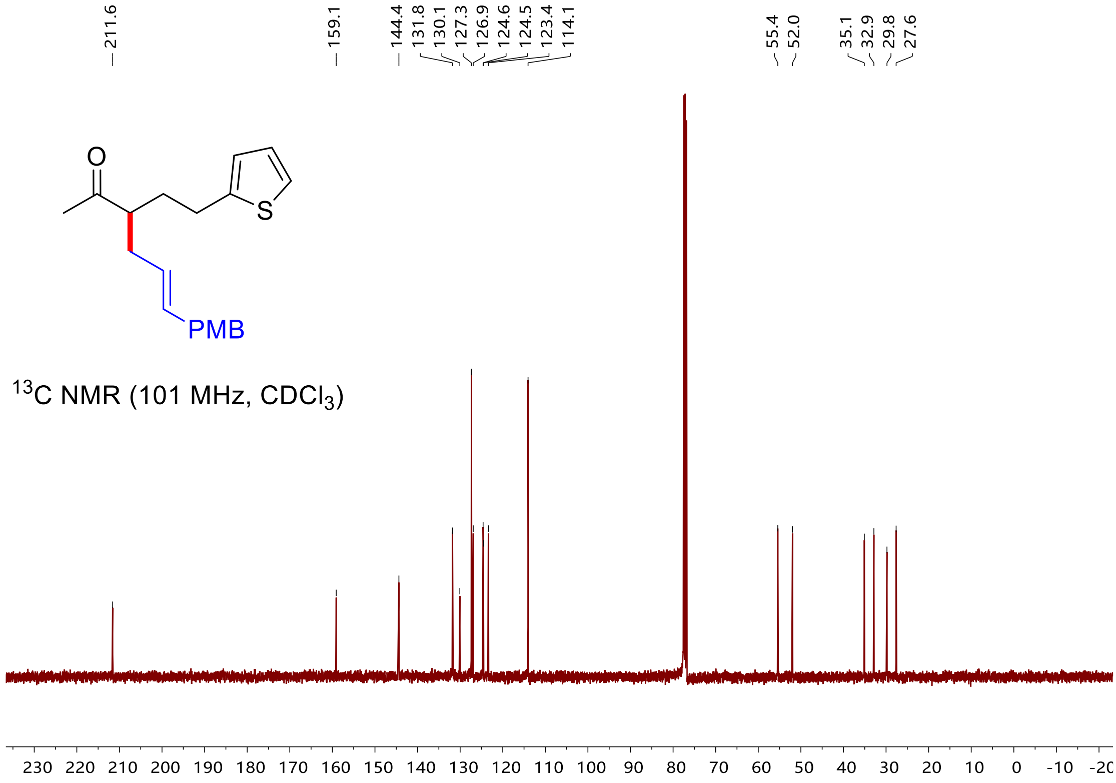


**Supplementary Fig. 95.** $^{13}$C NMR spectrum of compound **3y**.

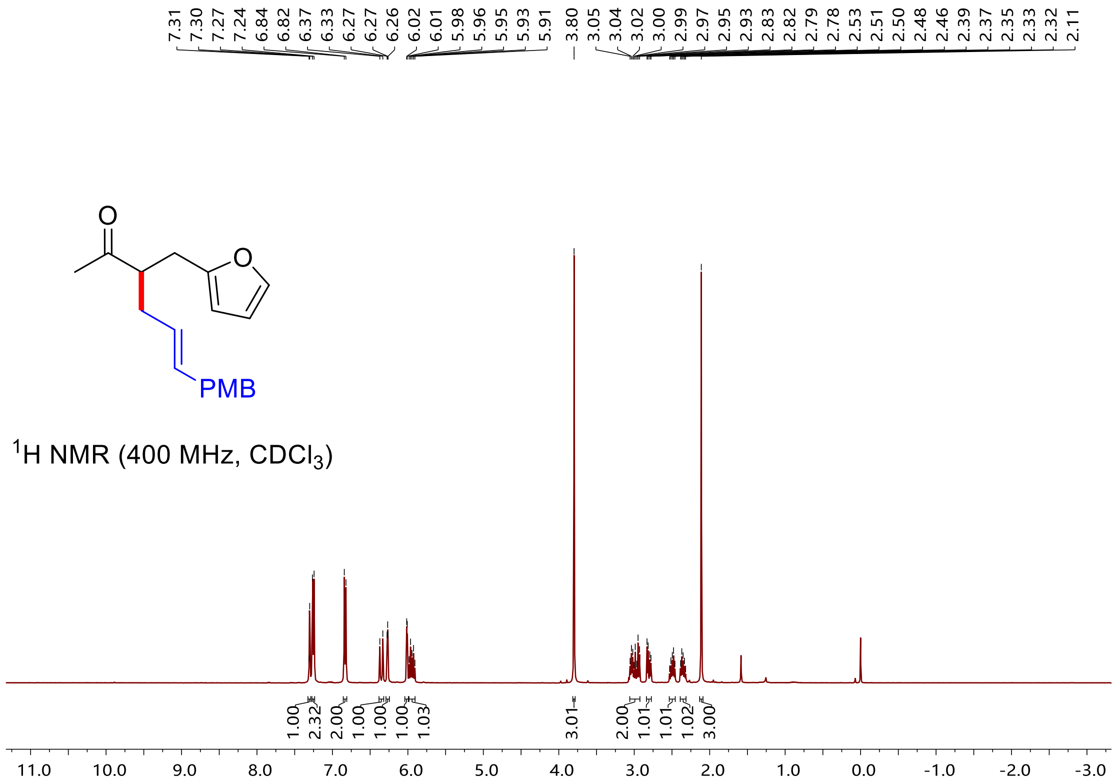


**Supplementary Fig. 96.** $^{1}$H NMR spectrum of compound **3z**.

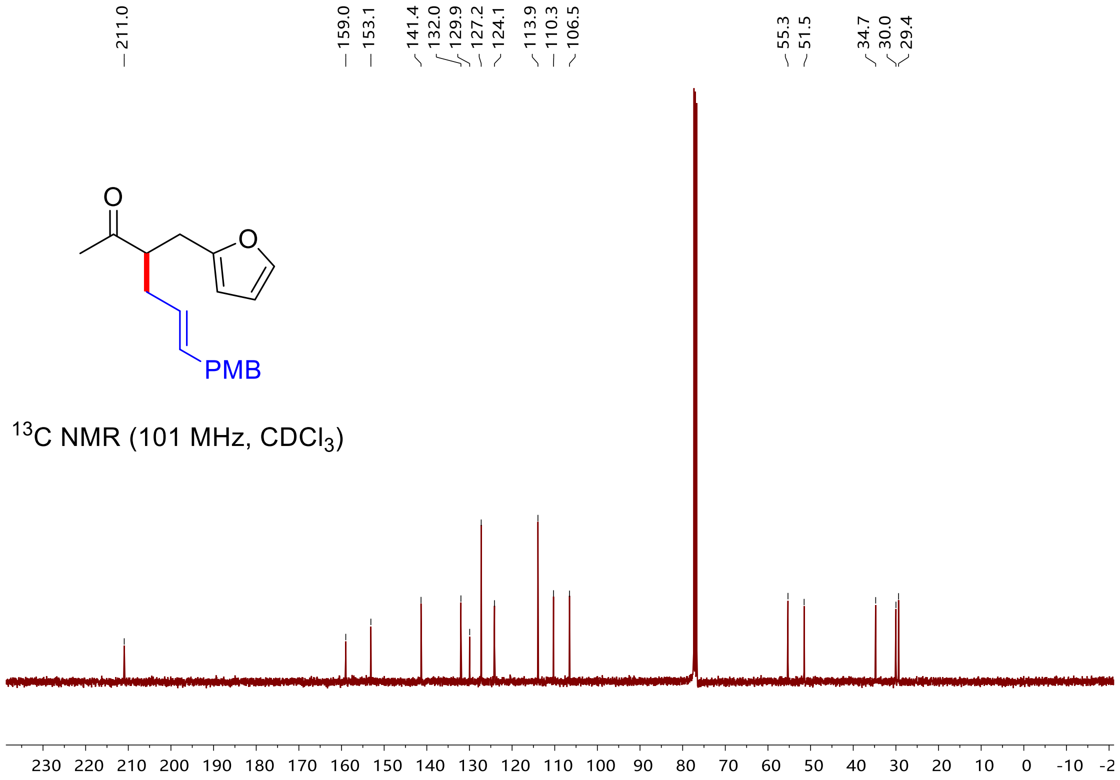


**Supplementary Fig. 97.** $^{13}$C NMR spectrum of compound **3z**.

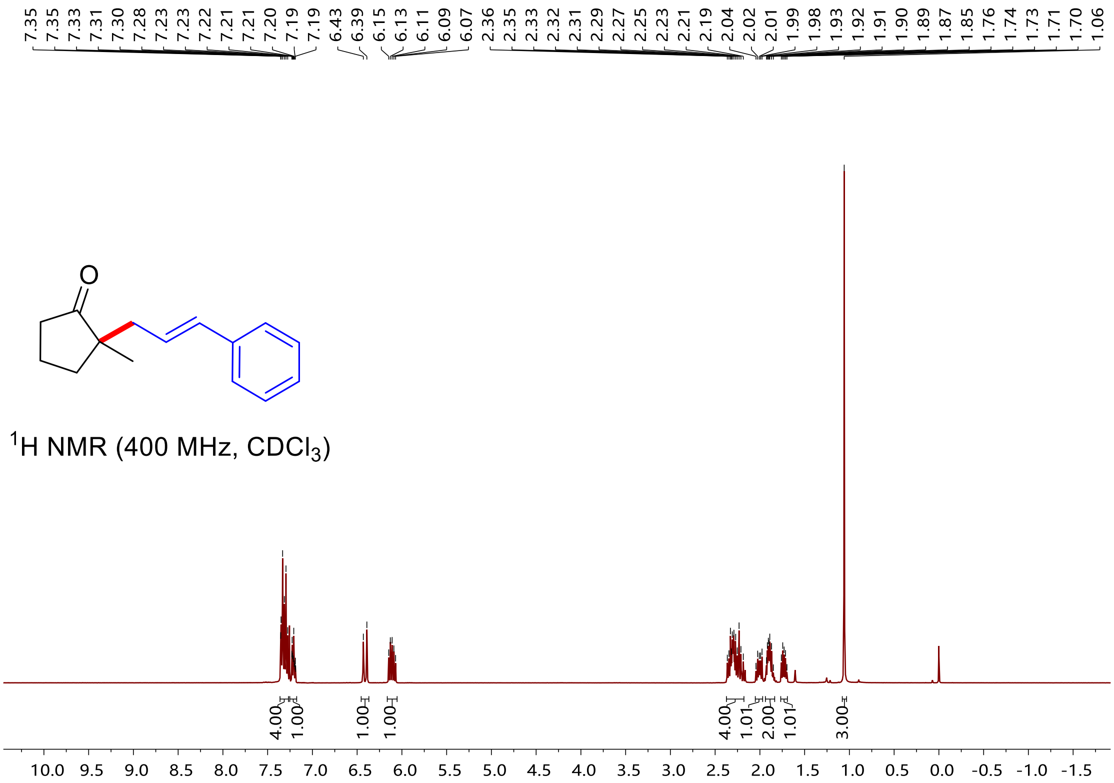


**Supplementary Fig. 98.** $^{13}$C NMR spectrum of compound **6a**.

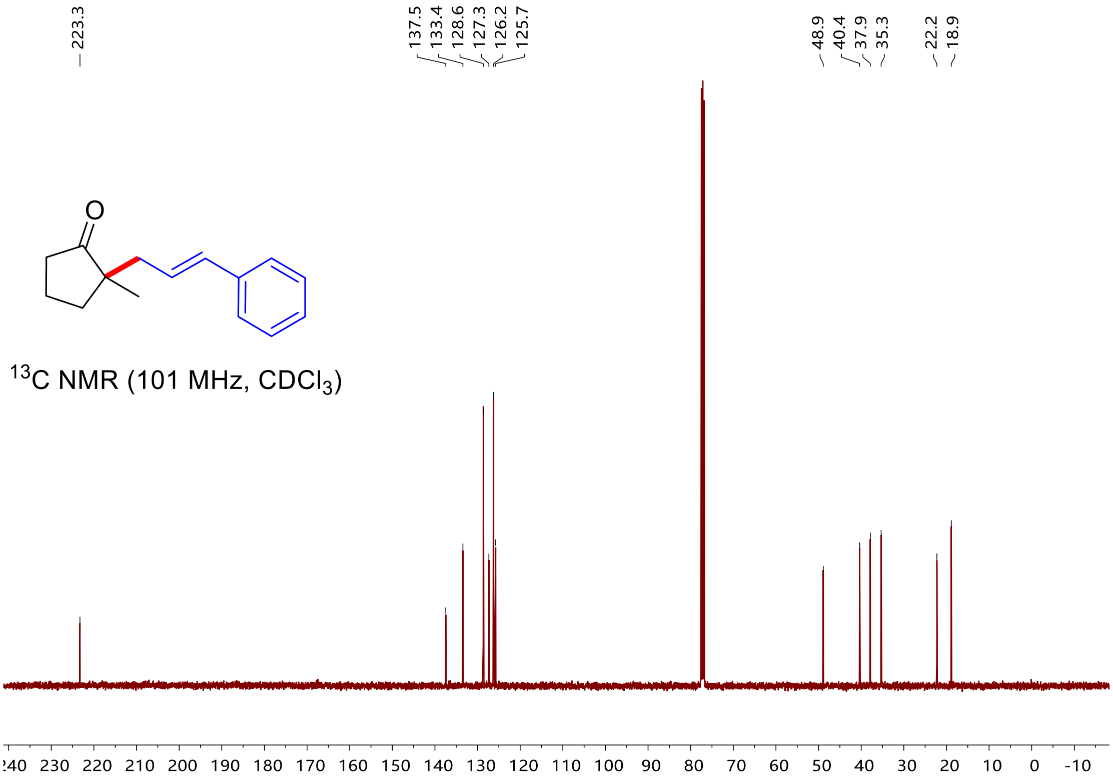


**Supplementary Fig. 99.** $^{13}$C NMR spectrum of compound **6a**.

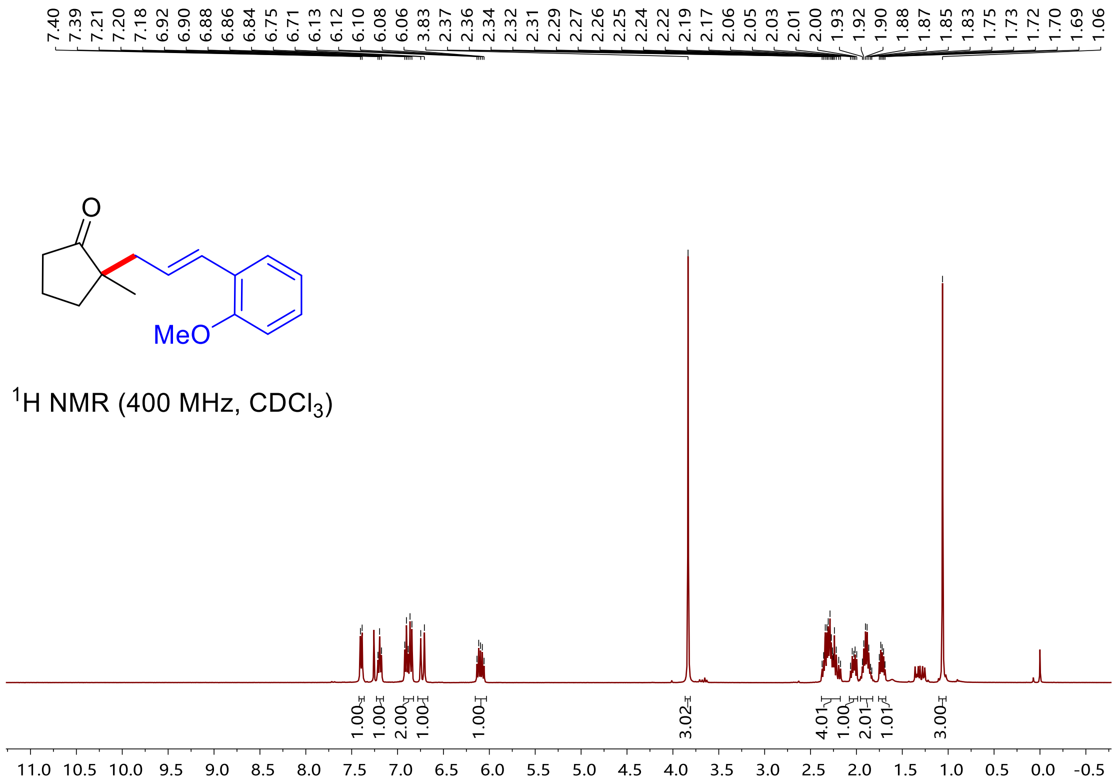


**Supplementary Fig. 100.** $^{1}$H NMR spectrum of compound **6c**.

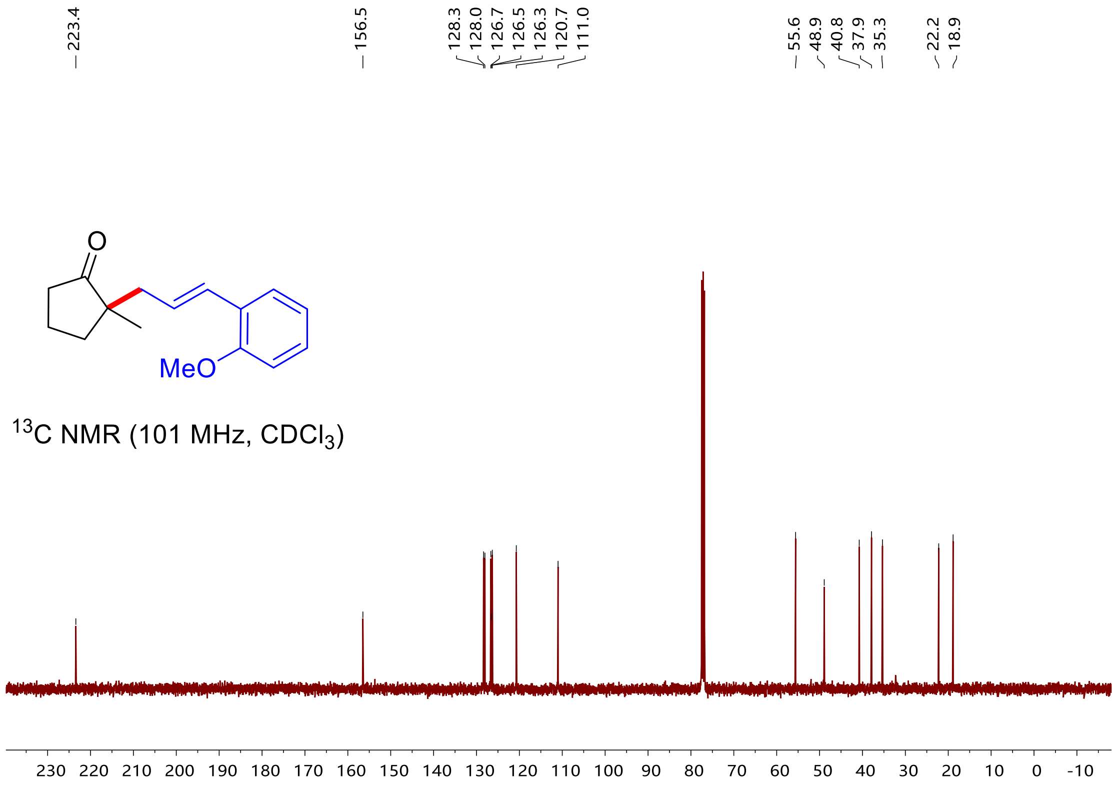


**Supplementary Fig. 101.** $^{13}$C NMR spectrum of compound **6c**.

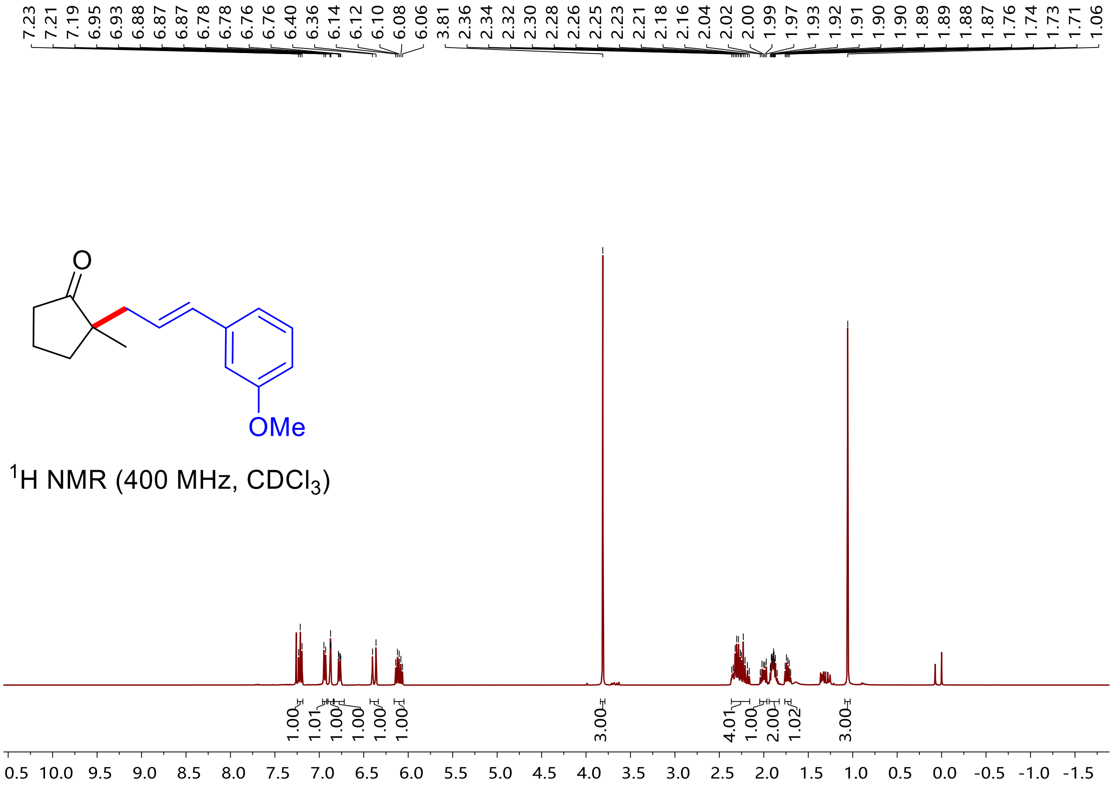


**Supplementary Fig. 102.** $^{1}$H NMR spectrum of compound **6d**.

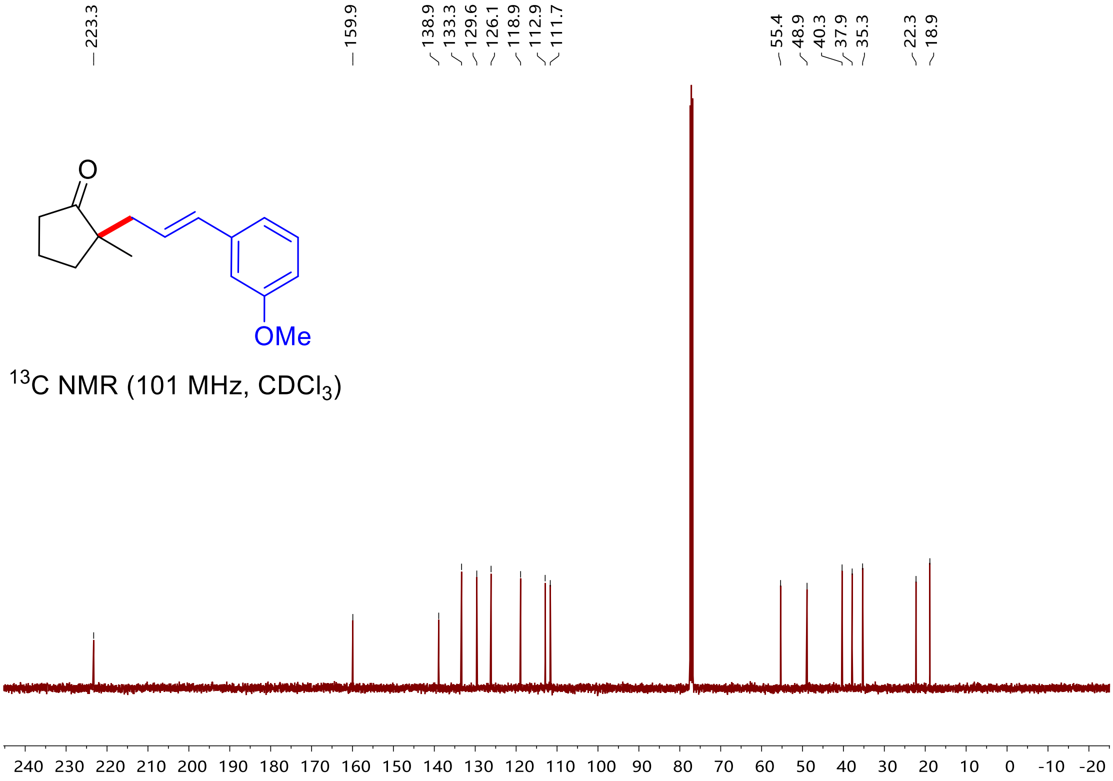


**Supplementary Fig. 103.** $^{13}$C NMR spectrum of compound **6d**.

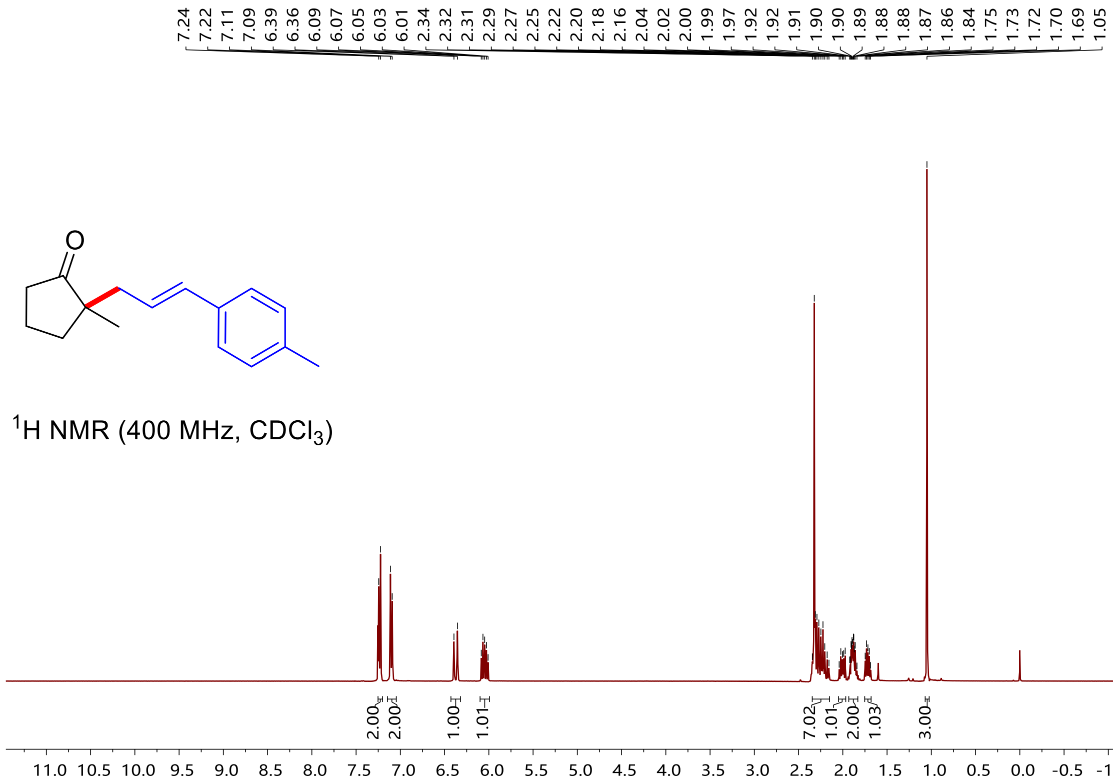


**Supplementary Fig. 104.** $^{1}H$ NMR spectrum of compound **6e**.

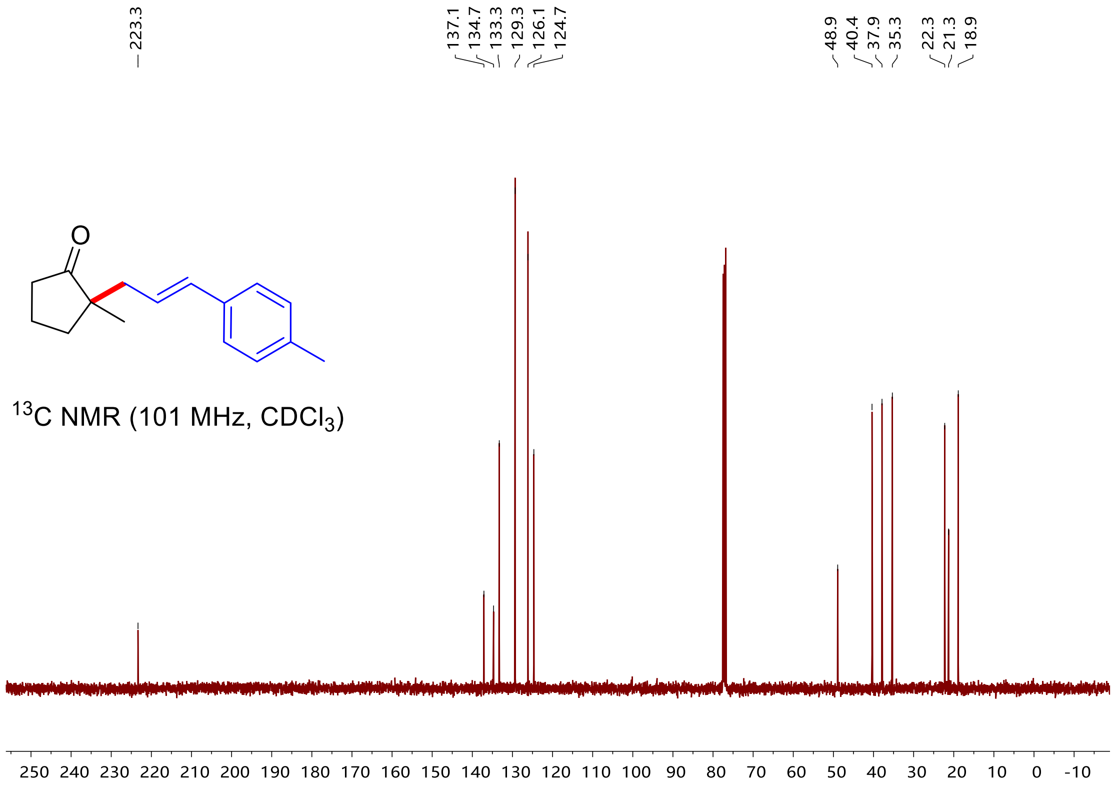


**Supplementary Fig. 105.** $^{13}C$ NMR spectrum of compound **6e**.

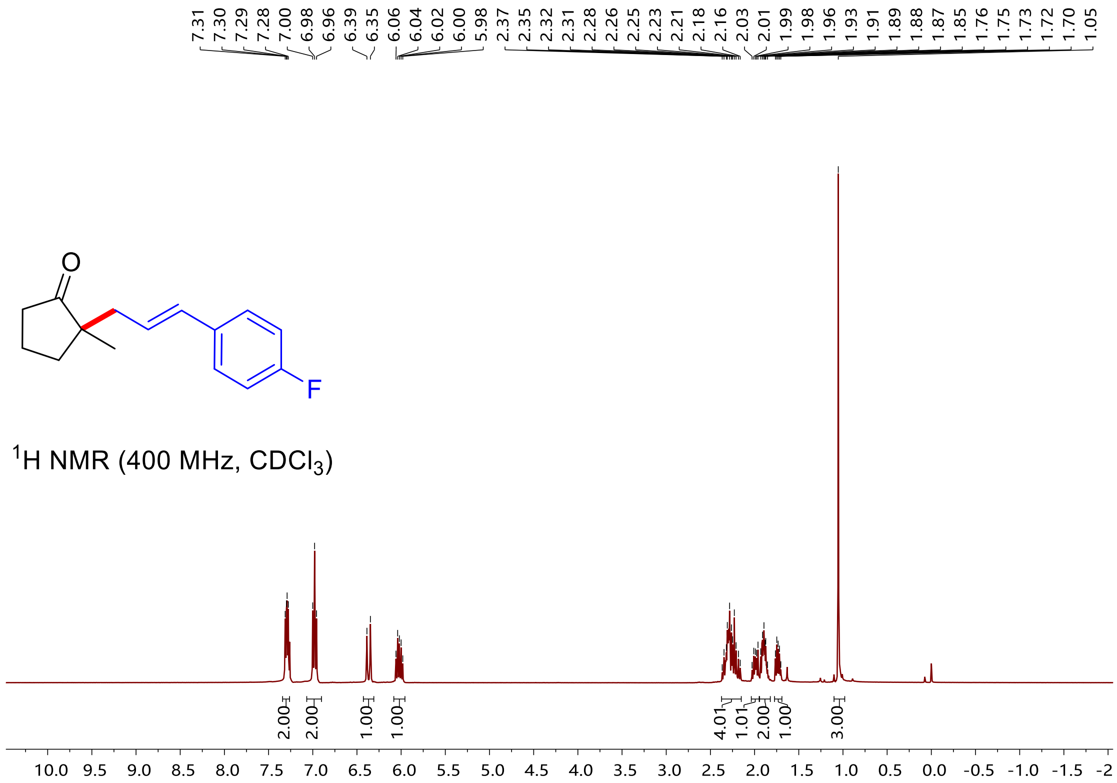


**Supplementary Fig. 106.** $^{1}$H NMR spectrum of compound **6f**.

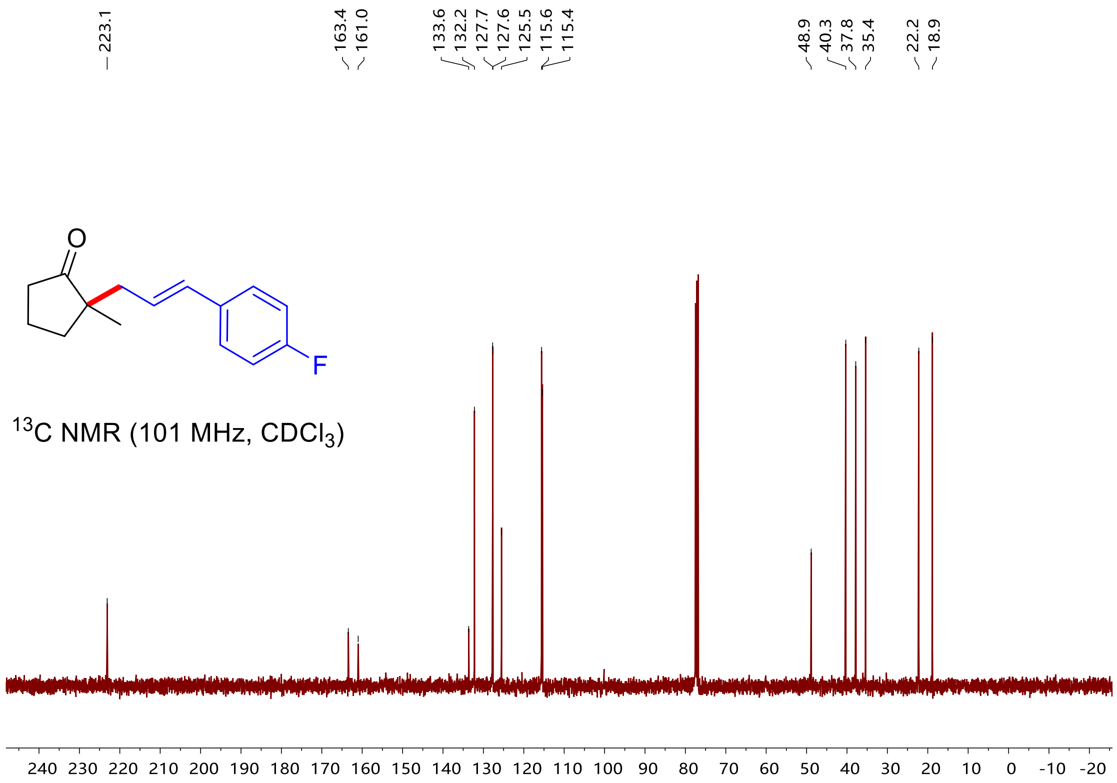


**Supplementary Fig. 107.** $^{13}$C NMR spectrum of compound **6f**.

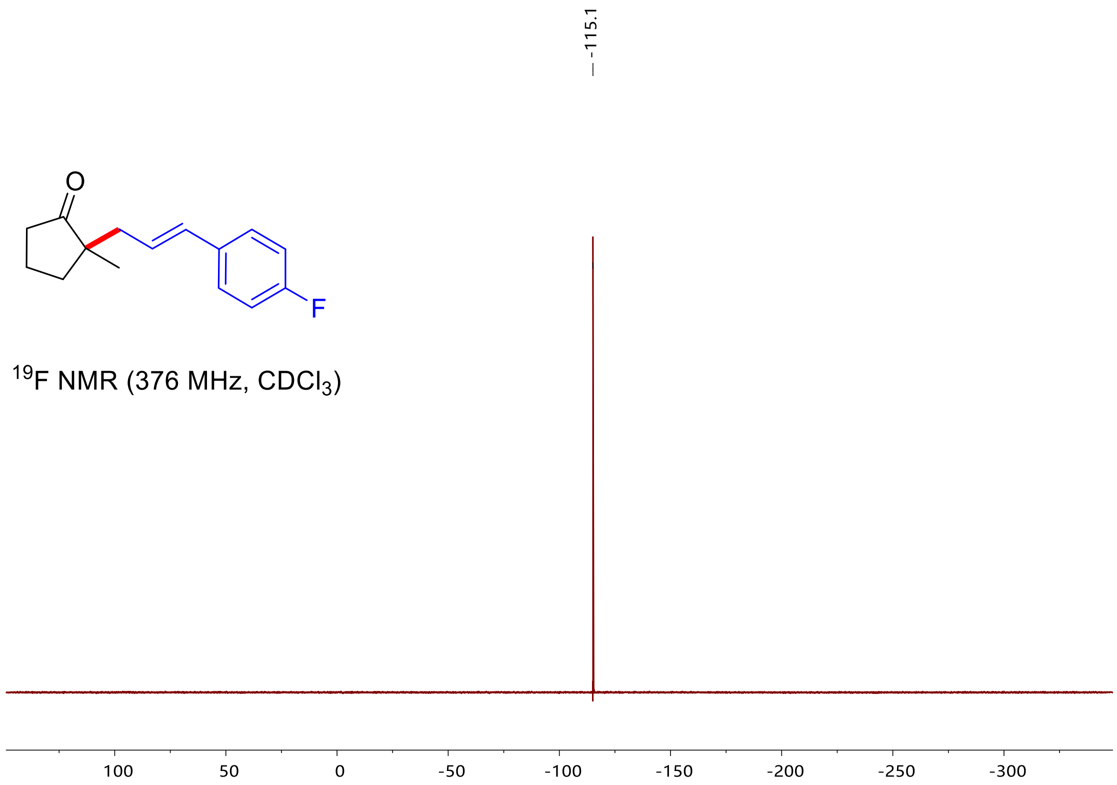


**Supplementary Fig. 108.** $^{19}$F NMR spectrum of compound **6f**.

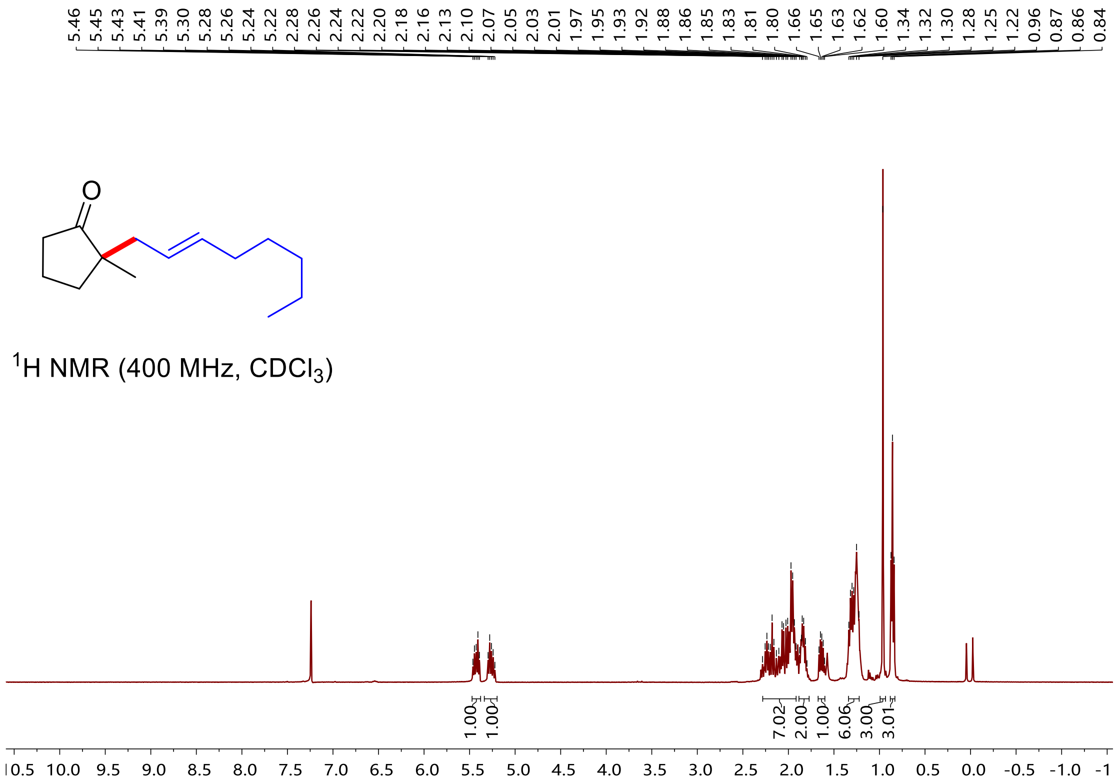


**Supplementary Fig. 109.** $^{1}$H NMR spectrum of compound **6h**.

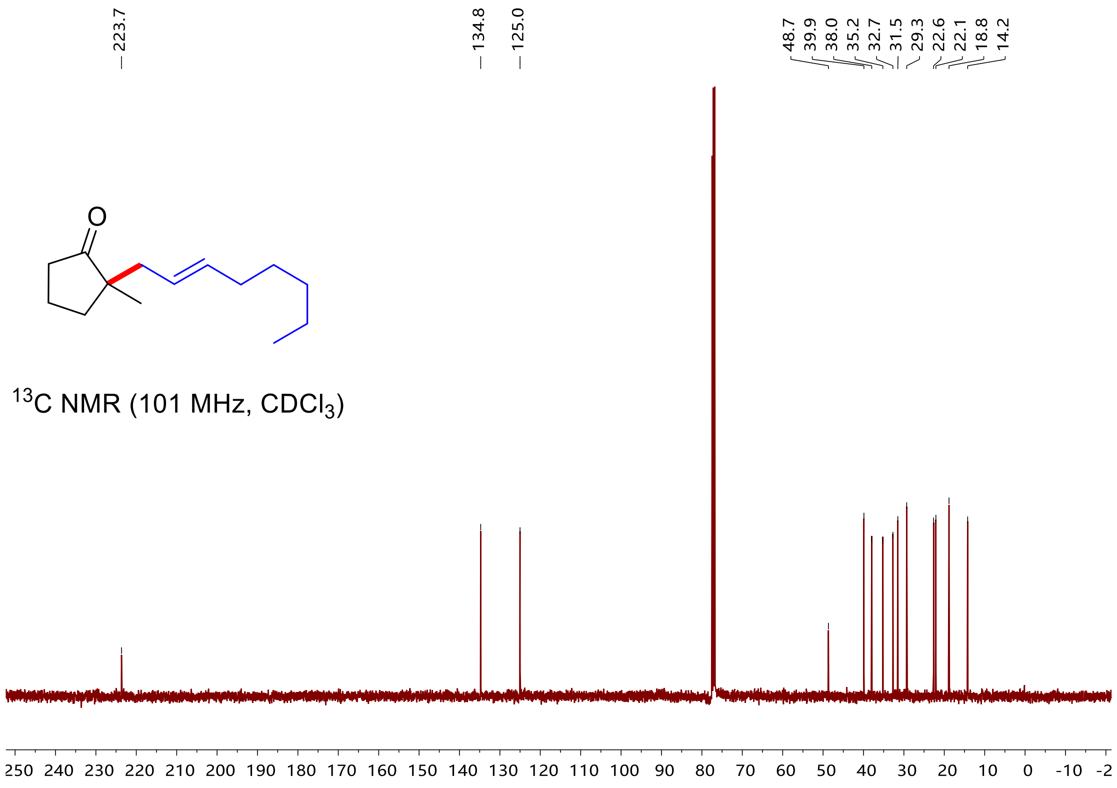


**Supplementary Fig. 110.** $^{13}$C NMR spectrum of compound **6h**.

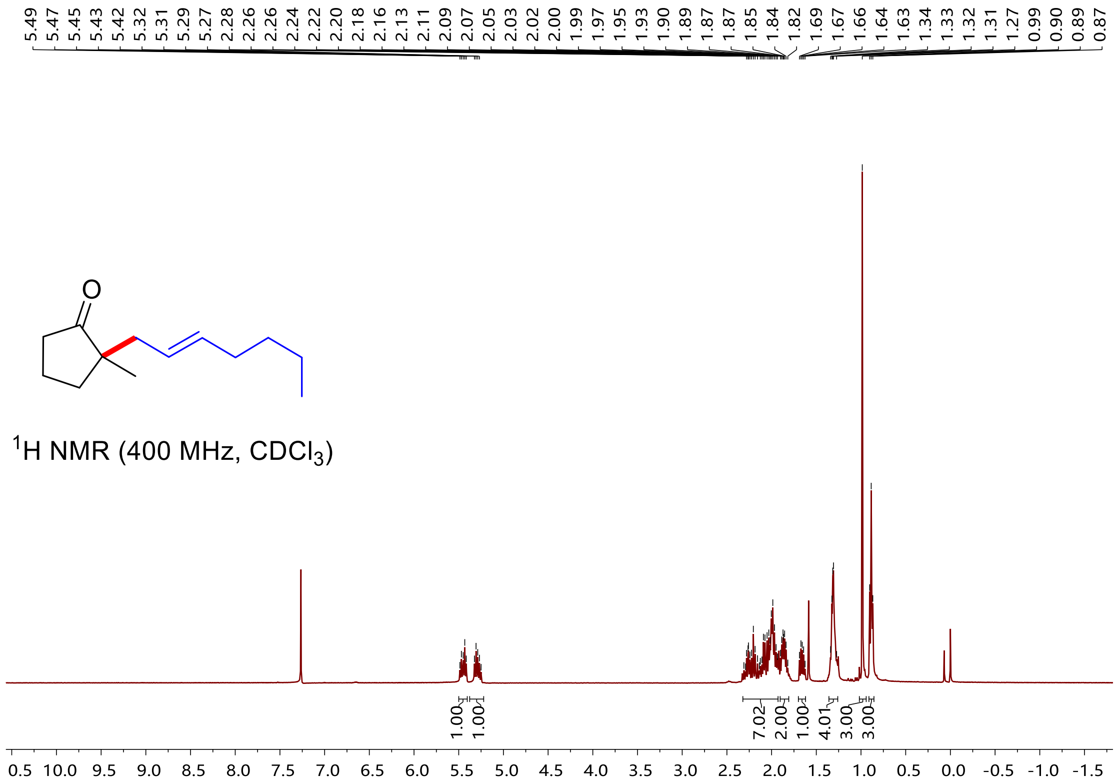


**Supplementary Fig. 111.** $^1$H NMR spectrum of compound **6i**.

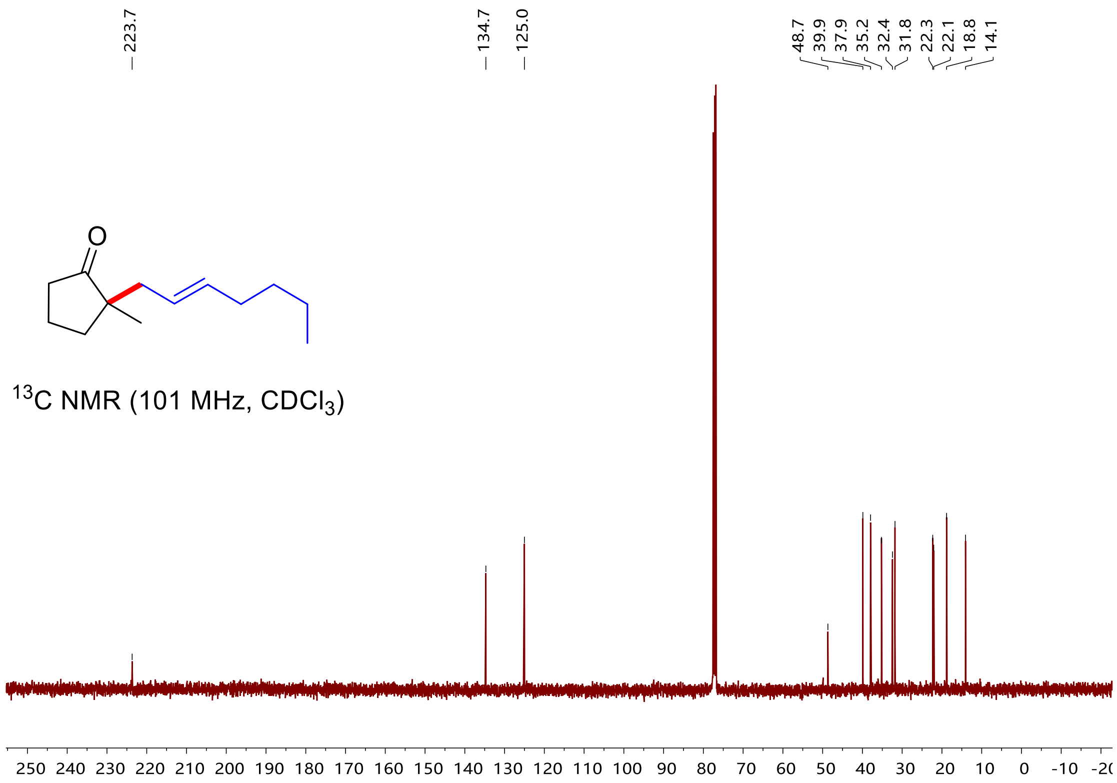


**Supplementary Fig. 112.** $^{13}$C NMR spectrum of compound **6i**.

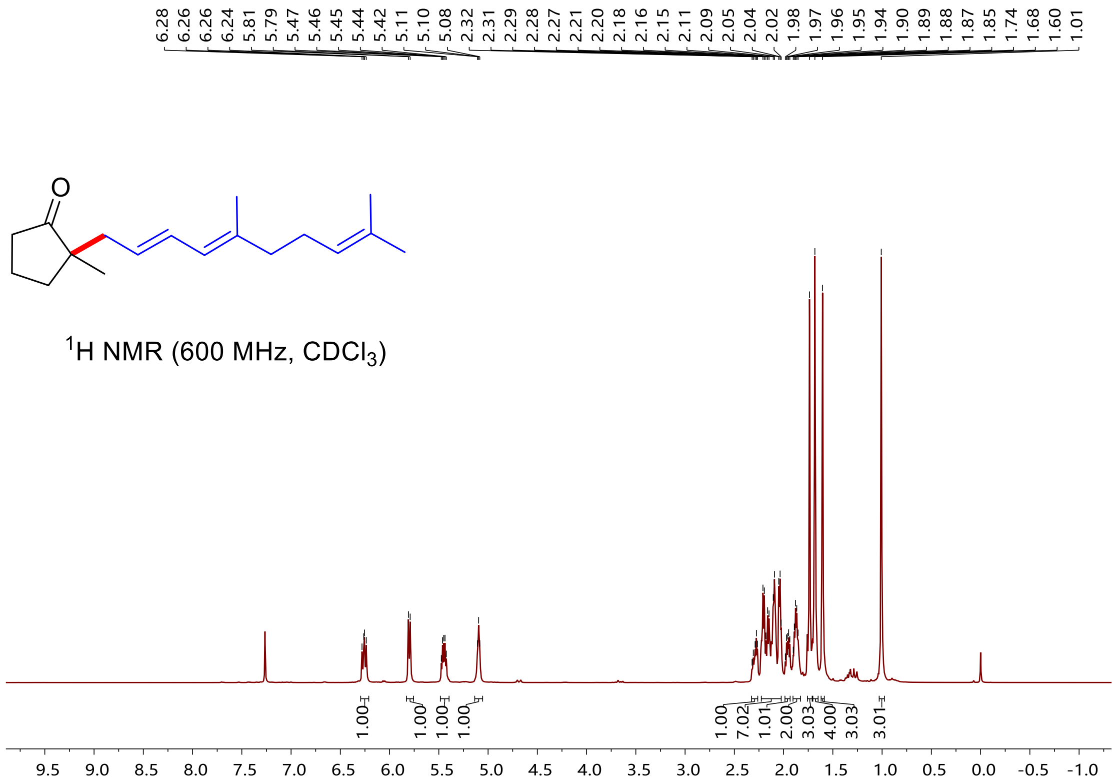


**Supplementary Fig. 113.** $^1$H NMR spectrum of compound **6j**.

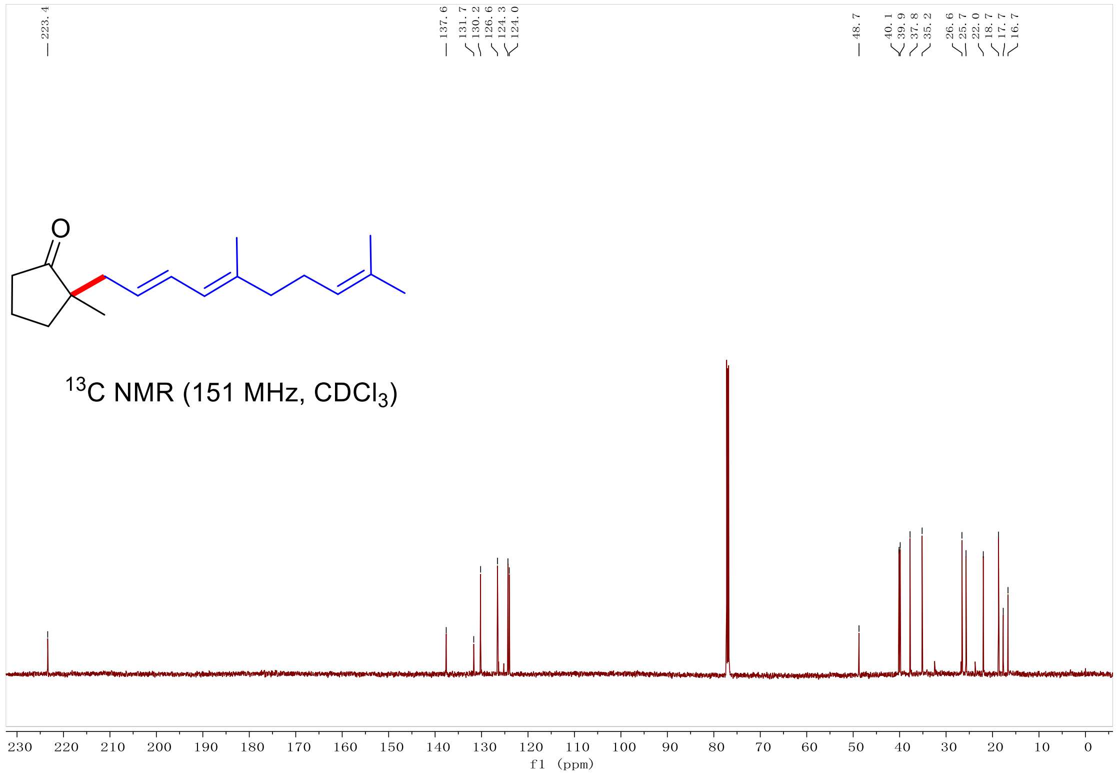


**Supplementary Fig. 114.** $^{13}$C NMR spectrum of compound **6j**.

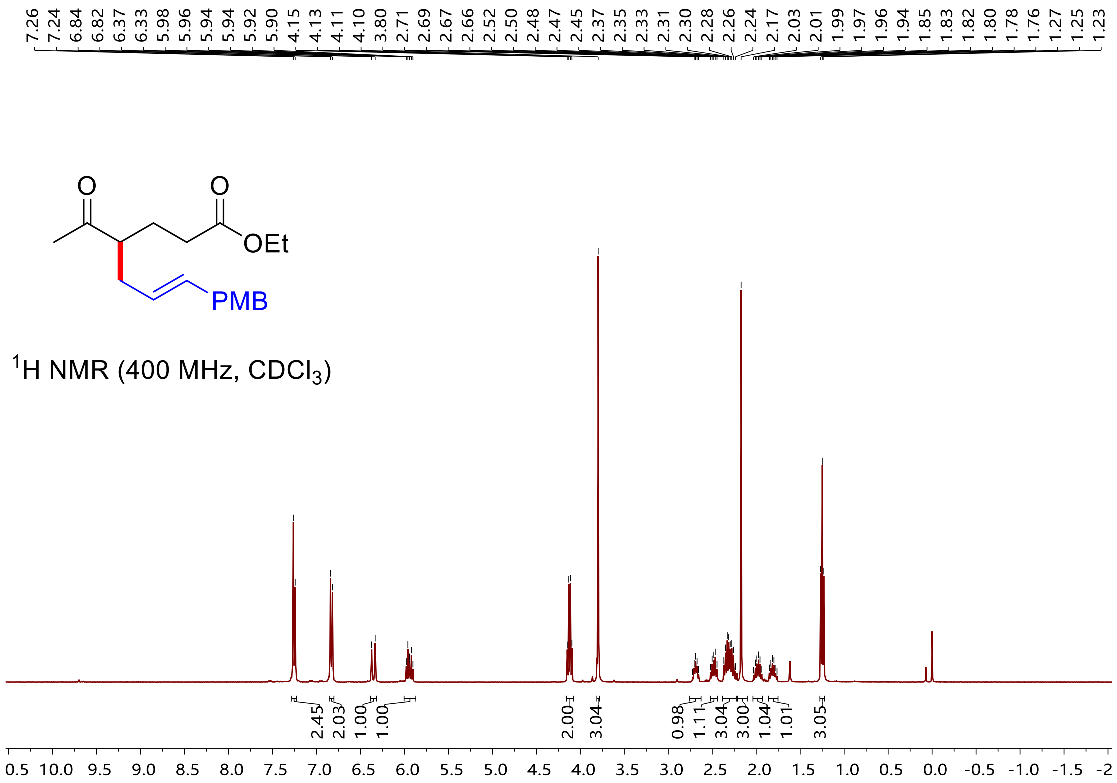


**Supplementary Fig. 115.** $^{1}H$ NMR spectrum of compound **3aa**.

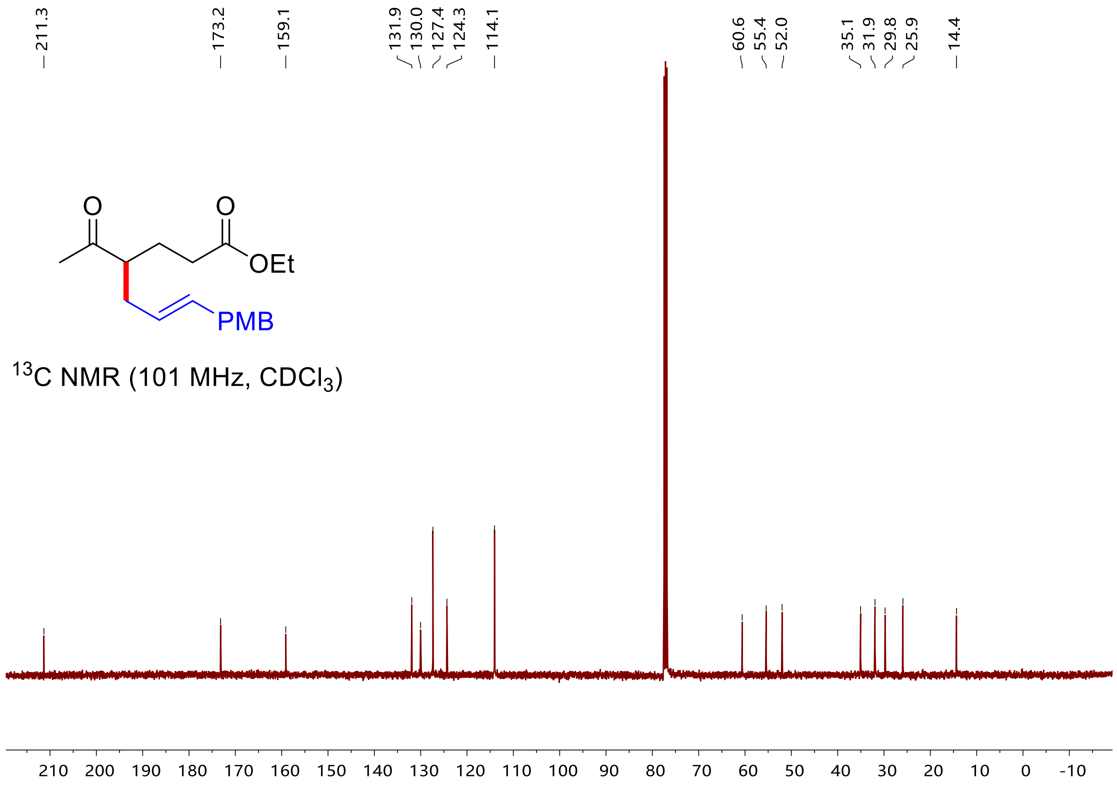


**Supplementary Fig. 116.** $^{13}C$ NMR spectrum of compound **3aa**.

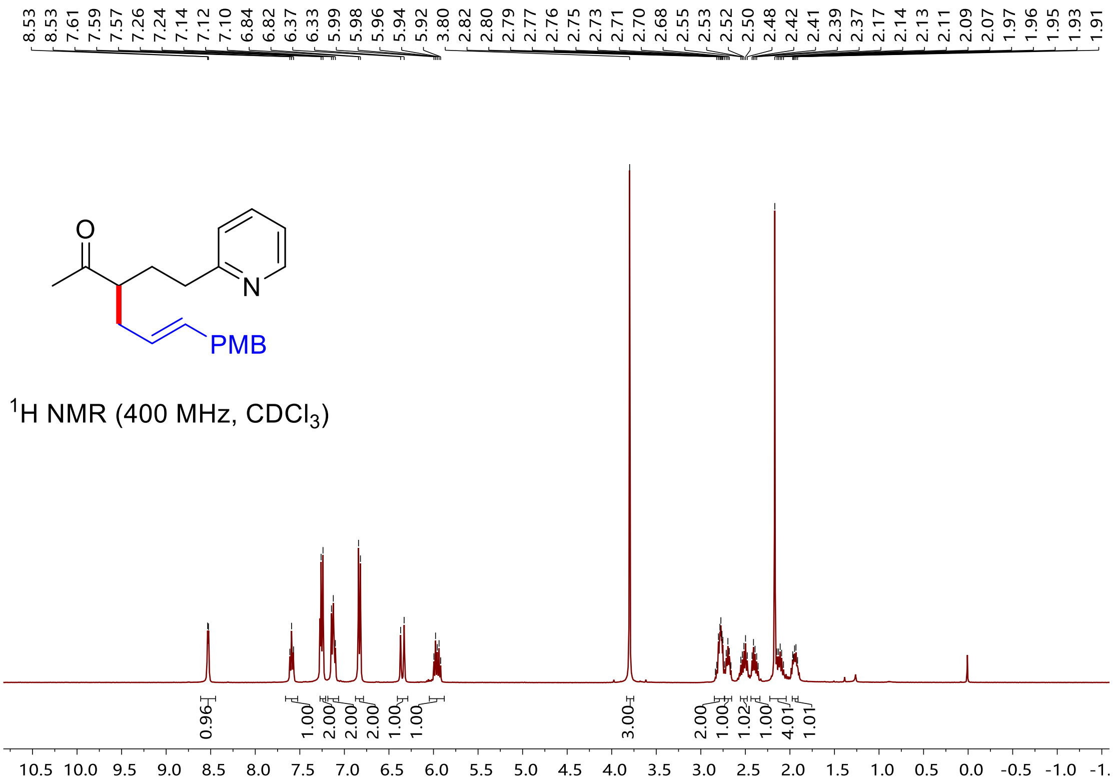


**Supplementary Fig. 117.** $^{1}$H NMR spectrum of compound **3ab**.

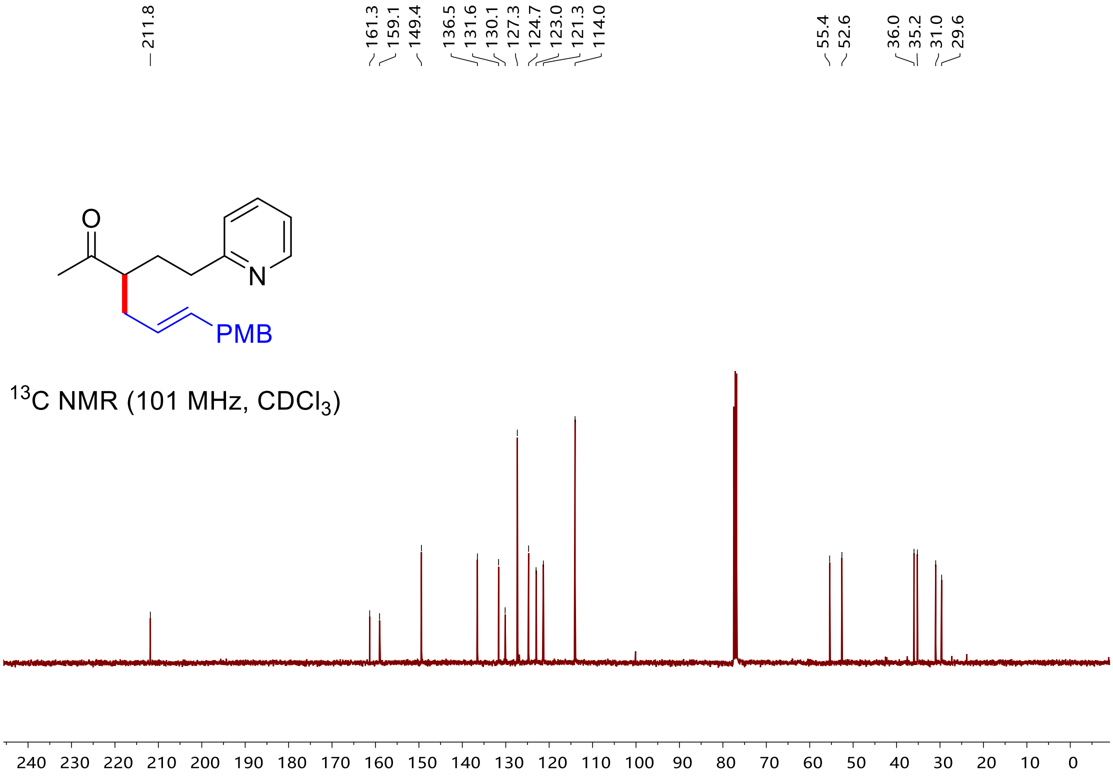


**Supplementary Fig. 118.** $^{13}$C NMR spectrum of compound **3ab**.

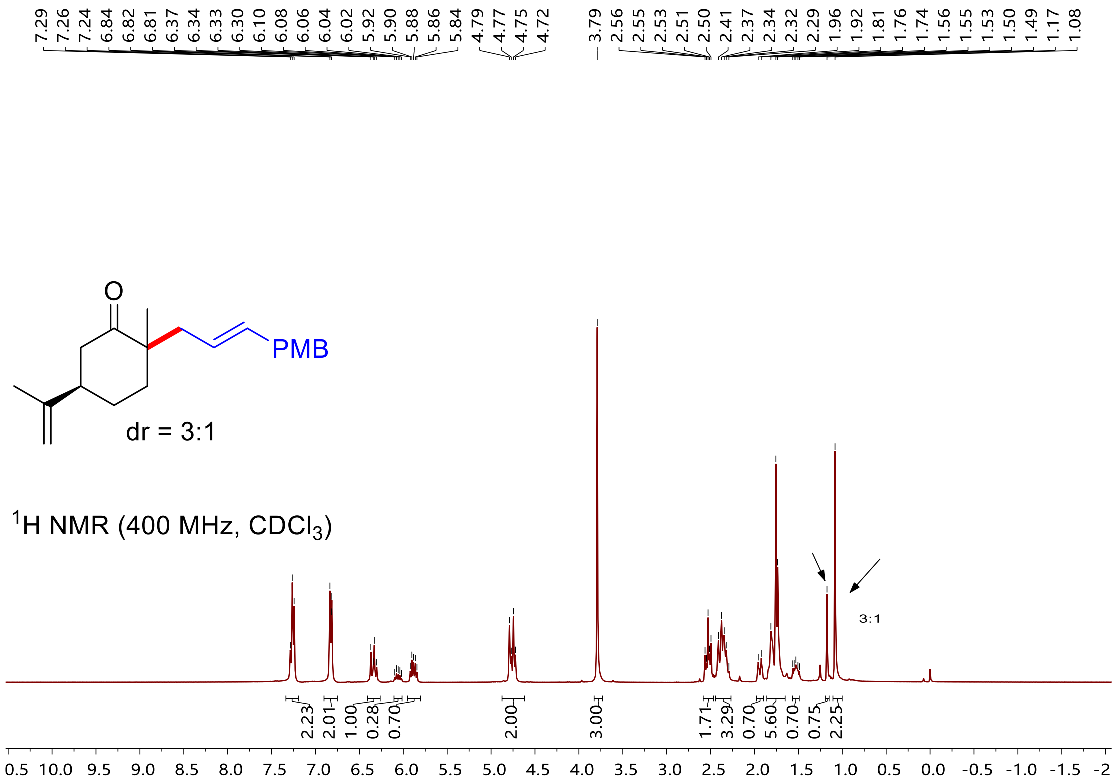


**Supplementary Fig. 119.** $^1$H NMR spectrum of compound **3ac**.

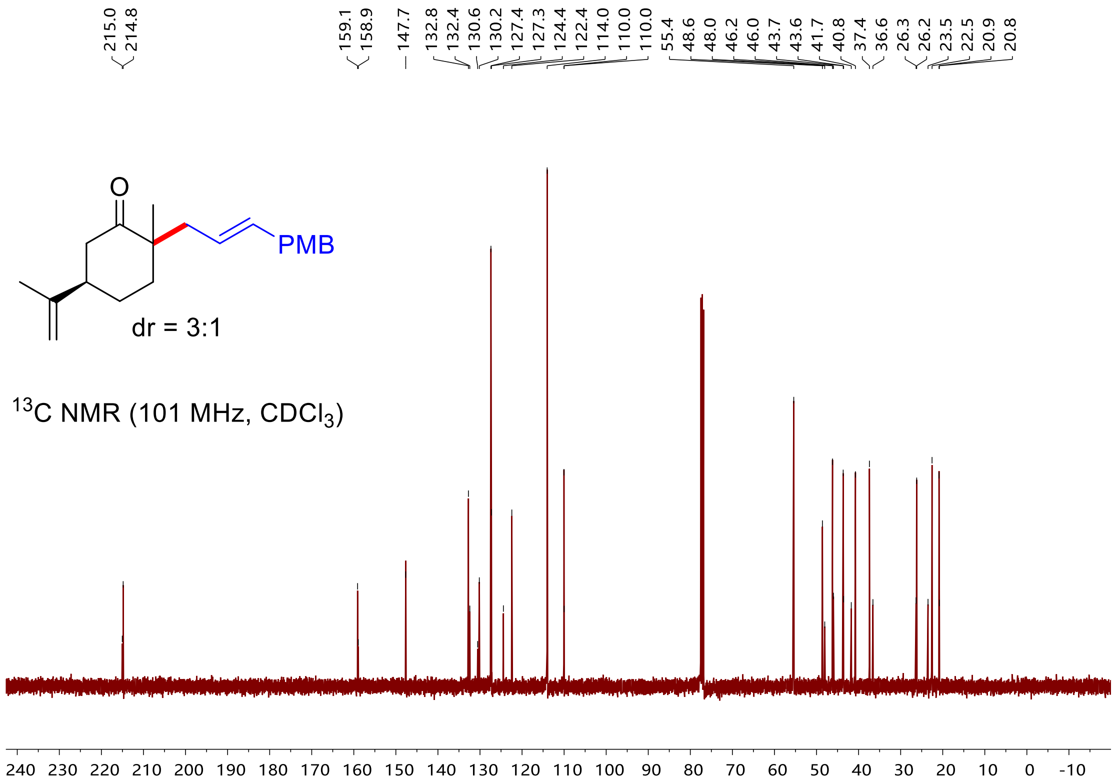


**Supplementary Fig. 120.** $^{13}$C NMR spectrum of compound **3ac**.

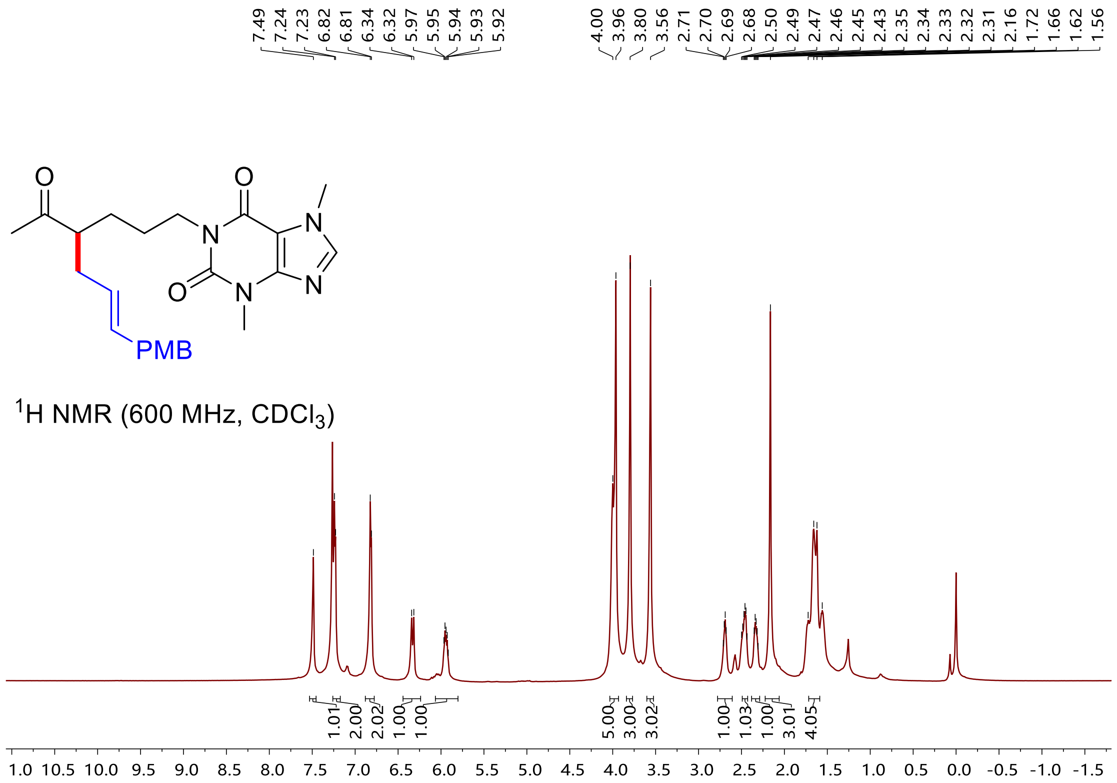

**Supplementary Fig. 121.** $^{1}$H NMR spectrum of compound **3ad**.

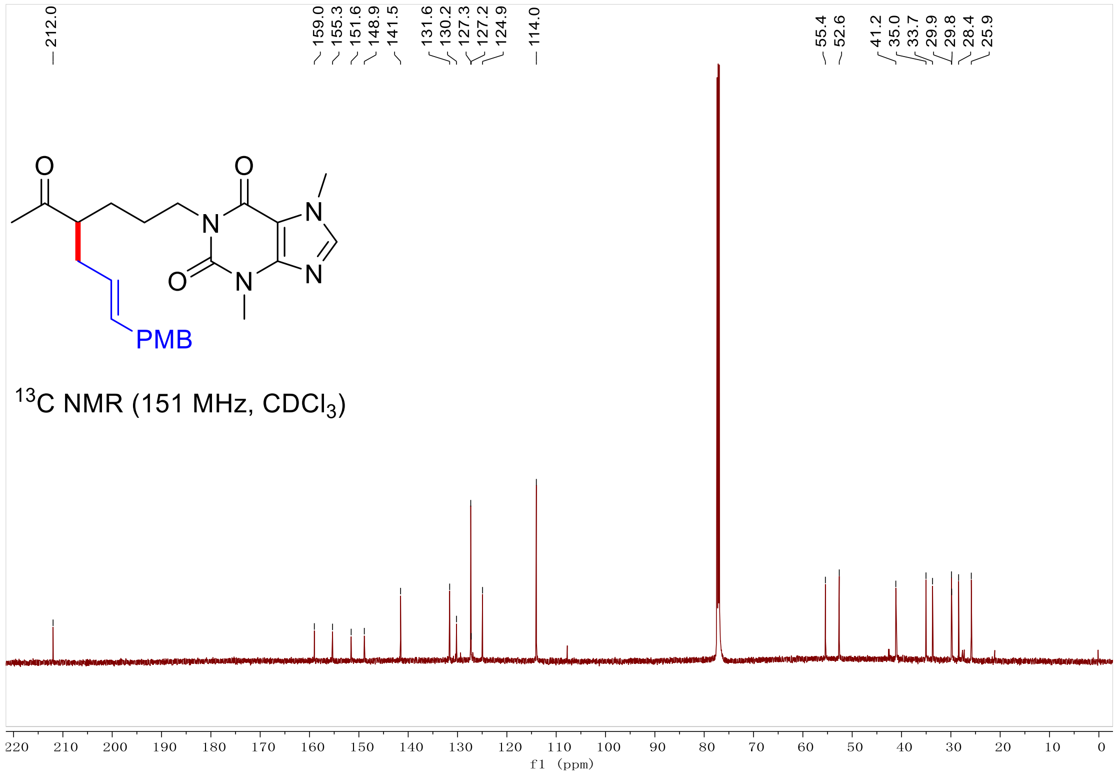

**Supplementary Fig. 122.** $^{13}$C NMR spectrum of compound **3ad**.

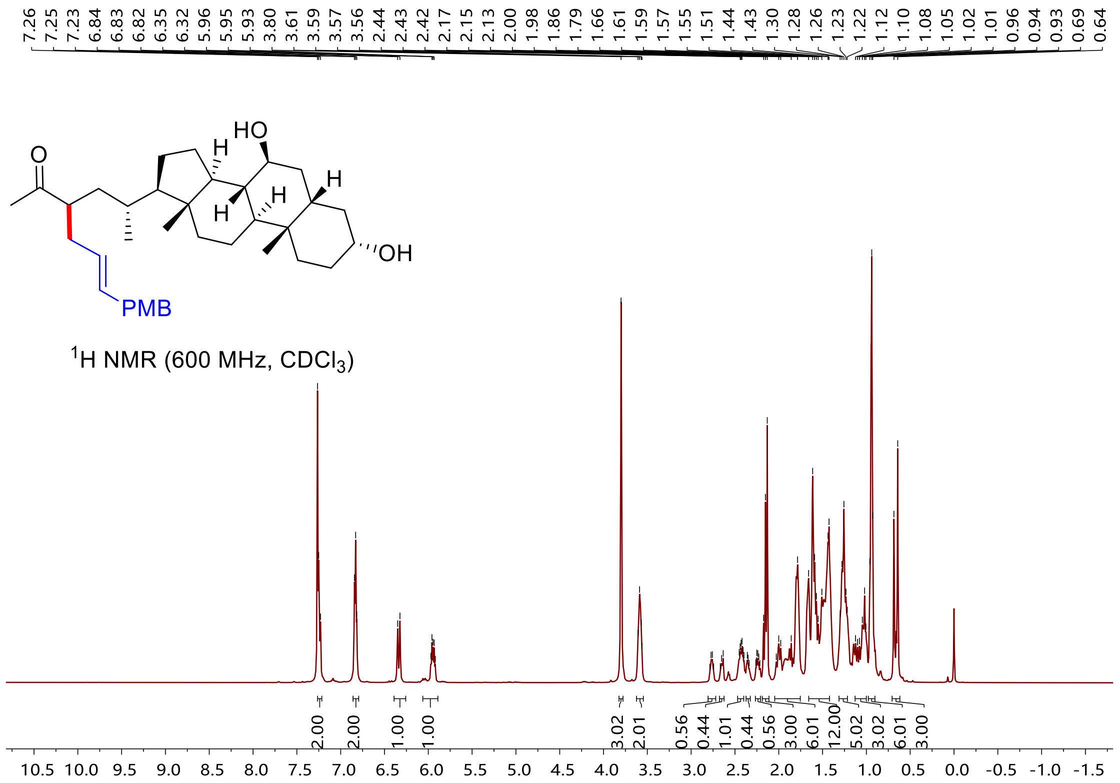


**Supplementary Fig. 123.** $^{1}H$ NMR spectrum of compound **3ae**.

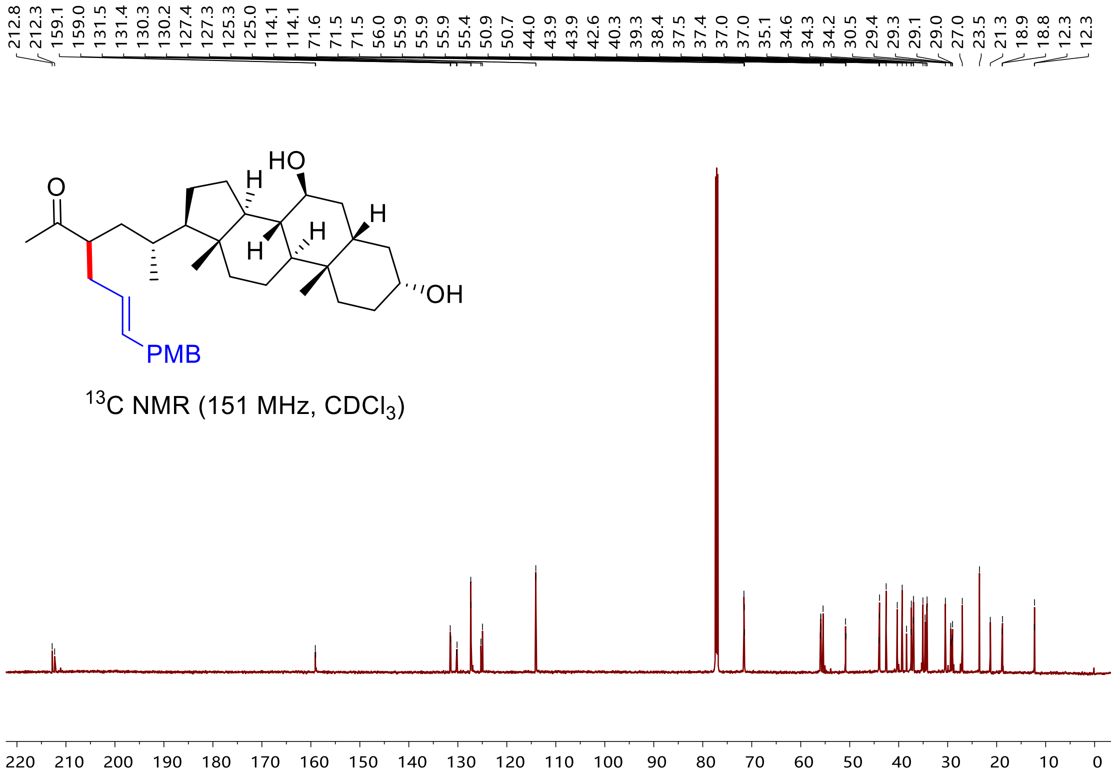


**Supplementary Fig. 124.** $^{13}C$ NMR spectrum of compound **3ae**.

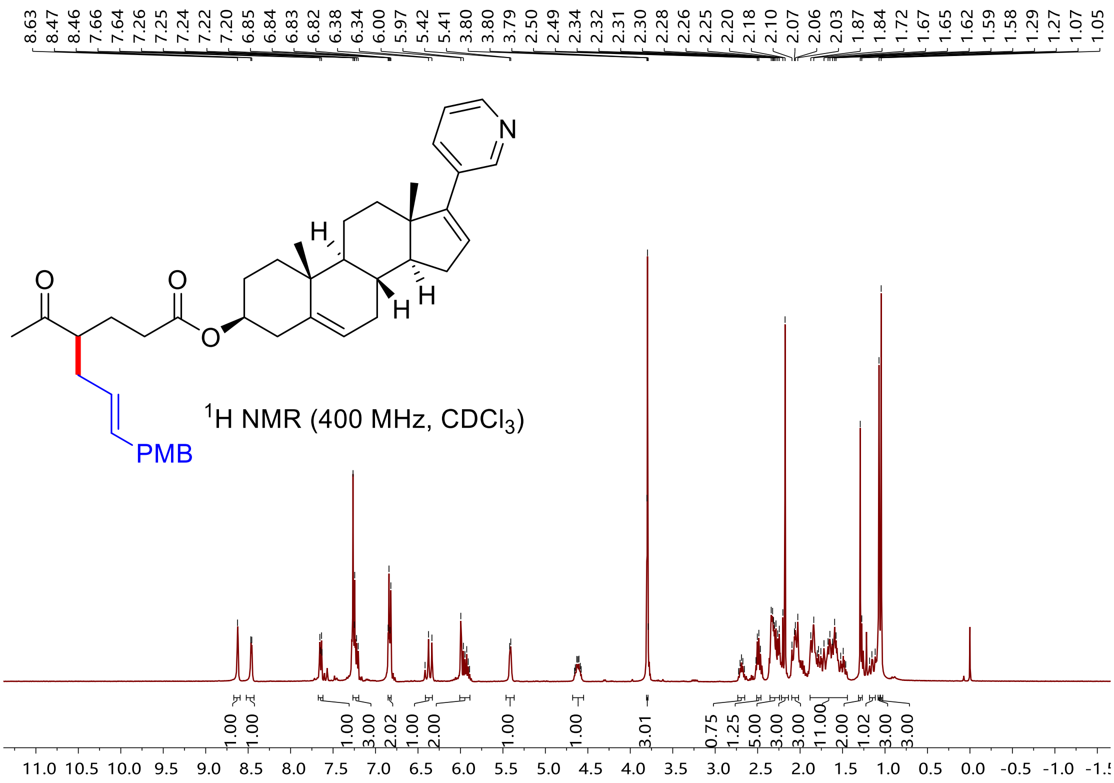


**Supplementary Fig. 125.** $^1$H NMR spectrum of compound **3af**.

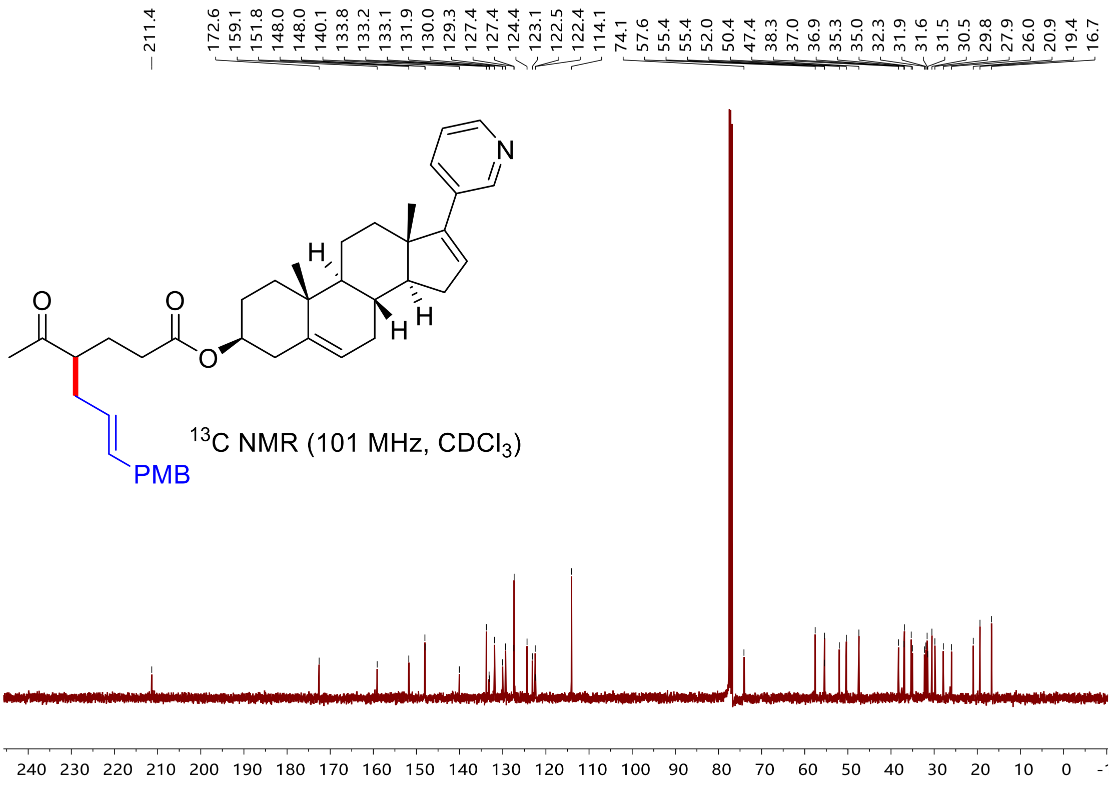


**Supplementary Fig. 126.** $^{13}$C NMR spectrum of compound **3af**.

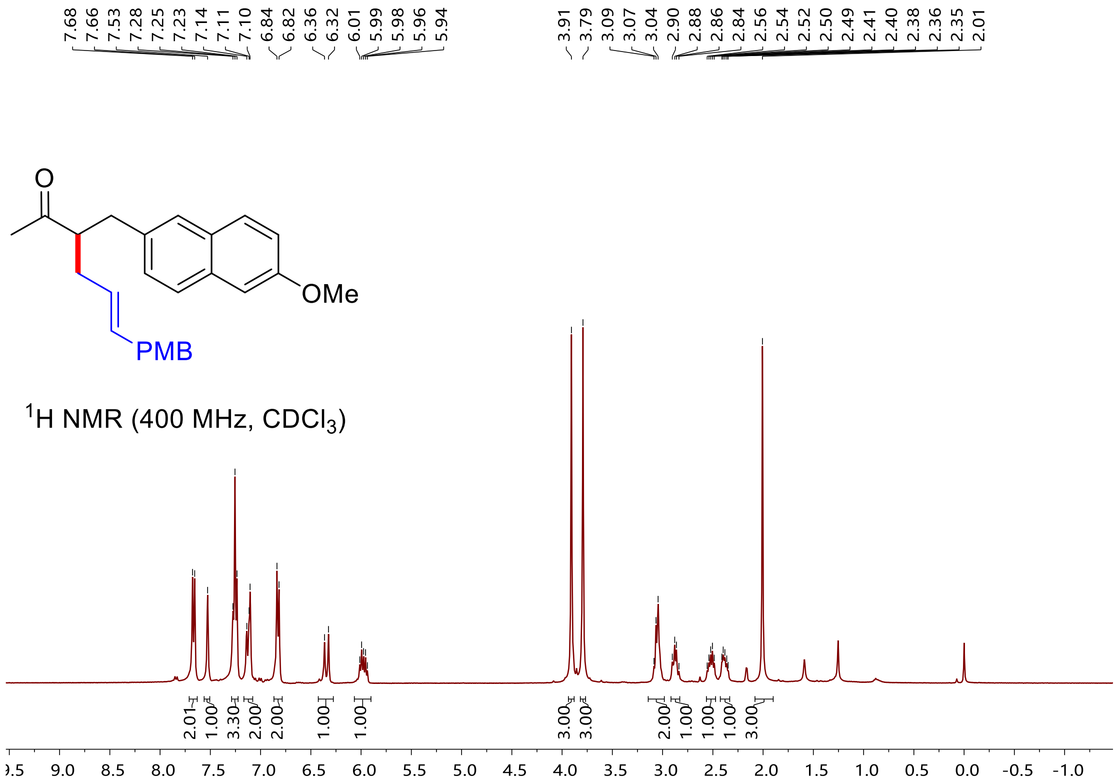


**Supplementary Fig. 127.** $^{1}H$ NMR spectrum of compound **3ag**.

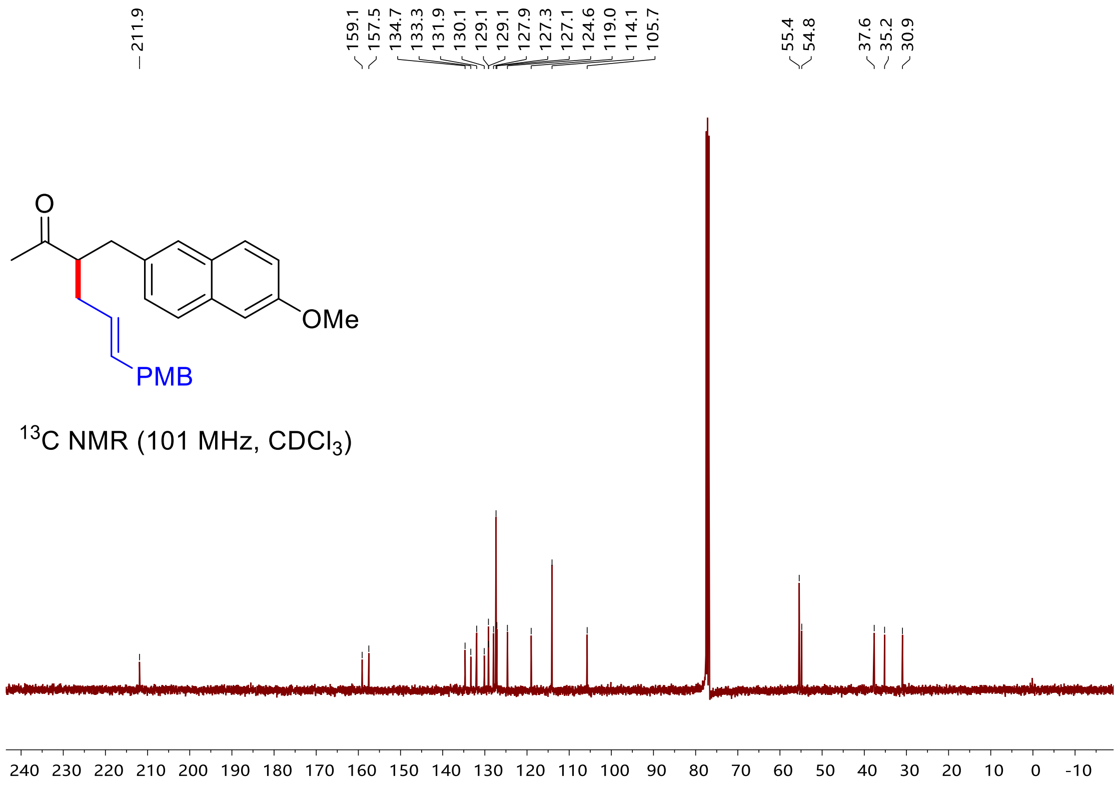


**Supplementary Fig. 128.** $^{13}C$ NMR spectrum of compound **3ag**.

## 4 Supplementary References


1. Li, J., Lin, L., Hu, B., Zhou, P., Huang, T., Liu, X. & Feng, X. Gold(I)/chiral *N*,*N*′-dioxide–nickel(II) relay catalysis for asymmetric tandem intermolecular hydroalkoxylation/claisen rearrangement. *Angew. Chem. Int. Ed.* **56**, 885–888 (2017).
2. Huang, X. & Sun, A. Unique regio- and stereoselectivity in pd-catalyzed chlorocarbonylation reaction of 2-phenylethynyl selenides and 2-alkylethynyl selenides. highly stereoselective synthesis of 2-seleno-3-chloroacrylates. *Angew. Chem. Int. Ed.* **56**, 885–888 (2017).
3. Spoehrle, S. S. M., West, T. H., Taylor, J. E., Slawin, A. M. Z. & Smith, A. D. Tandem palladium and isothiourea relay catalysis: enantioselective synthesis of α-amino acid derivatives via allylic amination and [2,3]-sigmatropic rearrangement. *J. Am. Chem. Soc.* **139**, 11895–11902 (2017).
4. Zhang, F.-H., Zhang, F.-J., Li, M.-L., Xie, J.-H. & Zhou, Q.-L. Enantioselective hydrogenation of dialkyl ketones. *Nat. Catal*. **3**, 621–627 (2020).
5. Chen, P.-A., Setthakarn, K. & May, J. A. A binaphthyl-based scaffold for a chiral dirhodium(II) biscarboxylate ligand with α-quaternary carbon centers. *ACS Catal.* **7**, 6155–6161 (2017).
6. Morrill, C., Jensen, C., Just-Baringo, X., Grogan, G., Turner, N. J. & Procter, D. J. Biocatalytic conversion of cyclic ketones bearinga-quaternary stereocenters into lactonesin an enantioselective radical approach to medium-sized carbocycles. *Angew. Chem. Int. Ed.* **57**, 3692–3696 (2018).
7. Gaussian 16 Rev. A.03 (Wallingford, CT, 2016).
8. Becke, A. D. Density-functional thermochemistry. III. The role of exact exchange. *J. Chem. Phys.* **98**, 5648–5652 (1993).
9. Becke, A. D. A new mixing of Hartree–Fock and local density-functional theories. *J. Chem. Phys.* **98**, 1372–1377 (1993).
10. Stephens, P. J., Devlin, F. J., Chabalowski, C. F. & Frisch, M. J. Ab Initio calculation of vibrational absorption and circular dichroism spectra using density functional force fields. *J. Phys.Chem.* **98**, 11623–11627 (1994).
11. Andrae, D., Häußermann, U., Dolg, M., Stoll, H. & Preuß, H. Energy-adjustedab initio pseudopotentials for the second and third row transition elements. *Theor. Chem. Acc.* **77**, 123–141 (1990).
12. Hehre, W. J., Ditchfield, R. & Pople, J. A. Self–consistent molecular orbital methods. XII. Further extensions of Gaussian–type basis sets for use in molecular orbital studies of organic molecules. *J. Chem. Phys.* **56**, 2257-2261 (1972).
13. Lee, C., Yang, W. & Parr, R. G. Development of the Colle-Salvetti correlation-energy formula into a functional of the electron density. *Phys. Rev. B* **37**, 785–789 (1988).
14. Marenich, A. V., Cramer, C. J. & Truhlar, D. G. Universal solvation model based on solute electron density and on a continuum model of the solvent defined by the bulk

dielectric constant and atomic surface tensions. *J. Phys. Chem. B* **113**, 6378–6396 (2009).
15. Grimme, S., Antony, J., Ehrlich, S. & Krieg, H. A consistent and accurate ab initio parametrization of density functional dispersion correction (DFT-D) for the 94 elements H-Pu. *J. Chem. Phys.* **132**, 154104 (2010).
16. Adamo, C. & Barone, V. Toward reliable density functional methods without adjustable parameters: The PBE0 model. *J. Chem. Phys. 110*, 6158–6170(1999).
17. Weigend, F. & Ahlrichs, R. Balanced basis sets of split valence, triple zeta valence and quadruple zeta valence quality for H to Rn: Design and assessment of accuracy. *PCCP* **7**, 3297–3305, doi:10.1039/B508541A (2005).
18. Weigend, F. Accurate Coulomb-fitting basis sets for H to Rn. *PCCP* **8**, 1057–1065, doi:10.1039/B515623H (2006).
19. Fukui, K. The path of chemical reactions-the IRC approach. *Acc. Chem. Res.* **14**, 363–368 (1981).
20. CYLview, 1.0b (Universitéde Sherbrooke, 2009).
21. Falivene, L., Credendino, R., Poater, A., Petta, A., Serra, L., Oliva, R., Scarano, V., Cavallo, L. SambVca 2. A web tool for analyzing catalytic pockets with topographic steric maps Organometallics **35**, 2286–2293 (2016).
