# Supplementary material for: Ketone α-alkylation at the more-hindered site
Source: Nat Commun. 2023 Jun 7;14:3326. doi: 10.1038/s41467-023-38741-w (PMC10247815; doi:10.1038/s41467-023-38741-w)
Supplement: Supplementary file 3 — Description of Additional Supplementary Files [file 41467_2023_38741_MOESM3_ESM.docx]

**Description of Additional Supplementary Files**

File Name: Supplementary Data 1

Description: the cartesian coordinates of the optimized structures.
