# Supplementary material for: Ketone α-alkylation at the more-hindered site
Source: Nat Commun. 2023 Jun 7;14:3326. doi: 10.1038/s41467-023-38741-w (PMC10247815; doi:10.1038/s41467-023-38741-w)
Supplement: Supplementary file 4 — Supplementary Data 1 [file 41467_2023_38741_MOESM4_ESM.docx]

**Supplementary Data**

**Cartesian coordinates (in Å) of related structure.**

For transition state structures, one imaginary frequency was observed and given below. For all minimum structures, no imaginary frequency was observed.

**1n**

C -0.52777900 0.16388900 -0.01351500

O -0.41340500 1.38316900 -0.01508400

C -1.88278300 -0.50337800 0.02309900

H -2.67758800 0.23771800 -0.09221500

H -1.96258700 -1.26326900 -0.76319500

H -2.00833800 -1.02156600 0.98279100

C 0.67921000 -0.75276200 -0.04010200

H 0.57161900 -1.47304300 0.78314200

H 0.60780100 -1.35469800 -0.95789400

C 2.01494500 -0.02183400 0.03273800

H 2.09793600 0.55823200 0.95832400

H 2.84487500 -0.73590300 0.00308500

H 2.13196500 0.67169000 -0.80667900

**1n_IPA**

C -1.96473300 -0.42234800 0.19115300

O -1.05781500 -0.59699500 1.00231200

C -2.97509400 -1.50651700 -0.07826300

H -2.65572200 -2.45125300 0.36777600

H -3.94039100 -1.21045600 0.35345900

H -3.13189700 -1.62931400 -1.15623900

C -2.12555900 0.86599700 -0.58448500

H -1.79063500 0.64494300 -1.60993400

H -3.19861200 1.07922400 -0.67229100

C -1.36596000 2.05902400 -0.01264100

H -0.28854600 1.87750200 0.01050000

H -1.54933900 2.94955700 -0.62310700

H -1.69207000 2.27692000 1.01081200

C 1.62793400 -0.43852900 -1.32672600

C 2.22739900 -0.46078400 0.07880000

H 0.56556900 -0.70480800 -1.30292400

H 1.72466600 0.56147900 -1.76739200

H 2.13990300 -1.15484300 -1.98087200

C 3.70462000 -0.09281800 0.07800500

H 2.11511300 -1.47650000 0.49168400

H 4.10824100 -0.11300000 1.09672400

H 4.28010700 -0.79437800 -0.53595200

H 3.84525700 0.91703400 -0.32729500

O 1.56615700 0.47338600 0.94256400

H 0.62998200 0.19260700 1.01098800

**Ea**

C -1.81530500 -0.60411200 0.00001000

C -0.46105200 0.11524500 -0.00000100

C 0.67506100 -0.66526100 0.00000300

C 2.06445000 -0.08962400 -0.00000500

O -0.51847300 1.40857900 -0.00000900

H -1.73583300 -1.69829700 0.00001500

H -2.40301900 -0.30179600 0.87945700

H -2.40302600 -0.30180500 -0.87943600

H 0.57265000 -1.75026800 0.00001400

H 2.00459800 1.00608000 -0.00005600

H 2.66673200 -0.37997700 0.87955700

H 2.66675200 -0.38006200 -0.87952400

**Ea′**

C -0.85003800 1.31184700 -0.00002500

C -0.56907400 -0.19685700 -0.00007500

C 0.72520400 -0.67304800 -0.00007300

C 2.02054400 0.09465300 -0.00005700

O -1.63422300 -0.93480900 0.00018600

H 0.03192500 1.96038100 -0.00064100

H -1.45967700 1.56654800 -0.87888000

H -1.45858700 1.56679000 0.87952500

H 0.82159800 -1.76361000 0.00006600

H 2.65548200 -0.12525300 0.87793500

H 2.65580200 -0.12587200 -0.87765400

H 1.86742100 1.17991100 -0.00045600

**Eb**

C 1.65459800 -0.77398000 -0.24138200

C 0.63828900 0.09640500 0.08800200

C -0.71003400 -0.51202600 0.51701700

C -1.85974000 -0.02989400 -0.37366900

O 0.69194200 1.38452100 0.07029000

H 2.62999600 -0.39779800 -0.55258100

H 1.52601200 -1.85309500 -0.19955900

H -0.67102300 -1.60916900 0.51911300

H -0.91443200 -0.19615600 1.55219000

H -2.83473800 -0.38956200 -0.02104400

H -1.88434100 1.06514300 -0.39381700

H -1.72568300 -0.37855200 -1.40643000

**Eb′**

C -0.72916300 1.36758900 -0.00014000

C -0.69593800 -0.00950000 0.00001700

C 0.66491400 -0.73539700 0.00004000

C 1.94386700 0.10293900 -0.00004800

O -1.71856100 -0.79611200 0.00015100

H -1.68790100 1.88719700 -0.00014900

H 0.16539100 1.98243700 -0.00023100

H 0.66770900 -1.40664600 0.87265200

H 0.66766800 -1.40676500 -0.87248000

H 2.83519000 -0.53774900 -0.00003400

H 1.99914800 0.74826500 -0.88495400

H 1.99919800 0.74837600 0.88477400

**Ea_IPA**

C 1.01018400 1.69777000 -0.43311600

C 1.69808100 0.38945700 -0.06263400

C 3.05957800 0.30747500 -0.16173300

C 3.83943600 -0.93436900 0.16867300

O 0.90902800 -0.58553600 0.32689700

H 1.71652000 2.49248900 -0.69901400

H 0.33214800 1.54331300 -1.28476400

H 0.38657200 2.04980100 0.39944600

H 3.61322500 1.17974400 -0.50687900

H 3.15751900 -1.71767600 0.51971800

H 4.39180400 -1.34519200 -0.69388300

H 4.59233400 -0.77618000 0.95928100

C -2.22737700 -1.37131800 -0.77198500

C -2.30103100 0.03160500 -0.16448100

H -1.18682700 -1.62603100 -1.00060800

H -2.61464100 -2.11415000 -0.06246900

H -2.81214100 -1.43833000 -1.69819600

C -3.73038800 0.42712000 0.18835100

H -1.92281600 0.74723200 -0.91665400

H -3.75483200 1.43333200 0.62372100

H -4.37390400 0.42000400 -0.69927400

H -4.14898700 -0.27189600 0.92392400

O -1.52095100 0.11925000 1.02163400

H -0.57149200 -0.13261600 0.76896100

**Eb_IPA**

C -3.18643700 -1.33996100 -0.24008000

C -2.08917900 -0.57266700 0.04231400

C -2.30351700 0.86741000 0.51333200

C -1.69710200 1.89673500 -0.44967200

O -0.85058100 -0.96866300 -0.07656700

H -3.07276900 -2.36629400 -0.58822000

H -4.19703700 -0.95541900 -0.13212700

H -3.37317400 1.07102100 0.64723800

H -1.82946800 0.98660800 1.49962100

H -1.88344300 2.92232600 -0.10680100

H -0.61472800 1.75944900 -0.52781200

H -2.13027200 1.79289600 -1.45292200

C 3.38880800 0.89081000 -0.18589600

C 2.17831900 -0.03296300 -0.25344000

H 3.09581800 1.92566500 -0.39897000

H 3.83394000 0.86260800 0.81712400

H 4.15564900 0.59269100 -0.91048200

C 2.55091700 -1.49019600 0.02954700

H 1.75394100 0.02080700 -1.27091200

H 1.65846300 -2.12478500 -0.01521000

H 3.27829500 -1.86898900 -0.69983600

H 2.98686600 -1.58241400 1.03281500

O 1.21605500 0.43343400 0.68831500

H 0.36327200 -0.06934400 0.47588000

**Ea_H**

C -1.82756900 -0.63082000 0.00001200

C -0.46682400 -0.00390000 0.00000000

C 0.68669900 -0.68951300 -0.00000100

C 2.07715400 -0.12101400 -0.00000400

O -0.57123000 1.37012600 -0.00000800

H -1.75818000 -1.72158900 0.00000300

H -2.39908000 -0.31517700 0.88272700

H -2.39910200 -0.31516300 -0.88268400

H 0.60522200 -1.77367900 0.00000500

H 2.09973400 0.97532300 -0.00007600

H 2.64355300 -0.45227800 0.88150000

H 2.64359100 -0.45239400 -0.88143900

H 0.31734500 1.76542900 -0.00001600

**Eb-H**

C 1.85248800 -0.58722400 -0.00028800

C 0.64157800 -0.01615100 0.00005900

C -0.65561900 -0.81553800 0.00046400

C -1.94730300 0.00552300 -0.00038600

O 0.56099100 1.35484000 0.00015300

H 2.75930800 0.01069000 -0.00047700

H 1.94909300 -1.66792100 -0.00037300

H -0.64976000 -1.47618900 -0.87467800

H -0.65008400 -1.47487200 0.87661000

H -2.81175200 -0.66624900 -0.00014500

H -2.03196600 0.64111400 0.89004600

H -2.03151300 0.63996100 -0.89168000

H -0.36811700 1.63508700 0.00038200

**Ea_H-IPA**

C 1.07797300 1.75934300 -0.37424000

C 1.77889200 0.47679500 -0.02883900

C 3.07963000 0.24615800 -0.26866900

C 3.80734600 -1.02565700 0.05795100

O 1.00010800 -0.49198200 0.55410900

H 1.77393900 2.48362600 -0.80597700

H 0.27074700 1.58184500 -1.09760900

H 0.61734000 2.20780200 0.51576500

H 3.64692500 1.04421500 -0.74142600

H 3.15100300 -1.74970100 0.55023000

H 4.21429100 -1.50426100 -0.84500200

H 4.66329900 -0.84242800 0.72368700

H 0.08619200 -0.14671200 0.71353400

C -2.12303100 -1.44025400 -0.68102400

C -2.34097300 0.00385200 -0.24645100

H -1.05525000 -1.64603400 -0.80822100

H -2.52386500 -2.13372300 0.06976600

H -2.63140100 -1.63845500 -1.63149600

C -3.80865500 0.34954600 -0.03318400

H -1.91168000 0.67911300 -0.99387300

H -3.91761500 1.38974400 0.29244400

H -4.37485900 0.21610200 -0.96208200

H -4.25218200 -0.30211100 0.73087400

O -1.58411400 0.29638700 0.95553400

H -1.91193000 -0.29297000 1.65898100

**Eb_H_IPA**

C -3.42068800 -0.79967500 0.05253300

C -2.15780800 -0.37645100 -0.09922100

C -1.78093000 1.04049400 -0.45025800

C -1.12970900 1.77437100 0.73129600

O -1.11826100 -1.23641400 0.13099400

H -3.64169300 -1.82695600 0.32987100

H -4.25143100 -0.11631200 -0.08519700

H -1.08257500 1.02700800 -1.29862500

H -2.67502800 1.57940100 -0.77890000

H -0.80088500 2.77696800 0.43533600

H -1.83990200 1.87643600 1.55998800

H -0.25779000 1.22675200 1.10176600

H -0.28126500 -0.87564300 -0.25225100

C 2.70057200 1.17532100 -0.05529800

C 2.46429100 -0.30815200 -0.30693000

H 2.72685400 1.73177500 -1.00067100

H 1.90864500 1.59672000 0.57328400

H 3.66145500 1.32726200 0.44971400

C 2.34561500 -1.11926100 0.97326500

H 3.29138600 -0.70597500 -0.91236100

H 2.14570900 -2.17227500 0.74616900

H 3.27683400 -1.06094700 1.54659400

H 1.52890000 -0.73934900 1.59821300

O 1.23686400 -0.52076400 -1.04220200

H 1.21391000 0.09267600 -1.79558600

**H_2_O_IPA**

O -2.72235400 -0.19089700 0.11616300

H -2.81242800 0.63118000 -0.39518700

C 2.15865700 -0.43314700 0.13663600

C 0.69988600 -0.02783400 0.29816900

H 2.31320400 -1.46287600 0.47893900

H 2.45603500 -0.37418400 -0.91793800

H 2.81360200 0.22602400 0.71727500

C 0.44320200 1.40204100 -0.17830000

H 0.43410500 -0.09250800 1.36727400

H -0.61579100 1.66272100 -0.06494600

H 1.03378100 2.12440200 0.39828400

H 0.70889300 1.50334600 -1.23797600

O -0.08401300 -0.96491000 -0.44601700

H -2.66331800 0.12113300 1.03520600

H -1.02761600 -0.73913800 -0.28113200

**OH^–^_IPA**

O 2.57729500 -0.15575800 -0.26414800

H 2.86242000 0.39920600 0.47860400

C -2.04642400 -0.51026300 -0.12872300

C -0.57501700 -0.06892000 -0.25634700

H -2.15051200 -1.56072800 -0.43109400

H -2.37000900 -0.43451500 0.91954000

H -2.72980700 0.09327500 -0.74349300

C -0.43899100 1.41879400 0.13962600

H -0.34154900 -0.10518900 -1.35518500

H 0.60393800 1.73439300 0.01425600

H -1.07525100 2.08643400 -0.45996100

H -0.70418900 1.54892100 1.19895300

O 0.25187500 -0.88309000 0.48815400

H 1.63419800 -0.48868000 0.05899800

**IPA**

C -1.26704200 -0.68202500 -0.08322000

C 0.00000400 0.04738600 0.35221600

H -2.15880800 -0.13597600 0.24494600

H -1.29958500 -0.76756900 -1.17646600

H -1.30440100 -1.69229000 0.34125100

C 1.26687500 -0.68232400 -0.08325200

H 0.00003000 0.14161500 1.44572400

H 2.15877700 -0.13645900 0.24485400

H 1.30403900 -1.69258200 0.34125600

H 1.29934800 -0.76791300 -1.17649600

O 0.00010100 1.36766300 -0.23374700

H 0.00077300 2.01163800 0.49044500

**COD**

C -1.56709100 -0.70899100 -0.50603500

H -2.33040200 -1.06607900 -1.19997500

C -1.57855100 0.60204500 -0.23218500

H -2.33270400 1.19858100 -0.74740300

C -0.66064800 1.39241200 0.67175200

H -0.45196000 0.85643900 1.59998000

H -1.17739000 2.31342000 0.96764200

C 0.66064800 1.80542000 -0.00713500

H 0.42325500 2.45266800 -0.86409200

H 1.22789900 2.44751000 0.68546200

C 1.56709100 0.70899100 -0.50603500

H 2.33040200 1.06607900 -1.19997500

C 1.57855100 -0.60204500 -0.23218500

H 2.33270400 -1.19858100 -0.74740300

C 0.66064800 -1.39241200 0.67175200

H 0.45196000 -0.85643900 1.59998000

H 1.17739000 -2.31342000 0.96764200

C -0.66064800 -1.80542000 -0.00713500

H -0.42325500 -2.45266800 -0.86409200

H -1.22789900 -2.44751000 0.68546200

**A0**

C -0.20212700 -2.86173900 3.84488400

C 0.24547900 -1.55778700 3.64761600

C -0.19841500 -0.77380000 2.56968800

C -1.12461300 -1.33144600 1.65224800

C -1.60174600 -2.62870200 1.89503300

C -1.14964000 -3.39187700 2.97129900

C 0.26739200 0.65206600 2.58020900

C -0.13763300 1.40313900 3.69623000

C 0.29186000 2.71166900 3.90096000

C 1.18657200 3.27908200 2.99594300

C 1.60582200 2.54564600 1.88648600

C 1.14331200 1.24429000 1.63738600

P -1.59540200 -0.38692900 0.12738800

P 1.58729200 0.32549800 0.08823500

C -3.09964800 -1.21908700 -0.52805900

C -2.31384700 1.12670200 0.89428600

C 2.38969100 -1.16161200 0.82313900

C 3.04102300 1.22761600 -0.58802400

C 2.01331700 -2.43579700 0.40574400

C 2.65345400 -3.59006900 0.87981400

C 3.73944900 -3.39688900 1.76464100

C 4.01607100 -2.14005600 2.36202300

C 3.34240800 -1.03397800 1.83686500

C 2.91816300 2.55497200 -1.00937200

C 3.95189000 3.23912200 -1.65811600

C 5.11966100 2.50064700 -1.97127700

C 5.31262200 1.17807400 -1.51104600

C 4.24535700 0.57054800 -0.83599500

C -3.03262900 -2.54319000 -0.98428800

C -4.08245700 -3.14565100 -1.67845900

C -5.21595200 -2.34246200 -1.97551500

C -5.37546000 -1.04956700 -1.43474800

C -4.27775200 -0.50882000 -0.74437500

C -1.91644400 2.38459300 0.44036200

C -2.48602500 3.56084600 0.93728600

C -3.50325000 3.42433100 1.91528400

C -3.80063700 2.18384800 2.52675200

C -3.21908700 1.04572900 1.95007800

H 0.17189700 -3.44543700 4.68113800

H -2.35518300 -3.05355300 1.24528800

H -1.54215100 -4.39378900 3.12153400

H 1.56788900 4.28479500 3.14944700

H 1.19236800 -2.51708000 -0.29173500

H 3.55082400 -0.04459300 2.22168400

H 1.97762400 3.06719400 -0.85345700

H 4.35287100 -0.45426600 -0.51051800

H -2.11968200 -3.09834800 -0.82784400

H -4.34756200 0.50415800 -0.37466400

H -1.14375000 2.42813800 -0.31331600

H -3.47257000 0.06639200 2.34053400

H -0.80988500 0.94063400 4.41146700

H 0.95799300 -1.12368900 4.34277900

C 3.69176700 4.73443500 -1.96900100

C 2.56742800 4.84568600 -3.02155900

C 3.22602200 5.42410900 -0.66204700

C 4.90319900 5.54520800 -2.46826900

H 2.86271400 4.37137400 -3.96444400

H 1.64158800 4.37146300 -2.67957900

H 2.34736300 5.90062200 -3.22804900

H 4.00172800 5.35684600 0.11034400

H 3.03024400 6.48590000 -0.85321500

H 2.30829800 4.99052200 -0.25698700

H 4.61477600 6.60336500 -2.49886300

H 5.76276800 5.45201100 -1.79820600

H 5.21937000 5.26548800 -3.47421000

C 6.58853800 0.31603300 -1.67802200

C 6.96071800 -0.28779700 -0.29904200

C 6.28030800 -0.83571400 -2.65975100

C 7.84680400 1.05545300 -2.17230100

H 7.16249300 0.50463600 0.43175800

H 6.17625700 -0.92969800 0.11008900

H 7.86634200 -0.89800000 -0.39626100

H 6.01175300 -0.44730500 -3.64918600

H 7.15852700 -1.48321800 -2.77604200

H 5.44924600 -1.45509200 -2.30399800

H 8.69809700 0.36710400 -2.09957600

H 7.77388700 1.37358700 -3.21312200

H 8.07252600 1.93313100 -1.55936200

C -1.95905700 4.93684300 0.47463000

C -1.49433000 5.74538300 1.70615100

C -0.73898700 4.78126600 -0.45527500

C -3.02236900 5.74170400 -0.30222200

H -2.31611100 5.92205100 2.40350800

H -0.69817500 5.21296800 2.23773900

H -1.09852400 6.71807500 1.38788700

H -1.00080200 4.29279200 -1.40023500

H -0.34388400 5.77434300 -0.69556600

H 0.06368800 4.20734300 0.01837500

H -2.55696700 6.62843000 -0.75046200

H -3.45404000 5.14460300 -1.11445500

H -3.83065200 6.08577100 0.34419900

C -4.66205600 1.96453900 3.79608300

C -5.95756700 1.21216100 3.42697900

C -3.84662800 1.09093300 4.78330200

C -5.02701600 3.23973800 4.58205200

H -6.57948700 1.79737400 2.74097700

H -5.73911500 0.25164100 2.94836100

H -6.54825600 1.01198300 4.32969500

H -2.92822700 1.60619300 5.08586100

H -4.44225800 0.90356500 5.68447500

H -3.56811100 0.11950700 4.36635600

H -5.46875300 2.93641400 5.53918500

H -4.14301200 3.84672300 4.79989600

H -5.75918200 3.86843300 4.07457600

C -6.64860900 -0.16905300 -1.49868700

C -6.91928900 0.40913900 -0.08565400

C -6.40918300 1.01100500 -2.46474700

C -7.94117300 -0.90864100 -1.89862600

H -7.04835000 -0.39470600 0.64821800

H -6.11919400 1.06366800 0.26722200

H -7.84066900 1.00271700 -0.10385300

H -6.21734000 0.66480600 -3.48608000

H -7.28843700 1.66696500 -2.48953400

H -5.54896300 1.61172400 -2.14737700

H -8.78952700 -0.23357100 -1.73298200

H -7.96666200 -1.20708600 -2.94644500

H -8.09984200 -1.80076500 -1.28424300

C -4.00432700 -4.64479100 -2.04330700

C -4.00955700 -4.89038400 -3.56730600

C -2.71755400 -5.29027200 -1.49125900

C -5.20120400 -5.37685300 -1.39662400

H -4.98018600 -4.67825400 -4.01580100

H -3.24974400 -4.27998300 -4.06929200

H -3.77696400 -5.94339800 -3.76852400

H -2.63072700 -5.17794800 -0.40513900

H -2.73460100 -6.36320300 -1.71230200

H -1.81647100 -4.87356400 -1.95469000

H -5.15905200 -6.44764800 -1.63224900

H -5.17689000 -5.27066800 -0.30524300

H -6.15365300 -4.98379000 -1.75961500

H -0.05587200 3.27012300 4.76544400

H 2.32290100 2.99881300 1.21615500

C 2.08446400 -4.98839300 0.53996100

C 1.74968800 -5.71670000 1.86129200

C 0.77330500 -4.87091500 -0.26331300

C 3.04313400 -5.86594600 -0.29127300

H 2.64299800 -5.86320300 2.47301900

H 1.02078100 -5.14392100 2.44409700

H 1.31597400 -6.70154100 1.64663900

H 0.93747800 -4.43968100 -1.25707600

H 0.35195800 -5.87242400 -0.40319300

H 0.02702100 -4.26423900 0.25781200

H 2.50318300 -6.74855800 -0.65569100

H 3.42490900 -5.32546200 -1.16549000

H 3.88888600 -6.22401700 0.29727500

C 4.87765200 -1.98837600 3.63756000

C 4.85177400 -0.53697000 4.15625100

C 4.25612700 -2.88410100 4.73349400

C 6.36068100 -2.37016800 3.45234400

H 5.32349200 0.15952900 3.45313900

H 3.83390100 -0.18548900 4.35706600

H 5.41107600 -0.48609300 5.09683600

H 4.27018300 -3.93726700 4.44130500

H 4.81862100 -2.78049900 5.66981500

H 3.21644000 -2.59517700 4.92796600

H 6.92708100 -2.06557500 4.34086900

H 6.49732700 -3.44569000 3.33417800

H 6.80096900 -1.85991700 2.58754800

O -4.21007000 4.56091300 2.27171800

O -6.17476500 -2.87490900 -2.82254100

O 6.08661200 3.08385800 -2.76852700

O 4.54241100 -4.48194900 2.08878100

C 5.66519900 -4.61402600 1.19951900

H 6.33150100 -3.75044400 1.26749000

H 6.20004600 -5.51496600 1.51035100

H 5.33949500 -4.71948400 0.16163000

C 5.86270100 2.85478800 -4.16118000

H 6.66573800 3.36675600 -4.69753100

H 5.89093700 1.78372300 -4.39635400

H 4.89396700 3.25942200 -4.47912400

C -5.50225100 4.62649600 1.65575700

H -6.15411300 3.81603000 1.99655400

H -5.93555900 5.58708900 1.94530700

H -5.42089900 4.57428900 0.56448800

C -6.07419600 -2.38854800 -4.16704200

H -5.06231300 -2.52671000 -4.56219400

H -6.78235700 -2.97144200 -4.76143100

H -6.33412000 -1.32796100 -4.23403400

Ni -0.01340500 -0.05808400 -1.32290700

C -1.16146900 0.95910600 -2.79202500

C 0.09404900 1.54983700 -2.69575600

C 1.13240100 -1.11634800 -2.76737100

C -0.12208200 -1.70542200 -2.64992100

H -1.98513800 1.42927100 -2.25874000

C -1.55427700 0.01625700 -3.92086200

H 0.18405500 2.46009800 -2.10727500

C 1.24196000 1.29266800 -3.65303000

H 1.96130900 -1.56743800 -2.22680300

C 1.51216500 -0.20672300 -3.92677800

C -1.26219000 -1.47635400 -3.62374500

H -0.21459800 -2.59784700 -2.03398300

H -1.03992700 0.32088900 -4.84029400

H -2.62649900 0.12823600 -4.12343200

H 2.14094200 1.73245400 -3.21700000

H 1.08054100 1.81877500 -4.60925100

H 0.97431900 -0.52524700 -4.82766000

H 2.57851700 -0.33510000 -4.15002300

H -2.15925300 -1.92761400 -3.19658300

H -1.07731100 -2.00885200 -4.57222900

**2a_IPA**

C -2.10322000 -0.44889200 -1.06860400

C -3.18220300 -0.11639500 -0.23189700

C -2.99920700 0.82012100 0.79150500

C -1.74809200 1.41558400 0.95942800

C -0.66337600 1.11138300 0.12496100

C -0.86821000 0.15370100 -0.89065000

C 0.63389300 1.75924200 0.34694700

C 1.66325000 1.78630100 -0.51311000

C 3.00081300 2.36477200 -0.18774900

O 3.94810500 1.27328900 -0.08594300

O -4.35426100 -0.76340500 -0.48970400

C -5.47758200 -0.46777000 0.34012500

H -2.25534100 -1.19164800 -1.84623300

H -3.81145700 1.08788500 1.45677100

H -1.61464000 2.14001300 1.75933300

H -0.04089300 -0.14167500 -1.52815800

H 0.75023700 2.25584500 1.31111000

H 1.57993700 1.32178600 -1.49299200

H 3.33103500 3.05426200 -0.97536700

H 2.97007400 2.91615500 0.76136300

H 4.81880200 1.59384600 -0.37328000

H -6.29510900 -1.08582700 -0.03576300

H -5.27737000 -0.72496600 1.38714100

H -5.75739700 0.59007700 0.26907000

C 1.42560000 -2.88382100 -0.01091300

C 2.30338600 -1.68272100 0.31082200

H 0.58954800 -2.59197600 -0.65621200

H 2.00775600 -3.65702600 -0.52801300

H 1.01378200 -3.31859100 0.90670900

C 3.46938200 -2.04302200 1.23010000

H 1.68660300 -0.91928100 0.80909700

H 4.08452300 -1.15869500 1.43416300

H 3.10934200 -2.43421500 2.18954500

H 4.10562800 -2.80443800 0.76165200

O 2.78275000 -1.16113800 -0.93565300

H 3.26559200 -0.33239100 -0.72533700

**A1**

C 0.30282800 -0.08966100 -5.06793700

C 0.16969800 1.03663600 -4.25653600

C 0.67199700 1.06316300 -2.94787700

C 1.35509900 -0.07667600 -2.45532400

C 1.50750400 -1.19171500 -3.29116000

C 0.98080400 -1.20756200 -4.58351000

C 0.50701400 2.34633800 -2.18694800

C 1.22683300 3.45425100 -2.65560000

C 1.11796400 4.70614500 -2.05141800

C 0.24721700 4.87470000 -0.97575500

C -0.49486700 3.78872200 -0.50952600

C -0.37324700 2.51885700 -1.09060600

P 1.88427900 -0.13383400 -0.68877500

P -1.22460900 1.05829100 -0.35125100

C 2.96430800 -1.60461500 -0.51896900

C 3.01676100 1.29043600 -0.53824500

C -2.17989000 0.38537600 -1.75342700

C -2.52909100 1.74618800 0.74013400

C -2.22262100 -0.99318800 -1.95491800

C -3.02801200 -1.56056300 -2.95312700

C -3.85971400 -0.67641700 -3.67874800

C -3.69451300 0.73072100 -3.63492200

C -2.85783500 1.23135200 -2.63461400

C -2.14948700 2.25200100 1.98654700

C -3.08237400 2.60175100 2.96767400

C -4.44759100 2.35123900 2.67531100

C -4.87888900 1.94816400 1.39121200

C -3.88667500 1.63652200 0.45163200

C 2.36232600 -2.87185900 -0.52065300

C 3.07037900 -4.02390300 -0.17855700

C 4.41892800 -3.85889100 0.24425700

C 5.09443200 -2.62616100 0.12991900

C 4.32013600 -1.50818300 -0.22763100

C 2.85990800 2.13598500 0.56271600

C 3.71598600 3.21641300 0.78462000

C 4.77681900 3.40006600 -0.13929100

C 4.85807400 2.67639500 -1.35218600

C 3.98095300 1.59118900 -1.49703300

H -0.11279600 -0.08771500 -6.07147000

H 2.04730500 -2.06045600 -2.93123500

H 1.10854000 -2.08808300 -5.20686900

H 0.13577600 5.84628500 -0.50276700

H -1.61039100 -1.62296100 -1.32196700

H -2.72505300 2.29949200 -2.52559300

H -1.09115700 2.34133200 2.20630800

H -4.18615000 1.25931700 -0.51558300

H 1.31133500 -2.94149200 -0.77191400

H 4.78964600 -0.53483800 -0.25589200

H 2.04591900 1.93118100 1.24559000

H 4.04494600 0.96930500 -2.38273300

H 1.89302700 3.31978500 -3.50172800

H -0.34615800 1.91501500 -4.63297400

C -2.51509200 3.20846500 4.27474500

C -1.67466100 2.13805200 5.00303900

C -1.59489000 4.39429700 3.89323800

C -3.54970600 3.77072400 5.26826200

H -2.27906300 1.26139200 5.25676200

H -0.83411600 1.79790600 4.38960700

H -1.26371000 2.54788900 5.93437000

H -2.16098000 5.17561900 3.37159600

H -1.16475200 4.83596200 4.80005600

H -0.76432200 4.09285900 3.24991300

H -3.00602200 4.29873400 6.06160800

H -4.22830500 4.48572200 4.79420100

H -4.14808200 2.99347900 5.74620200

C -6.34361800 1.78997900 0.91438600

C -6.49950600 2.52701500 -0.44007500

C -6.64165700 0.29021200 0.69833700

C -7.42306700 2.37243200 1.84697400

H -6.27530700 3.59505100 -0.33218400

H -5.84769500 2.12296100 -1.21958800

H -7.53252900 2.43160000 -0.79463700

H -6.55741600 -0.26598800 1.63869900

H -7.66223200 0.15766800 0.31742900

H -5.95108000 -0.15860900 -0.02375200

H -8.38591000 2.33338300 1.32244600

H -7.53311800 1.80895500 2.77459300

H -7.22249600 3.41754100 2.10160100

C 3.44556000 4.19463600 1.94794000

C 3.31522600 5.62807300 1.38638500

C 2.11560600 3.86309900 2.65497600

C 4.55614800 4.15406900 3.01820900

H 4.23353500 5.95093100 0.89151900

H 2.49418600 5.68446400 0.66297900

H 3.09827400 6.33026800 2.20098100

H 2.14214900 2.88951500 3.15763600

H 1.92078100 4.62228900 3.42020900

H 1.27209800 3.86858700 1.95679900

H 4.24703100 4.74258100 3.89096100

H 4.74137500 3.12746800 3.35625000

H 5.49387500 4.57460900 2.65292900

C 5.78503100 2.99300600 -2.55246900

C 6.83572600 1.87390600 -2.70852700

C 4.91426700 3.03709500 -3.83412900

C 6.50467600 4.35552000 -2.49639300

H 7.47819700 1.80258500 -1.82411300

H 6.36153100 0.89851500 -2.86118900

H 7.47695300 2.07564400 -3.57566900

H 4.16183500 3.83056300 -3.76316700

H 5.55056400 3.25040700 -4.70097700

H 4.39636600 2.09499100 -4.03284000

H 6.97098600 4.53555200 -3.47265400

H 5.80522700 5.17470600 -2.30395200

H 7.29705400 4.39712400 -1.74852600

C 6.60767200 -2.37849100 0.34645200

C 7.13228500 -1.47780300 -0.80122900

C 6.82074200 -1.62618600 1.67766900

C 7.48658900 -3.64516900 0.32110700

H 6.93634900 -1.93027300 -1.78055900

H 6.68861800 -0.47992000 -0.79286300

H 8.21583300 -1.34923500 -0.69780600

H 6.47574800 -2.21389400 2.53488200

H 7.88594000 -1.40763400 1.82412200

H 6.27704700 -0.67426300 1.68260800

H 8.53829800 -3.33527900 0.30182900

H 7.35406400 -4.28512500 1.19316200

H 7.29916300 -4.24493800 -0.57566800

C 2.40731500 -5.41224200 -0.31942600

C 2.20434700 -6.10916300 1.04255500

C 1.02083000 -5.30287200 -0.98361500

C 3.27799500 -6.30084800 -1.23574200

H 3.14722900 -6.42721300 1.48846400

H 1.68738600 -5.45001700 1.75040600

H 1.58489900 -7.00444100 0.90752500

H 1.07495000 -4.82067500 -1.96505900

H 0.61561100 -6.31033800 -1.12975300

H 0.30652200 -4.74748100 -0.36821700

H 2.80528100 -7.28285200 -1.36160000

H 3.38095900 -5.84761700 -2.22920900

H 4.27641700 -6.45190700 -0.82089000

H 1.70425300 5.54103400 -2.42461600

H -1.18170800 3.93508400 0.31656500

C -2.88311700 -3.05584300 -3.31861600

C -2.56762800 -3.16387000 -4.82798000

C -1.69651600 -3.68609600 -2.56470900

C -4.13477600 -3.89818300 -2.99630300

H -3.38218500 -2.76817600 -5.43852300

H -1.65246900 -2.61195500 -5.07117100

H -2.41282100 -4.21504100 -5.10179800

H -1.86345600 -3.71691300 -1.48484100

H -1.56273400 -4.71741200 -2.90956300

H -0.76355400 -3.14547000 -2.75493100

H -3.89218700 -4.96298600 -3.09890200

H -4.47950100 -3.73045300 -1.97038700

H -4.95754800 -3.68825700 -3.68189800

C -4.26314500 1.67521500 -4.71857000

C -3.77179300 3.12078200 -4.50810800

C -3.72871900 1.19847500 -6.08841400

C -5.80414500 1.72594600 -4.76475300

H -4.15027700 3.55330000 -3.57456200

H -2.67842700 3.18999700 -4.50025200

H -4.13666500 3.74347300 -5.33221500

H -4.06776400 0.18483700 -6.31669300

H -4.08252300 1.86566200 -6.88427900

H -2.63237800 1.20570700 -6.10129400

H -6.12003100 2.53749600 -5.43153900

H -6.23464000 0.80116700 -5.15063200

H -6.22807000 1.92661800 -3.77385100

O 5.74729100 4.33403700 0.17797700

O 5.05871700 -4.96622900 0.77580800

O -5.37976500 2.47930400 3.68645100

O -4.85573500 -1.20575300 -4.48654100

C -6.09532800 -1.36575200 -3.77316400

H -6.47485500 -0.40426500 -3.41709600

H -6.80416100 -1.80193900 -4.48128100

H -5.97543900 -2.03070100 -2.91396600

C -5.56227500 1.26367200 4.41712600

H -6.29764000 1.47352900 5.19797900

H -5.93715800 0.46380000 3.76705200

H -4.62311100 0.93363200 4.87766500

C 6.94740900 3.73809400 0.68794800

H 7.44423600 3.12146000 -0.06758700

H 7.60475100 4.56284900 0.97376100

H 6.73543200 3.11601000 1.56438700

C 5.10646900 -4.96570700 2.20812300

H 4.11284700 -4.79532900 2.63614200

H 5.46870200 -5.95281900 2.50616600

H 5.78958400 -4.20126700 2.58997300

Ni 0.21902500 -0.17790400 0.67470900

C -1.65386900 -1.86419300 1.95548100

C -1.34533200 -3.15929700 1.50426000

C -2.33206900 -4.04891500 1.07137900

C -3.67148000 -3.64887600 1.06441300

C -4.00829700 -2.36980200 1.53274100

C -3.01962100 -1.50171700 1.97596600

C -0.62437400 -0.89062500 2.36159500

C 0.77808600 -1.14766100 2.34537800

C 1.70991100 -0.36941600 3.22695200

H 1.14063000 -2.14913000 2.10365800

H -0.99042200 -0.07546600 2.98200400

H -0.31249200 -3.49117800 1.48681100

H -3.29925200 -0.51547900 2.33458700

H -2.04102700 -5.03734800 0.73574400

H -5.05289200 -2.07457500 1.54198000

H 2.71950900 -0.32586400 2.79739800

H 1.35774500 0.65547600 3.37295400

O 1.82075800 -0.91034600 4.57918100

H 2.07446000 -1.84561600 4.48814700

O -4.71298500 -4.42524800 0.62195200

C -4.41321600 -5.75916600 0.22077100

H -5.36235900 -6.19783600 -0.09406000

H -3.70915600 -5.78089100 -0.61871800

H -4.00139500 -6.34029600 1.05569600

C -0.37297600 -3.41103800 6.43591900

C -1.09938600 -2.46766700 5.47719700

H 0.71379400 -3.32539500 6.31136800

H -0.61696100 -3.16360200 7.47675500

H -0.65293200 -4.45606600 6.25300200

C -2.61333800 -2.54207900 5.62193900

H -0.83624100 -2.74282700 4.44625900

H -3.10009000 -1.87260300 4.90443000

H -2.97297300 -3.56087600 5.43785200

H -2.91749600 -2.24596200 6.63414900

O -0.71195200 -1.11077400 5.71748500

H 0.19593800 -1.00242600 5.35114100

**A2**

C 0.31214300 1.66529000 4.37434100

C 0.15674100 0.34788000 3.95188400

C 0.64178200 -0.08069000 2.71061900

C 1.32390400 0.84346800 1.88932700

C 1.49970100 2.15865400 2.33580200

C 0.99025800 2.57272900 3.56443400

C 0.46295700 -1.51603300 2.35061300

C 1.10812200 -2.46827800 3.14871900

C 0.99505100 -3.83069700 2.88685300

C 0.20191500 -4.26336600 1.82733000

C -0.47033600 -3.33132200 1.03993400

C -0.34590300 -1.95802500 1.28192900

P 1.92348000 0.32683200 0.23518000

P -1.18414500 -0.74451800 0.18643100

C 3.02220900 1.65856000 -0.34367500

C 2.99734900 -1.09637300 0.54232000

C -2.12811300 0.31034800 1.31327200

C -2.43426600 -1.73858500 -0.69006600

C -2.16071300 1.68687500 1.10819200

C -2.97328800 2.51615800 1.89012900

C -3.82435500 1.88114200 2.82550600

C -3.65934900 0.52236900 3.19341500

C -2.81223600 -0.24639900 2.39488200

C -2.03551200 -2.48230800 -1.80048500

C -2.94577900 -3.20827200 -2.57293000

C -4.31355400 -3.12853500 -2.20530900

C -4.73286300 -2.48815200 -1.01589400

C -3.76811500 -1.77061700 -0.30131600

C 2.45211000 2.87351900 -0.74266400

C 3.20467500 3.85757600 -1.37961600

C 4.56663700 3.56128300 -1.66440900

C 5.20225300 2.41152700 -1.15038000

C 4.38332000 1.45826700 -0.52445300

C 2.89361900 -2.21285300 -0.28633200

C 3.76274500 -3.29613600 -0.15218700

C 4.77444100 -3.20040500 0.83801100

C 4.78608600 -2.16847000 1.80652100

C 3.90466000 -1.10098200 1.59784000

H -0.09056200 1.97907800 5.34044200

H 2.05154100 2.87302000 1.72329900

H 1.13503300 3.60588600 3.88898900

H 0.09238100 -5.32946800 1.61483900

H -1.53360100 2.11234900 0.32460900

H -2.67557100 -1.30512700 2.61330100

H -0.98129200 -2.47702100 -2.08503600

H -4.07848300 -1.20468600 0.57542800

H 1.38968500 3.04156700 -0.56453700

H 4.82781200 0.52352600 -0.18433000

H 2.11681200 -2.22236100 -1.05033100

H 3.92080300 -0.25149100 2.28382900

H 1.72483100 -2.12254200 3.98085500

H -0.36457900 -0.37132300 4.58810200

C -2.37048000 -3.99417700 -3.76899500

C -1.92212000 -2.99360500 -4.84427200

C -1.14085200 -4.78412300 -3.28734100

C -3.31678400 -5.01611900 -4.40932400

H -2.76600900 -2.38470900 -5.20400100

H -1.15049100 -2.30769900 -4.46068400

H -1.49572000 -3.52715600 -5.70926800

H -1.40914500 -5.48832100 -2.48349400

H -0.72299500 -5.36688300 -4.12296800

H -0.33971200 -4.13254000 -2.91432200

H -2.73521500 -5.62062200 -5.12370700

H -3.74993700 -5.70054300 -3.66573100

H -4.13807600 -4.55192000 -4.96810700

C -6.15663800 -2.49552600 -0.42088600

C -6.04928300 -2.87271400 1.06893100

C -6.75827100 -1.08733100 -0.53170900

C -7.13372000 -3.49643100 -1.04712500

H -5.60134300 -3.87156800 1.19351000

H -5.44778200 -2.15793900 1.64801400

H -7.05409200 -2.89370400 1.51940700

H -6.88014000 -0.78838900 -1.58420800

H -7.75286100 -1.06169300 -0.05733600

H -6.12871100 -0.33293800 -0.03436200

H -8.04671400 -3.51585000 -0.43055700

H -7.43544700 -3.22364600 -2.06562500

H -6.72317800 -4.51601400 -1.07056200

C 3.56617600 -4.56045500 -1.00577500

C 3.40410300 -5.77354200 -0.07663700

C 2.28895000 -4.45704400 -1.84560300

C 4.73492500 -4.79625300 -1.97202900

H 4.29784800 -5.93267300 0.54076300

H 2.53966800 -5.63943500 0.59245900

H 3.23187900 -6.68378200 -0.67354500

H 2.34406400 -3.65213300 -2.59511500

H 2.13620200 -5.40151200 -2.38964500

H 1.40021100 -4.28794100 -1.21941700

H 4.48308900 -5.61935800 -2.66017300

H 4.93632500 -3.90130600 -2.58209400

H 5.65717000 -5.07737500 -1.44953500

C 5.64468900 -2.13031600 3.08913200

C 6.70304200 -1.02490100 2.97555400

C 4.71858400 -1.80186100 4.27534600

C 6.32935600 -3.45314500 3.45431700

H 7.39691600 -1.20999800 2.14151100

H 6.23984500 -0.03869000 2.81935900

H 7.29752600 -0.97330500 3.90210700

H 3.95357900 -2.58320200 4.40350700

H 5.31257100 -1.75708800 5.20153300

H 4.20643500 -0.83512700 4.17021800

H 6.74386400 -3.35628600 4.47033600

H 5.61874200 -4.29242300 3.46214000

H 7.16255400 -3.71276800 2.79097900

C 6.71265700 2.10146800 -1.19728100

C 7.14536600 1.56936800 0.18164700

C 6.97722700 1.00268000 -2.23700100

C 7.60868900 3.31369700 -1.47957400

H 6.92165200 2.29563900 0.97927800

H 6.66337500 0.61815100 0.44235100

H 8.23181500 1.39037900 0.18151200

H 6.68782700 1.31746800 -3.25094500

H 8.04885900 0.74557800 -2.25598500

H 6.41512900 0.08599400 -1.99728500

H 8.65546200 3.01993600 -1.30304700

H 7.54546300 3.67708700 -2.51143400

H 7.37956400 4.15239600 -0.80526300

C 2.57807200 5.22661200 -1.69759000

C 2.48826600 5.49271800 -3.20670500

C 1.15684900 5.32425200 -1.13474100

C 3.41323600 6.32543000 -1.02216900

H 3.47203500 5.64843900 -3.66450500

H 1.98515500 4.66426400 -3.73062700

H 1.89666700 6.40544500 -3.38316300

H 1.13034600 5.15403500 -0.04837100

H 0.76390000 6.33513100 -1.32152400

H 0.46633200 4.61227000 -1.61282600

H 2.96742800 7.31311500 -1.22266000

H 3.43697900 6.18257600 0.07010600

H 4.44651000 6.33351500 -1.39329000

H 1.52435700 -4.55188600 3.51450000

H -1.10931200 -3.68520600 0.22913300

C -2.83146900 4.04751000 1.80860300

C -2.53666500 4.58737800 3.21687500

C -1.64266200 4.42774600 0.92068300

C -4.07463700 4.74492400 1.24081300

H -3.36131500 4.37869300 3.91117300

H -1.61713700 4.13818200 3.62395700

H -2.39090700 5.67903000 3.17628800

H -1.80204300 4.14681800 -0.12978400

H -1.50323300 5.51889000 0.95326500

H -0.70889500 3.96114800 1.26870800

H -3.82860900 5.79033300 0.99413100

H -4.42430500 4.25564700 0.31950200

H -4.90274300 4.76901900 1.96015400

C -4.25591600 -0.07111600 4.48325900

C -3.74922000 -1.49906000 4.71132100

C -3.77718100 0.78704100 5.66495300

C -5.78909100 -0.13033600 4.48467700

H -4.09744800 -2.19472400 3.93181500

H -2.65031200 -1.55115100 4.75202300

H -4.13338000 -1.86597600 5.67518600

H -4.13249500 1.82315800 5.57853400

H -4.15838800 0.37253500 6.61226500

H -2.67703900 0.80170400 5.71993400

H -6.12925800 -0.71487100 5.35445800

H -6.24417800 0.86411100 4.56504700

H -6.17637200 -0.62553100 3.58021500

O 5.74486500 -4.15919000 0.84350000

O 5.25107100 4.44614300 -2.44694800

O -5.25116500 -3.68206500 -3.02289400

O -4.81382300 2.61256400 3.42230500

C -6.03830300 2.59077800 2.70091100

H -6.44880700 1.57242500 2.63092000

H -6.74569300 3.22837600 3.24859500

H -5.91478000 2.98010700 1.67968600

C -5.77412000 -2.79102500 -3.98505300

H -6.53188300 -3.33910800 -4.56200200

H -6.24864400 -1.91463900 -3.51086200

H -4.99015500 -2.43212000 -4.67383800

C 6.97665100 -3.73942900 0.28589500

H 7.44250200 -2.93278500 0.87360200

H 7.64492500 -4.61137700 0.28434800

H 6.84695300 -3.38191900 -0.74882500

C 5.41313900 4.04769600 -3.79544300

H 4.45458500 3.73753300 -4.24175000

H 5.80155200 4.91635100 -4.34486700

H 6.12924100 3.21742500 -3.89865600

Ni 0.27423500 0.15948700 -1.18115700

C -2.21376300 1.34577000 -2.65972300

C -2.20729500 2.74103900 -2.51677800

C -3.38423600 3.47602200 -2.39928900

C -4.61890900 2.81612400 -2.39678000

C -4.64694700 1.42291300 -2.54873500

C -3.46740500 0.70701000 -2.68578700

C -0.98219800 0.54881200 -2.77038400

C 0.29780100 1.11186200 -2.95750000

C 1.43532300 0.30977800 -2.80538200

H 0.42541700 2.19975100 -2.92416200

H -1.13131400 -0.48603100 -3.09815200

H -1.26150100 3.28719800 -2.49918900

H -3.51492400 -0.37571900 -2.82180600

H -3.32714400 4.56022700 -2.29668100

H -5.61525900 0.91875100 -2.56403500

H 2.42757800 0.76692500 -2.81694200

H 1.39809300 -0.75495000 -3.07587300

O -5.81075600 3.43252300 -2.25866400

C -5.85894000 4.84097900 -2.30577600

H -6.91926700 5.11801700 -2.24057400

H -5.32001800 5.30164800 -1.46187100

H -5.44456000 5.22605300 -3.25304200

**OH^–^(*^i^*PrOH)**

O 2.57729500 -0.15575800 -0.26414800

H 2.86242000 0.39920600 0.47860400

C -2.04642400 -0.51026300 -0.12872300

C -0.57501700 -0.06892000 -0.25634700

H -2.15051200 -1.56072800 -0.43109400

H -2.37000900 -0.43451500 0.91954000

H -2.72980700 0.09327500 -0.74349300

C -0.43899100 1.41879400 0.13962600

H -0.34154900 -0.10518900 -1.35518500

H 0.60393800 1.73439300 0.01425600

H -1.07525100 2.08643400 -0.45996100

H -0.70418900 1.54892100 1.19895300

O 0.25187500 -0.88309000 0.48815400

H 1.63419800 -0.48868000 0.05899800

**A3**

C 0.33602600 -2.67310900 4.36148800

C 0.52487200 -1.30831300 4.14933300

C -0.08572800 -0.63452900 3.08060400

C -0.93672000 -1.36836100 2.21512800

C -1.14086800 -2.73469500 2.45659000

C -0.50916200 -3.38874400 3.51415100

C 0.14051000 0.84934700 3.01013900

C -0.38488900 1.59908600 4.07356400

C -0.22595800 2.98142700 4.15009200

C 0.50650400 3.64075200 3.16447000

C 1.06400500 2.91003900 2.11522900

C 0.88755600 1.52263700 2.01098100

P -1.61111100 -0.58712800 0.69193400

P 1.48518900 0.61778500 0.51705600

C -2.93841700 -1.68807000 0.05920000

C -2.52671400 0.87350800 1.30235800

C 2.45998000 -0.75058400 1.23902400

C 2.77792300 1.69613300 -0.22035800

C 2.30550900 -2.03761100 0.72753400

C 3.09503300 -3.10627100 1.17354500

C 4.10787200 -2.79938100 2.11118200

C 4.15995300 -1.55732000 2.79358400

C 3.33383600 -0.53991100 2.30846300

C 2.37468100 2.82512900 -0.93688900

C 3.28164400 3.63660500 -1.62820200

C 4.63873800 3.22718100 -1.63911900

C 5.09925800 2.13668400 -0.86537400

C 4.13417600 1.38199000 -0.18598200

C -2.57933900 -2.85475700 -0.63199200

C -3.51217000 -3.60231400 -1.35353000

C -4.84139600 -3.09884400 -1.42347100

C -5.26765300 -2.00744900 -0.63924900

C -4.27619000 -1.30840100 0.07007100

C -2.38258600 2.05923400 0.57858400

C -3.12216400 3.20194000 0.88159400

C -4.05536600 3.10233100 1.94489100

C -4.09864400 1.98372100 2.81041800

C -3.34496400 0.86158300 2.42919100

H 0.83850500 -3.16801300 5.18750900

H -1.80663200 -3.29640800 1.81037800

H -0.68409200 -4.44872300 3.67517700

H 0.65751700 4.71556300 3.21308100

H 1.54706400 -2.19662800 -0.02675500

H 3.36091400 0.44117700 2.76361600

H 1.31781600 3.06838000 -0.96746400

H 4.45175100 0.51016000 0.36635400

H -1.54080400 -3.16051800 -0.61898300

H -4.56131400 -0.42499600 0.62358800

H -1.67243300 2.06646500 -0.23789800

H -3.39736400 -0.04326300 3.02464300

H -0.94476200 1.07982200 4.84418700

H 1.16994900 -0.74460800 4.81674100

C 2.68789100 4.90839000 -2.28442100

C 1.63538800 4.49315600 -3.33653600

C 1.99554500 5.72970700 -1.16838800

C 3.68279600 5.86562300 -2.96687000

H 2.09641900 3.90774500 -4.14077600

H 0.83012500 3.89310900 -2.90038100

H 1.18055500 5.38475500 -3.78575200

H 2.72771800 6.05812500 -0.42074400

H 1.52625900 6.62323100 -1.59773200

H 1.21794100 5.16308400 -0.65080300

H 3.13079200 6.76253600 -3.27499600

H 4.48292100 6.17935400 -2.29184300

H 4.13526900 5.43940700 -3.86428800

C 6.56916200 1.67758900 -0.69036300

C 6.83613300 1.43875400 0.81828600

C 6.77147900 0.34252700 -1.43948800

C 7.64512300 2.67620600 -1.15941800

H 6.65244100 2.35026100 1.39973200

H 6.21748700 0.64070000 1.23703200

H 7.88326900 1.14897100 0.96414400

H 6.62932300 0.46523300 -2.51715400

H 7.78877100 -0.03408600 -1.27232600

H 6.06680300 -0.42067000 -1.09225700

H 8.62469200 2.30055200 -0.83866900

H 7.68005600 2.79034800 -2.24332100

H 7.50487200 3.66602400 -0.71393500

C -2.84792700 4.51896200 0.12552300

C -2.45338400 5.60795000 1.14768000

C -1.66491300 4.36047800 -0.85132400

C -4.06255400 4.99185300 -0.70005900

H -3.25815900 5.79664500 1.86189500

H -1.56003700 5.30512800 1.70653500

H -2.22679500 6.54740100 0.62820100

H -1.88455000 3.65142800 -1.65819500

H -1.44978700 5.33015300 -1.31287900

H -0.75716600 4.02513300 -0.33953300

H -3.76596800 5.82983700 -1.34304500

H -4.43624400 4.18996400 -1.34776800

H -4.88039500 5.33649100 -0.06607900

C -4.86649300 1.89353200 4.15392900

C -6.02161300 0.87855200 4.02873700

C -3.88466000 1.38800000 5.24058600

C -5.42463700 3.22618500 4.69268000

H -6.74478500 1.18426800 3.26512100

H -5.65041600 -0.11683800 3.76262000

H -6.55505700 0.79362500 4.98376400

H -3.05845800 2.09447100 5.37426800

H -4.41340100 1.29924700 6.19681200

H -3.45964600 0.40772700 5.00955200

H -5.77648800 3.05851800 5.71795300

H -4.65466000 4.00289400 4.72684600

H -6.27091800 3.60597200 4.11964100

C -6.71868900 -1.49712400 -0.45077600

C -6.98921300 -1.38180900 1.07065400

C -6.85202200 -0.09266400 -1.08013200

C -7.83502700 -2.40291200 -1.00624000

H -6.88690700 -2.35711400 1.56169400

H -6.31414500 -0.68184700 1.56621900

H -8.01203700 -1.02384000 1.23732700

H -6.65408500 -0.11131400 -2.15661800

H -7.86755700 0.29382700 -0.92698200

H -6.15174300 0.61765300 -0.62549600

H -8.79676800 -2.01649100 -0.64729800

H -7.88192400 -2.41240400 -2.09533500

H -7.73726600 -3.43391000 -0.65385900

C -3.10882300 -4.95775400 -1.97505300

C -3.24330600 -4.97943100 -3.51222100

C -1.64621600 -5.31139900 -1.64613500

C -3.99804100 -6.06400100 -1.36385400

H -4.28519800 -4.98890300 -3.83207700

H -2.74388000 -4.11595700 -3.96654300

H -2.76918700 -5.88612600 -3.90767500

H -1.46239300 -5.33994300 -0.56758900

H -1.42065300 -6.30558900 -2.04747200

H -0.93990900 -4.60604600 -2.09808800

H -3.72086300 -7.04098600 -1.77923300

H -3.86678900 -6.11056200 -0.27594700

H -5.05538000 -5.88974700 -1.57631300

H -0.66504200 3.53312600 4.97645200

H 1.65090100 3.43135700 1.36797700

C 2.75029200 -4.55450200 0.75519400

C 2.51087900 -5.38775200 2.03461800

C 1.44415400 -4.59134800 -0.06232500

C 3.83894600 -5.23652800 -0.09808800

H 3.41021000 -5.43769700 2.65282500

H 1.70304900 -4.95281700 2.63322600

H 2.22221400 -6.41155200 1.76550400

H 1.54759500 -4.08587000 -1.02803200

H 1.18047400 -5.63508100 -0.26295900

H 0.61075000 -4.13375600 0.48009300

H 3.44350300 -6.17276200 -0.51099100

H 4.14293400 -4.60358900 -0.93907100

H 4.72358900 -5.48922600 0.48849200

C 4.95079600 -1.35623000 4.10712500

C 4.67012400 0.03093200 4.71759100

C 4.46109500 -2.41536500 5.12098500

C 6.48028000 -1.47617100 3.94711000

H 5.03709600 0.84452000 4.08108900

H 3.60259700 0.19399500 4.90205600

H 5.18760100 0.10651100 5.68019600

H 4.65750500 -3.42920200 4.76325600

H 4.97622700 -2.28473700 6.08083600

H 3.38375500 -2.31536000 5.29698500

H 6.96822100 -1.15181700 4.87433100

H 6.79426500 -2.50302000 3.75623000

H 6.84950600 -0.83700000 3.13670200

O -4.93614200 4.15506700 2.12211300

O -5.72997800 -3.72796800 -2.27809700

O 5.53205700 3.91080200 -2.44184400

O 5.06707600 -3.75986700 2.39898600

C 6.21269200 -3.65317800 1.53527000

H 6.70232500 -2.68165300 1.64283300

H 6.90032000 -4.44741800 1.83610400

H 5.93286100 -3.78594100 0.48687800

C 5.64950700 3.35022600 -3.75095800

H 6.31386400 4.00945600 -4.31560600

H 6.08157100 2.34394000 -3.71411800

H 4.67433500 3.30090600 -4.25019000

C -6.23783500 3.88480700 1.58528800

H -6.72608200 3.05683300 2.10861600

H -6.82407100 4.79700000 1.72062200

H -6.18013100 3.63987300 0.51909300

C -5.94697700 -3.02349500 -3.50917900

H -5.07358500 -3.09848400 -4.16314300

H -6.80221600 -3.50179100 -3.99300900

H -6.16158800 -1.96637100 -3.33787500

Ni -0.14125500 0.00682800 -0.76357500

C 1.55635100 -0.31645200 -3.06303700

C 1.81623400 -1.67993000 -2.82916100

C 3.08189500 -2.23577100 -3.02257600

C 4.13179900 -1.43295300 -3.48475500

C 3.88691000 -0.08371600 -3.76877100

C 2.62492600 0.45822200 -3.55978300

C 0.27230300 0.33041600 -2.73891800

C -0.97249700 -0.33090700 -2.53545300

C -2.28855500 0.38027200 -2.76596100

H -1.01516400 -1.41017000 -2.69315600

H 0.24135900 1.39684300 -2.97138800

H 1.02406500 -2.31731800 -2.44795300

H 2.46318300 1.51382300 -3.75830300

H 3.23775100 -3.28580000 -2.80201500

H 4.69963100 0.53041800 -4.14261700

O 5.41505600 -1.87434800 -3.69533800

C 5.76697300 -3.14458400 -3.15686500

H 6.83787900 -3.26189200 -3.33709800

H 5.57149000 -3.18395500 -2.07812700

H 5.22769700 -3.96091800 -3.65370800

C -4.14525300 2.57614800 -4.26815400

C -4.11980500 1.06558700 -4.27286600

C -2.75861100 0.39602600 -4.25572100

C -2.80117200 -1.00926000 -4.85404800

O -5.15997100 0.41330700 -4.26275200

H -3.41210400 2.98763400 -3.56609600

H -3.86494600 2.93490200 -5.26817500

H -5.14585200 2.94352200 -4.02601700

H -3.37539000 -1.68510800 -4.21387800

H -3.26488300 -1.00648400 -5.84708400

H -1.78886200 -1.41286600 -4.95283600

H -2.21647100 1.41415900 -2.41153300

H -3.07460600 -0.10439800 -2.17508800

H -2.05596100 1.02525700 -4.81776800

**B3**

C -0.56549100 3.17600000 4.05064000

C -0.71828800 1.79252300 3.97623600

C -0.06082100 1.02727000 3.00082000

C 0.79842900 1.68826100 2.08628800

C 0.96642700 3.07684400 2.19115500

C 0.29002600 3.82061700 3.15778300

C -0.24090200 -0.46126500 3.08455600

C 0.26249400 -1.07102600 4.24407400

C 0.15435500 -2.44310200 4.46163000

C -0.50229600 -3.23427600 3.52060500

C -1.04155100 -2.64241400 2.37862100

C -0.91832900 -1.26656700 2.13500000

P 1.53222200 0.77540100 0.66726100

P -1.50206600 -0.54860200 0.53677900

C 2.85806100 1.83348800 -0.03481100

C 2.46493100 -0.59880500 1.43342700

C -2.54795100 0.84862300 1.08226100

C -2.72667700 -1.75768800 -0.10635700

C -2.41554700 2.08178800 0.44620700

C -3.25158100 3.16315100 0.75542300

C -4.28980200 2.91725100 1.68345400

C -4.33009500 1.74889500 2.48610300

C -3.45272800 0.71795500 2.13869600

C -2.25234400 -2.92915400 -0.69983300

C -3.10313200 -3.85748900 -1.31057800

C -4.48163200 -3.53283300 -1.36613000

C -5.01193600 -2.39803900 -0.70925000

C -4.09847000 -1.51957600 -0.11217300

C 2.49633400 2.91608100 -0.84912300

C 3.43014600 3.58889500 -1.63805200

C 4.76523800 3.09580300 -1.63795300

C 5.19277700 2.09876100 -0.73739100

C 4.19888200 1.47088500 0.03208800

C 2.39999100 -1.84070600 0.79772500

C 3.18382100 -2.91952800 1.20646800

C 4.07266800 -2.69690100 2.28919500

C 4.03639800 -1.51517600 3.06682400

C 3.24578900 -0.46231100 2.57800000

H -1.10292900 3.74105200 4.80673400

H 1.63916300 3.58556400 1.50938200

H 0.43823800 4.89533400 3.21319900

H -0.61036000 -4.30419900 3.67592200

H -1.63637000 2.18883500 -0.29595800

H -3.46472100 -0.21103000 2.69283000

H -1.18331900 -3.11382200 -0.69763700

H -4.47127400 -0.61489000 0.34454000

H 1.45390500 3.20630600 -0.88532500

H 4.48351000 0.64973200 0.67317800

H 1.71902300 -1.94271300 -0.03685500

H 3.24110800 0.48752300 3.10105300

H 0.76720000 -0.44979700 4.97650500

H -1.36927500 1.28445100 4.68152100

C -2.42807600 -5.14935000 -1.83573000

C -1.38348100 -4.77413900 -2.91076000

C -1.71023500 -5.82046400 -0.63832600

C -3.35474300 -6.22218500 -2.43705400

H -1.86467600 -4.29956900 -3.77407400

H -0.62384100 -4.08552600 -2.52721500

H -0.86792300 -5.67547300 -3.26479200

H -2.43636700 -6.12061800 0.12685500

H -1.18170900 -6.72052200 -0.97524700

H -0.97718100 -5.16243300 -0.16566500

H -2.74584400 -7.10764300 -2.65900100

H -4.14396000 -6.52280400 -1.74355600

H -3.81917200 -5.90553200 -3.37290300

C -6.50847300 -2.01801800 -0.57475900

C -6.79285600 -1.65511100 0.90587300

C -6.79411500 -0.77583200 -1.44597000

C -7.51795300 -3.12265600 -0.94285000

H -6.55430600 -2.49500600 1.56946900

H -6.22544800 -0.78464400 1.24530800

H -7.85625700 -1.41821000 1.02794500

H -6.64230400 -0.99104500 -2.50785400

H -7.83384800 -0.45119500 -1.31112200

H -6.14099100 0.06082100 -1.17587000

H -8.52000200 -2.78007300 -0.65638700

H -7.54278100 -3.34159000 -2.01083900

H -7.31731400 -4.05442200 -0.40500200

C 3.00189100 -4.29989300 0.54017400

C 2.59332200 -5.32258100 1.62342800

C 1.87065800 -4.26624300 -0.50718900

C 4.27558900 -4.78359800 -0.18469400

H 3.36393300 -5.42153500 2.39166900

H 1.65951100 -5.01513200 2.10927800

H 2.43150300 -6.30821100 1.16940400

H 2.10924700 -3.61017500 -1.35259200

H 1.72043900 -5.27588300 -0.90418500

H 0.92222100 -3.93457500 -0.07347100

H 4.04204400 -5.67360600 -0.78208300

H 4.65719200 -4.01499400 -0.86679300

H 5.06892600 -5.05394600 0.51295800

C 4.75724000 -1.28470600 4.41952700

C 5.88227800 -0.24347400 4.24359200

C 3.72538500 -0.72426100 5.43023700

C 5.33966500 -2.54830600 5.08449500

H 6.64894300 -0.59281500 3.54416200

H 5.49084000 0.70771600 3.86715700

H 6.36952300 -0.04880900 5.20723900

H 2.91214600 -1.44010000 5.59032200

H 4.21785100 -0.54841200 6.39370500

H 3.28576400 0.22405800 5.11034800

H 5.64880100 -2.28611100 6.10364800

H 4.59486500 -3.34681400 5.15624700

H 6.21802200 -2.94245300 4.57288600

C 6.64406400 1.60623000 -0.51679000

C 6.89417000 1.45152300 1.00485200

C 6.81032800 0.21501100 -1.16899500

C 7.74307000 2.56051800 -1.02546000

H 6.71487900 2.39478100 1.53455400

H 6.26745200 0.68218800 1.45971100

H 7.93691000 1.15993300 1.17529400

H 6.62842000 0.24297800 -2.24707800

H 7.82686200 -0.16367000 -1.00318100

H 6.10682500 -0.50402900 -0.73229600

H 8.71148800 2.20018200 -0.65817400

H 7.80962200 2.61262800 -2.11146500

H 7.59848400 3.57622300 -0.64213300

C 3.01068700 4.84345000 -2.43542900

C 3.10782900 4.63117100 -3.96148800

C 1.55305800 5.23892100 -2.12951600

C 3.90319200 6.03271100 -2.01576100

H 4.14025200 4.57545500 -4.30677300

H 2.58763500 3.71505000 -4.26583300

H 2.63198700 5.47325400 -4.47915100

H 1.38885500 5.40875400 -1.06036400

H 1.31936600 6.17195000 -2.65412700

H 0.83887900 4.48239900 -2.47245100

H 3.60812400 6.93478700 -2.56620200

H 3.79225800 6.24098800 -0.94458400

H 4.95731900 5.83543400 -2.22117400

H 0.57528500 -2.88502200 5.36026300

H -1.57381100 -3.26471500 1.66873600

C -2.93175600 4.57436300 0.20920600

C -2.75611300 5.53119400 1.41026000

C -1.60100100 4.57183100 -0.56885500

C -4.00929400 5.14231500 -0.73666500

H -3.67672600 5.61414600 1.99270600

H -1.95823400 5.17781300 2.07238300

H -2.48454300 6.53279100 1.05391800

H -1.65985300 3.97499700 -1.48494500

H -1.35799400 5.59939300 -0.85946000

H -0.77408100 4.19157600 0.03921300

H -3.62493300 6.04758400 -1.22255900

H -4.26830200 4.42611700 -1.52395200

H -4.91910300 5.42207800 -0.20324200

C -5.16978500 1.65101000 3.78081000

C -4.87851100 0.33807900 4.53382100

C -4.75248100 2.81818000 4.70418200

C -6.69400700 1.70685600 3.54969200

H -5.19449700 -0.54335500 3.96354700

H -3.81629400 0.22704100 4.77774600

H -5.43623400 0.33838900 5.47667900

H -4.96159400 3.78584000 4.24099300

H -5.30345700 2.76530000 5.65148100

H -3.68099500 2.76992100 4.93070300

H -7.21125000 1.45912300 4.48468000

H -7.02852000 2.69957200 3.24643900

H -7.01121600 0.98028300 2.79277300

O 4.98857400 -3.69361100 2.57733600

O 5.64352000 3.63873900 -2.56044000

O -5.32703600 -4.34719900 -2.09545900

O -5.28857600 3.86797300 1.83793700

C -6.39329000 3.63684500 0.94546000

H -6.85258100 2.66141300 1.12663800

H -7.12013900 4.42857400 1.14299500

H -6.07522300 3.68161100 -0.09943800

C -5.47749100 -3.92450500 -3.45209100

H -6.10216100 -4.67321300 -3.94605100

H -5.96781500 -2.94632200 -3.51085600

H -4.50716000 -3.86719400 -3.95987300

C 6.29817500 -3.41160200 2.06663000

H 6.74199100 -2.53857500 2.55482400

H 6.90856100 -4.29356200 2.27552600

H 6.26919400 -3.23213000 0.98639200

C 5.84841000 2.81371600 -3.71606200

H 4.89751200 2.45539200 -4.11996300

H 6.35230600 3.44002800 -4.45675800

H 6.47776600 1.95032400 -3.48759100

Ni 0.12316000 0.01219700 -0.77124800

C -1.56289000 0.02019300 -3.09674200

C -1.88190600 1.38937400 -3.02997900

C -3.17352300 1.86058700 -3.27100700

C -4.18988400 0.96105100 -3.61445400

C -3.88538200 -0.40023300 -3.73842700

C -2.59828500 -0.85603600 -3.48329400

C -0.25151700 -0.52591200 -2.70187000

C 0.96031400 0.21084900 -2.56933900

C 2.31018100 -0.46069000 -2.70400300

H 0.96233600 1.26404200 -2.86276900

H -0.17080400 -1.60936300 -2.81190500

H -1.11641500 2.10309900 -2.74081600

H -2.39055400 -1.91950800 -3.55369700

H -3.37578400 2.92197400 -3.18019700

H -4.67220500 -1.09042100 -4.02422600

O -5.49476700 1.31644600 -3.85431700

C -5.90509000 2.61430800 -3.43591400

H -6.98362300 2.65797200 -3.60331400

H -5.69371900 2.77024700 -2.37072300

H -5.41897600 3.40397400 -4.02237700

H 2.29249700 -1.43634900 -2.20676500

H 3.07028600 0.14066400 -2.19292700

C 2.76002800 -0.66714600 -4.17652400

C 4.17424300 -1.19628900 -4.18985200

C 4.36029600 -2.68064700 -3.94075300

C 5.78860100 -3.07428100 -3.57902100

O 5.13335300 -0.44600500 -4.34584800

H 2.73297500 0.28834700 -4.71114200

H 2.07606100 -1.37101300 -4.66546800

H 3.65115200 -2.99900700 -3.16598100

H 4.03227000 -3.19313100 -4.85843700

H 5.86457100 -4.15725700 -3.43477700

H 6.49006900 -2.78445300 -4.36810700

H 6.10881100 -2.58379800 -2.65271600

**3b**

C 1.27241800 0.51228400 0.26077600

C 1.88708000 -0.75104800 0.28223100

C 3.26050100 -0.90258100 0.10398600

C 4.06426200 0.22703200 -0.10444300

C 3.47320700 1.49766900 -0.12953300

C 2.10308800 1.63035600 0.05061400

C -0.16969200 0.71991400 0.44128700

C -1.11126500 -0.21386500 0.65173700

C -2.57043500 0.09821900 0.80452900

H -0.83544100 -1.26542600 0.70729500

H -0.48664400 1.76296800 0.39530300

H 1.28865300 -1.64330200 0.44107400

H 1.65915300 2.62277200 0.02815000

H 3.69224100 -1.89626700 0.12870300

H 4.10407100 2.36674400 -0.29160700

O 5.41551400 0.19056700 -0.29095600

C 6.05811100 -1.08376000 -0.27528500

H 7.11837000 -0.88380800 -0.44155100

H 5.92854000 -1.58346700 0.69219900

H 5.67902600 -1.73091700 -1.07536300

C -5.20031400 1.46389300 -0.70576900

C -4.85094500 0.09756400 -0.17024100

C -3.42714900 -0.39066800 -0.40377800

C -3.37444300 -1.90884300 -0.58160000

O -5.66563400 -0.58294000 0.44112900

H -4.41287500 2.18972500 -0.47294100

H -5.26589600 1.40628900 -1.80087800

H -6.15864900 1.80450700 -0.30601100

H -3.70038900 -2.42106800 0.32973800

H -4.02860300 -2.22910100 -1.40039000

H -2.35751000 -2.23766700 -0.81635900

H -2.70332900 1.17973000 0.92752000

H -2.96833800 -0.38464300 1.70829400

H -3.03797400 0.10492900 -1.30162300

**4b**

C 1.61048000 0.56121400 0.09956200

C 2.18888900 -0.65441500 0.50140200

C 3.55030200 -0.90740400 0.34556100

C 4.37902800 0.06937000 -0.22383400

C 3.82483600 1.29122300 -0.62914300

C 2.46617700 1.52623800 -0.46663000

C 0.18172900 0.86988800 0.24245800

C -0.78904100 0.06000100 0.69338200

C -2.23147900 0.45612000 0.80695800

H -0.55338300 -0.96175900 0.99350100

H -0.09694000 1.88033900 -0.06071900

H 1.57072800 -1.42538000 0.95215300

H 2.05112000 2.47936200 -0.78567800

H 3.95381200 -1.85881200 0.67172700

H 4.47511500 2.04261000 -1.06752900

O 5.72225100 -0.07407700 -0.41995900

C 6.32923700 -1.30094100 -0.01657400

H 7.38903600 -1.20187100 -0.25920800

H 6.21617200 -1.46617600 1.06168700

H 5.90837200 -2.15296300 -0.56389400

H -2.35461500 1.51322700 0.54431000

H -2.56862000 0.34798700 1.84725900

C -3.13671000 -0.40371500 -0.08392100

C -4.61216100 -0.07658900 0.04401300

C -5.55135500 -0.80984800 -0.89399900

C -7.02680100 -0.51194900 -0.65041800

O -5.02310600 0.73689500 0.86176500

H -3.01440200 -1.47088900 0.15271000

H -2.85079400 -0.30598200 -1.14004800

H -5.25944900 -0.54233700 -1.91991200

H -5.34164500 -1.88502800 -0.80662700

H -7.65078100 -1.06641000 -1.35946300

H -7.32606500 -0.79909100 0.36348400

H -7.23960600 0.55545000 -0.77110900

**TS1**

**Imaginary frequency: -268.84 cm^-1^**

C 0.14533100 1.55600600 4.47640800

C 0.00227000 0.25378500 3.99969100

C 0.56008100 -0.14398300 2.77673700

C 1.30723400 0.79875800 2.03069200

C 1.46797600 2.09752600 2.53233300

C 0.88667000 2.48051200 3.74149900

C 0.38538100 -1.57684000 2.37652000

C 0.98922900 -2.54101800 3.19592600

C 0.87358600 -3.90373800 2.92657500

C 0.11667800 -4.32634400 1.83487800

C -0.51058400 -3.38279300 1.02115700

C -0.38395600 -2.00944500 1.26985400

P 1.96349000 0.34577500 0.37303500

P -1.14370500 -0.78932500 0.11594100

C 3.12033200 1.67202900 -0.11959000

C 3.00346400 -1.11347600 0.68270600

C -2.12705300 0.27417700 1.21756200

C -2.39303100 -1.73933400 -0.82446300

C -2.10820800 1.65684000 1.04523300

C -2.92436200 2.49764200 1.81440900

C -3.83306300 1.86998100 2.69806500

C -3.73515000 0.49725300 3.03798100

C -2.87971400 -0.28153300 2.25614300

C -1.97866500 -2.50044900 -1.92115400

C -2.89044400 -3.15617100 -2.75660200

C -4.27004400 -2.97786600 -2.47227300

C -4.71988000 -2.31029200 -1.31013900

C -3.75045800 -1.67499400 -0.52534400

C 2.58967100 2.90648300 -0.52135300

C 3.38275200 3.88362600 -1.12273100

C 4.74500200 3.55667700 -1.37073300

C 5.34217500 2.38823600 -0.85410400

C 4.48406100 1.44586900 -0.26154900

C 2.87521600 -2.22579500 -0.15242400

C 3.69639000 -3.34584100 -0.00114700

C 4.69293200 -3.28633100 1.00669100

C 4.72609300 -2.26079500 1.98019200

C 3.88833200 -1.15924000 1.75819300

H -0.31329400 1.84139200 5.41861500

H 2.05894100 2.81819800 1.97885300

H 1.02142500 3.49447800 4.10657300

H 0.00546000 -5.38430000 1.61628600

H -1.43958300 2.07388300 0.30404800

H -2.78986000 -1.34208300 2.44824200

H -0.91922800 -2.55898600 -2.14073100

H -4.07134600 -1.09471400 0.32650600

H 1.53182400 3.08553300 -0.37767900

H 4.89365500 0.50303600 0.07276100

H 2.12001300 -2.20351900 -0.92660300

H 3.92115700 -0.31781200 2.44117400

H 1.57208700 -2.20653400 4.04798100

H -0.56426500 -0.47311300 4.57367500

C -2.30601600 -3.98048000 -3.93015300

C -1.80606100 -3.01262800 -5.02511300

C -1.09796800 -4.80084000 -3.41358300

C -3.27493300 -4.99539500 -4.57040800

H -2.61777700 -2.37838500 -5.39783800

H -1.00603200 -2.36479000 -4.65423700

H -1.40418900 -3.57969900 -5.87391900

H -1.38381200 -5.43468400 -2.56539500

H -0.72894100 -5.45174800 -4.21313700

H -0.26224100 -4.16982600 -3.10659500

H -2.69896600 -5.63702500 -5.24820900

H -3.74582500 -5.63871800 -3.81978600

H -4.06298700 -4.52303100 -5.15735800

C -6.18264700 -2.19418200 -0.81296900

C -6.21687900 -2.55048400 0.69547300

C -6.66030900 -0.73601100 -0.98360900

C -7.20125100 -3.12709900 -1.49608300

H -5.84317100 -3.56701000 0.86730600

H -5.62453200 -1.86465100 1.30709000

H -7.24941600 -2.50259400 1.06034300

H -6.69193100 -0.45347800 -2.04144000

H -7.67128100 -0.61970200 -0.57313800

H -6.00154500 -0.03137600 -0.46408100

H -8.14869700 -3.05895100 -0.94736300

H -7.40409300 -2.85191300 -2.53180700

H -6.87638900 -4.17180600 -1.47473100

C 3.45802400 -4.60784900 -0.85773000

C 3.28556200 -5.83196400 0.06937600

C 2.16162600 -4.48010900 -1.68113100

C 4.61037600 -4.86225900 -1.85141500

H 4.18167500 -6.02049500 0.66356800

H 2.44282400 -5.68031800 0.75322200

H 3.07684000 -6.72579900 -0.53143300

H 2.22542400 -3.70124400 -2.44718600

H 1.97496900 -5.42869400 -2.19624300

H 1.29518400 -4.27404700 -1.04418700

H 4.33769600 -5.68174100 -2.52799200

H 4.80435700 -3.97386900 -2.46415200

H 5.53490600 -5.14623600 -1.34614400

C 5.55675600 -2.26020400 3.28749300

C 6.63761400 -1.16150400 3.21960600

C 4.59793800 -1.94180100 4.46317300

C 6.22422600 -3.60154600 3.65122100

H 7.33964600 -1.34136000 2.39818800

H 6.19304000 -0.17142000 3.07304100

H 7.21101700 -1.13755400 4.15465800

H 3.82242300 -2.71121800 4.54859300

H 5.16368400 -1.92511300 5.40197600

H 4.10360100 -0.97186100 4.36213900

H 6.61671900 -3.52130300 4.67222400

H 5.50774300 -4.42831100 3.63264700

H 7.06268700 -3.85868800 3.00334000

C 6.85047400 2.03842600 -0.86173200

C 7.24067000 1.51929600 0.54568000

C 7.11038500 0.90704400 -1.87975800

C 7.79696400 3.22071200 -1.15095200

H 7.02023800 2.26633500 1.31756800

H 6.72618600 0.59384700 0.81222100

H 8.31635400 1.31076900 0.57284300

H 6.85221600 1.21260000 -2.89908800

H 8.17040500 0.62419200 -1.86885200

H 6.52082300 0.01499800 -1.63851100

H 8.82463200 2.89646200 -0.94755200

H 7.76514900 3.56051600 -2.18604000

H 7.58213000 4.07594200 -0.50197600

C 2.79566200 5.27755200 -1.43560900

C 2.75678700 5.58074800 -2.94845000

C 1.35257500 5.40530000 -0.91146000

C 3.63967600 6.35088600 -0.71204000

H 3.75253700 5.73623300 -3.36406200

H 2.26926800 4.77009000 -3.50284000

H 2.17988300 6.49747900 -3.12185100

H 1.28884900 5.21298700 0.16421600

H 0.99786900 6.42656600 -1.08822900

H 0.66306600 4.72594000 -1.42438400

H 3.22570400 7.34746200 -0.90951900

H 3.62507400 6.18900200 0.37264500

H 4.67845300 6.33878700 -1.04866100

H 1.36804800 -4.62667600 3.56895100

H -1.11051200 -3.72310100 0.18485300

C -2.72513500 4.02956800 1.77281300

C -2.49290400 4.54054800 3.21258100

C -1.46693300 4.39328500 0.96042000

C -3.91677100 4.78164000 1.14425700

H -3.35628900 4.34700600 3.85231900

H -1.61621200 4.05591500 3.65708500

H -2.31183300 5.62251500 3.19812200

H -1.57478800 4.14946900 -0.09966500

H -1.29602000 5.47258200 1.03543300

H -0.57666800 3.88554500 1.34528500

H -3.62016000 5.81261800 0.91532200

H -4.23702500 4.30732000 0.21118800

H -4.77174300 4.83281900 1.82101800

C -4.40163000 -0.10526800 4.29557900

C -3.96840300 -1.56938800 4.50549500

C -3.91855700 0.70239600 5.52159800

C -5.94357100 -0.09334000 4.25367200

H -4.32260600 -2.22410700 3.70065600

H -2.88025600 -1.67327000 4.57953400

H -4.40013500 -1.93631000 5.44297600

H -4.21977900 1.75061100 5.45113800

H -4.34685000 0.28367000 6.44067000

H -2.82627300 0.66274900 5.60709700

H -6.33314500 -0.69014400 5.08731000

H -6.34964400 0.91357900 4.35655200

H -6.32208200 -0.53435300 3.32431600

O 5.64937800 -4.28462600 1.02104100

O 5.48063600 4.43874500 -2.14116700

O -5.20486000 -3.47308300 -3.35702500

O -4.83562200 2.63058300 3.27872900

C -6.03303500 2.64722700 2.47911700

H -6.44540100 1.64145800 2.36262600

H -6.74815400 3.27930300 3.01101200

H -5.84115900 3.06125500 1.48620300

C -5.59340000 -2.52825900 -4.35731700

H -6.35446500 -3.01769700 -4.97006700

H -6.01525900 -1.62296000 -3.90523300

H -4.74316000 -2.24679100 -4.98885000

C 6.89785300 -3.86507800 0.45357000

H 7.36594900 -3.07534600 1.04953600

H 7.54409800 -4.74604300 0.44435700

H 6.76013600 -3.49844500 -0.56949800

C 5.62773700 4.01738200 -3.50473400

H 4.65512400 3.79400400 -3.95506300

H 6.09622600 4.84970600 -4.03553800

H 6.26396000 3.13158800 -3.58670600

Ni 0.36802200 0.17586700 -1.11002500

C -1.81668600 1.37407600 -2.76114400

C -1.74447500 2.76095900 -2.54842800

C -2.88774800 3.55651400 -2.46432400

C -4.15206600 2.96677200 -2.56994700

C -4.24891300 1.58480800 -2.78646200

C -3.10217900 0.80819900 -2.88790500

C -0.62352700 0.50996900 -2.81858800

C 0.70919800 1.02443800 -2.90381700

C 1.81451400 0.17209000 -2.85417400

H 0.89611700 2.09495500 -2.87295800

H -0.80220200 -0.48730600 -3.21736500

H -0.77860100 3.24413100 -2.44326700

H -3.19852000 -0.25692900 -3.07178400

H -2.78115100 4.62310300 -2.30546700

H -5.23355100 1.13933500 -2.88429100

H 2.79840700 0.56511300 -2.64504400

H 1.70482400 -0.90473100 -2.86830400

O 2.58361000 -0.13427900 -4.96230000

H 1.78180100 0.27673700 -5.32528900

O -5.33502000 3.64955800 -2.48020400

C -5.28535400 5.07493800 -2.53014400

H -6.32501200 5.40850600 -2.54540700

H -4.78565900 5.49592100 -1.65092200

H -4.77666900 5.41941000 -3.43903900

C 2.07424700 -4.73122500 -5.66191000

C 2.49787800 -3.26848300 -5.74295200

H 2.24459900 -5.13235800 -4.65696400

H 1.00652800 -4.83123500 -5.89288000

H 2.63868800 -5.34344400 -6.37603500

C 2.27018600 -2.69251000 -7.14470200

H 3.58183500 -3.21368000 -5.52655400

H 2.57039200 -1.63910200 -7.16729700

H 2.84628900 -3.23652800 -7.90420600

H 1.20599600 -2.75158100 -7.40874300

O 1.77697600 -2.52540300 -4.77236300

H 2.10507800 -1.52752200 -4.85565500

**TS1′**

**Imaginary frequency: -244.60 cm^-1^**

C 0.14922100 1.58369400 4.46100000

C -0.00036000 0.27652500 3.99942300

C 0.54951200 -0.13327800 2.77753200

C 1.29139000 0.79959400 2.01428900

C 1.45878400 2.10313700 2.50070300

C 0.88738900 2.49861200 3.71084100

C 0.38237700 -1.56942500 2.38895700

C 1.02620200 -2.52303100 3.18968400

C 0.93191600 -3.88657500 2.91681000

C 0.15476900 -4.31936100 1.84328600

C -0.51532400 -3.38608700 1.05186800

C -0.40798900 -2.01082400 1.30142500

P 1.92488900 0.31729300 0.35616000

P -1.20193500 -0.79113000 0.16765900

C 3.04136900 1.65328500 -0.19471900

C 3.00186000 -1.11413400 0.67296300

C -2.20531400 0.24419800 1.27501400

C -2.42247800 -1.75451200 -0.79307900

C -2.19916000 1.63036800 1.12642100

C -3.02277400 2.44923000 1.91188100

C -3.92266900 1.79613700 2.78656300

C -3.81345300 0.41790500 3.09986900

C -2.95532300 -0.33935600 2.29980200

C -1.96605700 -2.57182400 -1.83157800

C -2.84249900 -3.22248800 -2.70600400

C -4.22769700 -2.96353300 -2.53946300

C -4.73069100 -2.23495700 -1.43705900

C -3.79356100 -1.62346900 -0.59421200

C 2.48125000 2.88114300 -0.57724500

C 3.24866600 3.87889100 -1.17854800

C 4.61057200 3.57547900 -1.45760400

C 5.23611700 2.40999200 -0.96789700

C 4.40519600 1.44927300 -0.36660500

C 2.90597900 -2.23001800 -0.16097000

C 3.77010000 -3.31792700 -0.02755700

C 4.76483500 -3.23347000 0.98028700

C 4.77020300 -2.20756000 1.95481300

C 3.89906400 -1.13047400 1.73860400

H -0.30115300 1.88029900 5.40371900

H 2.04586400 2.81760700 1.93525400

H 1.02657400 3.51600700 4.06454900

H 0.05857100 -5.37850900 1.62294400

H -1.53411000 2.06681200 0.39273500

H -2.85943400 -1.40368700 2.46759100

H -0.89674400 -2.68462000 -1.97572700

H -4.14708100 -1.00071800 0.21470700

H 1.42364800 3.04224500 -0.40974800

H 4.83593100 0.51177600 -0.04383300

H 2.14616700 -2.23692500 -0.92706300

H 3.91414100 -0.28713700 2.41973800

H 1.62502800 -2.17889600 4.02670900

H -0.56291200 -0.44416800 4.58516200

C -2.19817400 -4.15939000 -3.75734900

C -1.31457200 -3.32992000 -4.71630100

C -1.30309400 -5.17347200 -3.00355100

C -3.17212600 -4.99693500 -4.60863900

H -1.89138700 -2.52408300 -5.18580500

H -0.44393600 -2.88774600 -4.22067900

H -0.92928200 -3.97551300 -5.51554600

H -1.90256200 -5.79727800 -2.32926400

H -0.80383900 -5.83357200 -3.72275300

H -0.52506500 -4.68555400 -2.41181100

H -2.57918500 -5.71203200 -5.19213900

H -3.87391600 -5.56621300 -3.99315000

H -3.74429200 -4.39547400 -5.31731200

C -6.22026400 -2.02010300 -1.07214100

C -6.40571300 -2.32835300 0.43543500

C -6.58816400 -0.54118900 -1.32098900

C -7.23175300 -2.91056600 -1.81916100

H -6.12550000 -3.36420400 0.66114300

H -5.81463800 -1.67066800 1.07863900

H -7.45827200 -2.19419800 0.71063500

H -6.47890700 -0.28062400 -2.37958000

H -7.63090300 -0.35710100 -1.03331800

H -5.95306400 0.13428000 -0.73778600

H -8.21698200 -2.76923300 -1.35790000

H -7.32611100 -2.65589700 -2.87552700

H -6.97581300 -3.97172800 -1.74292600

C 3.57523200 -4.57273200 -0.90580700

C 3.38130300 -5.80123200 0.01140800

C 2.30932700 -4.45691900 -1.78009900

C 4.76729600 -4.81425300 -1.85515400

H 4.25229100 -5.96947100 0.64853500

H 2.50365900 -5.66687600 0.65410700

H 3.22160700 -6.70035300 -0.59652100

H 2.37976600 -3.67320500 -2.54355300

H 2.15771500 -5.40634200 -2.30551800

H 1.41656100 -4.27084900 -1.17354300

H 4.51457100 -5.61246000 -2.56393700

H 4.99753600 -3.91336400 -2.43587700

H 5.66500200 -5.12271200 -1.31766200

C 5.60626400 -2.18064700 3.25869800

C 6.65718100 -1.05350400 3.18262100

C 4.64441000 -1.88360200 4.43764700

C 6.31014200 -3.50278400 3.62454200

H 7.36522200 -1.22110600 2.36379200

H 6.18622600 -0.07692800 3.02716700

H 7.22847800 -1.00628500 4.11806200

H 3.88665200 -2.67026000 4.52594900

H 5.21283800 -1.85402400 5.37448000

H 4.12817900 -0.92498900 4.33845400

H 6.70479300 -3.40840700 4.64349200

H 5.61509400 -4.34780500 3.61245400

H 7.15220000 -3.74111200 2.97429700

C 6.74957500 2.08469800 -1.00853000

C 7.17556000 1.54857900 0.38244100

C 7.00924600 0.97470700 -2.04979300

C 7.67038400 3.28773200 -1.29519000

H 6.95177600 2.27583800 1.17198100

H 6.68788800 0.60616400 0.64064900

H 8.25601900 1.36473600 0.38696700

H 6.73176600 1.29373500 -3.05996300

H 8.07307700 0.70661400 -2.05978900

H 6.43568900 0.07067800 -1.81462800

H 8.70712700 2.97793700 -1.11717300

H 7.61240100 3.64449300 -2.32341800

H 7.45361600 4.12805500 -0.62759700

C 2.63758500 5.27083100 -1.45330200

C 2.54481900 5.58980800 -2.96036300

C 1.21246800 5.37826700 -0.87685000

C 3.49352200 6.34533000 -0.74543900

H 3.52520600 5.75566100 -3.40781900

H 2.04394600 4.78132700 -3.50608200

H 1.95672600 6.50443900 -3.10489800

H 1.18863100 5.16908600 0.19752600

H 0.84439600 6.39942200 -1.02502400

H 0.51033100 4.70188300 -1.37443800

H 3.05980900 7.33854900 -0.91538400

H 3.51786200 6.16957600 0.33693600

H 4.52006900 6.35158800 -1.11720000

H 1.45850200 -4.60220600 3.54157200

H -1.13368500 -3.73822700 0.23471500

C -2.83263700 3.98358900 1.90877500

C -2.58154800 4.45149000 3.36042300

C -1.58841900 4.37576500 1.08890000

C -4.03446500 4.75473200 1.32460000

H -3.43655500 4.23818100 4.00533900

H -1.69878300 3.95527600 3.77898100

H -2.40192700 5.53369800 3.37576200

H -1.70939400 4.15434100 0.02537900

H -1.42470700 5.45466500 1.18549900

H -0.68902800 3.86682300 1.45010500

H -3.74791700 5.80012900 1.15717900

H -4.35457900 4.33592200 0.36504500

H -4.88732200 4.75970900 2.00565000

C -4.46837400 -0.21239700 4.34996400

C -4.01251100 -1.67264600 4.53788300

C -3.99345800 0.58324500 5.58715500

C -6.01036500 -0.22148800 4.31148700

H -4.36063300 -2.32230700 3.72648300

H -2.92246000 -1.76065700 4.60496800

H -4.43383300 -2.05843500 5.47251400

H -4.31084400 1.62759800 5.53484900

H -4.41205400 0.14302000 6.50060300

H -2.90030000 0.55862500 5.66860300

H -6.39000400 -0.83533300 5.13730300

H -6.43018400 0.77816900 4.42931900

H -6.38506600 -0.65425200 3.37651500

O 5.74599600 -4.20709400 0.99556700

O 5.31726800 4.47955000 -2.22963900

O -5.11006300 -3.42423000 -3.49431500

O -4.92801400 2.53730400 3.38692900

C -6.12913200 2.56010700 2.59328500

H -6.53359600 1.55345700 2.45809300

H -6.84708500 3.17521100 3.14097800

H -5.94487800 2.99616700 1.60825500

C -5.32491900 -2.48166900 -4.54796000

H -6.00792100 -2.95522400 -5.25745600

H -5.77628700 -1.55760200 -4.16723700

H -4.38454300 -2.23346700 -5.05509500

C 6.98632600 -3.75664500 0.43340700

H 7.42809800 -2.95050600 1.02710000

H 7.65687500 -4.61927700 0.43421500

H 6.84533400 -3.40076700 -0.59283000

C 5.44242000 4.08177000 -3.60194500

H 4.46469700 3.84458100 -4.03412100

H 5.88098900 4.93171200 -4.13040200

H 6.09575600 3.21143300 -3.71172600

Ni 0.31430400 0.11007500 -1.09907200

C -1.76589200 1.45518200 -2.71836900

C -1.46337900 2.82092000 -2.59302900

C -2.46023500 3.78887900 -2.46768700

C -3.80357400 3.39814800 -2.43465800

C -4.12968700 2.03975900 -2.56258200

C -3.12812500 1.08997700 -2.71252300

C -0.72653300 0.41680600 -2.79136200

C 0.65734300 0.70395600 -3.01161200

C 1.59707100 -0.30151200 -2.77418700

H 1.00891200 1.71831300 -3.18680400

H -1.08363200 -0.57163700 -3.07070800

H -0.42866400 3.14663300 -2.58558100

H -3.39806500 0.04424400 -2.82299100

H -2.17853500 4.83155800 -2.38165700

H -5.17553300 1.75011300 -2.55117700

H 2.65512100 -0.07635700 -2.73475800

H 1.30312800 -1.32806800 -2.61696300

O 1.92049200 -1.76881100 -4.62646300

H 1.01817300 -1.51534400 -4.87782400

O -4.85578800 4.25850800 -2.28004600

C -4.57492200 5.65734600 -2.28786600

H -5.54076300 6.15540400 -2.18261700

H -3.92749500 5.94335000 -1.45179100

H -4.10730800 5.95970700 -3.23306800

**TS1′′**

**Imaginary frequency: -264.28 cm^-1^**

C 0.15664400 1.58078700 4.51002200

C 0.01735700 0.27643900 4.03789600

C 0.59236500 -0.12913500 2.82540100

C 1.35372800 0.80827800 2.08738900

C 1.50918300 2.10988400 2.58347700

C 0.91047800 2.50043700 3.78152800

C 0.41512100 -1.56286600 2.42838500

C 1.00270200 -2.52732700 3.25892800

C 0.87867700 -3.89069400 2.99550000

C 0.12740500 -4.31372900 1.90013300

C -0.48364700 -3.36959100 1.07472000

C -0.34491400 -1.99614800 1.31532500

P 2.02667300 0.35611600 0.43843200

P -1.07316700 -0.77879800 0.14135600

C 3.20233000 1.66925800 -0.04283600

C 3.03692700 -1.12473800 0.73431200

C -2.06974600 0.30228600 1.21266900

C -2.30571100 -1.71396000 -0.83628700

C -2.02864900 1.68324200 1.02910300

C -2.85473800 2.54165300 1.76717800

C -3.78993500 1.93338700 2.63700700

C -3.71531600 0.56311700 2.99330100

C -2.85394300 -0.23468000 2.23728300

C -1.87295200 -2.47626800 -1.92486600

C -2.77235800 -3.07378500 -2.81653100

C -4.15381300 -2.84746900 -2.58893300

C -4.62871700 -2.19730500 -1.42698400

C -3.67214000 -1.61177400 -0.58988600

C 2.68011200 2.88999000 -0.49492700

C 3.47965700 3.83726400 -1.13300700

C 4.84339100 3.49682200 -1.35287500

C 5.43366700 2.35415100 -0.77549800

C 4.56730100 1.43633200 -0.15640700

C 2.88314600 -2.22356700 -0.11478600

C 3.68797800 -3.35783900 0.01274900

C 4.69863600 -3.32419800 1.00755000

C 4.75147900 -2.31756100 1.99962200

C 3.92629800 -1.20144400 1.80433600

H -0.31537100 1.87188900 5.44383700

H 2.10794100 2.82640100 2.03239900

H 1.04110300 3.51610400 4.14329800

H 0.00880700 -5.37205400 1.68724300

H -1.33436000 2.08420100 0.30289200

H -2.78299400 -1.29471700 2.44029000

H -0.80770500 -2.57242200 -2.10370100

H -4.00922500 -1.03753600 0.26027100

H 1.62047700 3.07474600 -0.37344300

H 4.97188000 0.50517000 0.21526300

H 2.12139900 -2.17919100 -0.88192800

H 3.97460600 -0.37287400 2.50218600

H 1.57937600 -2.19316400 4.11541500

H -0.56007200 -0.44588400 4.60688300

C -2.15427800 -3.89054500 -3.97774600

C -1.35500100 -2.93372800 -4.89077000

C -1.18495500 -4.93258700 -3.37012700

C -3.14109700 -4.68244500 -4.85715300

H -1.98425800 -2.11657100 -5.26010500

H -0.49416300 -2.49613500 -4.37632000

H -0.97069400 -3.48033800 -5.76023000

H -1.72110800 -5.62778200 -2.71237500

H -0.71779400 -5.51644000 -4.17154100

H -0.38263200 -4.46928100 -2.79280600

H -2.55398900 -5.31765700 -5.53197800

H -3.79155400 -5.33271100 -4.26559100

H -3.76814700 -4.04120000 -5.47882600

C -6.10494000 -2.05487100 -0.97960900

C -6.20241200 -2.48663200 0.50616400

C -6.53555600 -0.57641000 -1.08990300

C -7.12397100 -2.91998900 -1.74562000

H -5.87076200 -3.52376100 0.63592300

H -5.60498100 -1.85548700 1.16998900

H -7.24400800 -2.41891400 0.84110200

H -6.53436800 -0.24077000 -2.13181400

H -7.55225200 -0.45146500 -0.69653200

H -5.87070100 0.08358800 -0.52174000

H -8.09069800 -2.84482700 -1.23256300

H -7.27414000 -2.59120200 -2.77478800

H -6.83412700 -3.97508900 -1.76043600

C 3.42318300 -4.60305200 -0.86013200

C 3.29351500 -5.85041900 0.04245500

C 2.09328500 -4.46660900 -1.62783500

C 4.53650400 -4.81798200 -1.90573000

H 4.21406000 -6.05488500 0.59172700

H 2.48152500 -5.71668500 0.76672400

H 3.05679600 -6.72808500 -0.57153700

H 2.12665100 -3.67689000 -2.38358300

H 1.89012000 -5.40755700 -2.15062600

H 1.25533700 -4.27512400 -0.94980000

H 4.26483700 -5.65101000 -2.56604800

H 4.66147300 -3.92456500 -2.52881000

H 5.49502500 -5.06233600 -1.44455700

C 5.59099900 -2.35336400 3.30033300

C 6.67855900 -1.26062800 3.25124400

C 4.64065700 -2.05624900 4.48838800

C 6.25209700 -3.70703700 3.62764200

H 7.36937500 -1.42137700 2.41638400

H 6.23910400 -0.26388000 3.13859300

H 7.26312900 -1.26697100 4.17963300

H 3.86003700 -2.82201600 4.55966400

H 5.21099800 -2.06495500 5.42453400

H 4.15298100 -1.08067500 4.41141600

H 6.65104000 -3.65373000 4.64789400

H 5.53059800 -4.52886800 3.59332100

H 7.08527700 -3.95334200 2.96871100

C 6.94160800 2.00600200 -0.74466600

C 7.31297900 1.58804600 0.70062700

C 7.21174100 0.80741600 -1.67966000

C 7.89381200 3.16392200 -1.10429700

H 7.09533400 2.39394400 1.41177900

H 6.78263700 0.69243000 1.03006000

H 8.38558500 1.36867800 0.75447200

H 6.96123800 1.04300100 -2.71947300

H 8.27220700 0.52874000 -1.64077100

H 6.62219800 -0.06791500 -1.38333100

H 8.91835200 2.85104300 -0.86951100

H 7.87324800 3.43272000 -2.16040200

H 7.67622300 4.06159100 -0.51670000

C 2.89521900 5.21127500 -1.52642800

C 2.88122300 5.43469400 -3.05368600

C 1.44268700 5.36053000 -1.03540000

C 3.72570500 6.32240100 -0.84602200

H 3.88339900 5.57425300 -3.45914300

H 2.40812900 4.59278800 -3.57296700

H 2.30247400 6.33742800 -3.28481400

H 1.35890800 5.22109000 0.04707100

H 1.08876700 6.37062600 -1.26891100

H 0.76512600 4.65414000 -1.52810800

H 3.31554700 7.30687200 -1.10320300

H 3.69269600 6.21814500 0.24529500

H 4.77020900 6.29333600 -1.16386000

H 1.36114900 -4.61404800 3.64645400

H -1.07905500 -3.70879000 0.23461300

C -2.63912300 4.07121100 1.70979900

C -2.42263200 4.59565300 3.14703400

C -1.36804100 4.41396300 0.90846700

C -3.81239900 4.82921500 1.05472700

H -3.29765800 4.41744000 3.77550800

H -1.55790700 4.10675800 3.60990100

H -2.23041500 5.67551700 3.12413300

H -1.46702200 4.15858700 -0.15015900

H -1.18846300 5.49246700 0.97230900

H -0.48607900 3.90346600 1.30860700

H -3.50531600 5.85853900 0.83218900

H -4.10960100 4.35620900 0.11283700

H -4.68329900 4.88415700 1.71012000

C -4.41425100 -0.01820500 4.24337100

C -3.99483000 -1.48194500 4.48267500

C -3.95373900 0.80196600 5.46980500

C -5.95473400 0.00233300 4.16602800

H -4.33587700 -2.14603300 3.67987500

H -2.90912800 -1.59116300 4.58136600

H -4.44882600 -1.83295700 5.41569500

H -4.24663000 1.85098200 5.38001600

H -4.40506600 0.39699200 6.38397800

H -2.86390200 0.75677700 5.58027000

H -6.36701400 -0.58381800 4.99625700

H -6.35717300 1.01252400 4.24987300

H -6.31478100 -0.44545000 3.23244600

O 5.64989800 -4.32724100 0.98827200

O 5.58599100 4.33442200 -2.16487200

O -5.06276500 -3.25516700 -3.54332700

O -4.79553000 2.71133500 3.18826200

C -5.97780800 2.72890800 2.36647000

H -6.39839300 1.72599200 2.25592100

H -6.69593900 3.37564400 2.87617600

H -5.76304200 3.12714700 1.37178800

C -5.35246500 -2.23266400 -4.50046600

H -6.06229600 -2.66215400 -5.21177500

H -5.80298300 -1.35668000 -4.01929600

H -4.44595100 -1.92067500 -5.03320600

C 6.89385600 -3.89983100 0.41682300

H 7.37821400 -3.13549800 1.03276200

H 7.53132300 -4.78588300 0.36803000

H 6.74444100 -3.49696700 -0.59100900

C 5.74237700 3.83161400 -3.50018400

H 4.77204300 3.58857900 -3.94520500

H 6.22257100 4.62728900 -4.07488300

H 6.37148300 2.93723800 -3.52270100

Ni 0.44780100 0.20189000 -1.06175200

C -1.77084400 1.30186300 -2.79744800

C -1.79862300 2.67479700 -2.50689100

C -2.98030500 3.41586800 -2.56158100

C -4.18075200 2.78248100 -2.90666800

C -4.17425200 1.41085700 -3.19889100

C -2.99079400 0.68834800 -3.14213100

C -0.53357600 0.50302900 -2.77310600

C 0.77518000 1.08579200 -2.83999400

C 1.91750700 0.28474500 -2.79401000

H 0.90027400 2.16522500 -2.80743100

H -0.64511300 -0.51016900 -3.15582500

H -0.88349800 3.18832700 -2.22959500

H -3.00238500 -0.36982900 -3.37945000

H -2.95294200 4.47394900 -2.33012100

H -5.10539900 0.93009900 -3.48053900

H 2.88001800 0.71318300 -2.56130200

H 1.84894100 -0.79450500 -2.83823900

O 2.80219300 0.07025400 -4.89836600

H 1.93596500 0.19178900 -5.32320300

O -5.39300400 3.40857700 -2.98932100

C -5.42730900 4.82206600 -2.80227200

H -6.46784400 5.11709000 -2.95218600

H -5.11367700 5.10184000 -1.78979900

H -4.79325100 5.33542000 -3.53577500

C 2.37499600 -4.56380700 -5.53603400

C 2.81033200 -3.10981700 -5.69422900

H 2.85240700 -5.01834100 -4.66069800

H 1.28834000 -4.62220300 -5.39977500

H 2.64521600 -5.15565000 -6.41937100

C 2.18802100 -2.46093100 -6.93617100

H 3.90962600 -3.09879100 -5.82892200

H 2.52521000 -1.42278800 -7.01968600

H 2.47340500 -2.99674000 -7.85071600

H 1.09425700 -2.45860100 -6.85989100

O 2.46378400 -2.40106600 -4.51662300

H 2.65213700 -1.37360000 -4.68563000

O -0.11043900 0.10573600 -5.93855400

H -0.17013200 0.44124500 -5.02772500

C -0.46302600 1.17329600 -6.83642900

C -1.81319700 1.77745300 -6.46233400

C -0.45522400 0.57438900 -8.23473200

H 0.30795500 1.95711300 -6.77754300

H -1.78105000 2.22136100 -5.46133300

H -2.09391600 2.56417600 -7.17284400

H -2.59306700 1.00631200 -6.47143600

H 0.52099600 0.13063200 -8.45976700

H -1.21774900 -0.20996400 -8.31731500

H -0.66617600 1.34450500 -8.98471500

**TS2**

**Imaginary frequency: -66.54 cm^-1^**

C -0.40281100 1.98903900 4.35832900

C -0.46479700 0.64028700 4.01000000

C 0.20080000 0.14642000 2.88015400

C 0.97477800 1.03876900 2.10012300

C 1.05395600 2.38669300 2.47530200

C 0.36444400 2.86451400 3.59031300

C 0.10687000 -1.32479000 2.60995300

C 0.73036600 -2.18016600 3.52867700

C 0.69003000 -3.56552600 3.37819500

C -0.01262400 -4.12083900 2.30971200

C -0.65951900 -3.28632000 1.39792900

C -0.60395300 -1.89159600 1.52480700

P 1.74512400 0.44042400 0.54313200

P -1.35005800 -0.81556200 0.22875800

C 2.86131100 1.75505700 -0.05914200

C 2.82369400 -0.93170600 1.03975700

C -2.44445400 0.29737300 1.15871400

C -2.49114300 -1.88374800 -0.71777100

C -2.48227000 1.65450500 0.84483500

C -3.38474700 2.52114100 1.47650700

C -4.31264600 1.93538100 2.36928400

C -4.17044300 0.61002900 2.85191500

C -3.23393300 -0.19576300 2.20113700

C -1.95671800 -2.73580800 -1.69377700

C -2.77235500 -3.43089200 -2.58631000

C -4.17583000 -3.21662800 -2.48783700

C -4.75212100 -2.50544700 -1.41432400

C -3.87099500 -1.80745200 -0.57379400

C 2.30176400 2.93478800 -0.56790500

C 3.08589200 3.90961400 -1.18597500

C 4.47355000 3.63365900 -1.34292800

C 5.08623700 2.50401000 -0.75601200

C 4.23571100 1.56612400 -0.14628400

C 2.77144000 -2.11517700 0.30116300

C 3.63617700 -3.17743200 0.56759500

C 4.59418500 -2.98747300 1.59590600

C 4.54616600 -1.88513400 2.48157000

C 3.67117000 -0.84404900 2.14155100

H -0.94419600 2.34911600 5.22826200

H 1.66279000 3.07122700 1.89581100

H 0.43607800 3.91461500 3.85826700

H -0.06583300 -5.19835100 2.18466400

H -1.79058200 2.03098400 0.10241000

H -3.10831900 -1.22751500 2.50044400

H -0.88047200 -2.82411900 -1.76616900

H -4.28341100 -1.17550100 0.19920900

H 1.23274200 3.07940800 -0.47987600

H 4.65975400 0.65965900 0.25895200

H 2.04577500 -2.18757400 -0.49695700

H 3.64703000 0.05361600 2.74925400

H 1.26973300 -1.74210300 4.36234600

H -1.05075300 -0.04796200 4.61153100

C -2.15269500 -4.42366200 -3.59341400

C -2.36496400 -3.98484000 -5.05794000

C -0.63292300 -4.56229600 -3.37818100

C -2.77660700 -5.81968300 -3.37281000

H -3.40607000 -4.07621400 -5.36857700

H -2.04335900 -2.94775000 -5.21061200

H -1.76509400 -4.62104400 -5.72011600

H -0.39009200 -4.87584600 -2.35786900

H -0.24423100 -5.32540300 -4.06117000

H -0.09746900 -3.63008700 -3.59125800

H -2.33742500 -6.54040400 -4.07355500

H -2.57873900 -6.17624100 -2.35466500

H -3.85749100 -5.80569300 -3.52943100

C -6.25444000 -2.43061500 -1.04575200

C -6.39099600 -2.70489300 0.47487700

C -6.79536800 -1.01326600 -1.33018800

C -7.15295300 -3.47108200 -1.74304800

H -5.97670500 -3.68582900 0.73623200

H -5.88977300 -1.95112200 1.08813500

H -7.45109800 -2.69880400 0.75312500

H -6.75290000 -0.77322700 -2.39777800

H -7.84205500 -0.93861600 -1.00987400

H -6.22250400 -0.25124000 -0.78989100

H -8.14271600 -3.44221300 -1.27177200

H -7.29615700 -3.27775800 -2.80639300

H -6.75773400 -4.48548900 -1.62856300

C 3.49865700 -4.50246100 -0.21058200

C 3.37963100 -5.67999500 0.78175300

C 2.21568400 -4.50748500 -1.06576400

C 4.69134900 -4.72588300 -1.16394100

H 4.26865200 -5.77357700 1.40794000

H 2.50949400 -5.54595000 1.43532300

H 3.24655400 -6.61905400 0.23036300

H 2.24159700 -3.75033100 -1.85471600

H 2.11513900 -5.48397200 -1.55175800

H 1.32151300 -4.34127900 -0.45574400

H 4.48565900 -5.58307600 -1.81763400

H 4.85085700 -3.84709000 -1.79795100

H 5.61342600 -4.94024900 -0.62063000

C 5.32578200 -1.73881700 3.81150800

C 6.35614700 -0.59661600 3.69286600

C 4.30600800 -1.37527600 4.92126400

C 6.03982600 -3.01213400 4.30651100

H 7.10491400 -0.81102100 2.92282400

H 5.87305200 0.35272800 3.43846900

H 6.88138300 -0.46193800 4.64665300

H 3.55784200 -2.16827800 5.03408300

H 4.82946200 -1.26442900 5.87809900

H 3.77893100 -0.43744900 4.72595200

H 6.39605600 -2.82852300 5.32745800

H 5.36094800 -3.86983800 4.33896600

H 6.90795800 -3.28369000 3.70543400

C 6.60586600 2.20295800 -0.70198500

C 6.94828500 1.55478100 0.66456400

C 6.98318000 1.18693200 -1.80212900

C 7.50068600 3.45435800 -0.81323000

H 6.58939600 2.16752700 1.49967200

H 6.53258900 0.55008800 0.77225000

H 8.03601800 1.46057600 0.75611500

H 6.88944500 1.61485600 -2.80416900

H 8.02488300 0.86650200 -1.67299000

H 6.34301700 0.29956500 -1.76408300

H 8.53522500 3.16573900 -0.59252200

H 7.49233800 3.90683800 -1.80401600

H 7.20566400 4.22043800 -0.08709600

C 2.45319100 5.25808400 -1.59800800

C 2.50793400 5.50049200 -3.12124100

C 0.97022200 5.32984000 -1.18485600

C 3.18381200 6.40336000 -0.86137300

H 3.51907200 5.70738700 -3.47121200

H 2.11633500 4.63835800 -3.67417900

H 1.88809500 6.36909200 -3.37526900

H 0.83678800 5.19323000 -0.10714700

H 0.57603400 6.31831200 -1.44472900

H 0.35765800 4.58484700 -1.70433800

H 2.73299000 7.36704100 -1.12926300

H 3.09705000 6.28163000 0.22521200

H 4.24378200 6.43661700 -1.12112300

H 1.19962000 -4.20321500 4.09471000

H -1.21709700 -3.72891000 0.58026500

C -3.25561800 4.05003600 1.29280900

C -3.11520400 4.70455300 2.68582600

C -1.97841300 4.39602900 0.50345600

C -4.44839700 4.68203200 0.54566700

H -3.99872000 4.52986700 3.30336200

H -2.23983100 4.30734000 3.21229600

H -2.98230300 5.78795800 2.57564700

H -2.02252200 4.03955200 -0.52932700

H -1.86286900 5.48485700 0.47346700

H -1.08463400 3.97772000 0.97773900

H -4.19027500 5.70549000 0.24732200

H -4.69699100 4.11900800 -0.35978400

H -5.33891200 4.74350900 1.17390000

C -4.88131700 0.10012000 4.12608900

C -4.39053700 -1.31001600 4.50864800

C -4.51162300 1.05014700 5.28794600

C -6.41667000 0.02478200 3.99856800

H -4.66124500 -2.05994700 3.75628600

H -3.30529500 -1.34577300 4.65492700

H -4.86125700 -1.60537200 5.45259700

H -4.85702300 2.06887600 5.09512300

H -4.97399900 0.70206000 6.21986500

H -3.42578000 1.07683700 5.43716200

H -6.82667600 -0.50589600 4.86636100

H -6.87577300 1.01378400 3.97490800

H -6.71719500 -0.52638900 3.09987100

O 5.59913600 -3.92913200 1.71606100

O 5.21871800 4.54694400 -2.06610200

O -4.97452600 -3.74033500 -3.48704200

O -5.38237700 2.69508800 2.81476400

C -6.52933000 2.56928000 1.95347500

H -6.89024100 1.53781300 1.92311000

H -7.30154700 3.21761400 2.37431900

H -6.29587600 2.88636900 0.93405200

C -5.40299600 -2.75718600 -4.43988800

H -5.95883400 -3.29689400 -5.21035200

H -6.05303500 -2.00692400 -3.98093800

H -4.54547500 -2.24962900 -4.89383700

C 6.84254400 -3.48640500 1.15312900

H 7.26065200 -2.64476000 1.71411000

H 7.52617300 -4.33698200 1.20895600

H 6.71391000 -3.18345500 0.10868400

C 5.49992200 4.16111700 -3.41837100

H 4.59165600 3.83744000 -3.93354700

H 5.90383100 5.04907300 -3.91118800

H 6.24035200 3.35915600 -3.46371900

Ni 0.23062000 0.03723100 -0.97417200

C -1.77887100 1.07722700 -3.00012000

C -1.68702000 2.47987200 -2.98693000

C -2.82093700 3.29314500 -3.00633500

C -4.09341300 2.71114000 -3.01678300

C -4.20947700 1.31326000 -3.04229600

C -3.07336800 0.51648600 -3.04501300

C -0.60553300 0.19426700 -2.91314700

C 0.74586600 0.66585000 -2.97729100

C 1.84420400 -0.17306300 -2.79478500

H 0.93885800 1.73612800 -2.99768700

H -0.78568200 -0.84844200 -3.16885900

H -0.71561700 2.96290600 -2.96227000

H -3.18297300 -0.56214300 -3.07621800

H -2.69942100 4.36979500 -3.00299500

H -5.19995100 0.87009100 -3.06911400

O -5.26644400 3.41516800 -3.00637100

C -5.19410000 4.82796800 -3.19479500

H -6.22798300 5.17812100 -3.21885200

H -4.66596300 5.31984000 -2.37087500

H -4.70147700 5.07323800 -4.14375700

H 1.72751300 -1.25192600 -2.79248100

H 2.82020200 0.21612000 -2.55503200

C 3.51190000 -2.83690400 -4.62053800

C 4.01093500 -1.47359100 -4.14564800

C 3.61755500 -0.35547800 -4.86960000

C 4.11686100 1.03352500 -4.59569500

O 4.72458900 -1.46295600 -3.07454500

H 2.92070000 -2.78495000 -5.54193400

H 4.36533900 -3.50685500 -4.79328300

H 2.89827500 -3.31019300 -3.84303300

H 3.02741700 -0.50724500 -5.77159600

H 4.58947300 1.08530600 -3.61003100

H 4.86887400 1.37495500 -5.32809400

H 3.30912000 1.78173000 -4.61595500

**TS2′**

**Imaginary frequency: -158.53 cm^-1^**

C -0.45317100 2.15439600 4.48424200

C -0.54990300 0.80814100 4.13531900

C 0.09183100 0.29796000 2.99800700

C 0.87348000 1.17586300 2.20971800

C 0.98061400 2.52332800 2.57974600

C 0.32044400 3.01499700 3.70575800

C -0.02533100 -1.17517700 2.74698800

C 0.55278800 -2.01704900 3.70811500

C 0.50380700 -3.40465800 3.59207800

C -0.16707700 -3.97845300 2.51340900

C -0.77524100 -3.15898100 1.56286100

C -0.71015500 -1.76122500 1.65451700

P 1.64496200 0.58698200 0.65165600

P -1.41873400 -0.71240000 0.31235100

C 2.79996600 1.89354400 0.10427800

C 2.69621000 -0.81148800 1.15166400

C -2.45706100 0.48292500 1.21500800

C -2.62398400 -1.78042300 -0.55833200

C -2.40679600 1.83711000 0.89113400

C -3.27074500 2.76517500 1.48882900

C -4.24240800 2.24912500 2.37744300

C -4.18765500 0.92319200 2.87715100

C -3.29371300 0.05325800 2.24901200

C -2.15002400 -2.76919400 -1.43057100

C -3.01848300 -3.56488700 -2.17914500

C -4.41218900 -3.30336200 -2.06147100

C -4.92781100 -2.40604600 -1.10312400

C -3.99550500 -1.62581800 -0.40225600

C 2.27196200 2.95835000 -0.63504300

C 3.09177100 3.86819300 -1.30099700

C 4.49707400 3.66701800 -1.21575300

C 5.06357900 2.70074400 -0.35318500

C 4.17849300 1.79787200 0.25859900

C 2.68269800 -1.96001000 0.35612700

C 3.51748000 -3.04498700 0.62884400

C 4.40737600 -2.91671300 1.72625800

C 4.32219200 -1.85013700 2.65105700

C 3.47618600 -0.78546900 2.30570600

H -0.97452600 2.52480100 5.36199900

H 1.59074000 3.19460500 1.98491800

H 0.41793200 4.06296000 3.97345600

H -0.23103900 -5.05801500 2.41303900

H -1.67548500 2.16247700 0.16501200

H -3.23891700 -0.98078300 2.56114200

H -1.08036000 -2.90103500 -1.52835400

H -4.36160000 -0.87197200 0.27786200

H 1.19653800 3.03518500 -0.72675400

H 4.58020500 0.98933400 0.85136300

H 2.00412500 -1.98828900 -0.48633000

H 3.41988400 0.08224200 2.95311800

H 1.06442600 -1.56399900 4.55030500

H -1.14343400 0.13344300 4.74472700

C -2.46137100 -4.72264600 -3.03682700

C -2.73858200 -4.53221600 -4.54306200

C -0.93509900 -4.85793000 -2.87202600

C -3.09052200 -6.04989700 -2.55703700

H -3.79339500 -4.65871900 -4.78708900

H -2.41570300 -3.54119600 -4.88366100

H -2.17603300 -5.28010800 -5.11518400

H -0.64823200 -5.02199300 -1.82868100

H -0.59046700 -5.72252900 -3.44965700

H -0.39689000 -3.97848400 -3.24445100

H -2.69270100 -6.88640200 -3.14489100

H -2.84948900 -6.23414700 -1.50303200

H -4.17718800 -6.04152400 -2.66577200

C -6.41269700 -2.20872700 -0.70464600

C -6.50490000 -2.22017000 0.84476400

C -6.91015700 -0.83533200 -1.20438500

C -7.37523300 -3.31195600 -1.18678800

H -6.10105200 -3.15270900 1.25667600

H -5.97261100 -1.38605400 1.30860000

H -7.55545400 -2.14283400 1.14754800

H -6.90929600 -0.77492200 -2.29656900

H -7.93621500 -0.65917700 -0.85756300

H -6.28127100 -0.02302000 -0.82383100

H -8.34607200 -3.15864700 -0.70050000

H -7.54893500 -3.29827200 -2.26275600

H -7.01650000 -4.30827900 -0.90917700

C 3.39338600 -4.34751600 -0.18947800

C 3.07559200 -5.51341100 0.77356300

C 2.22939100 -4.25968200 -1.19587700

C 4.67268800 -4.66888700 -0.99053000

H 3.87304900 -5.65694800 1.50603700

H 2.14056700 -5.32402300 1.31320100

H 2.95798400 -6.44547700 0.20691100

H 2.40297000 -3.50048000 -1.96711300

H 2.12298200 -5.22463300 -1.70291600

H 1.27929500 -4.03815700 -0.69884100

H 4.47135300 -5.49173100 -1.68763600

H 4.99790100 -3.80423700 -1.57898800

H 5.49407800 -4.97847200 -0.34319500

C 5.03841600 -1.76559700 4.02269900

C 6.09460900 -0.64161400 3.99068800

C 3.98149700 -1.41798300 5.10135400

C 5.70474800 -3.06911000 4.50701500

H 6.86337300 -0.83415700 3.23465100

H 5.63806300 0.32852500 3.76858700

H 6.59181200 -0.56368800 4.96558100

H 3.22729200 -2.20921300 5.17243600

H 4.47282400 -1.33016000 6.07735700

H 3.46631600 -0.47313900 4.90905200

H 6.00602000 -2.92909200 5.55226500

H 5.01265700 -3.91576300 4.46939000

H 6.60154100 -3.33280500 3.94593800

C 6.57076100 2.55400300 -0.03134100

C 6.74578100 2.03346100 1.41674100

C 7.22213200 1.51250500 -0.96666400

C 7.33638200 3.89238200 -0.09213600

H 6.20329300 2.65613100 2.13798100

H 6.41128000 1.00043700 1.53492200

H 7.80872500 2.05586500 1.68189600

H 7.17690000 1.80371800 -2.01732300

H 8.27654400 1.37492800 -0.69504300

H 6.71675800 0.54472300 -0.87357500

H 8.35201700 3.73809800 0.29080900

H 7.41969000 4.30249800 -1.09686500

H 6.85268400 4.64693900 0.53987600

C 2.46085500 5.02452500 -2.10483300

C 2.69632100 4.83945300 -3.61880500

C 0.93605100 5.09035500 -1.88969000

C 3.04047500 6.37676100 -1.63575200

H 3.74292600 4.96678600 -3.89342100

H 2.37452500 3.84324800 -3.94492900

H 2.11310300 5.58199600 -4.17760400

H 0.67768600 5.19826600 -0.83150500

H 0.53825500 5.96234200 -2.42044800

H 0.42214700 4.20603100 -2.28334600

H 2.58007900 7.19618900 -2.20158900

H 2.82533800 6.54099700 -0.57258100

H 4.12112900 6.42437200 -1.77913600

H 0.97984200 -4.02896300 4.34264900

H -1.31664200 -3.61881800 0.74484300

C -3.05219300 4.28182500 1.27952900

C -2.83867900 4.93718200 2.66283800

C -1.78108900 4.54233600 0.44792300

C -4.21917800 4.99162300 0.56248700

H -3.71972000 4.82140600 3.29845400

H -1.98067800 4.48962600 3.17523500

H -2.64211800 6.00970900 2.54145200

H -1.87700100 4.17439400 -0.57890000

H -1.60684700 5.62221400 0.39527000

H -0.89581200 4.08333700 0.89962500

H -3.90453100 6.00078800 0.27007800

H -4.51741800 4.45868600 -0.34655400

H -5.09248000 5.09834900 1.20775100

C -4.94059900 0.47469300 4.15100500

C -4.56270400 -0.96795500 4.54016900

C -4.49952500 1.39994800 5.30804400

C -6.47744900 0.51870100 4.02545800

H -4.88482500 -1.69592800 3.78634300

H -3.48482200 -1.08719700 4.69578700

H -5.06224800 -1.22399500 5.48077500

H -4.76358100 2.44151300 5.10739100

H -4.99011000 1.09587600 6.24098800

H -3.41546100 1.34297700 5.45979200

H -6.92506900 0.02761800 4.89793800

H -6.85788200 1.54028700 3.99594700

H -6.82240500 -0.01383200 3.13191200

O 5.37588000 -3.89131500 1.87861200

O 5.29495000 4.47888700 -1.99687300

O -5.26389600 -3.97857600 -2.91730400

O -5.26667600 3.07917000 2.80429200

C -6.41727000 3.00368100 1.94231700

H -6.81625500 1.98683600 1.90194800

H -7.16521500 3.67675400 2.36816500

H -6.17154200 3.32161200 0.92592500

C -5.72785800 -3.18239500 -4.01551100

H -6.33107300 -3.84485300 -4.64118500

H -6.34368500 -2.34564200 -3.67584100

H -4.88974400 -2.79136900 -4.60084000

C 6.66296500 -3.47245700 1.40271700

H 7.05537900 -2.63636800 1.98986900

H 7.32638700 -4.33427900 1.50716000

H 6.61310200 -3.17069600 0.35111500

C 5.88532200 3.86468600 -3.15668300

H 5.36844200 2.93867600 -3.42503600

H 5.81569300 4.58489000 -3.97709700

H 6.94328900 3.65069200 -2.97611000

Ni 0.16244800 0.12458700 -0.89964000

C -1.99636300 0.61523700 -2.99296300

C -2.33080000 1.91181100 -2.56944200

C -3.64030100 2.38787500 -2.62155100

C -4.65749900 1.56365700 -3.11996100

C -4.33706800 0.28461400 -3.59373200

C -3.03009700 -0.17824700 -3.52419300

C -0.64711900 0.05059400 -2.82226700

C 0.55191800 0.82943600 -2.89235600

C 1.82305400 0.27052000 -2.73897100

H 0.47779400 1.91351600 -2.96047900

H -0.55977100 -1.00573400 -3.08018500

H -1.56225100 2.55521300 -2.15219200

H -2.80460000 -1.18425100 -3.86348000

H -3.85583000 3.38680600 -2.26191000

H -5.12782100 -0.33966100 -3.99470900

O -5.97817400 1.91477800 -3.18164900

C -6.37379300 3.11309900 -2.51810100

H -7.45919700 3.16881800 -2.62334800

H -6.11046800 3.07890800 -1.45379700

H -5.92008900 3.99897700 -2.97884000

H 1.94702600 -0.80453000 -2.66930900

H 2.68197200 0.88504400 -2.51388400

C 3.20925600 0.13752900 -4.90759300

C 4.25728500 -0.24211500 -4.07916600

C 4.47564000 -1.73852500 -3.83216200

C 5.94323900 -2.15161600 -3.96875400

O 4.99560100 0.56437200 -3.41315800

H 3.04306400 1.18948500 -5.13061100

H 2.65163900 -0.59111400 -5.48897700

H 4.14358300 -1.95078400 -2.80379400

H 3.84578600 -2.33983800 -4.49928300

H 6.08634200 -3.21246200 -3.73137600

H 6.30626600 -1.98372800 -4.99106000

H 6.56846700 -1.56130300 -3.29072300

**TS3**

**Imaginary frequency: -93.27 cm^-1^**

C 1.32853000 -2.49041400 4.31150400

C 1.26377800 -1.11224500 4.11098700

C 0.47149400 -0.55672700 3.09682300

C -0.30030800 -1.42125700 2.28428000

C -0.24886100 -2.80336300 2.51206400

C 0.56478100 -3.33923000 3.51085300

C 0.44765600 0.93759700 2.98591400

C -0.13168500 1.64496600 4.04823100

C -0.17524100 3.03818400 4.05889300

C 0.39738300 3.75219600 3.00722800

C 0.99614400 3.06648700 1.95024600

C 1.02289900 1.66559200 1.91687200

P -1.24144900 -0.73874900 0.85960200

P 1.71744600 0.79079600 0.45261400

C -2.30616000 -2.08554900 0.23269500

C -2.35771000 0.50191600 1.57589500

C 2.96226500 -0.32493500 1.16695900

C 2.69747700 2.04081900 -0.45092200

C 3.07194800 -1.63236500 0.69767100

C 4.08454200 -2.48631100 1.15561100

C 5.03739300 -1.92912300 2.04002600

C 4.83838600 -0.68146000 2.68300500

C 3.79647400 0.11255300 2.19945300

C 2.02659000 2.93505700 -1.29567900

C 2.71781600 3.78539900 -2.15865100

C 4.13781400 3.69128600 -2.16713600

C 4.84468200 2.93480800 -1.20876900

C 4.08539300 2.07751600 -0.39747800

C -1.69820500 -3.12500500 -0.48673700

C -2.44980000 -4.10223200 -1.13888000

C -3.86539200 -3.97447400 -1.09268600

C -4.50880200 -3.02412500 -0.27138800

C -3.69163400 -2.06118700 0.34698400

C -2.44237600 1.75500600 0.96228500

C -3.32895600 2.73034900 1.42135900

C -4.16712800 2.37931600 2.51053500

C -3.97551700 1.19965400 3.26557800

C -3.08411500 0.25429300 2.73860100

H 1.96598000 -2.89483500 5.09228700

H -0.85217200 -3.47035100 1.90685500

H 0.59175700 -4.41407600 3.66459300

H 0.38739400 4.83815700 3.00755300

H 2.35152900 -1.98093400 -0.03062100

H 3.62042000 1.09129500 2.62496700

H 0.94422100 2.93196500 -1.29269700

H 4.60153000 1.41105000 0.27790400

H -0.61719300 -3.14998600 -0.54239600

H -4.15409500 -1.26842700 0.91849000

H -1.80102800 1.95373200 0.11407600

H -2.94971000 -0.69390700 3.24668600

H -0.56683300 1.08446900 4.86930700

H 1.84881400 -0.44629400 4.73799900

C 1.95005200 4.81023400 -3.02087800

C 2.10244400 4.53777300 -4.53241200

C 0.44187500 4.78719700 -2.70508300

C 2.46536300 6.22927600 -2.69286000

H 3.10990200 4.75228300 -4.88959900

H 1.85950100 3.49594200 -4.77291900

H 1.40975000 5.17917000 -5.09086900

H 0.24309400 4.97959600 -1.64616100

H -0.05561000 5.57283400 -3.28412200

H -0.02526800 3.83370300 -2.97785200

H 1.92116500 6.97202000 -3.28916800

H 2.30488700 6.46451500 -1.63368900

H 3.53090600 6.32998000 -2.91071200

C 6.36885200 2.96735800 -0.93493000

C 6.57916300 3.09985400 0.59635300

C 7.01711900 1.64514000 -1.39678800

C 7.12179900 4.15464600 -1.56696700

H 6.09224800 4.00259100 0.98380600

H 6.19373000 2.24137000 1.15311000

H 7.65116300 3.17280400 0.81251100

H 6.93983500 1.51648300 -2.48138300

H 8.08198800 1.63616000 -1.13279400

H 6.54372700 0.77986800 -0.91940200

H 8.13998600 4.17301600 -1.16016400

H 7.20862000 4.08551100 -2.65146900

H 6.64634400 5.10950600 -1.32070700

C -3.32177700 4.14766500 0.80921100

C -3.06233800 5.17670300 1.93214200

C -2.18379900 4.29924600 -0.21908900

C -4.64357900 4.48891200 0.08905200

H -3.84770100 5.14749600 2.69074900

H -2.10127800 4.98301300 2.42133000

H -3.03059200 6.18923200 1.51058600

H -2.32534500 3.65294900 -1.09205900

H -2.16339600 5.33405300 -0.57734900

H -1.20580400 4.07878100 0.22114400

H -4.51955500 5.41763100 -0.48198900

H -4.93311300 3.69481100 -0.60501100

H -5.46113900 4.64200000 0.79521700

C -4.62202700 0.87529500 4.63402600

C -5.61646500 -0.29334100 4.47729400

C -3.49622900 0.44180200 5.60703500

C -5.33399200 2.05617900 5.32343700

H -6.42456600 -0.03975100 3.78285700

H -5.11810100 -1.19294600 4.10101400

H -6.06742100 -0.53912800 5.44686500

H -2.77204600 1.25226000 5.74759400

H -3.93015200 0.20193100 6.58480600

H -2.95309200 -0.44343500 5.26503600

H -5.58565200 1.75338100 6.34713700

H -4.68755100 2.93719300 5.38491200

H -6.26360100 2.34654900 4.83343000

C -6.02521800 -2.95536800 0.03647100

C -6.20910800 -2.72098000 1.55742900

C -6.65611100 -1.76123700 -0.70896400

C -6.81538000 -4.24022500 -0.28766100

H -5.72338600 -3.51112800 2.14282800

H -5.81177600 -1.75895100 1.88657500

H -7.27786200 -2.72720800 1.80059700

H -6.52442600 -1.84119700 -1.79168100

H -7.73241600 -1.71833400 -0.49897100

H -6.21719000 -0.81024800 -0.39507200

H -7.81681300 -4.14561400 0.14924300

H -6.94552400 -4.41305000 -1.35589200

H -6.34319200 -5.12660500 0.14870000

C -1.74942000 -5.30607000 -1.80684100

C -1.97771700 -5.35685300 -3.33259300

C -0.22710300 -5.26347000 -1.57727100

C -2.26929300 -6.60992300 -1.16023700

H -3.00011500 -5.63717000 -3.58640200

H -1.75454200 -4.39068600 -3.80025000

H -1.31062500 -6.10558200 -3.77724300

H 0.02600000 -5.24233000 -0.51290300

H 0.22459700 -6.16381200 -2.00791300

H 0.24084500 -4.39886900 -2.05999600

H -1.76979400 -7.47605900 -1.61191200

H -2.05529300 -6.62075600 -0.08454700

H -3.34626800 -6.72494700 -1.29889200

H -0.64756900 3.55876100 4.88687500

H 1.45356500 3.63119500 1.14578000

C 4.05591500 -3.98969200 0.80001900

C 4.08588400 -4.80917300 2.10985100

C 2.74544700 -4.35144800 0.07463500

C 5.22476700 -4.42492200 -0.10837000

H 5.00318300 -4.63131000 2.67491400

H 3.23119900 -4.55112500 2.74578400

H 4.02660600 -5.88022800 1.87985100

H 2.67295100 -3.87791200 -0.90832000

H 2.71060100 -5.43565900 -0.07689400

H 1.86689900 -4.06733800 0.66296400

H 5.01961800 -5.42251400 -0.51566600

H 5.35198400 -3.73626700 -0.94996400

H 6.16768400 -4.48578500 0.43818800

C 5.60804000 -0.26251500 3.95611600

C 5.05745600 1.05821100 4.52837300

C 5.38643300 -1.35883700 5.02307000

C 7.12199700 -0.06389700 3.73858200

H 5.21970200 1.90236500 3.84796300

H 3.98674900 0.99835500 4.75280200

H 5.57922700 1.28427300 5.46456000

H 5.78090100 -2.32271000 4.69145800

H 5.89338100 -1.08380300 5.95623600

H 4.31856500 -1.47998500 5.23943000

H 7.55927800 0.39280700 4.63467300

H 7.63981800 -1.00845300 3.56827300

H 7.32073700 0.60608900 2.89398100

O -5.20487700 3.23882600 2.81705000

O -4.60711800 -4.84477200 -1.86986200

O 4.81855100 4.37884800 -3.15424400

O 6.19316300 -2.64285000 2.31363200

C 7.25458000 -2.32328200 1.39450900

H 7.53266400 -1.26815700 1.46198100

H 8.10508900 -2.94731600 1.67908600

H 6.96489100 -2.54127200 0.36364200

C 5.26805800 3.53301000 -4.22243600

H 5.72449400 4.19182700 -4.96507200

H 6.00878600 2.80613300 -3.87764100

H 4.42981500 2.99426300 -4.67640600

C -6.45934400 2.78067500 2.28297300

H -6.84811300 1.93269100 2.85704600

H -7.15544100 3.61917200 2.36164600

H -6.34925400 2.47905000 1.23798200

C -5.10054800 -4.26338400 -3.08458900

H -4.27833700 -3.90424900 -3.70996600

H -5.63799500 -5.05825100 -3.60775500

H -5.78183800 -3.43163600 -2.88787900

Ni 0.12299800 -0.08491500 -0.71536000

C 2.08006700 -0.67881300 -2.97723800

C 2.16365500 -2.07665200 -3.09608800

C 3.37939500 -2.72857400 -3.30595600

C 4.56211900 -1.98462000 -3.38289300

C 4.50182400 -0.58699600 -3.28018600

C 3.28315000 0.04938200 -3.09073200

C 0.82013200 0.03032900 -2.70010200

C -0.46397600 -0.60461100 -2.71856800

C -1.64703200 0.07064600 -2.41895400

H -0.52655000 -1.68429500 -2.82773000

H 0.84652300 1.10570700 -2.86932100

H 1.26645700 -2.68244700 -3.02781300

H 3.25559100 1.13138200 -3.02550100

H 3.39244400 -3.80825500 -3.39687300

H 5.42079200 -0.01523200 -3.35982100

O 5.80660000 -2.52548200 -3.55870300

C 5.88684400 -3.90849500 -3.90166000

H 6.94670500 -4.11299500 -4.06662600

H 5.51783900 -4.54896500 -3.09333200

H 5.32550200 -4.11914700 -4.82029100

H -1.65940700 1.15028500 -2.30815800

H -2.55209700 -0.46345200 -2.16727900

C -3.96943100 2.37944700 -3.60329000

C -4.01170900 0.86611700 -3.73222700

C -3.12678000 0.24144100 -4.58982800

C -3.12924100 -1.23762800 -4.83622300

O -4.84555800 0.23557100 -2.96213500

H -3.21126600 2.84003700 -4.24573700

H -4.94629700 2.81312300 -3.85510700

H -3.75960100 2.65750800 -2.56257500

H -2.48381800 0.86164300 -5.21075500

H -3.72972500 -1.74339400 -4.07307800

H -3.55224100 -1.50840400 -5.81768900

H -2.11504100 -1.66521400 -4.80844600

C -8.15319800 1.02140600 -2.68891600

C -7.39473800 2.14768100 -1.98423200

H -7.47869100 0.46301900 -3.34644900

H -8.57557500 0.32438500 -1.95466600

H -8.97445800 1.42168000 -3.29672000

C -8.28749600 2.92184500 -1.02175300

H -7.02911800 2.84778800 -2.75501300

H -7.73636500 3.74478900 -0.55346800

H -9.15248600 3.34329100 -1.54730500

H -8.65599600 2.25943900 -0.22805100

O -6.28697100 1.63973800 -1.24318900

H -5.70069400 1.13080800 -1.88490000

**TS3′**

**Imaginary frequency: -151.86 cm^-1^**

C -1.52752100 3.20746200 3.86619100

C -1.40504200 1.81877000 3.88872000

C -0.57990400 1.14264200 2.97946800

C 0.16352300 1.89841300 2.04256900

C 0.05538800 3.29586400 2.04661100

C -0.78955000 3.94939100 2.94423000

C -0.48904800 -0.34737600 3.11195300

C 0.10706300 -0.84335300 4.27977100

C 0.20153300 -2.21231700 4.52559900

C -0.33330700 -3.11411000 3.60686200

C -0.94116800 -2.63947500 2.44442900

C -1.01893400 -1.26649100 2.17411900

P 1.14349300 1.03533100 0.75107800

P -1.72307700 -0.68067200 0.57505900

C 2.14927000 2.29735600 -0.10359200

C 2.31191600 -0.01981100 1.65836300

C -3.02703800 0.47864700 1.08610100

C -2.63040100 -2.10782600 -0.11735800

C -3.17890800 1.69052300 0.41554500

C -4.22984500 2.56497600 0.72396000

C -5.17525600 2.11571400 1.67546400

C -4.93963000 0.99306700 2.50852900

C -3.85983100 0.17629900 2.16697500

C -1.90524000 -3.09295100 -0.80111700

C -2.54233400 -4.09967900 -1.52670200

C -3.96476000 -4.07520300 -1.56349900

C -4.72107800 -3.21224000 -0.74303200

C -4.01590200 -2.20130900 -0.07206600

C 1.49868500 3.17487700 -0.98475500

C 2.21022200 3.98563100 -1.86804900

C 3.62568700 3.84954300 -1.87920800

C 4.31525300 3.10304000 -0.90253800

C 3.53783500 2.29936500 -0.05024000

C 2.43188700 -1.36288700 1.28771100

C 3.36003900 -2.20640000 1.90157500

C 4.20906100 -1.62794100 2.88001200

C 3.98948900 -0.33373800 3.40398200

C 3.04959900 0.46410300 2.73619900

H -2.18906800 3.70416300 4.56987200

H 0.63866700 3.88218600 1.34560400

H -0.86150600 5.03296200 2.92491500

H -0.28385700 -4.18331700 3.79039500

H -2.45923600 1.94818000 -0.35041300

H -3.65220700 -0.71404100 2.74491100

H -0.82434300 -3.03814300 -0.78789100

H -4.57388100 -1.46094900 0.48259400

H 0.41603200 3.19048400 -0.99306200

H 4.03716100 1.64051500 0.64694300

H 1.78227600 -1.73874000 0.50836600

H 2.88965800 1.48583100 3.06199100

H 0.51197800 -0.13568600 4.99657400

H -1.96812500 1.23788500 4.61286300

C -1.71446900 -5.20797500 -2.21208500

C -1.85987400 -5.18609200 -3.74855300

C -0.21364800 -5.05880200 -1.89695700

C -2.16441600 -6.58140800 -1.66617400

H -2.85118500 -5.50334300 -4.07274900

H -1.66295700 -4.18508700 -4.15037600

H -1.13012700 -5.87342500 -4.19364800

H -0.01811100 -5.07575900 -0.82035400

H 0.33083300 -5.89640800 -2.34625800

H 0.20675600 -4.13498600 -2.31097200

H -1.57683200 -7.38095100 -2.13398400

H -2.00765600 -6.63782700 -0.58215100

H -3.22079100 -6.76843400 -1.87191200

C -6.24572900 -3.27474900 -0.47892400

C -6.46981300 -3.19081000 1.05372100

C -6.94419000 -2.06590500 -1.13644000

C -6.93802000 -4.57445400 -0.93422600

H -5.95402400 -4.00813800 1.57153200

H -6.12305600 -2.24642000 1.48206600

H -7.54087800 -3.27341200 1.27125200

H -6.84848600 -2.09153700 -2.22684800

H -8.01385000 -2.07084100 -0.89245300

H -6.52224700 -1.11831200 -0.78296500

H -7.95969300 -4.57795600 -0.53586000

H -7.01386700 -4.66853600 -2.01774000

H -6.42555400 -5.46094000 -0.54715900

C 3.39028200 -3.71526600 1.57436700

C 3.22639700 -4.51929100 2.88384300

C 2.21353900 -4.10539200 0.65888900

C 4.69370700 -4.14123300 0.86543000

H 4.04223700 -4.32142000 3.58202400

H 2.28042400 -4.26807600 3.37623900

H 3.21806800 -5.59373500 2.66180600

H 2.28302100 -3.64058100 -0.32894000

H 2.22363900 -5.19053100 0.51037800

H 1.24916800 -3.83604600 1.10086400

H 4.58384300 -5.16314400 0.48131300

H 4.92787500 -3.48552600 0.02216800

H 5.54715700 -4.13556700 1.54580600

C 4.65273900 0.26900400 4.66543500

C 5.58120500 1.43228500 4.25969800

C 3.52727700 0.82142200 5.57686500

C 5.44589800 -0.72431800 5.53709100

H 6.38709300 1.08774900 3.60260700

H 5.03044700 2.21971000 3.73438800

H 6.03887000 1.87969900 5.15088900

H 2.84794400 0.01736400 5.88316700

H 3.96777200 1.25622900 6.48168500

H 2.93258500 1.60237800 5.09505600

H 5.71263100 -0.21918000 6.47349000

H 4.84987000 -1.60622600 5.79115000

H 6.37415700 -1.05950100 5.07345600

C 5.84382500 3.08160700 -0.67052400

C 6.10946200 3.23172400 0.84877200

C 6.41235200 1.72226300 -1.12572200

C 6.62761300 4.22312400 -1.34871600

H 5.68626100 4.16748000 1.23370000

H 5.69648400 2.40618300 1.43205300

H 7.19025900 3.24798100 1.03097000

H 6.21393900 1.53045500 -2.18478900

H 7.49804800 1.70311900 -0.97139200

H 5.97703100 0.89470200 -0.55728800

H 7.65358100 4.21165200 -0.96146900

H 6.69119100 4.12336500 -2.43225600

H 6.19499100 5.20227000 -1.11864400

C 1.47368200 5.01935900 -2.74712800

C 1.58659100 4.70175700 -4.25339100

C -0.02713200 5.07205700 -2.40535900

C 2.05403700 6.42361500 -2.46630700

H 2.59518900 4.86398500 -4.63409200

H 1.29742700 3.66501600 -4.46283800

H 0.91086700 5.35701400 -4.81679200

H -0.19864700 5.29603300 -1.34760000

H -0.50041000 5.86504200 -2.99473700

H -0.54005700 4.13499600 -2.64571700

H 1.52970000 7.17258200 -3.07269100

H 1.92265200 6.69281300 -1.41120000

H 3.11838000 6.47418200 -2.70512400

H 0.68327000 -2.56789700 5.43175500

H -1.36402400 -3.34887800 1.74198400

C -4.25184900 3.99380100 0.13625200

C -4.33969500 5.00793500 1.29890400

C -2.94256800 4.28989800 -0.62043400

C -5.41788200 4.23104900 -0.84602900

H -5.26090000 4.88424800 1.87205500

H -3.48899900 4.88863600 1.97971200

H -4.31658800 6.03072500 0.90279100

H -2.83668300 3.67311700 -1.51696100

H -2.94498200 5.33801800 -0.93847200

H -2.06419500 4.13486800 0.01463800

H -5.24253000 5.15837300 -1.40508300

H -5.50138300 3.41217100 -1.56796700

H -6.37358900 4.34002700 -0.32998200

C -5.71427400 0.74505600 3.82277200

C -5.11781100 -0.44269100 4.60310100

C -5.56162600 2.00377500 4.70685000

C -7.21324200 0.44384700 3.61959700

H -5.23180800 -1.39029500 4.06380000

H -4.05475700 -0.30007300 4.82624100

H -5.64581300 -0.54096900 5.55774700

H -5.99199800 2.88470800 4.22392300

H -6.07333000 1.85442300 5.66564300

H -4.50437800 2.20672000 4.91413400

H -7.64752000 0.10994600 4.56969600

H -7.76950500 1.32543500 3.29910600

H -7.36480200 -0.35631800 2.88570300

O 5.28382700 -2.37852100 3.31651500

O 4.31688200 4.48201700 -2.89663100

O -4.59778400 -4.93895400 -2.43770900

O -6.36117000 2.81751700 1.82234700

C -7.39561600 2.31825100 0.95382300

H -7.63745100 1.27728800 1.18399900

H -8.27194100 2.94719800 1.12764600

H -7.09709000 2.38167500 -0.09543200

C -5.06477200 -4.29086700 -3.62976600

H -5.47019600 -5.07861900 -4.26929200

H -5.84960600 -3.56101200 -3.41170800

H -4.24463100 -3.78356000 -4.14836200

C 6.50684300 -1.99832200 2.66224000

H 6.84567400 -1.01281900 2.99971700

H 7.25069700 -2.75085200 2.93487800

H 6.37669200 -1.97654900 1.57730300

C 4.70416800 3.58998700 -3.95221900

H 3.84182300 3.03055700 -4.32985900

H 5.11483900 4.21503900 -4.74891500

H 5.46474400 2.87811100 -3.61978200

Ni -0.15772300 0.07402800 -0.71482200

C -2.08373300 0.18786000 -3.03830000

C -2.23254600 1.54559000 -3.37001900

C -3.47369200 2.09437800 -3.69560500

C -4.61697700 1.28783200 -3.67654300

C -4.49114500 -0.07296700 -3.36055800

C -3.24750900 -0.60940100 -3.05677900

C -0.79721200 -0.40538900 -2.63315300

C 0.45762200 0.27599700 -2.76821500

C 1.67282000 -0.27972100 -2.36452900

H 0.47252500 1.31420800 -3.08966100

H -0.77384800 -1.49433800 -2.62726400

H -1.36883900 2.20204200 -3.37848300

H -3.16985900 -1.66590400 -2.82493600

H -3.53712600 3.14604800 -3.94905200

H -5.37888700 -0.69739200 -3.36651300

O -5.88205400 1.73149900 -3.95163900

C -6.02201600 3.04019100 -4.50345800

H -7.08640000 3.16134500 -4.71515000

H -5.70502800 3.81500400 -3.79705100

H -5.44991700 3.13806400 -5.43433500

H 1.72503700 -1.30383800 -2.01436600

H 2.54786900 0.33830400 -2.21890600

C 3.07591000 -1.07181400 -4.36641200

C 3.98522200 -1.61734800 -3.47776900

C 3.69983600 -3.00940200 -2.92202900

C 4.85353300 -4.00136400 -3.09793100

O 5.01406800 -0.98410500 -3.02166900

H 3.24949700 -0.08489500 -4.79033100

H 2.27384900 -1.66727500 -4.79022000

H 3.49974700 -2.89395200 -1.84759200

H 2.78599500 -3.41448200 -3.37273800

H 4.57879100 -4.98898100 -2.70778300

H 5.11648900 -4.11865000 -4.15700000

H 5.74202000 -3.66399000 -2.56048100

C 8.79734300 -1.66459200 -0.20147500

C 7.88802100 -1.49053400 -1.41192800

H 8.51715100 -0.96762500 0.59690100

H 8.71856000 -2.68649200 0.19041500

H 9.84396200 -1.47778200 -0.46872600

C 8.29174800 -2.40106100 -2.57257900

H 7.96645100 -0.44842100 -1.75737800

H 7.60670600 -2.26654300 -3.41688200

H 9.30773600 -2.16914400 -2.91645000

H 8.26298300 -3.45450600 -2.26696200

O 6.54963000 -1.74711200 -0.98562900

H 5.93383700 -1.51369300 -1.74205500

**TS4**

**Imaginary frequency: -51.99 cm^-1^**

C -0.23712900 2.77277900 3.76690500

C -0.28477700 1.38499700 3.64437600

C 0.37739300 0.71908700 2.60364300

C 1.13146500 1.48173800 1.68127500

C 1.18975100 2.87494700 1.82411300

C 0.50716300 3.52072400 2.85486100

C 0.29720800 -0.77672400 2.58364700

C 0.90064300 -1.45451900 3.65234300

C 0.86751100 -2.84447700 3.74806000

C 0.19067600 -3.58300300 2.77888400

C -0.43632600 -2.92553200 1.72049100

C -0.38653900 -1.52989000 1.59861500

P 1.93813600 0.66717400 0.24784000

P -1.11422800 -0.70444100 0.11752600

C 3.04688100 1.91186300 -0.50265300

C 3.02109500 -0.60108900 0.96772500

C -2.19596000 0.57761500 0.82546800

C -2.27243600 -1.92620100 -0.59809600

C -2.20861500 1.86293000 0.28768200

C -3.11336700 2.83463700 0.73743600

C -4.05644000 2.42537400 1.70882400

C -3.93824600 1.20120900 2.41465100

C -3.00898600 0.28189800 1.92338300

C -1.75481900 -3.00682000 -1.32386600

C -2.58829900 -3.93778600 -1.94530800

C -3.99252500 -3.72881800 -1.84712000

C -4.54563500 -2.72777400 -1.02108500

C -3.64873000 -1.81107100 -0.45137700

C 2.47488100 2.86464300 -1.35822000

C 3.25815700 3.69376100 -2.15945400

C 4.66830900 3.51836600 -2.09283800

C 5.27799400 2.69814100 -1.12173300

C 4.42928500 1.86424900 -0.37177800

C 3.01194700 -1.88191400 0.40785200

C 3.87387200 -2.88057200 0.86596000

C 4.79476100 -2.52089000 1.88297700

C 4.69900800 -1.30370600 2.59646300

C 3.81872300 -0.34178600 2.08096300

H -0.77369900 3.26370200 4.57340600

H 1.77942500 3.46185600 1.12854800

H 0.56584100 4.60126000 2.94603100

H 0.14058800 -4.66587300 2.84440700

H -1.49612000 2.10113000 -0.48898900

H -2.90608700 -0.68544300 2.39587500

H -0.68029600 -3.10402600 -1.40803400

H -4.04624300 -0.98570100 0.11958600

H 1.39513100 2.91450500 -1.42221800

H 4.86841600 1.14957100 0.31092900

H 2.31671600 -2.08813900 -0.39506100

H 3.74939800 0.62848400 2.55973000

H 1.41732700 -0.87366200 4.40917800

H -0.85728900 0.79881000 4.35668000

C -1.97920900 -5.17311500 -2.64456200

C -2.28486100 -5.20896500 -4.15693600

C -0.44599600 -5.20253400 -2.49377200

C -2.52899000 -6.45108500 -1.97228100

H -3.33285500 -5.42796600 -4.36155800

H -2.02397300 -4.25823900 -4.63685500

H -1.68541500 -5.99542500 -4.63134700

H -0.13790000 -5.20490400 -1.44396500

H -0.06048500 -6.11921900 -2.95314200

H 0.03896100 -4.35629200 -2.99403200

H -2.09662700 -7.33994000 -2.44824600

H -2.26134500 -6.47470100 -0.90887200

H -3.61623100 -6.51272000 -2.05530300

C -6.03646600 -2.54619000 -0.63796600

C -6.12164700 -2.31894600 0.89514300

C -6.60669100 -1.29501200 -1.34003600

C -6.94070200 -3.76017600 -0.92966100

H -5.66548300 -3.15199100 1.44313700

H -5.63395100 -1.39488000 1.21544800

H -7.17354100 -2.25089100 1.19529200

H -6.60370500 -1.40318500 -2.42855300

H -7.64214800 -1.12345600 -1.01992400

H -6.02389500 -0.40128200 -1.09106900

H -7.91461600 -3.58567300 -0.45688200

H -7.12359600 -3.92205800 -1.99175300

H -6.52539600 -4.68128200 -0.50828100

C 3.75127500 -4.32359600 0.33210900

C 3.58152500 -5.29149200 1.52427500

C 2.50345000 -4.47445400 -0.55987500

C 4.96817800 -4.73946900 -0.51909600

H 4.45243400 -5.28171300 2.18283700

H 2.69810500 -5.02299100 2.11544100

H 3.44226600 -6.31468300 1.15401500

H 2.58630400 -3.88731500 -1.47961900

H 2.40003400 -5.52634300 -0.84837000

H 1.59028300 -4.18213500 -0.03066300

H 4.79556300 -5.73962700 -0.93647000

H 5.10046500 -4.04919800 -1.35854500

H 5.88959000 -4.77931600 0.06340300

C 5.43009500 -0.95492600 3.91633200

C 6.43050200 0.19362200 3.67312500

C 4.36709800 -0.47757500 4.93805200

C 6.16632100 -2.12489400 4.59853800

H 7.18550000 -0.08305300 2.92904000

H 5.92230300 1.09706900 3.32067800

H 6.95096300 0.44525000 4.60555800

H 3.64825300 -1.27683400 5.15040100

H 4.85995700 -0.20568100 5.87883100

H 3.80976500 0.39840700 4.59490000

H 6.47981400 -1.79413300 5.59618500

H 5.51567300 -2.99543400 4.72381000

H 7.06421500 -2.44027500 4.06608000

C 6.78648600 2.64199200 -0.77591500

C 6.92591200 2.76074500 0.76260400

C 7.37348600 1.28401900 -1.21529200

C 7.64438000 3.78048300 -1.36276400

H 6.49322400 3.70110800 1.12466400

H 6.44235900 1.93827700 1.29298500

H 7.98694100 2.74716100 1.03752300

H 7.31130200 1.15349100 -2.30017000

H 8.42990300 1.21890200 -0.92641200

H 6.84333200 0.44953200 -0.74369900

H 8.63783400 3.73210800 -0.90071100

H 7.78732500 3.70664400 -2.44098500

H 7.21952300 4.76376300 -1.13791200

C 2.60016600 4.78299900 -3.03324600

C 2.82970800 4.55142400 -4.54205100

C 1.07717700 4.83142100 -2.80863500

C 3.16853400 6.16122400 -2.62752600

H 3.86325600 4.73757500 -4.83443100

H 2.56300600 3.52807700 -4.83152600

H 2.19502900 5.23695900 -5.11682900

H 0.82498900 5.00257300 -1.75782900

H 0.65587800 5.65823900 -3.39069500

H 0.58097700 3.91127700 -3.13894700

H 2.70216800 6.95058000 -3.22992700

H 2.95695000 6.37280200 -1.57234300

H 4.24924000 6.20916600 -2.77887800

H 1.36117100 -3.34252200 4.57744700

H -0.97616900 -3.51048300 0.98547400

C -2.96377300 4.30709500 0.28860000

C -2.75313200 5.17590300 1.54961400

C -1.72001200 4.48486500 -0.60322200

C -4.17119200 4.85244800 -0.50129700

H -3.61685500 5.12587700 2.21698200

H -1.86811200 4.84630900 2.10411800

H -2.60390500 6.22369400 1.26064900

H -1.81747500 3.95595700 -1.55741000

H -1.59359000 5.54894100 -0.82879300

H -0.80817800 4.13932300 -0.10579500

H -3.90667500 5.82143500 -0.94171300

H -4.45456000 4.18156300 -1.31938700

H -5.04074000 5.01296700 0.13756000

C -4.66074000 0.93148500 3.75480800

C -4.20720300 -0.40713400 4.36952300

C -4.26089200 2.05473400 4.73859500

C -6.19833600 0.87767700 3.64524700

H -4.49667100 -1.26440700 3.75035200

H -3.12355400 -0.44576200 4.52640400

H -4.68683900 -0.52899800 5.34669300

H -4.58177900 3.03440200 4.37517600

H -4.72798600 1.88222400 5.71609100

H -3.17422300 2.07881500 4.88057500

H -6.61480700 0.51751700 4.59368000

H -6.63174700 1.85948300 3.45158900

H -6.52015500 0.18641400 2.85805800

O 5.80893900 -3.41425700 2.17221400

O 5.44058500 4.18906400 -3.02263900

O -4.81549700 -4.56410400 -2.58061100

O -5.11521700 3.26679700 2.00965700

C -6.26775100 2.99791200 1.18957600

H -6.61150400 1.96787000 1.31447800

H -7.04717600 3.68931800 1.51838300

H -6.04826300 3.16550900 0.13207400

C -5.33483300 -3.96705300 -3.77596900

H -5.89778800 -4.75030900 -4.28948500

H -6.00162900 -3.13004800 -3.55290300

H -4.52663400 -3.61477800 -4.42417200

C 7.05696400 -3.03948400 1.57205400

H 7.43535000 -2.09920800 1.98581300

H 7.76059400 -3.84534500 1.79391800

H 6.95257200 -2.92903800 0.48694500

C 5.88526500 3.34600400 -4.09575600

H 5.03566100 2.88411000 -4.60716600

H 6.42529500 3.99267200 -4.79162600

H 6.55423900 2.55787500 -3.73837300

Ni 0.48077500 -0.05999000 -1.20395700

C -1.78151500 0.03118800 -3.42366800

C -2.16051600 1.35815700 -3.16225400

C -3.48675700 1.77482200 -3.25579200

C -4.47503800 0.85604200 -3.63398400

C -4.11049500 -0.46087000 -3.94615800

C -2.78743600 -0.86397200 -3.83376600

C -0.41363300 -0.45114000 -3.20145200

C 0.75161500 0.34796800 -3.35080900

C 2.00442600 -0.10237100 -2.92876900

H 0.64031600 1.40190300 -3.60248300

H -0.28208900 -1.53161100 -3.23017400

H -1.41268200 2.07336100 -2.83393000

H -2.52530400 -1.89580600 -4.04460300

H -3.73925300 2.80076400 -3.01688100

H -4.88142000 -1.15803200 -4.25445400

O -5.80705400 1.14652600 -3.71923500

C -6.24383000 2.40429800 -3.20741300

H -7.33206800 2.40069100 -3.29541200

H -5.96172700 2.51900200 -2.15382600

H -5.83647500 3.23979900 -3.78932500

H 2.19361600 -1.15565900 -2.74865300

H 2.84647700 0.57307900 -2.88908600

C 5.27917800 -1.11030100 -3.16004300

C 4.15783300 -1.77159300 -3.96864200

C 3.61909600 -1.16045600 -5.08966500

C 2.47680600 -1.81468700 -5.81950600

O 3.74570300 -2.90602600 -3.48559800

H 4.97172500 -1.15552100 -2.10524100

H 5.41244300 -0.05122600 -3.39921700

H 2.18050600 -2.74051300 -5.31716600

H 1.58954300 -1.15757500 -5.87258600

H 2.72492400 -2.06412200 -6.86631500

C 4.08478700 0.13498300 -5.69897800

H 4.93934900 0.58293000 -5.18499500

H 4.38285400 0.00876300 -6.75506900

H 3.28138900 0.89561400 -5.71432300

C 6.61835800 -1.84159200 -3.32147600

H 7.00077500 -1.73394000 -4.34492100

H 7.38174800 -1.45495200 -2.63462600

H 6.49708800 -2.91286100 -3.12384900

**TS4′**

**Imaginary frequency: -139.83 cm^-1^**

C 0.38555500 -2.60792300 4.39616500

C 0.56422600 -1.24509700 4.16292500

C -0.06112700 -0.59718900 3.08822400

C -0.90681000 -1.35179900 2.24042900

C -1.09559800 -2.71691800 2.49496600

C -0.45395100 -3.34577600 3.56206600

C 0.14542700 0.88264300 2.96854100

C -0.36352700 1.67004700 4.01156000

C -0.20929500 3.05492900 4.02554300

C 0.49406900 3.67712200 2.99538700

C 1.02575300 2.90978900 1.95927000

C 0.85683200 1.51849400 1.92236200

P -1.65387000 -0.57582400 0.75181200

P 1.48598000 0.55130000 0.48422400

C -2.88509200 -1.76306600 0.10283100

C -2.61736600 0.82220100 1.40869600

C 2.50323800 -0.74302500 1.26496200

C 2.68451400 1.65197000 -0.34940300

C 2.37695700 -2.07008900 0.85655300

C 3.19756100 -3.07793100 1.38156900

C 4.21261600 -2.66826300 2.27772700

C 4.23727500 -1.37567200 2.85943300

C 3.37713100 -0.42363200 2.30781400

C 2.19465000 2.67211200 -1.17575500

C 3.04825200 3.47002500 -1.93834700

C 4.44144600 3.18786400 -1.86998200

C 4.97677100 2.27298600 -0.93869600

C 4.05791600 1.48382000 -0.23006100

C -2.40595600 -2.80869800 -0.69907500

C -3.26098000 -3.60223200 -1.46072000

C -4.64838800 -3.29437400 -1.40944500

C -5.17975300 -2.38127700 -0.47553200

C -4.25786600 -1.59107000 0.23536400

C -2.52218600 2.06763500 0.78088600

C -3.28707600 3.15671800 1.20871500

C -4.19503500 2.93026400 2.27483900

C -4.18506900 1.74261700 3.04141000

C -3.40445400 0.68661900 2.55085900

H 0.89429300 -3.08609300 5.22804500

H -1.75496000 -3.29480300 1.85628200

H -0.61527000 -4.40498700 3.73964200

H 0.63943800 4.75335300 2.99691400

H 1.61721300 -2.30969900 0.12561100

H 3.37693000 0.58850300 2.68913500

H 1.12381800 2.81802600 -1.23665400

H 4.43523900 0.71077100 0.42266700

H -1.33593900 -2.95934900 -0.75946400

H -4.62978600 -0.80996400 0.88536300

H -1.83798100 2.17150800 -0.05129000

H -3.40380600 -0.26257100 3.07471000

H -0.90164600 1.17916800 4.81568400

H 1.20829200 -0.66503500 4.81687600

C 2.47468600 4.63361400 -2.77679400

C 2.67194300 4.41510200 -4.29171700

C 0.96183600 4.80589300 -2.53801200

C 3.15434400 5.95269500 -2.34611200

H 3.71758600 4.49939300 -4.58892700

H 2.29615600 3.43240200 -4.60081500

H 2.10858400 5.17530700 -4.84658000

H 0.73332300 4.96893100 -1.48042100

H 0.61427100 5.68564100 -3.09078000

H 0.37522200 3.94982000 -2.89008000

H 2.74527700 6.78985200 -2.92516000

H 2.96733100 6.15521800 -1.28439300

H 4.23407300 5.92101900 -2.50589100

C 6.47089600 2.06360800 -0.58330700

C 6.60130200 2.03980100 0.96318700

C 6.95924000 0.70254700 -1.12457300

C 7.41923400 3.17951700 -1.06477900

H 6.20407800 2.96066200 1.40645700

H 6.08359500 1.19249600 1.42040400

H 7.65914100 1.95952400 1.23854700

H 6.94983700 0.67196400 -2.21813100

H 7.98802200 0.51519900 -0.79207500

H 6.33210700 -0.11902400 -0.76087400

H 8.40237900 3.01914800 -0.60625100

H 7.56530400 3.18986900 -2.14492500

H 7.06441300 4.16850700 -0.75698200

C -3.06621500 4.56028600 0.60295600

C -2.67161000 5.53000900 1.74002400

C -1.90718200 4.54992400 -0.41163400

C -4.31045200 5.10884900 -0.12730200

H -3.45977800 5.61207200 2.49203800

H -1.75531500 5.19105200 2.23545300

H -2.48504900 6.52995700 1.32880100

H -2.13297600 3.95153900 -1.30023600

H -1.72349100 5.57641600 -0.74757400

H -0.97990500 4.17815300 0.03670200

H -4.03979800 6.02796100 -0.66147400

H -4.68667500 4.39485200 -0.86739600

H -5.11702300 5.35489600 0.56426800

C -4.91136000 1.51175400 4.38944300

C -6.01661900 0.45120200 4.20638000

C -3.87359400 0.97636300 5.40819900

C -5.52345900 2.76668000 5.04291200

H -6.75910900 0.77045800 3.46663000

H -5.59926200 -0.50563400 3.87575700

H -6.53747700 0.27871200 5.15643300

H -3.08089100 1.71394700 5.57608300

H -4.36802700 0.78564300 6.36788700

H -3.40535300 0.04109200 5.08962200

H -5.84071600 2.50040400 6.05852000

H -4.79492700 3.57892600 5.12226500

H -6.40268800 3.14260000 4.51881200

C -6.67527500 -2.18095500 -0.12779900

C -6.81569800 -2.27118000 1.41275500

C -7.13536900 -0.77919600 -0.58009400

C -7.64003700 -3.23918100 -0.69817000

H -6.48676000 -3.25124200 1.77860400

H -6.23793800 -1.50432300 1.93295900

H -7.86660200 -2.13840200 1.69528900

H -7.04617700 -0.65809200 -1.66481100

H -8.18625600 -0.62193200 -0.30705300

H -6.54310700 0.00901700 -0.10347700

H -8.62247000 -3.08995100 -0.23408200

H -7.77924200 -3.16395300 -1.77704400

H -7.31076300 -4.25587700 -0.46298500

C -2.69908600 -4.77817800 -2.28736700

C -2.87568700 -4.56292500 -3.80569600

C -1.19235500 -4.96921100 -2.02733100

C -3.40756500 -6.08269700 -1.86038600

H -3.91853600 -4.64266900 -4.11397300

H -2.49433500 -3.58187100 -4.11325400

H -2.30989500 -5.32773300 -4.35175200

H -0.97912300 -5.13122400 -0.96598500

H -0.84395900 -5.85126900 -2.57566100

H -0.60062700 -4.11319300 -2.37191200

H -3.00832000 -6.92970900 -2.43191300

H -3.23856700 -6.28503700 -0.79573900

H -4.48445100 -6.02966100 -2.03468100

H -0.63084400 3.63896100 4.83852700

H 1.58672700 3.40305500 1.17412600

C 2.88846900 -4.56715900 1.09807400

C 2.67172200 -5.28330900 2.45046600

C 1.58558600 -4.71212500 0.28774200

C 3.99430200 -5.30146000 0.31306400

H 3.57366000 -5.25280800 3.06626600

H 1.85434900 -4.81640500 3.00961100

H 2.41015400 -6.33473800 2.27780300

H 1.68140200 -4.29853500 -0.72118600

H 1.34753100 -5.77628900 0.18591700

H 0.73913000 -4.22659100 0.78351400

H 3.61951900 -6.28013700 -0.01075100

H 4.28605600 -4.74356100 -0.58259400

H 4.88232400 -5.47896000 0.92141900

C 5.03967800 -1.04655900 4.13907600

C 4.73001300 0.37884200 4.63694500

C 4.59426200 -2.03101600 5.24425100

C 6.56916500 -1.13894700 3.96181300

H 5.06468300 1.14530600 3.92815500

H 3.66151600 0.52906900 4.82712100

H 5.26044600 0.54790900 5.58028100

H 4.81464100 -3.06540600 4.96872500

H 5.11885800 -1.80697600 6.18132700

H 3.51699200 -1.94572100 5.42937900

H 7.06198300 -0.72482000 4.84975900

H 6.90841700 -2.16959700 3.85183700

H 6.90718800 -0.56029100 3.09435200

O -5.10629700 3.92967700 2.56206100

O -5.47695900 -3.92268300 -2.32113900

O 5.27290100 3.85493300 -2.75179000

O 5.20041600 -3.57506100 2.62784900

C 6.33101700 -3.50894000 1.73900600

H 6.79578900 -2.51984800 1.76197500

H 7.04291300 -4.25933600 2.09090800

H 6.03867000 -3.73109100 0.70946800

C 5.69765700 3.05113100 -3.86037200

H 6.27510100 3.71007100 -4.51349800

H 6.32837300 2.21875600 -3.53743300

H 4.83918700 2.65314800 -4.41059700

C -6.40649900 3.66121900 2.01915500

H -6.85245800 2.76770400 2.46781200

H -7.02420200 4.53225600 2.25054300

H -6.35711100 3.52241700 0.93364900

C -5.83716700 -3.07563900 -3.42198900

H -4.94547700 -2.70725700 -3.93958900

H -6.42852300 -3.69117500 -4.10430100

H -6.43382900 -2.22016400 -3.09356000

Ni -0.12837200 -0.14255500 -0.76277500

C 2.03384500 -0.69158100 -2.87199000

C 2.25465400 -2.05708000 -2.63125900

C 3.51934500 -2.63244400 -2.76446200

C 4.60458500 -1.83701900 -3.15556100

C 4.39686200 -0.48009500 -3.43698600

C 3.13535800 0.07781400 -3.29143600

C 0.73299300 -0.03932700 -2.65365100

C -0.52474500 -0.70432900 -2.82142100

C -1.73519800 -0.03787700 -2.62994000

H -0.54237200 -1.77897000 -2.99404200

H 0.73607100 1.03613900 -2.82532000

H 1.43109900 -2.68436400 -2.30303800

H 2.99676300 1.13566900 -3.48780500

H 3.64729600 -3.68686100 -2.55095600

H 5.23873900 0.12366900 -3.75713300

O 5.89024700 -2.28353300 -3.29197200

C 6.16582000 -3.63604400 -2.93738500

H 7.23500100 -3.77786300 -3.10797700

H 5.93773200 -3.82247400 -1.88096100

H 5.60236500 -4.33963100 -3.56254200

H -1.75368600 1.02518600 -2.42592200

H -2.65497300 -0.59461300 -2.52150100

C -2.54755400 0.94458200 -4.89139900

C -3.12167100 1.94073600 -4.10534200

C -4.59891000 1.83142900 -3.66472300

C -4.70650400 1.65828200 -2.13914100

O -2.48552200 2.95981000 -3.64915700

H -3.17482400 0.13962500 -5.26252300

H -5.02615900 2.81806300 -3.90195000

H -5.73472600 1.82997500 -1.79729500

H -4.42306300 0.64595900 -1.82949100

H -4.05568100 2.36274800 -1.61824800

C -1.18142100 1.07227700 -5.49765600

H -1.21777800 1.24042100 -6.58717800

H -0.64423900 1.91848500 -5.05555600

H -0.56363200 0.17349200 -5.34424900

C -5.44057200 0.76936400 -4.37829100

H -5.40982800 0.88785400 -5.46763400

H -5.09261100 -0.24258200 -4.13946600

H -6.48995000 0.83784300 -4.06372500
